# Voltage imaging of neurons distributed across entire brains of larval zebrafish

**DOI:** 10.1038/s41592-026-03179-7

**Published:** 2026-08-14

**Authors:** Zeguan Wang, Jie Zhang, Panagiotis Symvoulidis, Wei Guo, Davy Deng, Adam Amsterdam, Lige Zhang, Takato Honda, Steven Roche, Matthew A. Wilson, Edward S. Boyden

**Affiliations:** 1 McGovern Institute for Brain Research, MIT, Cambridge, MA USA; 2 Department of Media Arts and Sciences, MIT, Cambridge, MA USA; 3 Picower Institute for Learning and Memory, MIT, Cambridge, MA USA; 4 Department of Brain and Cognitive Sciences, MIT, Cambridge, MA USA; 5 Harvard-MIT Health Sciences and Technology, Cambridge, MA USA; 6 David H. Koch Institute for Integrative Cancer Research, MIT, Cambridge, MA USA; 7 Department of Biological Engineering, MIT, Cambridge, MA USA; 8 Mindspan Institute, Cambridge, MA USA; 9 Center for Neurobiological Engineering, Yang Tan Collective, and K. Lisa Yang Center for Bionics at MIT, Cambridge, MA USA

**Keywords:** Optogenetics, Light-sheet microscopy

## Abstract

Neurons interact in networks distributed throughout the brain. While much effort has focused on whole-brain calcium imaging, advances in genetically encoded voltage indicators raise the question of whether it might be possible to image neuronal voltage across entire brains. Achieving this requires a microscope with high volumetric imaging rates and signal-to-noise ratio. Here we present a remote-scanning light-sheet microscope capable of imaging genetically encoded voltage indicator-expressing neurons distributed throughout much of the brain of larval zebrafish at a volumetric rate of 200.8 Hz. We measured voltage traces from approximately one-quarter of all brain neurons. We found that neurons firing at different times during a sequence occupied different locations: visually evoked sequences mapped across the optic tectum, whereas stimulus-independent bursts were mapped across the cerebellum and medulla. Imaging voltage of neurons distributed in many brain regions may open new frontiers for understanding fundamental neural system operations.

## Abstract (web summary)

Voltage imaging across much of the zebrafish brain is achieved at 200.8 Hz, by optimizing a remote-scanning light-sheet microscope.

## Main

Advances in optical microscopy and genetically encoded calcium indicator engineering have enabled the imaging of calcium, a proxy for neural activity, across the entire brains of larval zebrafish^1–4^, an important model organism in neuroscience; however, the temporal resolution of whole-brain calcium imaging, typically around several hundreds of milliseconds^1,4,5^, is insufficient to reveal millisecond-scale neural processes. Genetically encoded voltage indicators (GEVIs) offer the promise of imaging neural voltage from many neurons in parallel. Yet, simultaneously meeting all the technical requirements for imaging GEVI-expressing neurons distributed across entire brains is challenging. The microscope must achieve temporal resolution comparable to millisecond-scale action potential timing, while having a three-dimensional field of view (3D-FOV; defined as the 3D volume that a microscope can cover) sufficient to cover the whole larval zebrafish brain, together with high enough spatial resolution to resolve single cells, and high enough signal fidelity to detect individual action potentials. Optimizing for one goal often degrades the microscope’s performance in another. We here ask whether a microscope can be designed to cross the threshold of imaging voltage of neurons distributed across much of the zebrafish brain by satisfying all these criteria simultaneously.

Light-field microscopy (LFM)^3,6^ provides high volumetric rates and single-shot 3D acquisition, but two intrinsic limitations hinder dense whole-brain voltage imaging. First, resolution degrades as axial range increases: when capturing the full ~200-µm brain thickness in one snapshot, reported lateral resolution is ~3 µm (refs. ^7,8^), insufficient to visualize densely packed cells. Although optical sectioning or scanning can improve resolution^9,10^, doing so requires multiple snapshots and reduces volumetric rate. Second, LFM mixes fluorescence from many *z*-planes, increasing the shot noise and lowering the signal-to-noise ratio (SNR). For dense whole-brain imaging in larval zebrafish, we estimate ≥4× higher shot noise than light-sheet microscopy (LSM) because LFM baselines integrate ~200 µm of tissue versus less than 12 µm for LSM (Supplementary Note 1).

LSM offers strong optical sectioning, low phototoxicity and diffraction-limited lateral resolution. Conventional whole-brain light-sheet calcium imaging in zebrafish achieves ~0.8–3 Hz (refs. ^1,4^), but scaling conventional LSM to hundreds of volumes per second while maintaining whole-brain 3D-FOV is limited by (1) axial scanning mechanisms (objective translation is hard to push to >100 Hz over >200 µm) and (2) camera pixel throughput at the required camera FOV (C-FOV; defined as the number of row and column pixels captured in one frame). With widely used state-of-the-art scientific cameras (for example, Hamamatsu ORCA-Flash, Andor Zyla), a 512 × 1,280 C-FOV yields only ~400 Hz frame rate, implying ~10–15 Hz for ~30 planes.

Multiple approaches have been proposed to enhance LSM speeds. Remote refocusing^11^ can rapidly shift focus using tunable lenses^12^, moving tertiary objectives^13^ or translating mirrors^14,15^, but tunable lenses add aberrations that constrain numerical aperture (NA) and two-dimensional FOV (2D-FOV; defined as the area a microscope can image in a single 2D focal plane), and heavy moving components limit scanning speeds. SCAPE/oblique plane microscopy (OPM)^16–20^ can achieve high volume rates via remote lateral sweeping, but effective NA and/or aberrations can constrain light efficiency and diffraction-limited 2D-FOV; recent glass-tipped tertiary designs^18^ enabled an 800 × 420 µm^2^ 2D-FOV at NA = 0.97 in OPM and suggest a possible path toward fast whole-brain imaging with further optimization (Supplementary Note 1).

Here we optimized translational-mirror remote refocusing to cross the feasibility threshold for voltage imaging of neurons distributed across much (~85% volume) of larval zebrafish brains. Our method promises comparable or lower aberrations (as it uses one fewer relay lens pair between the detection and remote objectives) and samples the fish brain in a way closer to conventional LSMs compared to SCAPE/OPMs. We combined a lightweight silver-coated mirror (~0.01 g) with a piezo bender actuator (270 µm travel, 930 Hz resonance and sub-ms response) to scan ~200 µm axially at up to 300 Hz. Using optical simulations, we selected tube lens assemblies and relay optics to minimize aberrations while preserving a large 3D-FOV at NA = 1.0. We paired this with high-throughput cameras and distributed planar imaging^21^ to sustain more than six kilohertz frame rates at brain-scale C-FOV. By incorporating a second remote-refocusing module, we could efficiently capture emission light of all polarizations, improving fluorescence collection by 75% in voltage imaging experiments.

A common practical challenge in zebrafish light-sheet imaging is blood-flow-induced moving-stripe artifacts^22,23^. In calcium imaging, these artifacts are largely averaged out due to low imaging rates (typically a few hertz) and are eclipsed by large calcium signals (Δ*F*/*F* often >50%). By contrast, voltage imaging operates at hundreds to thousands of hertz and relies on much smaller signals (Δ*F*/*F* ≈ 10% in vivo for Positron2-Kv), making it highly sensitive to such artifacts. While stopping the heart has been one way that multiple teams have dealt with this problem in the context of behavior^24,25^, we asked whether there is a simpler, for example software-only, way of doing so. We here showed three methods to address the moving stripes: transient heart arrest, resonant scanning and a computational method.

Using our microscope, we imaged voltage activity of neurons distributed across larval zebrafish brains expressing pan-neuronal Positron2-Kv^26,27^ at 200.8 Hz. The microscope covered ~85% of the brain volume in 5 days-post-fertilization (dpf) zebrafish, with the remainder either shadowed by the eye, or affected by light scattering (problems not unique to voltage imaging and also present in light-sheet calcium imaging^1^). Across four fish (fish A–D), we segmented 17,419–25,552 regions of interest (ROIs) corresponding to putative neurons using a custom-trained Cellpose v.3.0 model^28^. After computationally excluding regions corrupted by blood-flow-induced moving stripes, we retained 12,935–19,039 putative neurons (59–92% yield), up to ~one-quarter of the estimated ~78,000 neurons in a 7-dpf fish^29^.

To illustrate the kinds of observations enabled by our technique, we delivered a UV stimulus and identified a stimulus-onset responsive group of neurons concentrated in the optic tectum (OT). Sorting neurons by peak firing-rate latency revealed a reproducible temporal sequence (~200 ms) whose ordering correlated with OT medial–lateral position: lateral OT neurons fired earlier than medial OT neurons (both sides of OT in fish A, B, D and one side in fish C). We also identified a stimulus-independent bursting group in cerebellum/medulla with consistent intra-burst sequential activation; in two fish (fish B and C), a putative anatomical cluster of neurons at the ventral side of the medulla oblongata consistently fired at the beginning of bursts. These results demonstrated the capabilities of our technique to study large-scale, millisecond-level neural processes in the brain. Voltage imaging of neurons distributed across much of the entire brain may be useful for generating many new kinds of hypotheses, which could be tested by further neuroscience experiments in the future.

## Results

### Microscope design and implementation

**Fig. 1:**
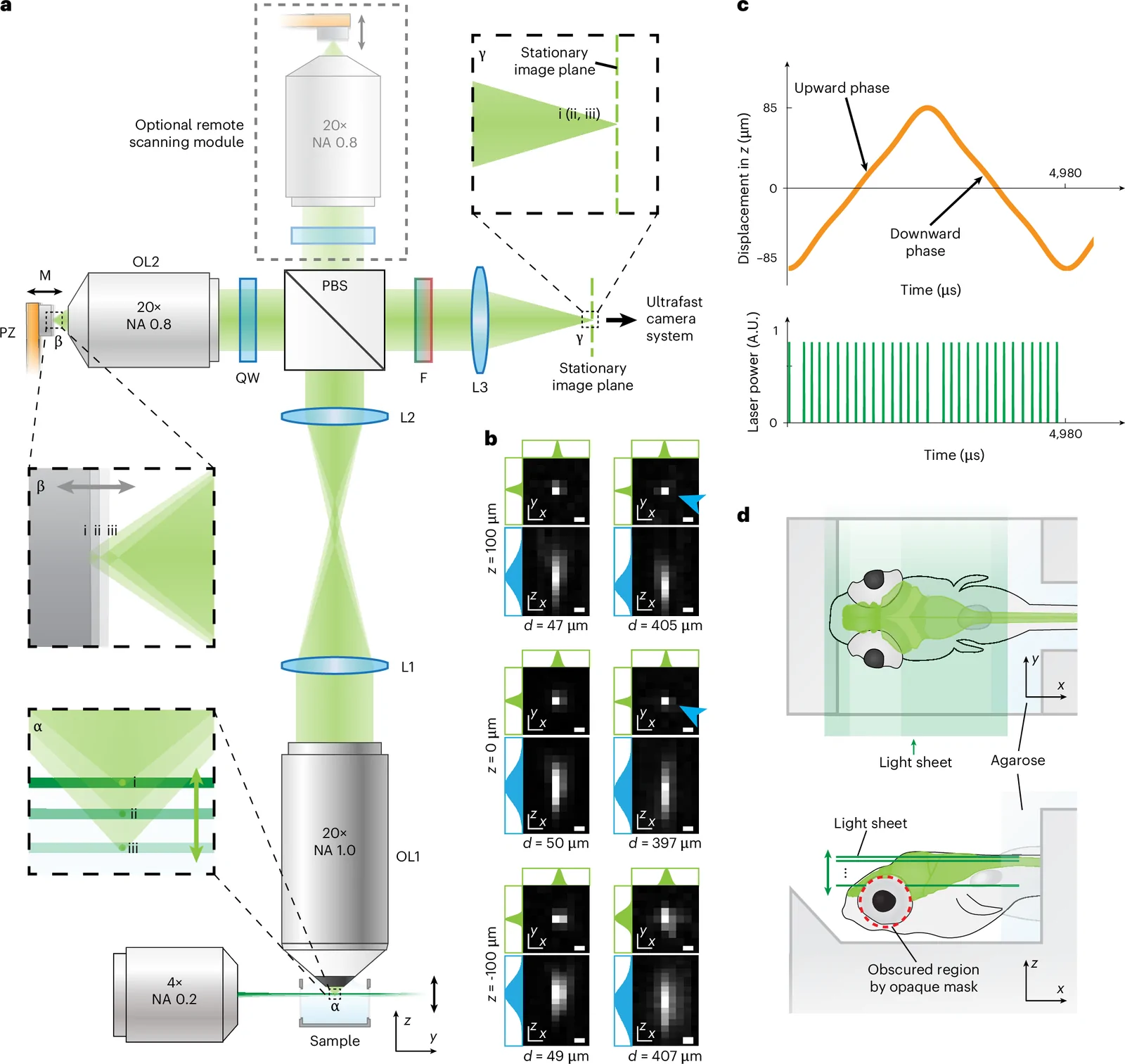
Remote-scanning light-sheet microscopy for voltage imaging of neurons distributed across much of the larval zebrafish brain. **a**, Overview and operational principles of our remote-scanning LSM optimized for volumetric voltage imaging of neurons distributed across much of the larval zebrafish brain. An infinitesimal fluorescent bead (used here for illustration) in a water chamber is illuminated laterally by a rapidly scanned light sheet. Fluorescence is collected orthogonally by a ×20 water-immersion objective (OL1, NA = 1.0) and relayed through a 4f system (tube lenses L1 and L2) to a PBS. The PBS directs one polarization through a quarter-wave plate (QW) into a remote air objective (OL2, ×20, NA = 0.8), which forms a real image. This image is reflected by a mirror (M) mounted on a piezo actuator (PZ) and reimaged by OL2. After a second pass through the QW, the fluorescence polarization is rotated by 90°, allowing it to transmit through the PBS, pass an emission filter (F), and be focused by tube lens L3 onto a stationary image plane (green dashed line). Synchronous translation of the remote mirror with light-sheet scanning ensures that fluorescence from different sample *z*-planes remains in focus at the stationary focal plane of L3, where images are recorded by an ultrafast camera system (Extended Data Fig. 1b–d). An optional second remote-scanning module (gray dashed box above the PBS) can be added to improve light efficiency. To illustrate this imaging process, magnified views of the sample (α), remote mirror (β), and stationary image plane (γ) are shown at three time points (i–iii). At the sample, the light sheet excites the bead at different axial positions, and emitted fluorescence is collected by OL1. At the remote side, the mirror translates in synchrony with the light sheet, reflecting fluorescence back into OL2. At the image plane, fluorescence from different time points is refocused onto the same stationary plane. **b**, Measured PSFs across the microscope’s 3D-FOV using 200-nm-diameter fluorescent beads. Each subpanel shows the lateral (PSF_*xy*_, top) and axial cross-sections (PSF_*xz*_, bottom). PSF_*xz*_ was fitted with a 2D Gaussian function; line profiles along *x* and *y* through the Gaussian function center (green shadings) are shown above and to the left. The axial line profile through the brightest pixel of PSF_*xz*_ was fitted with a one-dimensional double Gaussian function (cyan shadings shown at left). Rows correspond to three axial positions (*z* = 100 µm, 0 µm, −100 µm). For each *z*, randomly selected PSFs near the 2D-FOV center and periphery are shown, with distance from the center denoted by *d*. PSFs were acquired by accumulating 40-µs exposures, while continuously scanning across a 206-µm thick volume at 200.8 Hz. Cyan arrows indicate under-sampled PSF_*xy*_ due to the camera pixel size (0.73 µm). Scale bars, 1 µm. **c**, Synchronization of focal-plane scanning and pulsed light-sheet excitation. The axial position of the microscope focal plane during one scan cycle (4.98 ms) is shown as the orange curve. Green lines indicate 40-µs light-sheet pulses exciting individual *z*-planes. Pulse timing was calibrated to evenly sample the *z* axis. Each scan cycle includes upward and downward phases of focal-plane motion, and 30 evenly spaced *z*-planes are sampled once per cycle in an interleaved acquisition scheme. **d**, Larval zebrafish preparation for light-sheet imaging. A pan-neuronally labeled larval zebrafish is immobilized on a 3D-printed holder, with the body embedded in agarose and the head exposed. A laterally incident light sheet scans the brain along the *z* axis. Excitation light at the eyes is blocked by a circular opaque mask (red dashed line). Top and side views are shown.

To enable 3D, large-volume, high-speed, cellular-resolution voltage imaging of the larval zebrafish brain, we developed a remote-scanning LSM (rsLSM) optimized across optical design, mechanical scanning, detection hardware and system control (Fig. 1). The design target was imaging over whole zebrafish brain volume at >200 volumes per second (VPS), with spatial resolution sufficient to resolve individual neuron somas and temporal resolution compatible with GEVIs.

A central design challenge was increasing the volumetric scan rate of LSM without sacrificing optical performance. We adopted and extended a translational-mirror-based remote refocusing strategy^11,14,15^, in which axial scanning is performed at a remote image plane rather than by mechanically translating the detection objective. Unlike previous implementations, we employed a custom lightweight silver-coated mirror with a mass of ~0.01 g and actuated it using a piezo bender scanner with a large travel range (270 µm), high-resonant frequency (930 Hz), and submillisecond response time. This combination enabled scanning over a 200-µm axial range at the sample at rates up to 300 Hz, supporting volumetric imaging well above 200 VPS.

To preserve spatial resolution across the large 3D-FOV, we carefully optimized the detection optics. Although ideal remote refocusing is theoretically near aberration-free, in practice, accumulated aberrations from multiple high-NA optical components can substantially degrade resolution at the edges of the field, particularly along the ~900-µm rostral–caudal (*x*) extent of the zebrafish brain. We therefore used Zemax to simulate wavefront aberrations across a Φ900 × 200 µm^3^ 3D-FOV for a series of candidate optical configurations. Because Zemax models were unavailable for detection and remote objectives, simulations focused on the remaining detection-path elements, including three tube lenses, a polarizing beam splitter (PBS) and a relay lens pair. The relay lenses reduced the system magnification to ~×6.16 so that the full brain volume could fit within the C-FOV while maintaining high imaging speed.

These simulations revealed that tube lens selection was important in aberration control. Borrowing from earlier oblique plane microscopy (OPM) designs^18^, we constructed custom tube lenses by assembling multiple commercially available lenses to achieve the required focal lengths and apertures. The optimized configuration supported a diffraction-limited Φ900 × 200 µm^3^ 3D-FOV at NA = 1.0, with a wavefront error of 0.15λ RMS at 550 nm at the edge of the FOV. Although the objectives themselves were not included in the simulations, minimizing aberrations contributed by the remaining optics could substantially improve performance in the real system, due to the additive nature of aberrations in imaging systems^30^.

With the optical design established, we next addressed detection speed and sensitivity. In LSM spatial resolution and volumetric scan rate are jointly constrained by camera frame rate and C-FOV, which are inversely related to each other (a typical scientific camera’s frame rate is inversely proportional to the row number of C-FOVs). To image the entire zebrafish brain in a single plane, the camera must have a sufficiently large C-FOV (for example, 512 × 1,280) to sample a 2D-FOV of 900 × 370 µm^2^ with an effective pixel size smaller than a neuron soma (6.62 µm mean diameter^1^). On the other hand, the camera’s frame rate determines the LSM’s volume scan rate and *z*-plane step size. For example, at a fixed camera frame rate of 6,000 Hz, scanning at 300 VPS yields 20 *z*-planes per volume over a 200-µm thickness (10-µm *z*-steps), whereas scanning at 200 VPS yields 30 *z*-planes per volume (6.7-µm *z*-steps).

Conventional sCMOS cameras (for example, Hamamatsu ORCA-Flash, Andor Zyla) are limited to ~400 Hz at this C-FOV, which is insufficient for volumetric voltage imaging. Faster cameras such as the Lambert HiCAM Fluo and Teledyne Kinetix reach 3,000–4,000 Hz but suffer from either low quantum efficiency (QE 30–50%) or low full-well capacity (~200 e⁻) incompatible with the photon flux required for our application, where the brightest pixels can generate up to ~2,000 electrons per frame.

**Extended Data Fig. 1:**
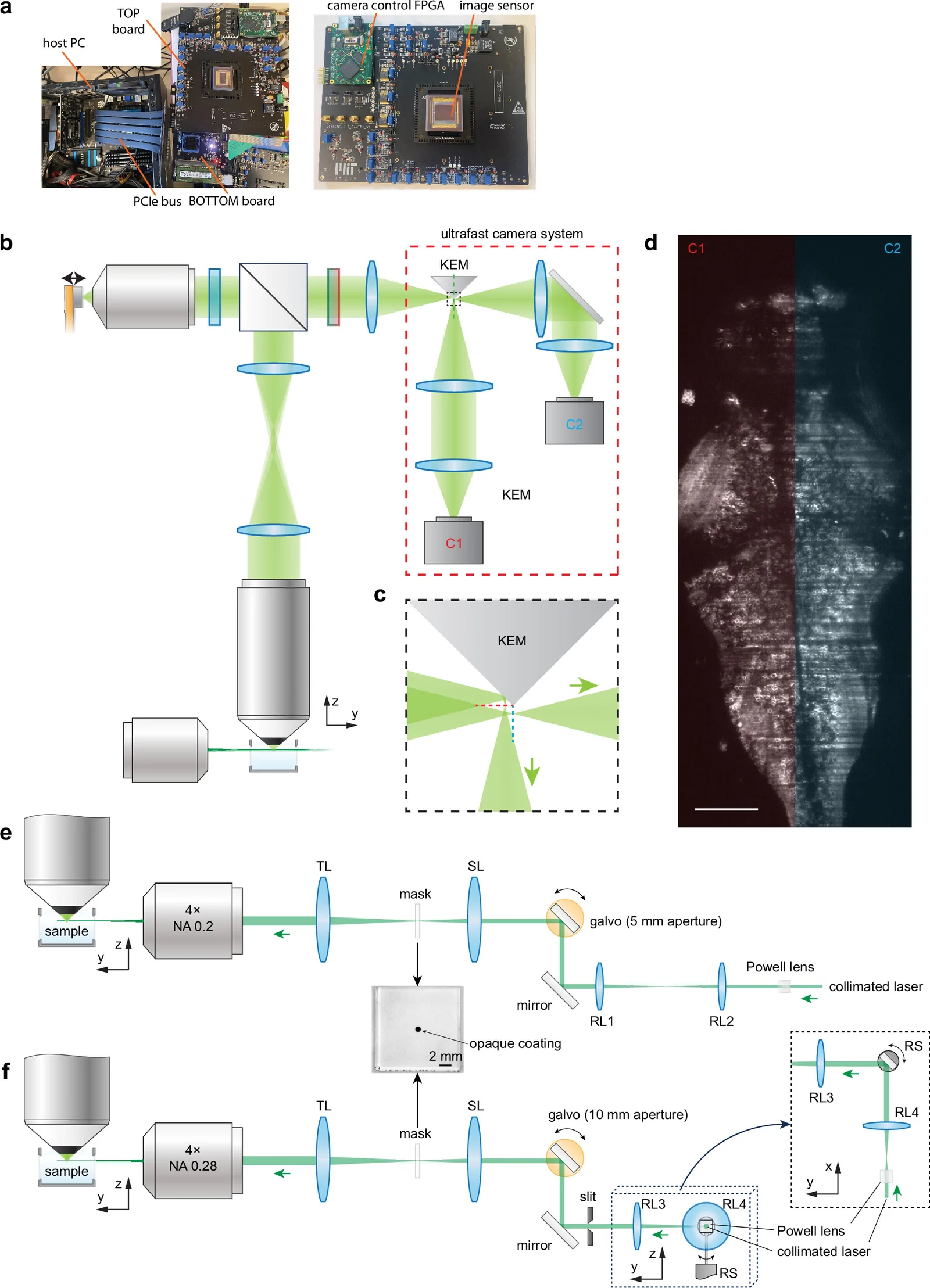
Ultrafast camera architecture and excitation light-path configurations. **(a)** Customized high-speed camera using GSPRINT sensors used for early testing. The camera system (left) adopts a two-board stack design. The TOP board (right) hosts the image sensor and uses an FPGA (Xilinx Spartan 6, Opal-Kelly XEM6010) to handle camera control and trigger synchronization. The BOTTOM board uses an FPGA (Xilinx Kintex-7) with external DDR RAM to buffer and transfer high-bandwidth data (2.6GBps) to the host computer through a PCIe bus. **(b-d)** Ultrafast camera system for image acquisition. **(b)** Images from the light-sheet microscope are recorded by an ultrafast camera system (red dashed box) consisting of an image splitter and two commercial ultrafast cameras equipped with GSPRINT4502 sensors. The focused image from the microscope is divided into two halves by a knife-edge mirror (KEM) and relayed through two identical lens pairs to cameras C1 and C2 for simultaneous recording. **(c)** Enlarged view of the black dashed box in (b) at the KEM, showing the KEM deflecting the upper half of the image into a separate optical path. **(d)** Raw images from cameras C1 (red shading) and C2 (blue shading) are stitched to reconstruct a complete zebrafish brain section. Scale bar, 100 µm. **(e, f)** Excitation light-path configurations. **(e)** Configuration 1 (without light-sheet pivoting). A collimated laser beam enters from the right, first passing through a Powell lens and then a pair of relay lenses (RL1, RL2). After deflection by a galvo scanner, the excitation light passes through a scan lens (SL), forming an enlarged light sheet near its focal plane. This enlarged light sheet is then relayed through a 0.25× microscope consisting of a tube lens (TL) and a 4× objective lens to illuminate the sample. An optical mask placed at the SL focal plane prevents excitation light from directly entering the fish’s eyes. **(f)** Configuration 2 (with light-sheet pivoting for stripe-artifact suppression). This configuration is largely identical to configuration 1, with the following modifications: 1) a resonant mirror scanner is inserted between the relay lenses (RL3, RL4) to pivot the light sheet in the focal plane; 2) a larger-aperture galvo scanner (10 mm) is used to accommodate excitation light rapidly scanned by the resonant scanner over an ~8 mm range on the galvo; 3) the illumination objective is replaced with a lens with higher numerical aperture and smaller focal length (NA = 0.28, f = 45 mm); 4) relay lenses with adjusted focal lengths (RL3, f = 100 mm; RL4, f = 75 mm) are used to match the aperture of the resonant scanner; and 5) an adjustable slit is inserted between the galvo scanner and RL3 to control light-sheet thickness and divergence. A top view of the resonant scanner section, including the resonant scanner, relay lenses, and the Powell lens, is shown in the dashed box on the right.

We therefore adopted the Gpixel GSPRINT4502 CMOS image sensor, which supports a 4.37-GHz pixel rate in 10-bit mode, with a full-well capacity of 7.4 ke⁻, readout noise of ~7 e⁻ and ~60% QE at 550 nm (Extended Data Fig. 1a). Using this sensor, we designed a camera around it, and achieved a frame rate of 3,900 FPS for a 512 × 1,280 C-FOV, sufficient to cover the entire larval zebrafish brain. To further increase imaging speed, we implemented a distributed planar imaging strategy^21^, splitting the 2D-FOV across multiple sensors. Operating two commercial cameras equipped with GSPRINT4502 sensors in parallel increased the effective frame rate to 7,300 Hz for a C-FOV of 512 × 1,280 pixels, which in principle, can enable the LSM to scan 30 image planes at a maximum volume rate of 243 VPS. (Extended Data Fig. 1b–d)

At the system level (Fig. 1a), the sample was excited by a laterally illuminated 515-nm light sheet generated using a Powell lens and scanned by a galvo mirror (Extended Data Fig. 1e,f). Emitted fluorescence was collected by a water-immersion detection objective (NA = 1.0) and reimaged by an air remote objective (NA = 0.8). The remote image was reflected by the piezo-actuated lightweight mirror, effectively treating the remote objective as a virtual tertiary lens whose axial position could be scanned rapidly. The magnification (Mag) between the sample and remote image was set to 1.33, matching the refractive index ratio between water and air ($\mathrm{{Mag}}_\mathrm{{remote}}={n}_\mathrm{{sample}}/{n}_\mathrm{{remote}}$), which eliminates spherical aberration during refocusing^11,31^.

Because fluorescence passes through the PBS, emission light of one polarization will be lost in remote refocusing with one remote-scanning module. Although we did not find signal levels were limiting, improved efficiency would benefit SNR, reduce excitation power and slow photobleaching. We therefore restored lost fluorescence by adding an identical remote-scanning module (remote objective, quarter-wave plate and piezo-driven mirror) at the orthogonal PBS port and synchronizing the two piezo scanners (Fig. 1a). The two images were aligned on the sensor with subpixel precision across the full 200-µm axial range (Supplementary Fig. 1). In zebrafish voltage imaging experiments, this dual-module configuration increased fluorescence intensity by 75% (Supplementary Fig. 2). The enhancement was less than 100% because GEVI emission was partially polarized due to linearly polarized excitation^32^, and the PBS orientation was chosen such that ~57% of emission was already directed to the primary module.

We empirically characterized system resolution by imaging 200-nm diameter fluorescent beads (605-nm emission) embedded in agarose while continuously scanning across a 206-µm axial range at 200.8 Hz. Red beads were chosen to avoid resolution overestimation and because green beads could not be efficiently excited at 515 nm. Due to the low system magnification (×6.16), the lateral effective pixel size (0.73 µm) under-sampled the true point spread functions (PSF), occasionally collapsing it into a single bright pixel (Fig. 1b). We therefore estimated lateral full width half maximum (FWHM) by fitting a 2D Gaussian to the downsampled PSF (Supplementary Fig. 3a; Supplementary Note 2) and axial FWHM by fitting a dual-Gaussian model to the *z*-profile (Supplementary Fig. 3). Across the full Φ900 × 200 µm^3^ FOV, we measured lateral and axial FWHMs of ~1 µm and ~3.5 µm, respectively (Supplementary Fig. 3b–f). These values exceed theoretical limits (0.37 µm lateral and 1.21 µm axial) due to motion blur from continuous scanning (~3 µm axial travel per frame) and pixel undersampling. Nevertheless, spatial resolution across the volume was ultimately limited by the effective pixel size and *z*-sampling step (5–6 µm). By Nyquist’s criterion, the 0.73-µm pixel size corresponds to an effective lateral resolution of 1.46 µm, ~one-fifth of the average larval zebrafish neuron soma diameter (6.62 µm^1^), sufficient for cellular resolution.

For system operation, we developed a synchronized hardware control system with 4-µs temporal precision. Because of the high scan rate, we used open-loop piezo control supplemented by an image-guided calibration pipeline. A custom 20× magnification line-camera imaging system measured mirror displacement at 70 kHz with 350-nm precision, providing feedback to tune FPGA-generated control signals. After calibration, the piezo scanned a 113-µm range (170 µm at the sample) at 200.8 Hz with a near-triangular displacement waveform composed of the first three Fourier components (200.8, 602.4 and 1,004 Hz; Fig. 1c) of the triangle wave. Compared with sinusoidal motion, this waveform provided more uniform *z*-plane sampling and more efficient use of camera speed.

The galvo-scanned light sheet was synchronized to the moving focal plane by iteratively adjusting control signals while imaging fluorescent beads and stroboscopically exciting individual *z*-planes. Each scan cycle included both upward and downward phases, which we exploited using an interleaved sampling strategy to image 30 evenly spaced *z*-planes from −85 to +85 µm (Fig. 1c). To minimize motion blur, the light sheet was flashed for 40 µs per frame (43 mW during the 40-µs excitation pulse), during which the focal plane moved ~2.7 µm, approximately half the measured light-sheet thickness (5.3 ± 1.4 µm FWHM, mean ± s.d., across 400 µm along the illumination (*y*) axis).

### Characterization and mitigation of stripe artifacts

**Fig. 2:**
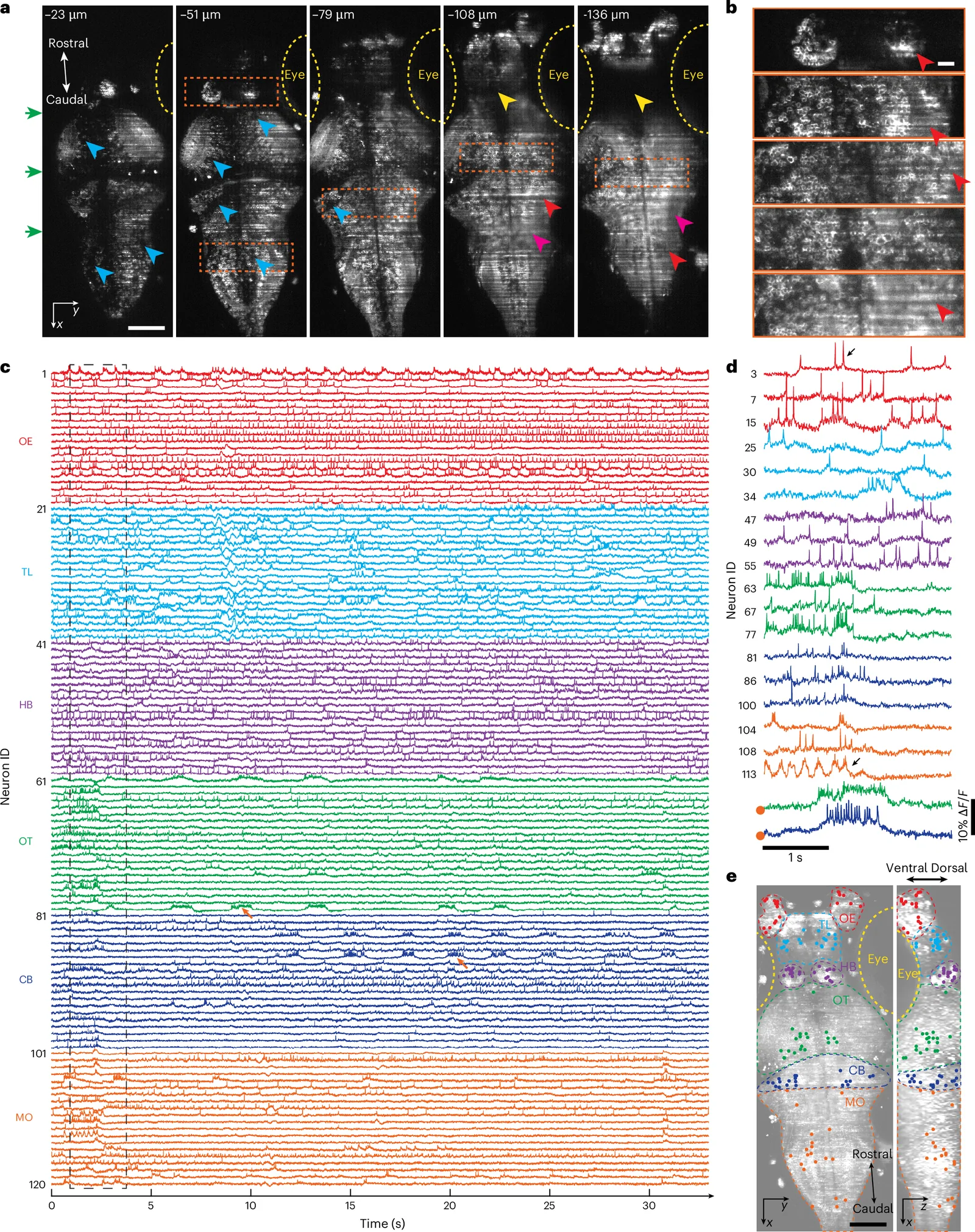
Voltage imaging of neurons distributed across much of the larval zebrafish brain at cellular resolution. **a**, Stitched raw images from a brain-wide voltage imaging experiment, showing 5 zebrafish brain sections out of a total of 30. Each section was imaged with 40-µs excitation at a 200.8 Hz imaging rate. Green arrows on the left indicate the direction of light-sheet illumination; cyan arrows mark examples of unlabeled brain regions; yellow dashed lines outline the eyes; yellow arrows indicate brain regions shadowed by the eyes; red arrows highlight light-sheet stripe artifacts (addressed computationally as described in Supplementary Note 4); magenta arrows indicate blurred regions. Six fish were imaged in the same session, and data from one randomly selected fish are processed and shown. Scale bar, 100 µm. **b**, Enlarged views of regions outlined by dashed rectangles in **a**. Scale bar, 20 µm. **c**, Spontaneous voltage activity traces from 120 exemplar neurons across six brain regions. Colors denote regions (referenced in **e**): OE (red), TL (cyan), HB (purple), OT (green), CB (blue) and (orange). Orange arrows indicate two randomly selected burst events. ROIs affected by moving-stripe artifacts were computationally identified and excluded; all traces shown correspond to putative neurons unaffected by stripe artifacts. **d**, Expanded views of the traces enclosed by the dashed rectangle in **c** and the two burst examples indicated by orange arrows. **e**, Spatial locations of the putative neurons (colored dots) corresponding to the activity traces in **c**, overlaid on the dorsal (left) and lateral (right) maximum-intensity projections (MIPs) of the imaged brain. Neuron colors match those of their respective traces. Brain regions are delineated by dashed outlines using the same color scheme as in **c**. Scale bar, 100 µm. Source data

In raw light-sheet voltage imaging data from larval zebrafish, we consistently observed bright and dark stripe-shaped patterns oriented parallel to the illumination axis of the light sheet (*y* axis; Fig. 2a,b). Such stripe artifacts are a common issue of LSM when imaging nonfully transparent samples, where absorption, refraction, or scattering of the excitation sheet perturbs its propagation^22,23^. Although larval zebrafish brains are translucent, they are optically heterogeneous: refractive index mismatches at the skin–water interface, nonuniform brain tissue, residual opaque structures, and circulating blood cells can all distort the light sheet. Blood cells in particular may act as dynamic microlenses^33^, converging or diverging excitation light and casting stripe-shaped shadows that extend along the illumination direction.

Across datasets, stripe artifacts were distributed throughout the brain but differed in their temporal behavior. In a substantial fraction of affected regions (ranging from ~50% to nearly 100% across animals), stripe patterns were stationary over time. These stationary stripes altered baseline fluorescence intensity but did not introduce temporal artifacts, allowing neural activity to be reliably extracted despite changes in signal amplitude or SNR. In contrast, a variable subset of regions (from near 0% up to ~50% of the brain, depending on the animal) exhibited dynamically ‘moving’ or ‘flickering’ stripes (Supplementary Video 1 and Supplementary Fig. 4). These moving stripes caused rapid baseline fluctuations in fluorescence traces, producing pulse-like temporal artifacts that could mimic or obscure neural spikes (Supplementary Figs. 4c and 5).

**Extended Data Fig. 2:**
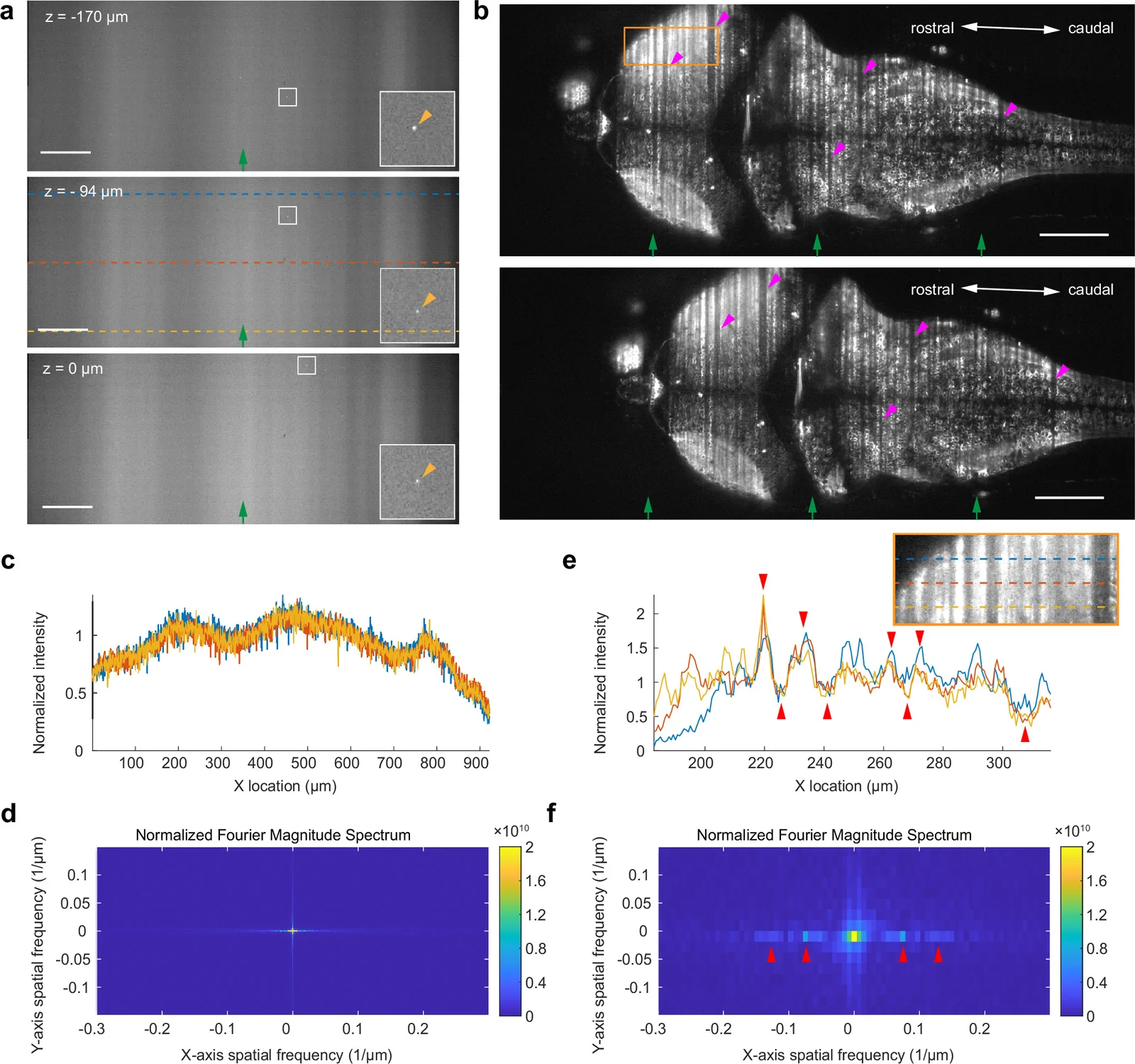
Control experiments examining whether stripe artifacts arise from the setup or the animal. **(a)** Raw light-sheet images of an agarose sample containing JF525 dye. Single-plane images at different depths (z) are shown. Green arrows denote the direction of light sheet illumination. The agarose also contained sparse 200-nm diameter fluorescent beads. Their images (orange arrows) were visible in the enlarged boxes. The intensity profiles along three lines (blue, red, and orange dashed lines) in the central image were plotted in (c). Scale bar: 100 µm. **(b)** Raw single-plane images of a zebrafish brain acquired while translating the fish along the x-axis (horizontal axis in these images). Green arrows denote the direction of light sheet illumination. The orange-boxed area in the top image was used for profile plot and Fourier analysis in (e, f). The orange-boxed area avoids fine neuron soma boundaries, which possess high spatial frequencies that could confound the Fourier analysis of stripe artifacts. Scale bar: 100 µm. **(c)** Intensity profiles along three lines (blue, red, and orange) in the central image of panel (a) normalized by their mean values. **(d)** Magnitude spectrum of the two-dimensional Fourier transform of the central agarose image shown in panel (a), normalized by its mean value. **(e)** Intensity profiles along three lines (blue, red, and orange dashed lines) within the orange-boxed area (zoomed in at the top right corner) of the top image in panel (b), normalized by their mean values. Red arrows show the locations of bright and dark stripes. **(f)** Magnitude spectrum of the two-dimensional Fourier transform of the orange-boxed area in panel (b), normalized by its mean value. Red arrows highlight high spatial frequency components corresponding to stripe artifacts.

**Extended Data Fig. 3:**
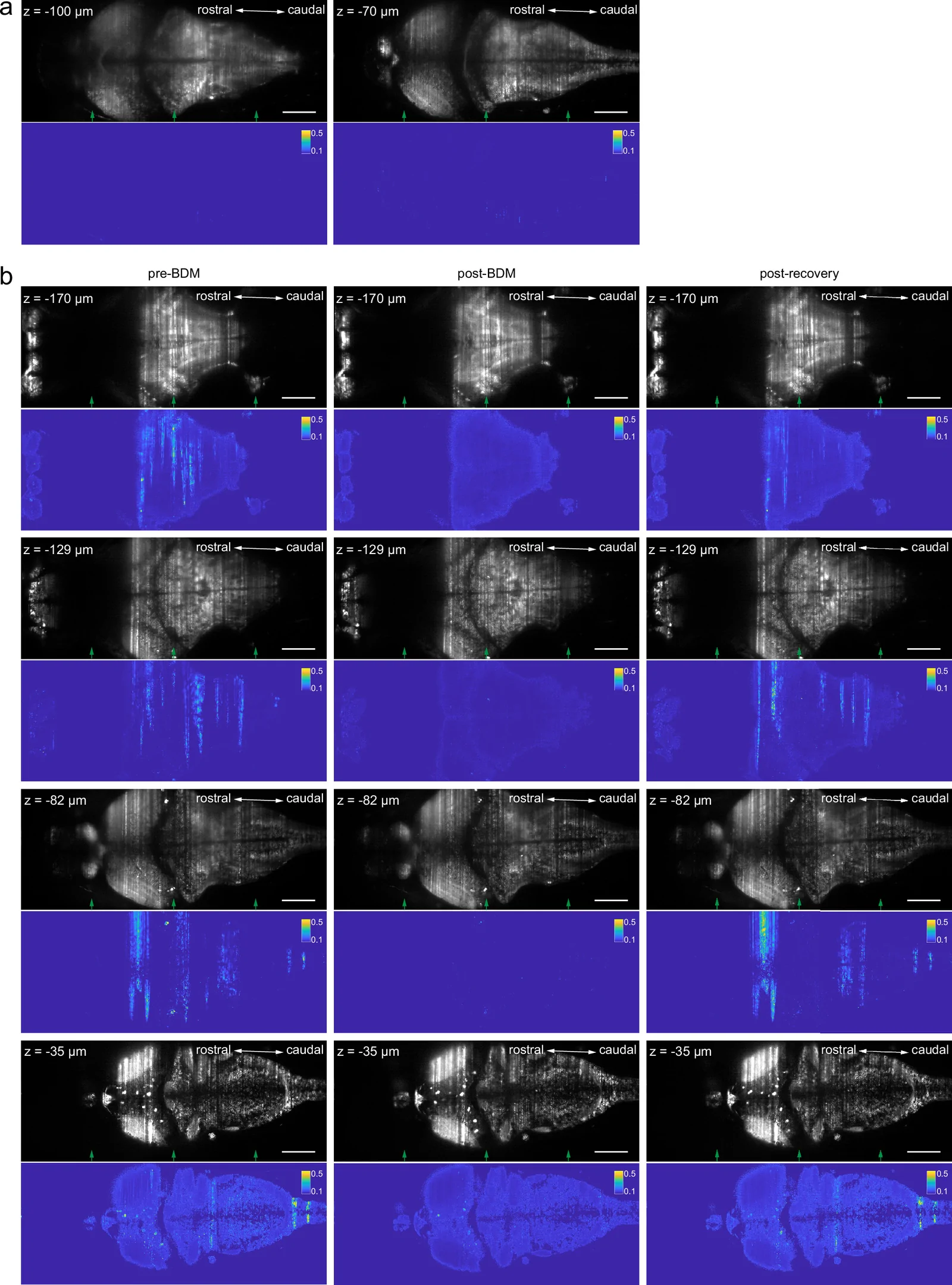
Imaging fixed fish and BDM-treated fish to test the origin of moving-stripe artifacts. **(a)** Raw videos of the fixed fish brain were recorded without resonant scanning. The top row displays two randomly selected planes of a fixed brain, with green arrows indicating the direction of light-sheet illumination. Below each plane, the corresponding normalized temporal standard deviation map (NTSDM, Supplementary Note 5) is shown to visualize temporal intensity fluctuations associated with moving stripes. Scale bar: 100 µm. **(b)** BDM treatment stripe removal results in the live zebrafish brain without resonant scanning. Each row presents the raw images of a randomly selected plane (out of 30) of a zebrafish brain and their corresponding normalized temporal standard deviation maps (NTSDM) before BDM treatment (left), after BDM treatment (middle), and following recovery in fresh water (right). Four planes from the same zebrafish brain are shown. Scale bar, 100 µm.

**Extended Data Fig. 4:**
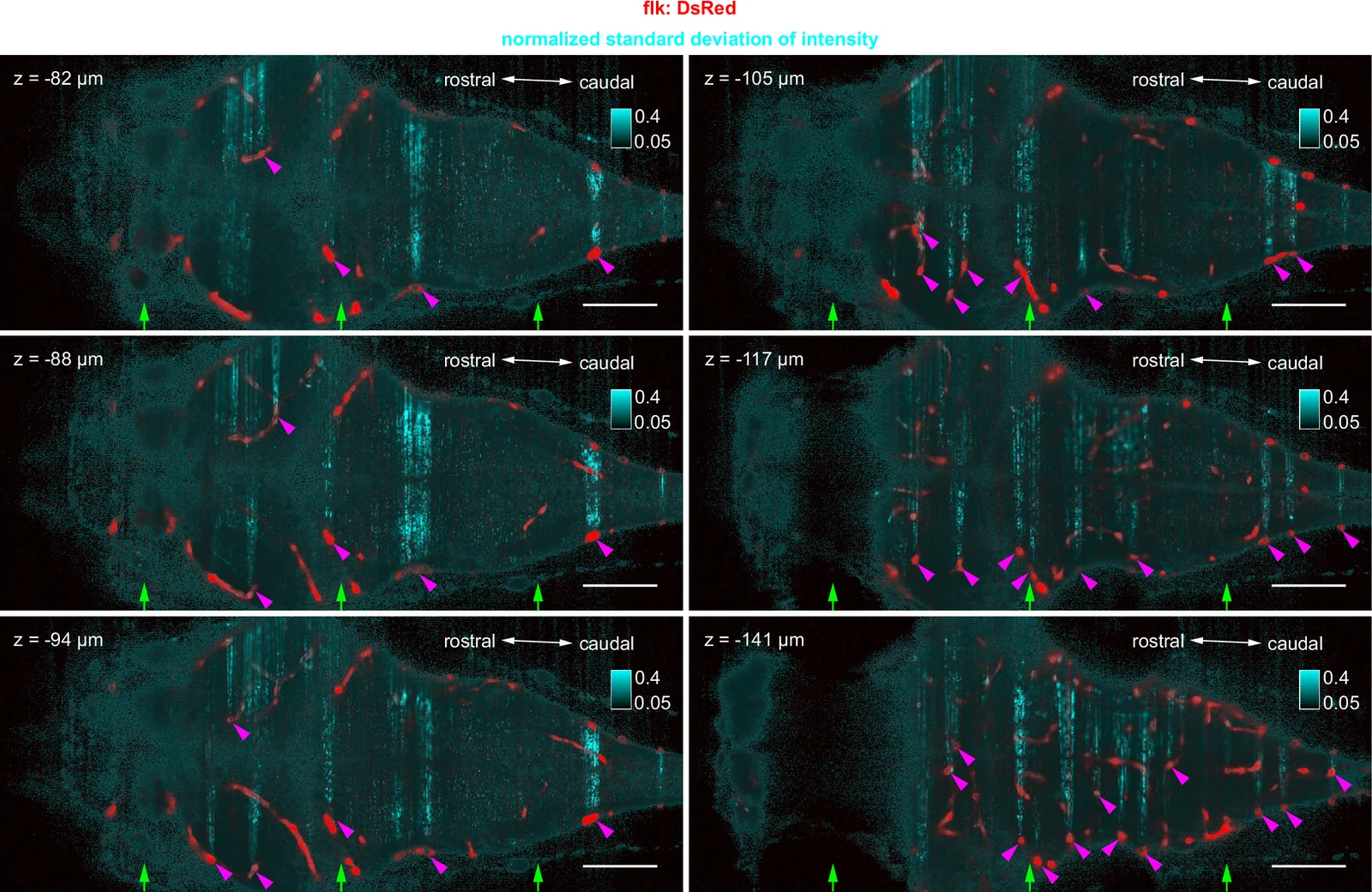
Spatial correspondence between blood vessels and moving stripes. Six randomly selected imaging planes (out of 30) from a zebrafish brain co-expressing the pan-neuronal Positron2-Kv voltage indicator and a vascular marker (flk:DsRed) are shown. In each plane, blood vessels (red) are overlaid with the map of moving stripes (cyan), represented by the normalized temporal standard deviation map (NTSDM, Supplementary Note 5) of fluorescence intensity in the GEVI channel. Magenta arrows highlight co-localization of moving stripes and blood vessels. Green arrows indicate the direction of light-sheet illumination. Scale bar: 100 µm.

To determine whether stripe artifacts arose from the optical system or from the biological sample, we performed a series of control experiments (Extended Data Figs. 2–4). Imaging a uniform fluorescent agarose sample under identical conditions revealed only a smooth (~±20% along an 800-µm *x* axis range; Supplementary Note 3), stationary intensity variation perpendicular to the illumination axis, attributable to nonuniformities in the Powell lens-generated light sheet (Extended Data Fig. 2a). This variation had low spatial frequency (<0.05 µm^−1^) and did not change over time (Extended Data Fig. 2c,d). In contrast, stripe patterns in zebrafish brains displayed sharp spatial features, varied across depths and exhibited higher spatial frequencies (0.05 µm^−1^ to 0.2 µm^−1^), indicating a distinct origin (Extended Data Fig. 2e,f). Shifting the fish orthogonally to the light sheet illumination axis during imaging showed that stripe patterns remained fixed relative to the brain rather than the microscope FOV, further supporting a sample-dependent origin (Extended Data Fig. 2b).

We next investigated the source of moving stripes. These artifacts were absent in paraformaldehyde-fixed fish and disappeared when cardiac contraction (and thus blood flow) was pharmacologically halted using 2,3-butanedione monoxime (BDM) (Supplementary Note 4), reappearing upon recovery (Extended Data Fig. 3, Supplementary Video 2 and Supplementary Note 5). Furthermore, in fish expressing fluorescent vascular markers, moving stripes spatially colocalized with blood vessels (Extended Data Fig. 4). Together, these results demonstrate that moving stripes arise from blood flow dynamically perturbing light-sheet propagation (Supplementary Note 6).

**Extended Data Fig. 5:**
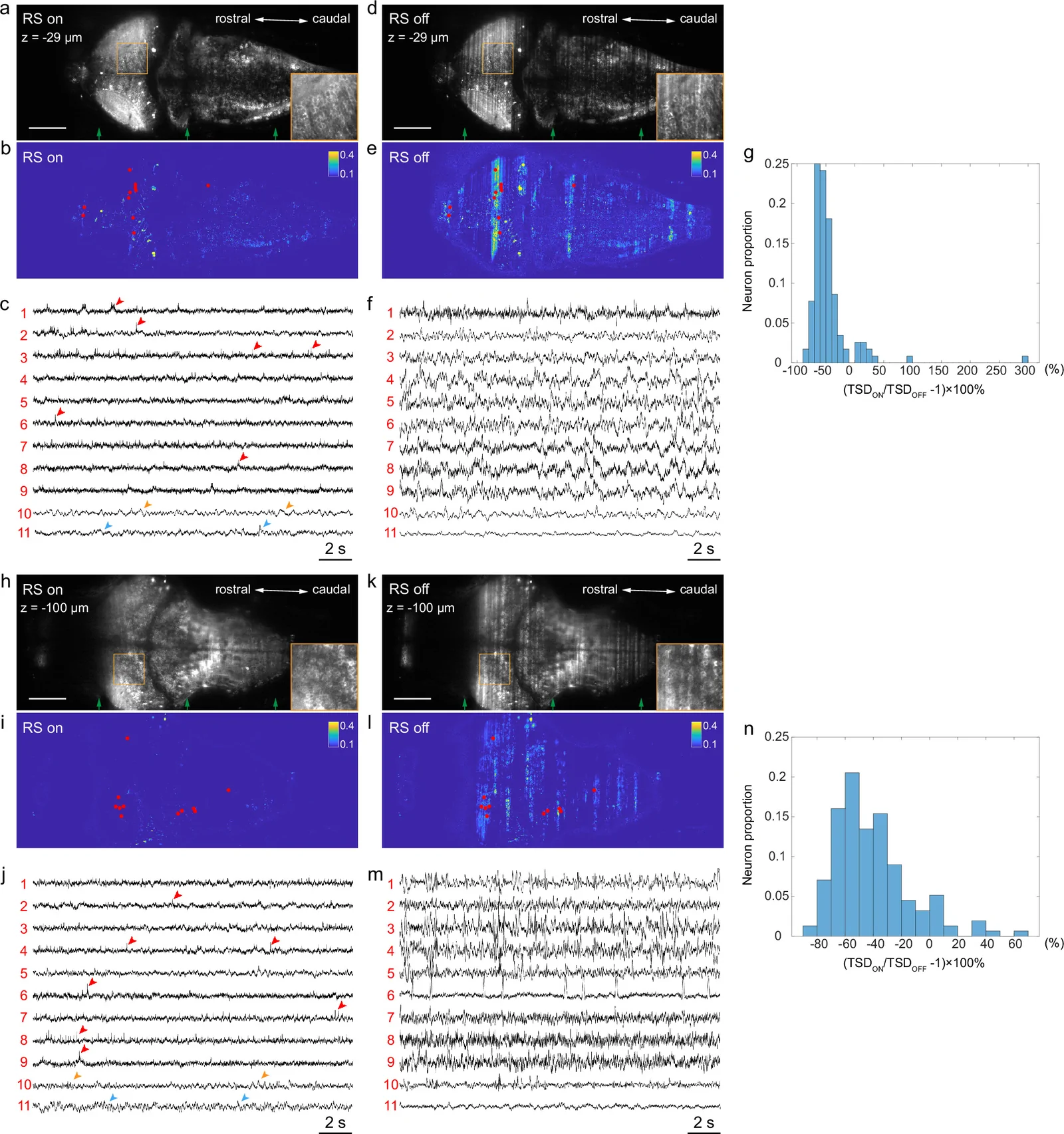
Suppression of stripe artifacts using a resonant mirror scanner. We imaged 20 planes of a zebrafish brain at 203 Hz for 50 s. The resonant scanner was enabled during the first 30 s and disabled during the remaining 20 s. **(a)** Raw image of a randomly selected plane (out of 20) of a zebrafish brain expressing pan-neuronal Positron2-Kv indicator, acquired with resonant scanner enabled (RS on). Green arrows indicate the direction of light-sheet illumination. An enlarged view of the orange-boxed region is shown at bottom right. Scale bar: 100 µm. **(b)** The normalized temporal standard deviation map (NTSDM) corresponding to the plane shown in (a). Red dots indicate representative neurons whose traces are shown in (c,f) for comparison between resonant scanning on and off conditions. **(c)** Intensity traces of the neurons labeled in (b). The majority of ROI traces (~75%) were unaffected by moving stripes and are not shown. Representative traces from three classes are shown. Traces 1–9 show examples of moving-stripe-affected ROIs in which neuronal spikes (red arrows) became detectable when resonant scanning was enabled. Trace 10 illustrates a subset (<10% of moving-stripe-affected ROIs) for which resonant scanning did not effectively suppress temporal artifacts (orange arrows). Trace 11 shows a small subset of neurons (~1% of all segmented ROIs) that did not exhibit moving-stripe-induced artifacts when resonant scanning was off but displayed temporal artifacts when resonant scanning was enabled (blue arrows). Traces 1–10 were intentionally selected from moving-stripe-affected ROIs to illustrate the efficacy of resonant scanning in suppressing moving-stripe artifacts. **(d-f)** Corresponding results from the same imaging plane as in (a–c), acquired without resonant scanning (RS off). **(g)** Histogram of differences in temporal standard deviation (TSD) between resonant scanning on (TSD_ON_) and off (TSD_OFF_) conditions. Only the neurons within the plane in (a–f) and affected by moving-stripe artifacts when the scanner was off were included in this analysis. **(h-n)** Same analyses as in (a–g) for a second randomly selected imaging plane from the same zebrafish brain. Source data

Because only moving stripes introduce deleterious temporal artifacts, and stationary stripes do not affect data quality more than other static causes of variability (for example, inevitable variability of protein expression across cells), we focused on mitigating moving-stripe effects. We explored three complementary strategies: transient cardiac arrest, optical averaging via resonant light-sheet pivoting (Extended Data Fig. 5), and computational exclusion of contaminated ROIs. Each approach entails tradeoffs between experimental invasiveness, optical complexity, spatiotemporal coverage, and data yield (Supplementary Notes 4 and 7). While cardiac arrest and resonant scanning can suppress moving stripes at the acquisition stage, both impose practical constraints (Supplementary Note 4). For the majority of datasets in this study, we therefore adopted a computational approach to identify and exclude ROIs affected by moving stripes based on their characteristic synchronous, pulse-like temporal signatures and stripe-like spatial organization. This strategy preserved 59–92% of segmented ROIs across the brain while yielding artifact-free voltage traces for downstream analysis (Supplementary Table 1).

### Performance of the microscope

To demonstrate the utility of our microscope, we sought to choose a voltage indicator with sufficient performance to explore the kinds of insights that could be derived. There is, admittedly, no perfect voltage indicator at the current moment, and each voltage indicator incurs specific tradeoffs and sacrifices. For example, one voltage indicator may be very bright or exhibit high SNR, but the kinetics may be challenging to capture. Another may have more ideal kinetics but be too dim. Acknowledging that voltage indicators are improving rapidly, and thus, the best voltage indicator by the time this paper is published might be much better than the ones available today, we focused our attention on the question of whether whole-brain voltage imaging was crossing the threshold of being feasible, rather than focusing on any one voltage indicator.

Using our microscope, we imaged voltage activity of neurons distributed across much of the entire brains of 5–6 dpf larval zebrafish with pan-neuronal expression of Positron2-Kv (Figs. 1d and 2). This indicator was selected after an initial comparison of five GEVIs, including ASAP3-Kv, Voltron2-Kv, Ace-mNeon2-Kv, pAce-Kv and Positron2-Kv, transiently expressed via embryonic microinjection. Among the tested indicators, Voltron2-Kv and Positron2-Kv exhibited the highest signal-to-noise ratio per action potential (SNR_AP_) (defined as the ratio of the fluorescence change per spike to the shot noise at the spike peak: ${{\rm{SNR}}}_{{\rm{AP}}}=({N}_{\mathrm{peak}}-{N}_{0})/\sqrt{{N}_{\mathrm{peak}}}$, where ${N}_{\mathrm{peak}}$ and ${N}_{0}$ are the detected photon numbers per neuron per frame at the spike peak and at the resting potential immediately preceding the spike, respectively; both were estimated by eye), presumably due to their high brightness (Supplementary Note 8).

We then generated stable transgenic zebrafish lines expressing Voltron2-Kv and Positron2-Kv pan-neuronally using Tol2 transgenesis and the Gal4/UAS system, with Gal4 driven by the HuC pan-neuronal promoter. We established one line per indicator. In the Voltron2-Kv line we generated, we did not observe robust voltage activity, potentially due to factors unrelated to the indicator itself. Because our goal was to validate the microscope’s capability for brain-scale voltage imaging rather than to optimize indicator expression, we proceeded with the Positron2-Kv line in our experiments, as a somewhat arbitrary choice.

In our Positron2-Kv fish, fluorescence was well localized to neuronal membranes throughout the brain (Fig. 2a,b). Individual neurons are visually resolvable, showing cellular-resolution voltage imaging at multiple depths (Fig. 2b). Ventral brain regions were densely labeled (Fig. 2a, *z* = −108 µm and −136 µm), whereas dorsal regions occasionally contained unlabeled regions (Fig. 2a, *z* = −23 µm, −51 µm and −79 µm). These unlabeled regions could result from several mechanisms, such as suboptimal genomic insertion sites that suppress expression in specific neuronal populations, incomplete dye labeling of Positron2-Kv, whereby indicator proteins synthesized during the ~4-h interval between staining and imaging remain unbound to dye and thus nonfluorescent, and transcriptional silencing associated with tandem UAS repeats^34^. To assess promoter effects, we compared Gal4/UAS-driven expression with direct expression under the HuC promoter. Direct HuC expression yielded more uniform pan-neuronal coverage but reduced fluorescence brightness (Supplementary Fig. 6). Compared with *Tg(HuC:Positron2-Kv)*, the Gal4/UAS line was brighter on average (~1.1× across the whole brain, ~1.7× in optic tectum and ~2.6× in habenula) but showed greater regional heterogeneity. Although this was not a systematic comparison, it suggests that improved transgenic strategies could increase recorded neuron yield, even at the expense of expression brightness, given the enhanced light efficiency of our microscope (75% improvement using dual remote mirrors; Supplementary Figs. 1 and 2).

For imaging, intact and awake larval zebrafish were mounted in 3% low-melting agarose on a 3D-printed holder in a customized water chamber, with the head exposed and the tail free (Fig. 1d). During initial experiments, we found tail motion and muscle contractions produced multidirectional, nonrigid motion artifacts that severely corrupted voltage traces. Because these artifacts were difficult to correct reliably, we paralyzed fish using a muscle relaxant, reducing motion to subpixel levels.

We imaged 30 *z*-planes spanning a volume of 930 × 370 × 170 µm^3^ with a volume acquisition time of 4.98 ms, corresponding to a volumetric rate of 200.8 Hz and an effective aggregate frame rate of 6,025 frames per second. Because the ventral larval zebrafish brain is dominated by neurites, extending the axial (*z*) imaging range beyond 170 µm yields only ~5.4% additional neuron somas^35^. With 30 planes, the axial step size was 5.86 µm, smaller than the average neuronal soma diameter (6.62 µm, mean^1^) and slightly larger (by 17%) than the value used to achieve single-cell resolution in previous studies^1,4^. Our experiments were performed with ~10 mW average excitation power (~43 mW temporal power within each excitation pulse), yielding 2.1 ± 0.8 × 10^4^ detected photons per neuron per frame (mean ± s.d., *n* = 15 neurons in one fish, single remote mirror).

To quantify baseline noise beyond shot noise, we imaged pan-neuronally labeled *Tg(HuC:Gal4; UAS:Kaede)* fish at 200.8 Hz under imaging conditions matched to the baseline fluorescence intensity in the voltage imaging experiments (Supplementary Fig. 7 and Supplementary Note 9). After correcting for photobleaching, the measured noise was 1.58 ± 0.45 times (mean ± s.d., *n* = 72 neurons from one fish) the theoretical shot noise and 0.83 ± 0.28% (mean ± s.d., *n* = 72 neurons from one fish) of baseline intensity.

Because each *z*-plane was revisited every 4.98 ms, an ideal indicator would have activation and deactivation kinetics on the order of several milliseconds to minimize the chance of missing narrow spikes. For this study, we prioritized SNR_AP_ over kinetics. Positron2-Kv, which shares the pAce rhodopsin domain, is expected to exhibit similar submillisecond activation and deactivation time constants (0.51 ms and 0.61 ms, respectively) at room temperature^27^. Consequently, some spikes may be missed at a 200 Hz sampling rate. We therefore confined our conclusions to observations robust to such losses and explicitly quantified spike loss.

To estimate the fraction of missed spikes, we recorded a single *z*-plane at 1,000 Hz with a 40-µs exposure time and downsampled the data to 200 Hz by extracting evenly spaced frames. After excluding stripe-affected ROIs, we analyzed 201 neurons that exhibited spiking activity during an 18 s recording and had SNR_VolPy_ > 4. SNR_VolPy_ is defined as the ratio between the average detected spike amplitude and the s.d. of the negative portions of the trace after 15-Hz high-pass filtering^36^. On average, 30.0% of spikes observed at 1,000 Hz were missed in the 200 Hz traces (Supplementary Fig. 8), consistent with the indicator’s submillisecond kinetics. We then evaluated whether slower indicators could mitigate this effect. Simulations using ASAP5-Kv^37^ (Supplementary Note 10), which has ~2.6 ms depolarization and ~4.3 ms repolarization time constants, showed that 99.7% of spikes detected at 1 kHz were recovered at 200 Hz (Supplementary Fig. 9). Experimentally, sparse transient expression of ASAP5-Kv confirmed that spikes observed at 1,000 Hz were preserved after downsampling to 200 Hz (Supplementary Fig. 9d,e). These results indicate that a future indicator combining ASAP5-like kinetics with Positron2-like brightness could substantially reduce spike loss while maintaining high SNR_AP_.

**Extended Data Fig. 6:**
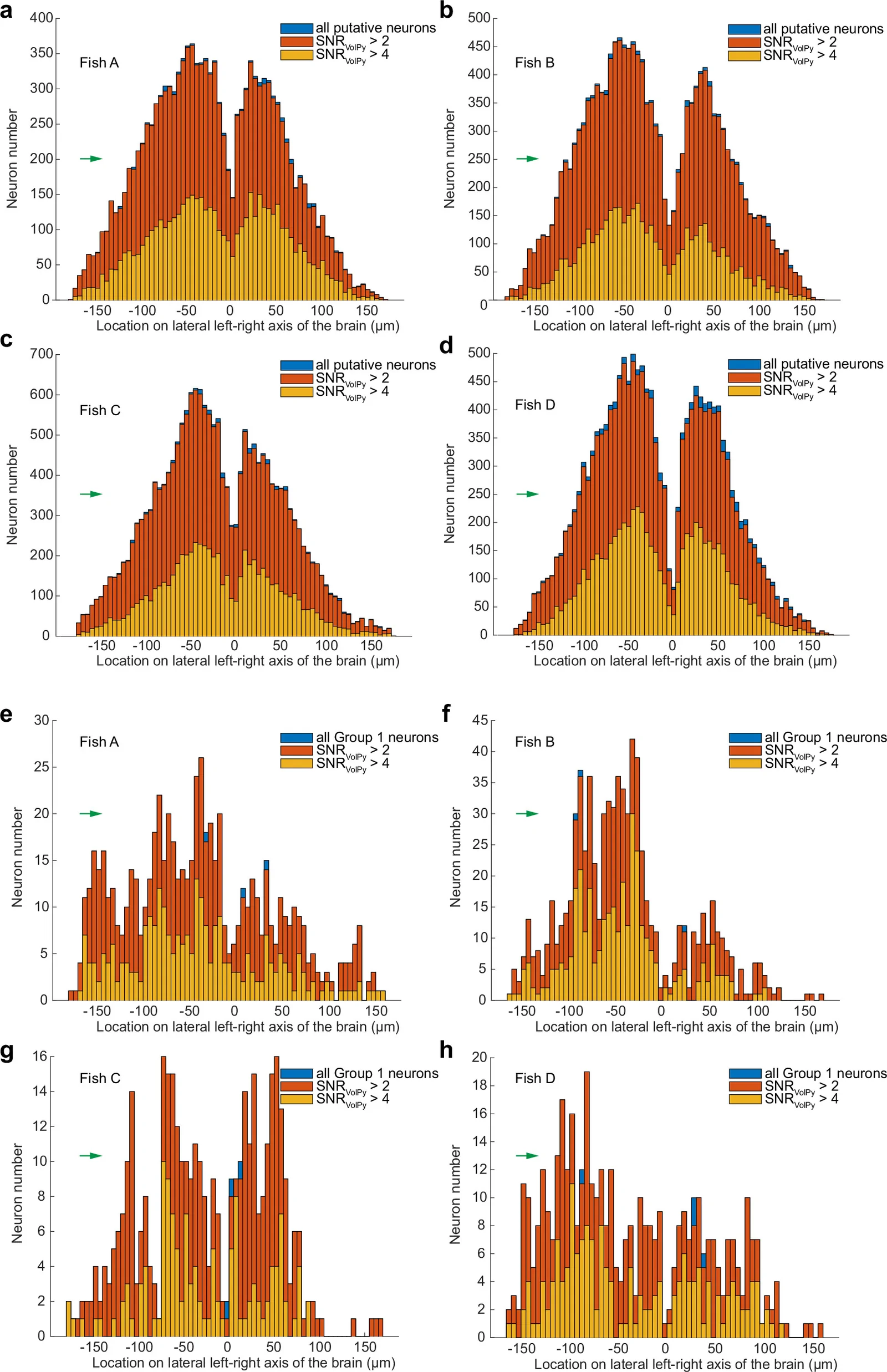
Histograms show the distribution of all putative neurons and Group 1 neurons along the lateral left-right axis of the brains of Fish A-D. **(a-d)** For each fish, three histograms are shown: one for all putative neurons, and two for putative neurons that passed two SNR_VolPy_ thresholds (SNR_VolPy_>2, SNR_VolPy_ > 4). Green arrows indicate the direction of light-sheet illumination. The zero position on the horizontal axis corresponds to the brain midline, separating the left and right hemispheres and characterized by a reduced density of GEVI-labeled neurons. ROIs affected by moving-stripe artifacts were computationally identified and excluded prior to all analyses shown. **(e-h)** Corresponding histograms for Group 1 neurons (UV-onset–responding neurons) from Fish A–D.

Illumination geometry imposed additional constraints. The fish’s eyes blocked illumination of the ventral telencephalon (Fig. 2a,e), and some regions were blurred due to refraction or scattering (Fig. 2a). By manually delineating these regions across all *z*-planes (Supplementary Fig. 10), we found that single neurons were visually resolvable in 84.7% of the imaged brain area; 8.6% was shadowed by the eyes and 6.7% was too blurred. Blurring was more pronounced on the side opposite the incident light sheet, resulting in an asymmetric distribution of detected neurons and higher signal quality on the illuminated side, where the light sheet traversed thinner tissue (Extended Data Fig. 6a–d). This asymmetry could be mitigated by dual-sided light-sheet illumination.

To minimize unintended visual stimulation and potential photodamage, we used an opaque optical mask in the light-sheet arm to block illumination of the fish’s eyes. Of note, introduction of the mask did not measurably increase the brain volume shadowed by the eyes.

For ROI segmentation, we trained a custom Cellpose v.3.0 model^28^ using manually segmented membrane-labeled neurons and iterative bootstrapping. On model-segmented datasets (Supplementary Fig. 11), manual review added 80.2 ± 27.5 ROIs (mean ± s.d., *n* = 30 layers from one fish) per layer and removed 10.9 ± 9.4 ROIs (mean ± s.d., *n* = 30 layers from one fish) per layer. All analyzed datasets first used this automated segmentation followed by manual correction. Neural activity traces were extracted with VolPy^36^ and ROIs affected by stripe artifacts were excluded computationally.

We show raw images (Fig. 2a,b) and spontaneous activity traces (Fig. 2c,d) from a 5.5-dpf larval zebrafish. We show activity traces from 120 exemplar putative neurons (Fig. 2c,d) across six brain regions (Fig. 2e), including the olfactory epithelium (OE), telencephalon (TL), habenula (HB), optic tectum (OT), cerebellum (CB) and medulla oblongata (MO), demonstrating our microscope’s ability to monitor voltage dynamics across the brain. These traces show diverse voltage dynamics, including isolated spikes, burst firing, and oscillatory activity, with SNR_AP_ values ranging from 5 to 10.

**Extended Data Fig. 7:**
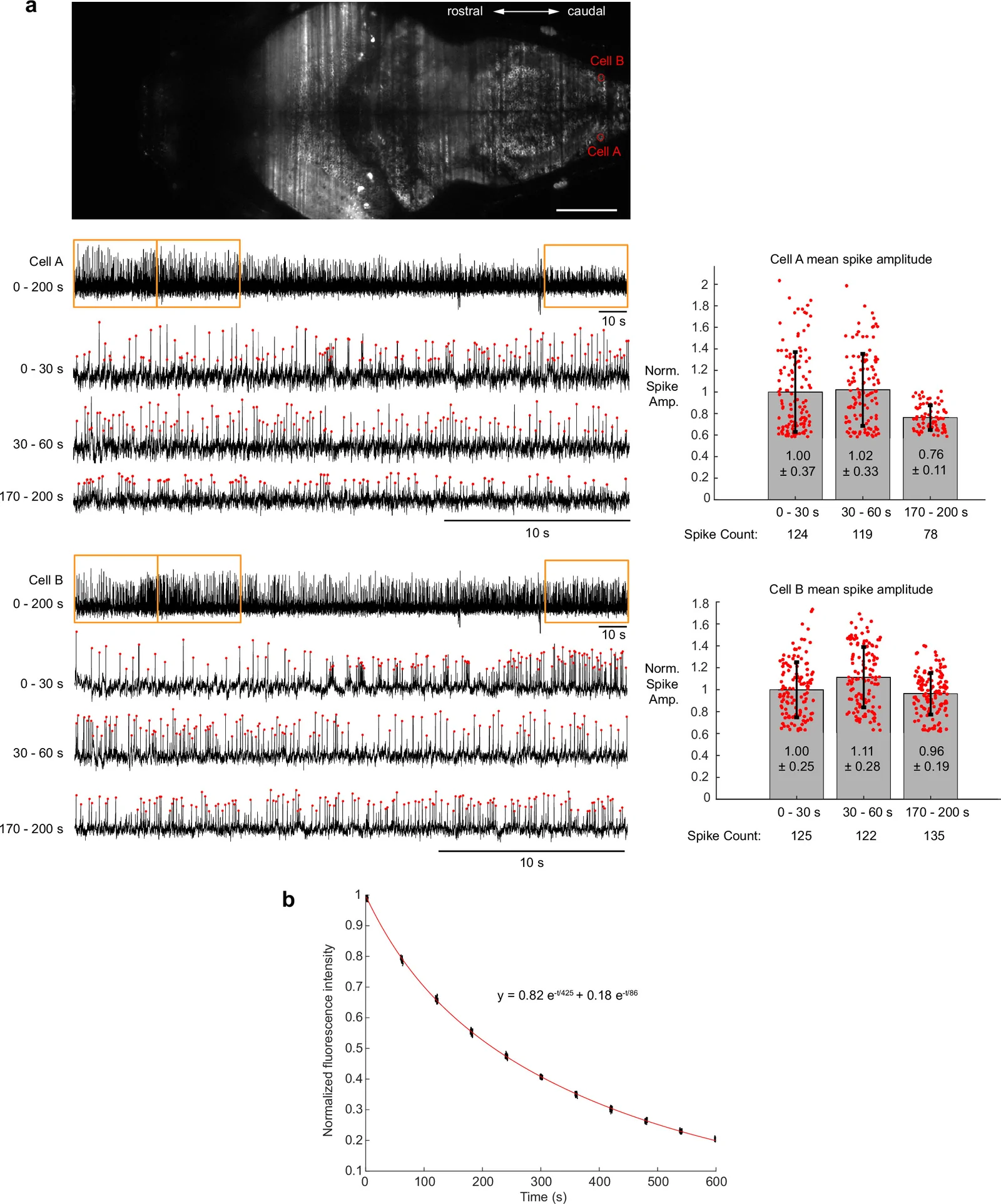
Voltage activity and photobleaching during prolonged imaging. **(a)** Voltage activity during a 200-s long imaging trial. The top panel displays a raw image from a randomly selected brain plane (out of a total of 30), with two cells (Cell A and Cell B) marked for neural trace analysis. These cells were arbitrarily selected from areas without visible moving stripes based on visual inspection of the raw videos. Below, full 200-s neural traces of Cell A and Cell B are shown. The first 60 s and the final 30 s of the traces (highlighted by orange boxes in the upper trace) are displayed at expanded time scales. Red dots indicate spikes detected by VolPy. Bar plots at right summarize spike counts and normalized mean spike amplitudes (mean ± standard deviations) for each time section (0-30 s, 30-60 s, and 170-200 s). Scale bar in the top panel: 100 µm. **(b)** Photobleaching during continuous whole-brain light-sheet scanning. The normalized photobleaching curve was measured from a zebrafish brain different from that shown in (a), expressing Positron2-Kv and stained with JF525. The brain was continuously illuminated by a 515-nm light sheet scanned at 200.8 Hz, with a total average power of 10.4 mW. To reduce data storage requirements, volumetric imaging was performed for 4.5 s at 1-min intervals. The measured fluorescent intensities averaged across the imaged brain volume at each time point are shown as black dots, with a double-exponential fit overlaid in red. The fitted function is shown. Source data

**Extended Data Fig. 8:**
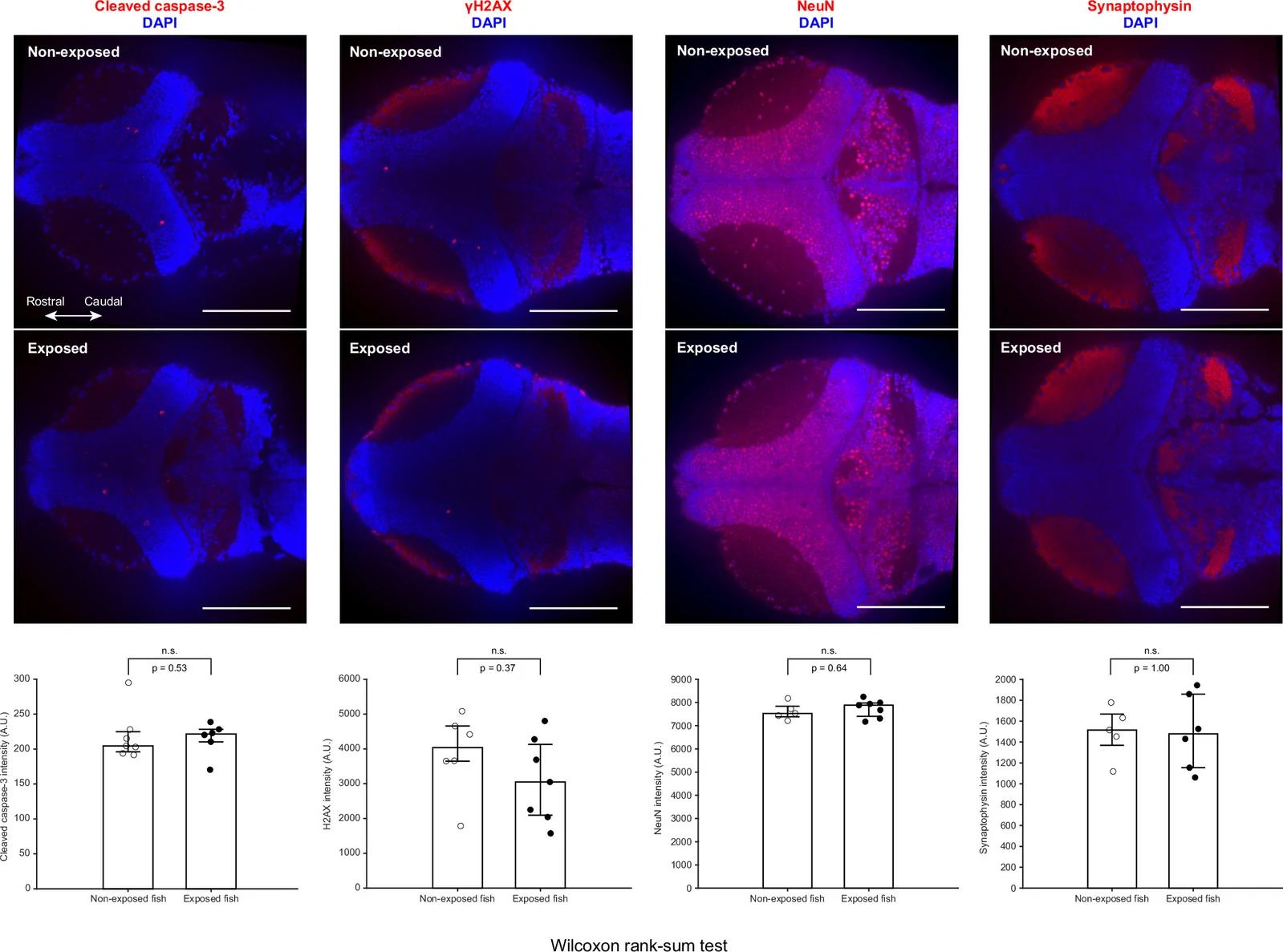
Immunostaining of cellular and synaptic health markers following light-sheet exposure under voltage-imaging conditions. Each column corresponds to a distinct cellular or synaptic health marker: the apoptotic marker cleaved caspase-3, the DNA damage marker γH2AX, the neuronal nucleus marker NeuN, and the synaptic marker synaptophysin, as used in previous neurotechnology safety studies (Linghu et al. 2020; Grossman et al. 2017). At the top of each column, representative single-plane immunostaining images display the markers (red) and nuclear DAPI staining (blue) in exposed and non-exposed fish brains. At the bottom, bar plots show the marker intensities between exposed and non-exposed fish. Sample sizes for the non-exposed and exposed groups in each column, from left to right, are n = 7,6;6,7;5,7;5,6 fish, respectively. No significant differences were detected for any marker (Wilcoxon rank-sum test; n.s.), with *p* values shown. Images were acquired using a confocal microscope, which does not generate stripe artifacts because it excites samples using high-NA light cones instead of parallel light sheet. Scale bar: 100 µm. Source data

Although most of our individual experimental trials were configured to last ~35 s, the imaging duration achievable with our system is not intrinsically limited by acquisition hardware, but instead by photobleaching and data storage capacity. To demonstrate the feasibility of extended recordings, we continuously imaged the brain of a larval zebrafish for 200 s at a volume rate of 200.8 Hz (Extended Data Fig. 7a). Voltage activity remained detectable throughout the recording. Traces from two arbitrarily selected neuron ROIs (cell A and cell B), chosen from regions without visible moving-stripe artifacts, exhibited clear spike events even at the end of the acquisition. Relative to the beginning of the recording, spike amplitudes were reduced by 24% for cell A and 4% for cell B, consistent with gradual photobleaching. To quantify photobleaching systematically, we measured the fluorescence decay of Positron2-Kv stained with JF525 over a 10-min imaging period (Extended Data Fig. 7b). Fluorescence intensity decreased by 50% after approximately 210 s of imaging. Notably, the reduction in spike amplitude observed in Extended Data Fig. 7a was slower than the ensemble photobleaching curve in Extended Data Fig. 7b, which showed a 42% decrease over the same duration. Several factors likely contribute to this discrepancy. First, the denoising and filtering steps implemented in VolPy can enhance apparent spike amplitudes, partially compensating for reduced fluorescence. Second, the curve shown in Extended Data Fig. 7b reflects population-averaged photobleaching behavior, whereas the results shown in Extended Data Fig. 7a illustrate individual neuronal examples that may deviate from the mean. Because our experiments used substantially higher excitation power (~10 mW, ~33.3×) than that used in whole-brain calcium imaging^1^, we assessed phototoxicity as in previous neurotechnology safety assays^38^. Following light-sheet exposure, we immunostained for cleaved caspase-3, γH2AX, NeuN and synaptophysin. We observed no significant differences between exposed and control fish for any marker (Extended Data Fig. 8; cleaved caspase-3, *P* = 0.53; γH2AX, *P* = 0.37; NeuN, *P* = 0.64; synaptophysin, *P* = 1.00; Wilcoxon rank-sum test). Together, these data demonstrate feasibility of extended, continuous voltage imaging of neurons distributed across most of the zebrafish brain and provide a path toward whole-brain voltage imaging.

### Imaging voltage activity of neurons distributed across most of the brain

Zebrafish larvae possess ultraviolet (UV)-sensitive photoreceptors, and UV vision is thought to support prey detection while also driving avoidance behaviors^39,40^. Previous whole-brain calcium imaging studies demonstrated that high-intensity 405-nm illumination (~0.6 mW mm^−2^) can elicit brain-wide neural activity within approximately one second^7,9^. Here, we adapted this established light-stimulation paradigm to image activity of neurons distributed across much of the entire brain in 5–6 dpf larval zebrafish.

**Fig. 3:**
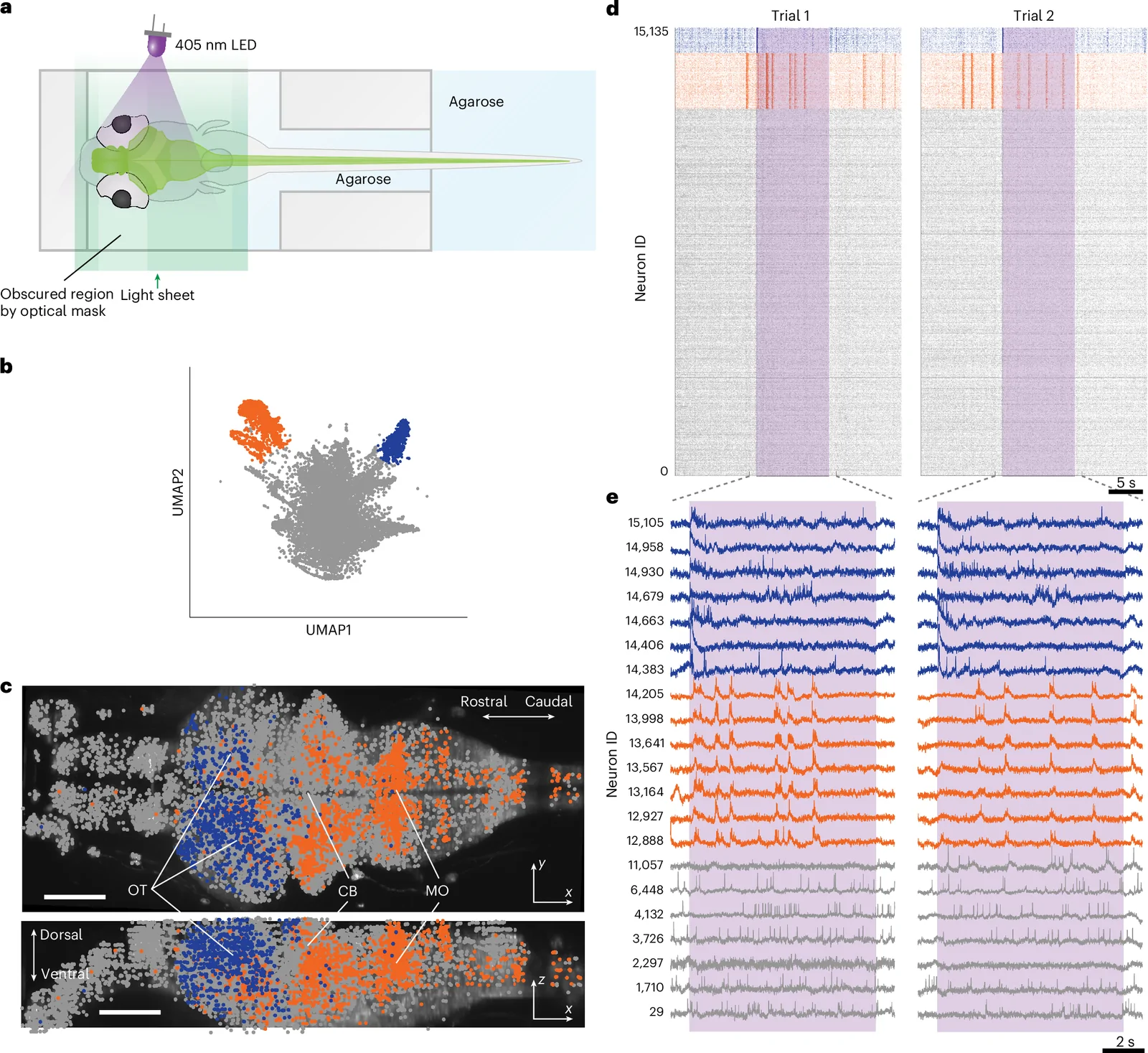
Imaging the activity of neurons distributed throughout much of the zebrafish brain during visual stimulation. **a**, Visual stimulation paradigm. Lateral light stimulation (illustrated from the right side; left-side stimulation is also possible) is delivered for 10 s during each trial. **b**, UMAP embedding of spiking activity from all putative neurons in fish B, projecting neuronal firing patterns into a two-dimensional space. Neurons with similar activity profiles cluster together and are color-coded by group. ROIs affected by moving-stripe artifacts were computationally identified and excluded from this and all subsequent analyses. **c**, Spatial distribution of neuron groups in fish B. Group 1 neurons (blue) are predominantly localized in the OT; group 2 neurons (orange) are primarily found in the hindbrain; group 3 neurons (gray) are distributed broadly across the brain. Scale bars, 100 µm. **d**, Spike raster plots for all putative neurons in Fish B across visual stimulation trials. Group 1 neurons (blue) show increased firing following stimulus onset; group 2 neurons (orange) show multiple episodes of elevated activity during the trials; group 3 (gray) comprise the remaining putative neurons. **e**, Representative traces of subthreshold membrane potential and spiking activity from putative neurons shown in **d**. Source data

We imaged the larval zebrafish brain at a volume rate of 200.8 Hz for 35 s per trial, with UV illumination applied for 10 s (*t* = 13–23 s; Fig. 3a). Each fish underwent two identical trials separated by a 20-min dark interval. Eight fish were imaged in total. One fish was excluded due to a slow heart rate indicative of poor health, and three additional fish were excluded due to large intertrial positional drift (>5 pixels laterally, together with axial drift that moved cells out of the plane), likely caused by movement between trials that prevented reliable cell registration. The remaining four fish (fish A–D) exhibited minimal drift (<1 pixel or <0.73 µm within trials; <2 pixels or <1.46 µm between trials along both *x* and *y* axes; Supplementary Fig. 12). Residual motion was corrected using NoRMCorre^41^. UV stimulation was delivered from the right side in fish A and B, and from the left side in fish C and D (Fig. 3a).

We segmented putative neuronal ROIs in each fish using a custom-trained Cellpose-based deep learning model followed by manual correction. This yielded 17,700, 25,552, 25,464 and 17,419 ROIs in fish A–D, respectively, before computational removal of moving-stripe-affected ROIs. Fluorescence time series were extracted using VolPy, which performs background subtraction, denoising and spike detection via adaptive thresholding^36^. Due to the presence of moving-stripe artifacts in the whole-brain datasets, a subset of ROIs exhibited correlated, pulse-like intensity fluctuations along the medio-lateral axis. These ROIs were computationally identified and excluded. After removal of artifact-contaminated ROIs, 12,935, 15,135, 19,039 and 15,998 ROIs remained in fish A–D, respectively (yields, 73%, 59%, 75% and 92%), and were treated as putative neurons for subsequent analyses.

To assess the spatial distribution of putative neurons following artifact removal, we analyzed neuron density in fish A (chosen arbitrarily). For each putative neuron, we computed the average distance to its ten nearest neighbor centroids within the same *z*-plane. The resulting distribution (Supplementary Fig. 13) showed a mean separation of 17.8 ± 15.2 µm (mean ± s.d.; *n* = 12,935 neurons from one fish), approximately 31% larger than a literature-based estimate of 13.6 µm. This increased spacing likely reflects a combination of factors, including regional variation in indicator expression (Supplementary Fig. 6), removal of artifact-corrupted ROIs, and reduced image quality in certain brain regions due to tissue scattering (Supplementary Fig. 10).

We next examined whether one-sided UV illumination or light-sheet excitation introduced spatial biases in data quality. For each fish, we analyzed the left–right (*y* axis) distribution of putative neurons using three subsets: all neurons, neurons with SNR_VolPy_ > 2, and neurons with SNR_VolPy_ > 4 (Extended Data Fig. 6a–d). Although the SNR_VolPy_ thresholds were chosen arbitrarily, they provide a practical sense of signal quality. Across all fish, 98.4% of putative neurons exceeded SNR_VolPy_ > 2, likely reflecting the effectiveness of VolPy’s denoising and footprint optimization procedures.

To identify patterns of neural activity, we embedded the spike trains of all putative neurons into a two-dimensional manifold using Uniform Manifold Approximation and Projection (UMAP) (Fig. 3b and Supplementary Fig. 14) and applied density-based spatial clustering of applications with noise (DBSCAN). This analysis revealed three broad groups of neurons. Group 1 neurons (Fig. 3b) showed increased spiking activity following the onset of UV illumination in both trials. This response was triggered by light onset but not offset, consistent with previous calcium imaging studies^42^. In the raw traces of all recorded neurons, we observed a reversible photoswitching effect during UV exposure, which could be effectively removed by VolPy and therefore did not affect this analysis (Supplementary Note 11 and Supplementary Fig. 15). Group 2 neurons (Fig. 3b) exhibited recurring burst activity throughout the recording that did not correlate with the UV stimulus. The remaining neurons, which lacked distinct temporal structure, were classified as group 3 (Fig. 3b). These groupings are not intended to define cell types but rather to summarize activity patterns revealed by voltage imaging.

The three groups exhibited distinct spatial distributions. Averaged across four fish, approximately 92% of group 1 neurons were located in the OT, whereas ~90% of group 2 neurons resided in the hindbrain (Fig. 3c). Group 3 neurons were broadly distributed throughout the brain.

We next focused on the temporal structure of group 1 neurons. Across fish, a majority of these neurons were located in the left OT (70%, 79%, 57% and 67% in fish A–D, respectively; Fig. 3c and Extended Data Fig. 6e–h). In all experiments, the excitation light sheet entered from the left. UV stimulation was delivered from the right in fish A and B and from the left in fish C and D. The observed leftward bias, therefore, most likely reflects higher signal quality on the illuminated side, where the light sheet traversed thinner tissue and yielded improved signal quality (Extended Data Fig. 6). This asymmetry could be mitigated in future experiments using dual-sided illumination.

**Fig. 4:**
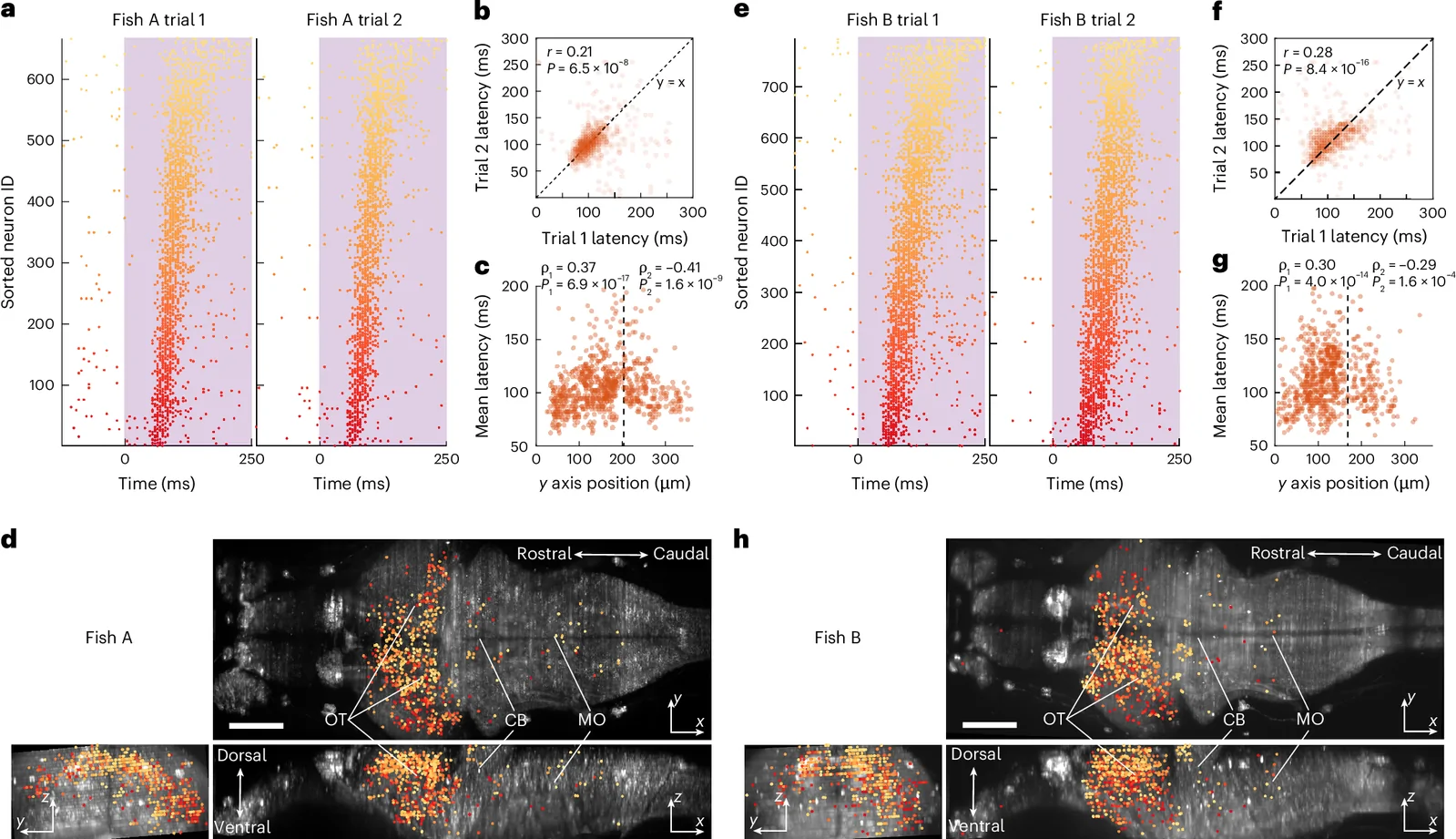
Spatial mapping of stimulus-evoked firing sequences in the zebrafish brain. **a**, Consistency of firing sequence order for group 1 neurons in fish A across trials. Neuron IDs are sorted by peak latency averaged across trials 1 and 2. ROIs affected by moving-stripe artifacts were computationally identified and excluded from all analyses in this figure. **b**, Trial-to-trial reliability of firing latency in fish A. Peak spiking rate latencies relative to UV stimulus onset in trial 1 are positively correlated with those in trial 2 (Pearson’s correlation, two-sided test; *r* and *P* values shown). **c**, Relationship between firing latency and neuronal spatial position along the lateral–medial (*y*) axis in fish A. The dashed line denotes the brain midline separating left and right hemispheres. Spearman’s rank correlations (two-sided) were computed independently for each hemisphere, with *ρ* and *P* values indicated. **d**, Spatial distribution of group 1 neurons in fish A, color-coded by firing latency. Scale bar, 100 µm. **e**–**h**, Same analyses as in **a**–**d** for Fish B.

**Extended Data Fig. 9:**
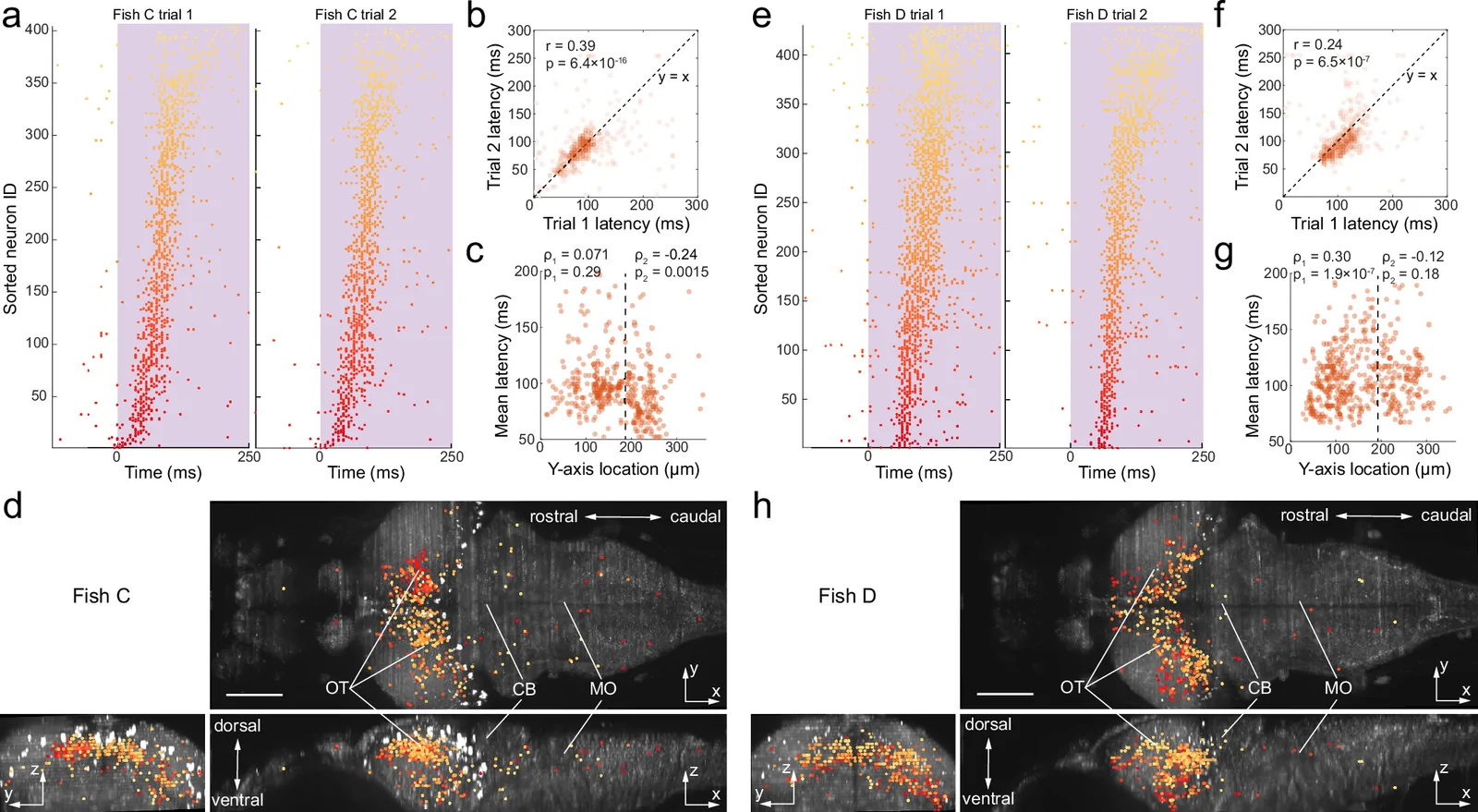
Spatial mapping of stimulus-evoked firing sequences in the zebrafish brain (Fish C and Fish D). **(a)** Consistency of firing sequence order for Group 1 neurons in Fish C across trials. The sequential ordering of neurons by peak spiking rate latency in trial 1 closely matches that in trial 2. Neuron IDs are sorted by peak latency averaged across trials 1 and 2. Regions of interest (ROIs) affected by moving-stripe artifacts were computationally identified and excluded from all analyses in this figure. **(b)** Trial-to-trial reliability of firing latency in Fish C. Peak spiking rate latencies relative to UV stimulus onset in trial 1 are positively correlated with those in trial 2 (Pearson’s correlation, two-sided test; r and p values shown). **(c)** Relationship between firing latency and neuronal spatial position along the lateral–medial (y) axis in Fish C. The dashed line denotes the brain midline separating the left and right hemispheres. Spearman’s rank correlations (two-sided) were computed independently for each hemisphere, with ρ and p values indicated. **(d)** Spatial distribution of Group 1 neurons in Fish C, color-coded by firing latency. The majority of Group 1 neurons are located on the left lateral side of the optic tectum. OT, optic tectum; CB, cerebellum; MO, medulla oblongata. Scale bar, 100 µm. **(e-h)** Same analyses as in (a–d) for Fish D.

To characterize stimulus-evoked temporal dynamics, we smoothed each group 1 neuron’s spike raster with a 100-ms Hanning window to estimate firing rates. For each neuron, we identified the peak firing rate and its latency relative to UV onset. Neurons were ordered by their mean latency across the two trials (Fig. 4a,e and Extended Data Fig. 9a,e). This ordering was highly consistent between trials, as evidenced by correlations in peak latency (Fig. 4b,f and Extended Data Fig. 9b,f), revealing a reproducible temporal sequence spanning approximately 200 ms following UV onset.

Notably, this temporal sequence mapped onto spatial structure within the OT. The mean latency to peak firing correlated with neurons’ positions along the medial–lateral axis (Fig. 4c,g). Neurons that fired earlier were located more laterally, whereas later-firing neurons occupied progressively more medial positions (Fig. 4d,h). This spatiotemporal pattern was largely consistent across all four fish and on both sides of the tectum. One exception occurred in fish C on the right side, where neurons in the medial region fired earlier in the sequence. This deviation may reflect lower SNR on the right OT or uncontrolled behavioral or physiological factors.

These results illustrate how voltage imaging of neurons distributed across most of the brain can generate novel hypotheses, in this case, a mapping of time onto space. How do these findings relate to known tectal anatomy and physiology? An anatomical map by Isa et al.^43^ describes the multilayered organization of the OT and visual processing pathways for various stimuli. Although UV responses were not isolated in that work, recent calcium imaging studies of multicolor visual circuits^39,42^ show optic-tectum-wide activation for both visible and UV stimuli, suggesting UV-processing circuits might follow the (better described) organizational principles of visible light ones. One such principle is the layered architecture of the tectum^44^: retinal ganglion cell axons synapse onto neurons in the most lateral layers, which then connect to progressively more medial layers and to extratectal regions. Our observation of sequential activation from lateral-to-medial locations is therefore consistent with known circuitry, although our experiment was not designed to do a full scientific study, but rather to show the kind of hypotheses that one could generate with our technique. Such hypotheses could be tested in future studies through registration to zebrafish brain atlases^45,46^ or targeted perturbations using optogenetics, anatomical tracing or ablations.

To better see the anatomical structures of OT, we acquired high-resolution confocal images of the OT from fish C (randomly chosen) immediately after the voltage imaging experiment. Confocal imaging provided voxel sizes of 0.16 × 0.16 × 1 µm, compared to 0.73 × 0.73 × 5.86 µm for the light-sheet data. After excluding artifact-affected ROIs, we co-registered functional and structural datasets and overlaid firing neuron centroids onto multiple *z*-sections (Supplementary Fig. 16). Although the functional data from fish C exhibited slightly more complex firing patterns than those of fish A, B, and D, the overall lateral-to-medial activation sequence remained evident. While the confocal data did not qualitatively alter our interpretation, this experiment demonstrates our technique’s potential to be integrated with higher-resolution structural modalities, including future applications such as expansion microscopy, ExFISH^47^ or ExSEQ^48^.

**Fig. 5:**
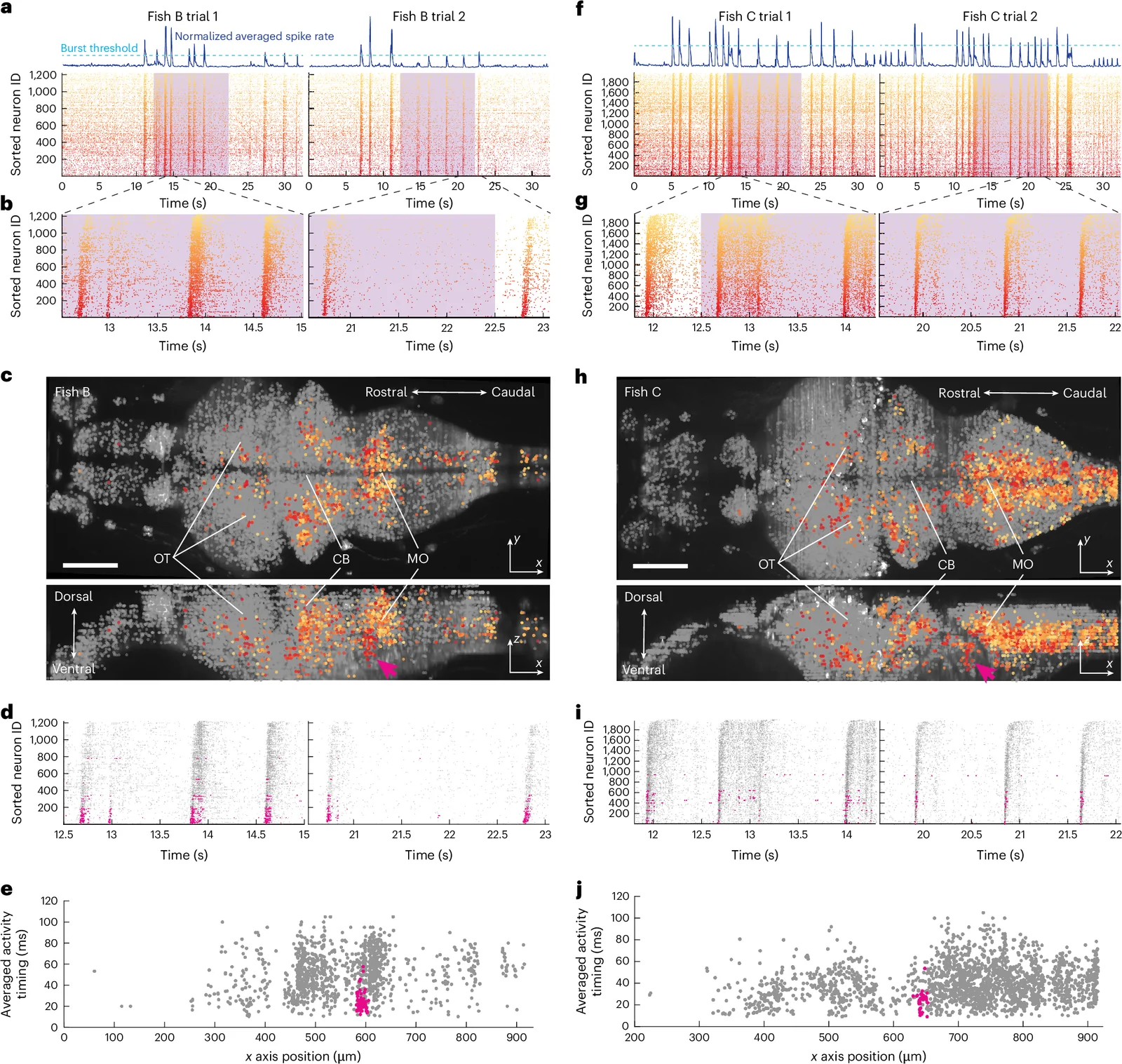
Spatial mapping of activity timing within burst sequences across the zebrafish brain. **a**, Raster plot showing burst activity in group 2 neurons during light-stimulation experiments in fish B. These neurons correspond to the orange group in the UMAP embedding in Fig. 3b. A burst was defined as a period during which the average spiking rate, computed over a 70-ms sliding window, exceeded five times the mean spiking rate over the entire trial. Burst onset was defined as the first time point at which this threshold was crossed. Neurons were sorted by their activation timing, averaged across all burst events. The two trials were separated by a 20-min dark interval. The UV stimulation period is indicated in purple. ROIs affected by moving-stripe artifacts were computationally identified and excluded from all analyses in this figure. **b**, Enlarged views of a 2.5-s time window in **a**. Activation order is color-coded from early (deep red) to late (light orange). **c**, 3D spatial locations of the neurons shown in **a**, overlaid on dorsal (top) and lateral (bottom) MIPs of the fish B brain. Neurons are color-coded by burst activation timing using the same color scale as in **b**. The magenta arrow indicates a putative physical cluster of early-firing neurons. Scale bar, 100 µm. **d**, Neurons within the putative physical cluster indicated by the magenta arrow in **c** are shown in magenta in the sorted raster plot. The remaining group 2 neurons are shown in gray. **e**, Relationship between neuronal position along the *x* axis and burst activation timing, averaged across all burst events in both trials. Each dot represents a neuron; neurons in the putative physical cluster are shown in magenta, and all other neurons are shown in gray. **f**–**j**, Same analyses as in **a**–**e** for fish C.

We next examined group 2 neurons, which exhibited spontaneous burst activity in all four fish (Fig. 5). All analyses were performed on clean traces after computational artifact removal. To define burst events, we smoothed each neuron’s spike raster with a 70-ms Hanning window and computed the population-averaged spiking rate (Fig. 5a). A burst was detected when the population rate exceeded five times its average value across the two trials. This threshold was chosen somewhat arbitrarily, to isolate burst events. Bursts were not perfectly synchronized across neurons; instead, neurons fired at slightly offset times within each event.

**Extended Data Fig. 10:**
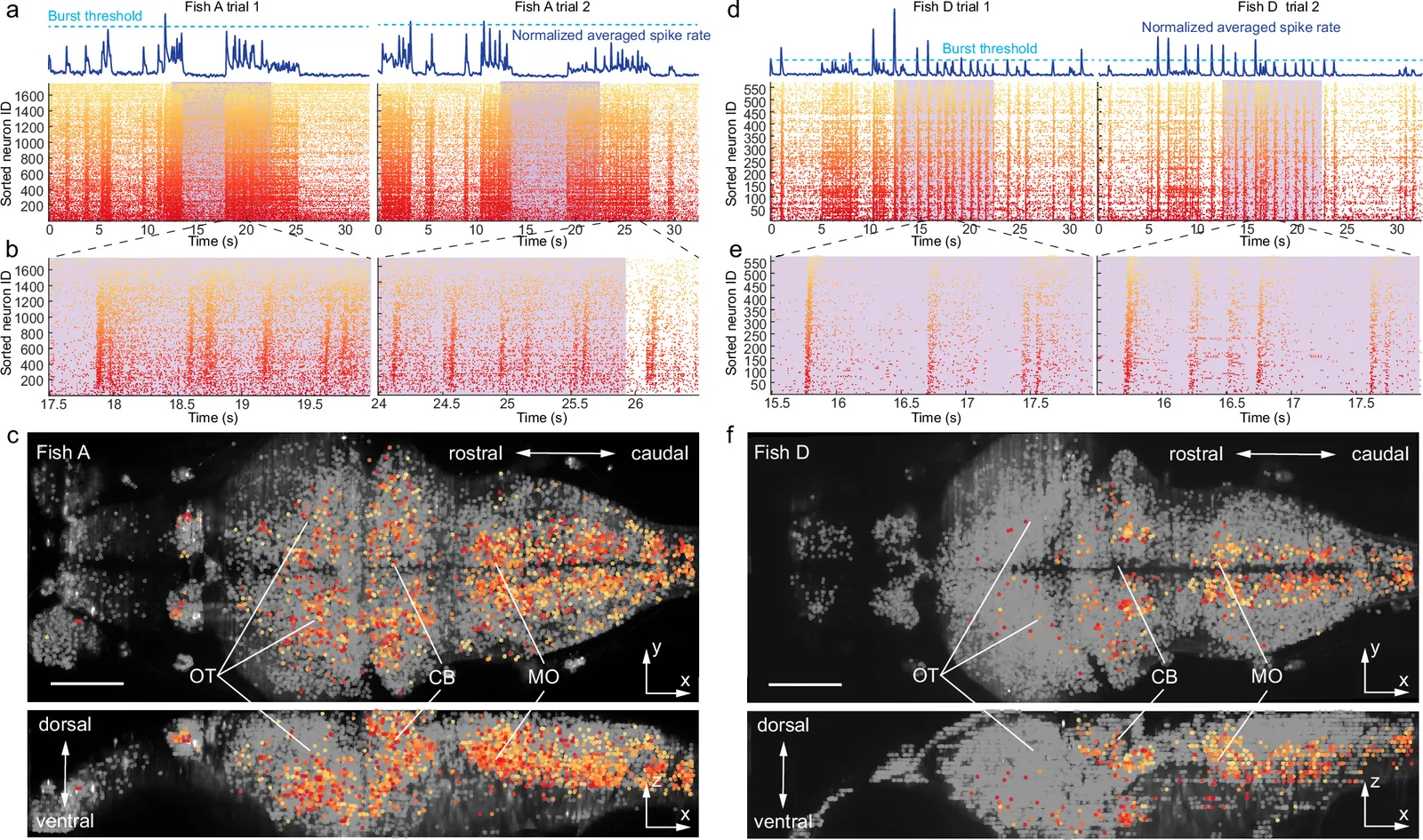
Spatial mapping of activity timing within burst sequences across the zebrafish brain (Fish A and Fish D). **(a)** Raster plot showing burst activity in Group 2 neurons during light stimulation experiments in Fish A. A burst was defined as a period during which the average spiking rate, computed over a 70-ms sliding window, exceeded five times the mean spiking rate over the entire trial. Burst onset was defined as the first time point at which this threshold was crossed. Neurons were sorted by their activation timing, averaged across all burst events. The two trials were separated by a 20-min dark interval. The UV stimulation period is indicated in purple. Regions of interest (ROIs) affected by moving-stripe artifacts were computationally identified and excluded from all analyses in this figure. **(b)** Enlarged views of a 2.5-s time window in (a), illustrating a consistent sequence of neuronal activation within bursts. Activation order is color-coded from early (deep red) to late (light orange). **(c)** Three-dimensional spatial locations of the neurons shown in (a), overlaid on dorsal (top) and lateral (bottom) maximum-intensity projections (MIPs) of the Fish A brain. Neurons are color-coded by burst activation timing using the same color scale as in (b). OT, optic tectum; CB, cerebellum; MO, medulla oblongata. Scale bar, 100 µm. **(d-f)** Same analyses as in (a–c) for Fish D.

To test whether these timing differences were consistent, we identified each neuron’s peak firing time within each burst and ordered neurons by their average peak time across all bursts (Fig. 5a,b,f,g and Extended Data Fig. 10). This ordering was highly consistent: neurons that fired early in one burst tended to fire early in subsequent bursts. Thus, there was a consistent ordering of neuron firing, within these nearly synchronized bursts.

We then asked whether this temporal ordering of group 2 neurons corresponded to spatial organization. In fish B and C, many of the earliest-firing neurons were concentrated within a compact volume (~20 × 110 × 40 µm^3^; Fig. 5c–e,h–j). Visual inspection suggested that this cluster lies near rhombomere 5 and may correspond to the MiD2 reticulospinal neuron cluster. Previous calcium imaging during fictive swimming showed that MiD2 neurons are involved in bilateral swimming but could not resolve activation order due to slow imaging rates^49^. Our experiment did not include behavioral measurements, so we cannot assign functional identity or behavioral relevance to these neurons. Nevertheless, the ability to resolve consistent temporal ordering within this cluster highlights the potential of voltage imaging to dissect fast dynamics in motor-related circuits.

A comparable early-firing cluster was not observed in fish A and D (Extended Data Fig. 10). Unlike group 1 activity, which was driven by a controlled stimulus, group 2 bursts were spontaneous and uncorrelated with UV illumination. The inter-fish variability may therefore reflect differences in internal state or behavior that were not monitored. While we cannot interpret these differences mechanistically, the observed consistency and variability in these bursts could lead to new hypotheses for future research.

## Discussion

We present a remote-scanning LSM (rsLSM) optimized for imaging membrane voltage from individual neurons distributed across much of the entire larval zebrafish brain. Our technique enables access to neural dynamics that would be too fast and too distributed, to be captured with previous technologies.

The ability to image voltage of neurons distributed across much of the brain opens immediate opportunities for systems neuroscience. Combined with sensory stimulation and fictive behavior paradigms (and potentially, in future implementations, freely moving preparations) this approach could be used to investigate questions such as the circuit mechanisms underlying brain-wide state transitions, such as sleep-related up and down states, or the maintenance of excitation–inhibition balance across distributed networks. With appropriate spectral separation, red-shifted voltage indicators^50^ and blue-shifted optogenetic opsins could be integrated, potentially alongside holographic stimulation^51^, to enable simultaneous perturbation and monitoring of voltage activity across large neural populations.

While the present work demonstrates that voltage imaging of neurons distributed across much of the brain is now feasible, substantial opportunities remain for improvement. Advances in GEVI design, such as increased brightness, sensitivity, imaging-rate-matched kinetics, and improved photostability, will directly enhance signal fidelity. On the instrumentation side, higher spatial and temporal resolution and improved photon efficiency are always desirable. At present, the imaging rate is limited by the camera pixel throughput; however, with the current optical design, imaging rates exceeding 1 kHz are achievable for smaller 3D-FOVs. In practice, whole-brain imaging at 200 Hz and kilohertz-rate regional imaging could be combined within a single experiment to interrogate neural dynamics across scales. As faster and more sensitive camera sensors become available, voltage imaging across whole brains with submillisecond temporal resolution is likely to become attainable.

Additional optical refinements are also possible. For example, introducing a second light sheet from the front side of the fish could illuminate brain regions currently shadowed by the eyes. Scattering could be reduced through the use of longer-wavelength excitation, red-shifted indicators, or multiphoton light-sheet excitation. Post hoc anatomical and molecular analyses, such as expansion microscopy^52^, neuron barcoding^53^ and in situ sequencing^48^, could further link observed dynamics to underlying structure and gene expression.

Finally, the scale of data generated by large-scale voltage imaging necessitates continued development of algorithms and software for efficient processing and analysis. While existing denoising and spike-extraction frameworks can be adapted, new algorithms are needed for efficient neuron segmentation, signal demixing in densely labeled tissue, and principled interpretation of distributed voltage dynamics.

## Methods

### High-speed remote-scanning light-sheet microscope

For the detection arm, fluorescence emission from the sample was collected by a high-NA detection objective (OL1, *f* = 9 mm, NA = 1.0, XLUMPLFLN20XW, Olympus). The emitted light then passed through a 4f relay system consisting of two tube lenses (L1, *f* = 200 mm, TTL200MP, Thorlabs; and L2, *f* = 150 mm, TTL200-A combined with two AC508-750-A achromats, Thorlabs) and entered a PBS (CCM1-PBS251, Thorlabs). The polarization component reflected by the PBS propagated through a quarter-wave plate (QWP; AQWP10M-580, Thorlabs) and a remote objective lens (OL2; *f* = 9 mm, NA = 0.8, UPLXAPO20X, Olympus), forming a virtually spherical-aberration-free three-dimensional image of the sample. This image was reflected by a lightweight silver-coated mirror (2 × 2 × 1 mm^3^, Chroma) mounted on a piezo bender actuator (PB4VB2S, Thorlabs). After reflection, the emission light retraced its path through the QWP, which rotated its polarization by 90° on the second pass. As a result, the light passed through the PBS instead of being reflected and was directed to a tertiary tube lens (L3; *f* = 176 mm, TTL200MP combined with AC508-1000-A, Thorlabs), forming an in-focus image at its focal plane. A band-pass emission filter (FF01-571/72-25, Semrock) was placed between the PBS and L3.

To achieve the kilohertz frame rates required for voltage imaging across most of the zebrafish brain, the image formed by L3 was split and captured by two ultrafast CMOS cameras (Ximea CB024MG-GP-X8G3; or a customized prototype camera in earlier experiments; Extended Data Fig. 1a), effectively doubling the achievable frame rate. A knife-edge mirror (KEM; MRAK25-P01, Thorlabs), aligned with the image’s central longitudinal axis, divided the image into two halves. Each half was relayed by an identical 4f system (*f* = 211 mm, ACT508-1000-A combined with three ACT508-750-A achromats, Thorlabs; followed by a 4× objective, TL4X-SAP, Thorlabs, or Nikon CFI Plan Apo 4x) and imaged onto one of the two cameras.

For fluorescence excitation, we constructed two light-sheet excitation configurations: a standard light-sheet arm for voltage imaging without light-sheet pivoting (Extended Data Fig. 1e) and a modified arm incorporating resonant scanning for stripe suppression (Extended Data Fig. 1f).

In the standard configuration, a collimated 515-nm laser beam (beam diameter ~1.5 mm; Cobolt 06-MLD, HÜBNER Photonics) passed through a Powell lens (5° fan angle, Laserline Optics) to form a divergent fan beam in the horizontal plane. This beam was relayed by a 4f system (RL2, *f* = 50 mm; RL1: *f* = 75 mm; Thorlabs) to a single-axis galvo mirror (5-mm aperture, Saturn 5B, ScannerMAX). The galvo mirror reflected the beam into a scan lens (CLS-SL, Thorlabs), which focused it into a horizontal light sheet at its focal plane. This light sheet then passed through a 0.25×, reversed microscope consisting of a tube lens (ITL200, Thorlabs) and an excitation objective (Plan Apo Lambda ×4, Nikon) to illuminate the sample.

At the sample, the light sheet had an approximate width of 1 mm, about 20% larger than the length of the zebrafish brain. Axial scanning of the light sheet was achieved by vertically scanning the galvo mirror. To prevent excitation light from directly entering the eyes, an optical mask was placed at the scan lens’s focal plane. The mask consisted of a 3-mm-thick glass substrate (14 × 14 mm) with a circular chromium coating (diameter 900 µm, optical density >5; PhotomaskPORTAL). This mask blocked excitation light over an approximately 225-µm-diameter region at the eye plane, larger than the pupil but smaller than the entire eye.

The second excitation configuration (Extended Data Fig. 1f) was based on the standard design but incorporated several modifications to enable rapid light-sheet pivoting for stripe suppression. First, an 8-kHz resonant mirror scanner (RESSCAN, Sutter Instruments) was installed between two relay lenses (RL3 and RL4). Second, the galvo mirror was replaced with a larger-aperture model (10-mm aperture, Saturn 9B, ScannerMAX) to accommodate the rapid horizontal scanning introduced by the resonant scanner, which caused the beam to sweep across an ~8-mm range on the galvo mirror. Third, the excitation objective was replaced with a higher-NA lens (XLFLUOR4X/340, Olympus; NA = 0.28) to allow a larger pivoting angle. Fourth, the relay lenses were adjusted to match the excitation beam size to the resonant scanner aperture (RL3, *f* = 100 mm, achromatic doublet; RL4, *f* = 75 mm, achromatic doublet; Thorlabs). Additionally, an adjustable mechanical slit (VA100CP, Thorlabs) was inserted between RL3 and the galvo mirror to control light-sheet thickness and divergence. This slit was unnecessary in the standard configuration, where the light-sheet parameters were already optimized for larval zebrafish.

In the resonant-scanning configuration, however, slightly reducing the slit width improved image quality by decreasing light-sheet divergence. This adjustment compensated for the increased axial motion blur associated with resonant scanning, where the focal plane shifted by ~6.1 µm during each exposure. Near the light-sheet waist, motion blur was the dominant limiting factor, allowing some relaxation of thickness constraints, whereas reducing divergence improved resolution at locations farther from the waist, at the expense of increased thickness near the waist.

To enhance light efficiency, we added a second remote-scanning module identical to the first on the orthogonal port of the PBS (Fig. 1a). Each module comprised a QWP, a remote objective lens, and a piezo-driven lightweight mirror. Alignment between the two modules was achieved using a two-step calibration procedure with a three-dimensional fluorescent bead sample. First, the lateral (*x*–*y*, perpendicular to its optical axis) position of the second remote objective lens was adjusted to align bead images from the two modules across a series of static *z*-planes, achieving subpixel alignment over a 200-µm axial range (Supplementary Fig. 1). Second, during volumetric imaging, the drive waveform of the second piezo bender was iteratively adjusted on a plane-by-plane basis to synchronize its focal position with that of the first module. This procedure was analogous to synchronizing axial light-sheet scanning with the motion of the primary remote mirror.

A full list of components is provided in Supplementary Table 2.

### Volumetric scanning control and hardware synchronization

High-frequency remote-mirror scanning was implemented using an open-loop piezo bender actuator. To compensate for piezo hysteresis, the mirror motion was directly measured and the drive waveform was iteratively calibrated. Mirror displacement was monitored at 70 kHz using a line-scan camera coupled to a custom microscope with a ×20 objective. For precise displacement measurement, a miniature imaging target with fine grid patterns, fabricated by two-photon polymerization, was attached to the side of the mirror. During calibration, the amplitudes and phases of high-order sinusoidal components in the piezo drive waveform were adjusted in LabVIEW to counteract hysteresis. This procedure was repeated until the deviation between the measured and target waveforms was reduced to <0.1% of the total scan range.

To improve uniformity of axial sampling and camera frame intervals, the remote mirror was driven with a non-sinusoidal waveform. Specifically, sinusoidal components at odd-integer multiples of the base volume scan frequency (200.8 Hz, 602.4 Hz and 1,004 Hz) were added to approximate a triangular waveform up to the third-order Fourier series. Compared with a pure sinusoid, this waveform yielded more uniform axial scanning. Stability tests showed that the piezo actuator exhibited high displacement consistency under periodic open-loop drive, with a maximum deviation of <0.5% over one minute, allowing long-term stable volumetric imaging.

Remote mirror motion directly determines the axial position of the microscope focal plane. For calibration of the galvo drive waveform to maintain overlap between the scanned focal plane and the illumination light sheet, the light sheet was temporally gated using short excitation pulses. Specifically, 40-µs laser pulses were introduced at defined time delays within each scan cycle while the remote mirror and illumination galvanometer were operating. These short pulses effectively sampled stationary focal planes on the camera. By adjusting data points of the galvanometer drive waveform corresponding to the pulse timing, we ensured the sampled plane was in-focus. Repeating this procedure across different pulse delays ensured co-alignment of the light sheet and focal plane throughout the entire scan cycle.

Each volumetric scan cycle lasted 4,980 µs and included both upward and downward scans. Within each cycle, 30 axial planes spanning the zebrafish brain were imaged in an interleaved sequence (Fig. 1c). To minimize motion blur arising from continuous axial scanning during image acquisition, illumination was restricted to 40-µs pulses for each plane. Pulse timing was calibrated to ensure uniform axial sampling. Camera exposures were synchronized to the laser pulses and set to be 10 µs longer than the pulse duration, ensuring full capture of the excitation period. The relative timing between exposure and illumination was carefully adjusted.

Coordinated, low-latency control of all hardware components was achieved using a real-time LabVIEW program implemented on a compactRIO system with an onboard FPGA (cRIO-9038, National Instruments). Analog and digital outputs were generated through NI-9262 and NI-9401 I/O modules. Control signals were updated every 4 µs, one-tenth of the exposure duration per frame and repeated every 4,980 µs, corresponding to a volumetric imaging rate of 200.8 Hz.

### Customized high-speed camera

During early system development, no commercial cameras incorporating the Gpixel GSPRINT4502 image sensor were available. We therefore designed a custom camera using this sensor (Extended Data Fig. 1a). The custom camera uses the Gpixel GSPRINT4521/10/02 image sensors. The camera system’s hardware employs a two-board stacked design, comprising the TOP and BOTTOM boards (Extended Data Fig. 1a). The TOP board, an 18-layer printed circuit board, houses the image sensor chip and a Xilinx Spartan 6 FPGA (Opal-Kelly XEM6010) used for camera control and acquisition trigger synchronization. The TOP board accommodates 144 pairs of low-voltage differential signaling (LVDS) lines for high-speed data output from the image sensor. These LVDS line trace lengths are carefully matched to minimize propagation delays during data transmission, operating at 1.2 Gbps per pair in double data rate format. The data lines are connected to the BOTTOM board via two high-pin count FPGA mezzanine card connectors.

The BOTTOM board (Numato Nereid K7) contains a Xilinx Kintex-7 FPGA, which buffers the high-speed data from the image sensor and transmits it to the host computer. Each LVDS’ line data delays are measured relative to the data clock at power-up. This delay is then compensated at the FPGA side to ensure data integrity at receiving high-speed data stream at 1.2 Gbps. The received data are buffered and rearranged using the onboard RAM, before transmission to the host computer using a PCIe link. The PCIe communication firmware is modified based on open-source projects: RIFFA and Open-Ephys ONIX. The host PC receives the data using an ANSI-C API from Open-Ephys ONIX^54^. The data are displayed, and storage is designed, using the Bonsai^55^ reactive programming language.

The custom camera satisfied our design requirements of spatiotemporal resolution (>6,000 FPS with 258 × 1,280 C-FOV) and noise (<7 e^−^). The only drawback is its large size as the camera prototype requires the PC to be placed nearby to access the PCIe slot. This could be solved in the future by adopting existing PCIe cable connectors, such as the Molex iPass connector system.

Commercial cameras using GSPRINT4521/10/02 sensors became available near the end of our custom camera’s development. We found that both our custom camera and the commercial camera (Ximea CB024MG-GP-X8G3) had the same readout noise performance at our operating speed. Readout noise was quantified as root-mean-square noise measurement in dark, calculated by taking the standard deviation of the pixel’s values in the absence of light.

### Transgenic zebrafish line construction and transient ASAP5-Kv expression

The *Tg(HuC:Gal4; 3×UAS:Positron2-Kv); nacre* and *Tg(HuC:Gal4; 2×UAS:Positron2-Kv); nacre* transgenic zebrafish lines were generated by embryonic microinjections using the Tol2 transposase system^56^. Positron2-Kv plasmids were designed in house and synthesized commercially (Epoch). Each DNA construct contained tandem UAS sequences^57^, the Positron2 gene, and a Kv2.1 soma-targeting sequence^24^, and was inserted between Tol2 arms in a Tol2 vector. To facilitate membrane trafficking, Kir2.1 and ER export sequences were inserted between the Positron2 gene and the Kv2.1 sequence^58^. Full DNA sequences of the Positron2-Kv constructs are provided in Supplementary Table 3.

For fish line generation, Tol2 plasmids (40 ng µl^−1^) were co-injected with Tol2 transposase mRNA (synthesized using the SP6 Transcription kit, Thermo Fisher) into single-cell stage zygotes from *Tg(HuC:Gal4); nacre* fish. At 3 dpf, embryos were incubated for 2 h in fish-facility water containing 3 μM JF525-HaloTag, followed by gentle washing in fresh fish-facility water. Embryos were screened for green fluorescence under a fluorescence stereoscope, and fluorescent larvae (F0) were raised to adulthood. At 3 months post-fertilization, F0 adults were outcrossed with either *Tg(HuC:Gal4); nacre* or *nacre* fish. Progeny were stained with JF525-HaloTag dye and screened at 3–4 dpf and larvae exhibiting pan-neuronal fluorescence were selected and raised to establish stable transgenic lines.

For transient expression of ASAP5-Kv, DNA plasmids containing a 2×UAS:ASAP5-Kv construct flanked by Tol2 transposon arms were co-injected with Tol2 transposase mRNA into single-cell-stage zygotes of *Tg(HuC:Gal4); nacre* zebrafish. Injected embryos were screened for fluorescence at 3 dpf. Positive larvae were paralyzed and embedded in agarose for imaging at 5 dpf.

### Larval zebrafish preparation for imaging

Pan-neuronally labeled larval zebrafish (5–6 dpf) were embedded and imaged using our high-speed microscope. At that age, larval zebrafish have not yet undergone sexual differentiation. For Positron2-Kv fluorescence labeling, fish were incubated in JF525-HaloTag ligand dye solution for 2 h. To minimize the presence of neurons generated after staining and thus remaining unlabeled, the interval between staining and imaging was kept as short as possible. Fish expressing detectable fluorescence were subsequently screened and selected for imaging.

To reduce motion-induced artifacts, fish were paralyzed by brief incubation in fish-facility water containing 0.3 mg ml^−1^ pancuronium bromide (Sigma) until no movement was observed in response to tactile stimulation with closed forceps tips. Fish were then mounted on a 3D-printed holder (Supplementary Fig. 17) and embedded in 3% low-melting-point agarose (Sigma) within a Petri dish (Fig. 1d). The holder was designed to cradle the torso in a groove while allowing the tail to extend out from the holder.

After agarose solidification, fresh fish-facility water was added to the dish, and agarose surrounding the head, particularly on the side intended for light-sheet illumination, was carefully removed using forceps, while the rest of the body remained restrained in agarose. The holder containing the embedded fish was then transferred to a 3D-printed sample chamber with transparent glass coverslip walls (Supplementary Fig. 17) filled with fish-facility water. The holder was secured to the chamber using small magnets, and the chamber was mounted on a 3D translation stage beneath the detection objective for imaging.

All procedures related to zebrafish husbandry and handling were conducted in accordance with the US National Institutes of Health Guide for the Care and Use of Laboratory Animals and approved by the MIT Committee on Animal Care (protocols 1221-100-24 and 2411000756).

### Imaging spontaneous and light-evoked voltage activity of neurons distributed across much of the brain

Spontaneous voltage activity of neurons distributed across much of the entire zebrafish brain was captured at a volume rate of 200.8 Hz for 35 s in an imaging trial, which produced approximately 250 GB of image data. For a volumetric scan, each brain slice was illuminated with a light sheet for a duration of 40 µs. The light sheet had a temporal power of 43 mW, corresponding to an average excitation power of 10.4 mW on the sample during imaging.

We recorded neural activity during the light-stimulation experiments using the same imaging parameters as spontaneous activity recording, with the addition of light stimulation during imaging.

For light stimulation, illumination from a 405-nm LED (M405L4, Thorlabs) was focused onto the fish to produce a light spot with an approximate diameter of 5 mm. The measured intensity at the sample was 0.6 mW mm^−^^2^. To prevent contamination of the recorded images by long-wavelength LED emission, the stimulus light was passed through a 450-nm short-pass filter (FESH0450, Thorlabs). The light stimulus was turned on 13 s after the start of light-sheet illumination, remained on for 10 s, and was then turned off for the remainder of the 35 s trial. To minimize sensory adaptation, fish were kept in darkness for 20 min between consecutive imaging trials involving light stimulation.

### Data processing

#### Pre-processing

Raw data streams from each camera were parsed into 30 videos corresponding to 30 *z*-stack planes. Owing to the high data throughput from each camera to the host computer via the PCIe bus (~2.6 GB s^−1^ per camera), rare frame losses occurred because of buffer overflow (drop rate ≈1 per 2,000 frames or ~0.05%). Consecutive frame losses were rare. Missing frames were replaced by linear interpolation using pixel values from the immediately preceding and following frames.

Videos from the two cameras were synchronized by aligning their timestamps to the onset of light-sheet excitation at the beginning of each experiment. Frames from the two cameras were then merged at each time point into a single image. Each C-FOV covered approximately half of the brain (Extended Data Fig. 1b–d). The merged frame size was 512 × 1,280 pixels, encompassing the entire brain. For downstream processing, corresponding *z*-stack frames from successive trials were concatenated in time into a single video for motion correction, ROI segmentation and temporal trace extraction.

#### Motion correction

Rigid motion correction was applied independently to each z-plane using NoRMCorre^41^.

#### ROI segmentation

ROIs corresponding to putative neurons were automatically segmented using deep learning models implemented in Cellpose v.3.0^28^. For training data preparation, motion correction was first applied to each *z*-plane video. A static image for each plane was then generated by averaging the first 100 frames. These images were spatially upsampled by a factor of two in the *x–y* plane to increase pixel density and facilitate manual labeling in the Cellpose graphical user interface (GUI).

The pre-trained Cellpose v.3.0 model performed poorly on our data, likely due to limited out-of-distribution generalization. We therefore, manually segmented several thousand neurons and trained a custom Cellpose model in grayscale mode. Manual annotations targeted objects approximately the size of a neuron (~6.6 µm) with ring-like boundaries characteristic of membrane labeling. Model training used a diam_mean of 15 (matching neuron size) and a learning rate of 0.02, which was chosen somewhat arbitrarily but yielded stable convergence. After 1,000 training epochs, the model correctly segmented >70% of neurons when evaluated against hand-labeled ground truth.

During testing, ROI detection sensitivity was adjusted using the flow threshold, cellprob threshold, and diameter pixel parameters in the Cellpose GUI. Cellprob threshold and flow threshold are two parameters controlling how sensitive the model is to detect ROIs^28^. Increasing the flow threshold or decreasing the cellprob threshold increased ROI yield but also raised false positive rates. The diameter pixel parameter specifies the expected neuron diameter in pixel units. For all analyses in this study, we used a diameter pixel of 18 (matching the average neuron size in our dataset), a flow threshold of 0.4, and a cellprob threshold of 0, corresponding to the GUI defaults for the latter two parameters. We noticed that using other values could lead to over or under segmentation. We adopted Cellpose’s iterative bootstrapping strategy^28^ by retraining the model with manually corrected predictions. Training data from five fish (>80,000 ROIs in total) were used. Model performance improved as the training set expanded and plateaued after inclusion of ROIs from five fish, at which point training was terminated.

#### ROI temporal trace extraction

Temporal fluorescence traces were extracted from segmented ROIs using VolPy’s processing pipeline, which includes background subtraction, trace denoising and spike extraction. Spike detection was performed using VolPy’s adaptive thresholding. To avoid edge effects introduced by VolPy filters, the first and last 1 s of each 35 s extracted time series were excluded from subsequent analyses.

#### Computational identification and removal of ROIs affected by moving stripes

After extracting putative spiking activity from all ROIs, we identified and removed ROIs contaminated by moving-stripe artifacts, using a computational clustering approach. Each ROI’s spike raster was first smoothed with a 250-ms moving Hanning window to estimate firing rates. The full dataset was organized into an N × D matrix, where N is the number of ROIs and D the number of temporal samples. This matrix was embedded into a two-dimensional manifold using UMAP, yielding an N × 2 representation, where each ROI was represented as a point within a 2D manifold.

We then applied DBSCAN to group ROIs with similar temporal activity patterns. The cluster most visually separated from the main population in the UMAP space was identified, labeled and temporarily removed. The remaining ROIs were re-embedded and reclustered iteratively using the same UMAP–DBSCAN procedure until the UMAP distribution became approximately Gaussian.

Clusters were classified as artifact-contaminated only if they satisfied both of the following criteria: (1) their ROIs exhibited highly synchronous pulse-like or fast-fluctuating temporal patterns (Supplementary Fig. 5, orange arrows); and (2) their spatial distributions matched the characteristic geometry of moving-stripe artifacts, with ROIs confined to regions narrow in *x* and z (<100 µm) and extending along the *y* axis (light-sheet illumination axis) from a specific origin, often within the brain, to the edge opposite the light-sheet incidence (Supplementary Fig. 5, green arrows). ROIs from clusters meeting both criteria were excluded from further analysis. The remaining ROIs were treated as putative neurons and retained for downstream analyses.

#### Clustering based on putative neuron activity

After removal of artifact-contaminated ROIs, the remaining ROIs (putative neurons) were reclustered using UMAP and DBSCAN based on their temporal spiking characteristics.

### Immunostaining of health markers

Larval zebrafish subjected to light-sheet exposure (200.8 Hz light-sheet scanning, 10.4 mW total power, 70 s continuous exposure matching two 35-s imaging trials) were fixed either 1 h post-exposure for γH2AX or after overnight incubation for the other markers (NeuN, cleaved caspase-3, synaptophysin). Larvae were immersed in 4% paraformaldehyde (PFA) at 4 °C for 14 h (γH2AX) or 22 h (NeuN, cleaved caspase-3, synaptophysin), quenched 6 h at 4 °C in 100 mM glycine/PBS, and rinsed 6 × 20 min in PBS. Brains were manually dissected into PBS containing 4′,6-diamidino-2-phenylindole (DAPI) (for NeuN and synaptophysin, PBS with DAPI + 0.2% Tween-20, ‘PBT’). For NeuN only, samples were incubated in methanol for 15 min at room temperature (RT), then fresh methanol for 2 h at −20 °C, followed by 5-min rinses in 75%, 50% and 25% methanol in PBT and 3 × 5 min in PBT. For all markers, antigen retrieval was performed in 150 mM Tris (pH 9.0): 5 min at RT then 15 min at 70 °C, followed by rinses in PBS (γH2AX, cleaved caspase-3) or PBT (NeuN, synaptophysin). For γH2AX and cleaved caspase-3, samples were blocked 1 h at RT in PAT (PBS + 3% BSA + 0.1% Triton X-100), incubated with primary antibody in PAT at 4 °C overnight, rinsed 2 × 30 min and 2 × 60 min in PAT, incubated with secondary in PAT at 4 °C overnight, then rinsed 2 × 30 min in PAT and 2 × 60 min in PBS. For NeuN and synaptophysin, samples were rinsed in ice-cold PBT, incubated 10 min at 4 °C in 0.05% trypsin in PBT, rinsed in ice-cold PBT then 3 × 5 min at RT in PBT, blocked 1 h at RT in PGADT (PBS + 2% goat serum + 1% BSA + 1% dimethylsulfoxide + 0.25% Triton X-100), incubated with primary antibodies in PGADT at 4 °C for 3 days, rinsed 8 × 30 min in PBT, incubated with secondary antibodies in PGADT at 4 °C for 2 days and rinsed for 8 × 30 min in PBT. Primary antibodies and dilutions were anti-γH2AX (GeneTex, cat. no. 127342, 1:200 dilution), anti-NeuN (Abcam, ab177487, 1:100 dilution), anti-cleaved caspase-3 (BD, 559565, 1:200 dilution), and anti-synaptophysin (Abcam, ab32594, 1:100 dilution). All primaries were detected with donkey anti-rabbit IgG (H + L) Alexa Fluor Plus 647 (Thermo Fisher, A32795TR, 1:200 dilution). Brains were embedded in low-melt agarose and imaged by confocal microscopy using a 40× objective, centering stacks on midbrain; excitation was at 405 nm (DAPI, 100% power and 200 ms exposure) and 640 nm (Alexa Fluor 647, 100% power) with exposure times of 50 ms (synaptophysin), 200 ms (cleaved caspase-3, NeuN) or 500 ms (γH2AX). The *z*-image-stacks comprised 30–50 optical sections at 2-µm steps.

### Neuron density estimation

Based on the literature^29^, for a 7-dpf zebrafish, there are on average 78,000 neurons in a volume of approximately 0.20 mm^3^. Assuming the neurons are distributed evenly, we can calculate the volume occupied per neuron as 2.6 × 10^−6^ mm^3^. If we assume each neuron occupies a cubic volume, then the length of each side of this cube is the cube root of the volume per neuron, which is 14 μm. This can be interpreted as the average distance between the centroids of neurons in the zebrafish brain.

### Reporting summary

Further information on research design is available in the Nature Portfolio Reporting Summary linked to this article.

## Online content

Any methods, additional references, Nature Portfolio reporting summaries, source data, extended data, supplementary information, acknowledgements, peer review information; details of author contributions and competing interests; and statements of data and code availability are available at https://doi.org/10.1038/s41592-026-03179-7.

## Supplementary information

Supplementary Information Supplementary Notes 1–12, Figs. 1–17 and Tables 1–3.

Reporting Summary

Peer Review File

Supplementary Video 1 A representative stripe artifact (shown in Supplementary Fig. 4). The light sheet illuminates the sample from the left side (green arrow). The location of the stripe artifact is indicated by the white arrows. Scale bar, 100 µm.

Supplementary Video 2 BDM treatment removed moving stripes in the live zebrafish brain (shown in Extended Data Fig. 3b). This video shows the raw light-sheet recordings of four randomly selected planes (out of 30) of the same zebrafish brain during a BDM-treatment experiment. The fish had pan-neuronal Positron2-Kv expression and was imaged at 6 dpf. Moving stripes that were visible before BDM treatment (pre-BDM) disappeared after the BDM treatment (post-BDM). After the fish recovered in fresh water (recovered), the moving-stripe patterns appeared again. Green arrows on the left side indicate the direction of light-sheet illumination. Scale bar, 100 µm.

Supplementary Data 1 Fish holder model file.

Supplementary Data 2 Imaging chamber model file.

Supplementary Data 3 Reporting table.

## Source data

Source Data Fig. 2 Trace plot data.

Source Data Fig. 3 Trace plot data.

Source Data Extended Data Fig. 5 Histogram plot data.

Source Data Extended Data Fig. 7 Photobleaching measurement data.

Source Data Extended Data Fig. 8 Immunostaining of health markers statistical data.

## Data Availability

Raw light-sheet imaging data, analysis results and other raw and processed data associated with this paper, are publicly available at https://doi.org/10.6019/S-BSST2153 ref. ^59^. Source data are provided with this paper.

## Code availability

The codes used to control the microscope and to analyze the image datasets, as well as the custom-trained Cellpose model, are available at Zenodo at https://doi.org/10.5281/zenodo.14920628 (ref. ^60^) under the Creative Commons Attribution 4.0 International License.
