# Supplementary material for: Voltage imaging of neurons distributed across entire brains of larval zebrafish
Source: Nat Methods. 2026 Aug 14;23(9):1895–907. doi: 10.1038/s41592-026-03179-7 (PMC13541626; doi:10.1038/s41592-026-03179-7)
Supplement: Supplementary file 1 [file 41592_2026_3179_MOESM1_ESM.pdf]

nature methods

Supplementary information https://doi.org/10.1038/s41592-026-03179-7

Voltage imaging of neurons distributed across entire brains of larval zebrafish

In the format provided by the authors and unedited

Supplementary Notes

1. Discussion of microscopy techniques for voltage imaging across the entire brain

Several imaging techniques hold potential for voltage imaging across whole brains, each with distinct advantages and limitations. Light-field microscopy (LFM)[1,2] holds great promise for population-level voltage imaging[3] due to its high volumetric imaging rate, single-shot acquisition, compatibility with widefield illumination, and simple hardware implementation. However, applying LFM to whole-brain voltage imaging in larval zebrafish presents two key challenges stemming from the intrinsic properties of LFM.

The first challenge is spatial resolution. In voltage imaging, fluorescent signals arise from neuronal membranes, which appear as cell contours in raw images. These neuronal contours contact each other in a densely labeled zebrafish brain, necessitating a spatial resolution much smaller than the neuronal cell body size (3-7 μm)[4] to distinguish individual GEVI-labeled neurons. Current LFM implementations achieve, at best, ~3 μm lateral resolution when imaging the entire ~200-μm-thick larval zebrafish brain in a single snapshot[5,6]. This limitation comes from an inherent trade-off in LFM between spatial resolution and axial field-of-view. Although spatial resolution can be improved by incorporating optical sectioning[7] or galvo scanning[8], these approaches require multiple snapshots to cover the full brain volume, substantially reducing the volumetric imaging rate.

The second challenge for LFM in voltage imaging is low signal-to-noise ratio (SNR). This arises from increased shot noise caused by the mixing of fluorescence signals from different axial planes (z-planes) in raw light-field images. This increased shot noise is inherent and cannot be efficiently compensated. For dense labeling across the entire larval zebrafish brain, we estimate that LFM would exhibit more than 4× higher shot noise than light-sheet microscopy (LSM). As a rough estimate, extracting signals from a neuron in LSM involves baseline fluorescence originating from no more than two axial layers (~6 μm per layer; neurons may reside at the interface of adjacent layers, yielding a total thickness of ~12 μm). In contrast, baseline fluorescence in LFM includes contributions from the entire brain volume (~200 μm thick), leading to shot noise at least $\sqrt{200/12} \sim 4.08$ times higher than in LSM.

Light-sheet microscopy (LSM) is an another promising technique for volumetric fluorescence imaging, as it selectively excites and efficiently images fluorophores in a two-dimensional (2D) focal plane, offering high throughput, strong optical sectioning, low phototoxicity, and good spatial resolution (down to ~0.3 μm lateral)[9–12]. Using conventional LSM, whole-brain calcium imaging with cellular-resolution has been demonstrated in larval zebrafish at volumetric imaging rates of 0.8-3 Hz[13,14]. However, it is difficult to scale conventional LSM to volume rates suitable for voltage imaging while maintaining large 3D-FOV (~900×370×200 μm^3) required to image the entire larval zebrafish brain at cellular resolution [9,11,12].

This difficulty arises from two factors. First, in conventional LSM, volumetric imaging is performed by physically translating the detection objective or the sample to scan the focal plane through the sample. Such strategies are difficult to extend to thick samples (>200 μm) at high volume rates (>100 Hz) because of the limited scanning range and speed of objective scanners. At the current

moment, the fastest off-the-shelf objective scanner (PI P-726) can operate at 560 Hz but over only a 100-μm range. Scanners with larger travel ranges (up to several millimeters) typically operate at frequencies below 100 Hz. Second, LSM volume rate is limited by camera acquisition speed. The volume rate equals the camera frame rate divided by the number of image planes per volume. Widely used scientific cameras (e.g., Hamamatsu ORCA Flash, Andor Zyla) offer full-speed pixel rates of ~0.5 × 10^9 pixels per second. When imaging a whole-brain 3D-FOV using a 512 × 1280-pixel camera field-of-view (C-FOV), this corresponds to a maximum frame rate of ~400 Hz, yielding a volume rate of only 10–15 Hz.

To overcome these limitations, numerous strategies have been proposed to increase LSM scan rates. One prominent approach is remote refocusing [15,16], which rapidly shifts the system's focal plane at a remote site using a tunable lens[17], translational tertiary objective lens[18], or translational mirror [19,20]. Tunable-lens-based systems introduce additional aberrations that limit numerical aperture (NA) and two-dimensional field-of-view (2D-FOV, defined as the area a microscope can image in a single two-dimensional focal plane); reported implementations are limited to ~0.3 NA over a 600-μm-diameter 2D-FOV[17]. Aberrations can be eliminated using translational tertiary objectives or mirrors; however, scanning a tertiary objective encounters the same speed and range limitations as conventional objective scanning. Prior mirror-based implementations employed commercial mirrors weighing several grams and actuators designed for heavy loads, resulting in scanning rates below 100 Hz and scanning ranges below 100 μm, with reported lateral 2D-FOVs smaller than Φ200 μm², likely due to high magnification ratios and optical aberrations[19,20].

An alternative strategy is swept confocally-aligned planar excitation microscopy (SCAPE) [21,22] or oblique plane microscopy (OPM)[23–27], which laterally sweeps an obliquely oriented focal plane using galvo or polygon scanners, enabling volume rates approaching scanner limits. SCAPE 2.0[21] can image a 197×293×78 μm^3 volume at a high rate of 321 volumes per second (VPS), or a 345×278×155μm^3 volume at 100 VPS. However, oblique alignment between the secondary and the tertiary objectives has limited the effective NA to ~0.35, corresponding to a ~10× lower light collection than a 1.0 NA lens. This makes whole-brain voltage imaging challenging, given that voltage imaging is far lower in signal-to-noise (SNR) than calcium imaging (indeed, in this paper, a 10× reduction in light collection for the currently proposed microscope would bring SNR for many neurons below acceptable levels). To increase effective NA, several new OPM configurations were proposed recently, where water chambers[23] and customized objectives[24,25] were used to reduce the misalignment angle between the light cones of the secondary and the tertiary objectives. These strategies have enhanced effective NAs up to the NAs of the detection objectives (e.g., NA = 1). While these OPMs possess high NA and the potential to scan volumes at high speeds, the light refraction on the secondary objective's oblique image plane and the oblique tertiary objectives cause extra aberrations that reduce the diffraction limited 2D-FOV. Recently, a specifically designed, glass-tipped tertiary objective lens[24] enabled an 800×420μm^2 2D-FOV at NA=0.97 in OPM. Theoretically, this SCAPE/OPM configuration could be further adapted for imaging the voltage across whole larval zebrafish brains, given sufficient optimization is made.

2. Point-spread-function (PSF) characterization

To measure the full-widths-at-half-maximum (FWHMs) of the lateral PSF (x-y), we fitted the PSF using a 2-D Gaussian function **(Supplementary Fig. 3a)**. The x-y position and the shape of the Gaussian function were iteratively updated until the correlation was maximized between the measured lateral PSF and the 2-D Gaussian function image that was down-sampled to the same 0.73-μm effective pixel size **(Supplementary Fig. 3a)**. Then lateral FWHMs of the PSF were measured as the FWHMs of the fitted Gaussian function. To measure the axial (z-axis, defined as the axis perpendicular to the microscope's focal plane. Z-axis roughly aligns with the ventral-dorsal axis in the fish brain.) FWHM of the PSF, we used a one-dimensional dual-Gaussian function to fit the axial line profile that contained the brightest pixel of the 3-D PSF. Then, the FWHM was measured on the fitted profile **(Supplementary Fig. 3)**. According to our measurements, a lateral FWHM of ~1 μm and an axial FWHM of ~3.5 μm of the PSF were maintained across the full measured 3D-FOV of Φ900 × 200 μm^3 **(Supplementary Fig. 3b-f)**. These values are larger than the theoretical FWHMs (0.37 μm lateral and 1.21 μm axial). This difference comes from the motion blur of continuous scanning and the lateral effective pixel size: (1) when imaging the PSF, the microscope's focal plane continuously translated across ~3 μm during the exposure of each frame, so that each measured pixel was blurred as an average of the intensity of the real PSF across a ~3-μm axial range, significantly increasing the 3-D size of the measured PSF. (2) The lateral effective pixel size (0.73 μm) in the images is larger than the real PSF's lateral (xy) size. Therefore, each pixel in the images acted as an averaging window applied to the real PSF, which smoothed the intensity peak of the real PSF and lowered the half maximum threshold to measure FWHMs, increasing the FWHM values. Nevertheless, considering the 0.73-μm lateral and 5-6 μm axial (z) sampling step size, our system maintains spatial resolution that is only bounded by effective pixel size and the z sampling step size across a Φ900 × 200 μm^3 3D-FOV. According to Nyquist's theorem, the 0.73 μm effective pixel size in our system corresponds to a lateral resolution of 1.46 μm, around one-fifth of the average diameter (6.62 μm[13]) of neuron somas in zebrafish.

3. Limited impact of gradual illumination intensity variation

The ~±20% gradual intensity variation along the x-axis in our Powell-lens-generated light sheet (**Extended Data Fig. 2**) is unlikely to pose challenges for imaging and extracting the voltage activity of neurons in the fish's brain. This is because: 1) the intensity variation was smooth and gradual across space, meaning that it does not corrupt the boundaries of individual neurons, and 2) the intensity variation stays stationary over time, ensuring that no temporal artifacts are introduced to the neural activity traces. If needed, this gradual intensity variation could be in principle corrected in the raw sample images by using reference images of a uniform fluorescent sample, such as a 3D fluorescent agarose sample.

4. Strategies to mitigate the effects of moving stripe artifacts

Of stripes in raw images, only the moving ones introduced rapidly-fluctuating temporal artifacts into fluorescence traces in affected brain regions. Other, static, stripes did not affect data quality,

any more than other static causes of variability (e.g., inevitable variability of protein expression across cells). Thus, our goal was to eliminate moving stripes, instead of all stripes. Through several control experiments, we verified that moving stripes were from shadows cast by blood cells flowing through vessels in the zebrafish brain (**Extended Data Fig. 3**; **Extended Data Fig. 4**) and associated text). We demonstrated three workarounds to remove or mitigate their effects. These include transient cardiac arrest, resonant scanning, and computational ROI exclusion. We clarified if and how the effect of moving stripes was mitigated for the data in each figure in **Supplementary Table 1**. Each method has its own advantages and limitations. Here we explain each method and clarify its trade-offs.

<u>4.1 Moving stripe removal through transient cardiac arrest</u>:

In past neuroscience studies of larval zebrafish, one strategy that has been used to remove moving stripes is to simply stop the heart. For example, in two recent studies performing voltage imaging in larval zebrafish[40,41], forceps were used to immobilize the heart mechanically for up to 30 mins, to remove moving stripes during voltage imaging. In one study[41], heart-stopped fish could respond to visual grating stimuli and fictively swim (as monitored using ventral nerve recording), in a well-established fictive behavior paradigm[14], performing similarly to fish with normal heartbeats[42,43]. Thus, heart-stopped fish maintained normal sensory and motor functions. In a second study[40], heart-stopped fish could not only respond to visual grating stimuli as normally as those with normal heartbeats, but also they could perform serotonin-mediated motor vigor learning as normal, for a period of 30 mins. In summary, heart-stopped fish maintained normal brain function, including complex functions such as learning. Perhaps this should not be too surprising: previous studies showed that larval zebrafish can survive in the total absence of blood flow until 7 days post fertilization, via oxygen diffusion into the body from outside, due to their small size[39,44,45].

Motivated by these prior results, here we chose to explore pharmacological cardiac arrest as a simple and reversible approach to suppress moving stripe artifacts in the context of whole-brain voltage imaging (**Extended Data Fig. 3b**). To this end, a 45 mM solution of 2,3-butanedione monoxime (BDM; Sigma) was prepared by dissolving BDM powder in fish facility water[46–48]. For treatment, the water in the imaging chamber containing a mounted larval zebrafish was removed with a pipette and immediately replaced with the BDM solution. After 3 min of incubation, the heartbeat was completely arrested, and the brain was imaged again using the light-sheet microscope (**Extended Data Fig. 3b**, post-BDM). For recovery, the BDM solution was removed and replaced with fresh fish facility water using the same procedure; this wash step was performed twice to minimize residual BDM. Circulation resumed within minutes after washout (**Extended Data Fig. 3b**, post-recovery). The fish were then left in the imaging chamber in the dark for approximately 20 min before re-imaging. Following BDM treatment, moving stripe artifacts disappeared and reappeared after recovery (**Extended Data Fig. 3b; Supplementary Video 2**), demonstrating both the effectiveness and reversibility of BDM-induced cardiac arrest for eliminating moving stripe artifacts.

Given that forceps-stopping has been shown to be compatible with a range of behaviors, it may be preferred to BDM, which like any drug, could have off-target effects. In the current study, we simply wanted to show that stopping the heart, which is an accepted practice in larval zebrafish neuroscience, would work in the context of our voltage microscopy. Going forward, it is possible

that an elegant way of stopping the heart would be optogenetics[49], using an opsin to inactivate cardiac contraction with light, so that the heart could be stopped precisely during the period of imaging, or perhaps slowed down in beat frequency so that moving stripes would occur with frequencies slow enough to be cleanly distinguished from neural spikes.

In summary, given the larval zebrafish's good tolerance for blood-flow interruption, and empirical studies showing that the sensory, motor, and learning functions of the fish brain remained intact during such short-term heart stoppage (<30 minutes), transient cardiac arrest may be applicable for a wide range of biological studies.

4.2 Mitigating effects of moving stripes using resonant scanning

We demonstrated an optical approach to mitigate both the stationary and moving stripe artifacts (**Extended Data Fig. 5, Extended Data Fig. 1f**). This approach is conceptually related to multidirectional illumination strategies used in earlier light-sheet microscopy studies[50–53]. In our implementation, we used a resonant mirror scanner to rapidly pivot the light-sheet in the image plane during each camera exposure. By pivoting the light sheet across a sufficiently large angular range within a single frame, stripe artifacts were spatially dispersed and effectively averaged out over broader regions, thereby being suppressed.

To achieve the required pivoting speed, we employed an 8-kHz resonant mirror scanner (RESSCAN, Sutter Instruments), which has a scan period of 127 μs. We set the camera exposure time to match the resonant scanner's scan period (127 μs), ensuring that the light sheet completed a full pivoting cycle during each frame exposure regardless of the exposure start time. To prevent temporal artifacts arising from frequency aliasing between volumetric light-sheet z-scanning and in-plane light-sheet pivoting, the start of each volumetric z-scan was synchronized with the start of a resonant pivoting cycle.

In our experiment, when the resonant scanner was running, both the static and the moving stripe artifacts were suppressed in the zebrafish brain (**Extended Data Fig. 5a,b,d,e, h,i,k,l**). Importantly, neuron activity spikes from ROIs that were previously obscured by the temporal fluctuations caused by moving stripes became detectable (**Extended Data Fig. 5c,f,j,m,** red arrows). We note that for traces 1-10 in these figures, we intentionally selected ROIs that were affected by moving stripes to show the effectiveness of resonant scanning in suppressing these artifacts.

To quantitatively assess stripe suppression performance, we measured the temporal standard deviations (TSDs, see **Supplementary Note 5**) of the traces from moving-stripe affected neurons, using TSD as a metric for artifact strength. Activation of the resonant scanner reduced the TSDs of these traces by an average of 42%, and more than 90% of the affected ROIs exhibited reduced TSDs (**Extended Data Fig. 5g,n**).

Despite its effectiveness, resonant scanning introduced several important trade-offs, primarily due to the extremely high imaging frame rates used in our voltage imaging experiments—over 6000 frames per second, tens of times faster than in prior studies[50–53]. In experiments without resonant scanning, we imaged 30 planes across a ~170-μm axial range at 200.8 Hz, using a camera frame rate of 6025 Hz and an exposure time of 40 μs, corresponding to a ~24% duty cycle. During each 40-μs exposure, the focal plane shifted by ~2.7 μm along the z-axis.

In contrast, resonant scanning required matching the exposure time to the resonant scan period (127 μs) to ensure completion of a full pivoting cycle within each frame. To limit axial motion blur under these conditions, we reduced the axial scan range to 120 μm and imaged 20 planes at 203 Hz. This represented an approximately one-third reduction in axial field-of-view compared with imaging without resonant scanning. Under this configuration, the camera operated at 4060 frames per second with a 52% duty cycle, and the focal plane shifted by ~6.1 μm along the z-axis during each 127-μs exposure.

In principle, the number of imaged planes could scale linearly with resonant frequency. For example, a 12-kHz resonant scanner would support imaging 20×(12 kHz / 8 kHz) ≈ 30 planes at 200.8 Hz, albeit with increased axial motion blur compared with imaging without resonant scanning (~6.1 μm z-shift vs. ~2.7 μm z-shift during an exposure). Achieving both the original axial field-of-view (~170 μm) and comparable axial motion blur (~2.7 μm per exposure) would require a resonant scanning rate of approximately 27.1 kHz (8 kHz × 3/2 × 6.1/2.7). To our knowledge, no commercially available resonant scanner can achieve this scanning rate while providing the required optical aperture (~5 mm in our system).

A second limitation of resonant scanning arises from the spatial averaging mechanism itself. By spreading illumination over a broader area, resonant scanning occasionally introduced moving stripe artifacts into regions that were previously unaffected, creating new artifacts where none were before, in approximately 1% of all segmented ROIs (**Extended Data Fig. 5c,f,j,m**, trace 11).

Thus, while resonant scanning has promise, and in the future could be of use, it comes with caveats. As a result, we recorded most voltage datasets (see **Supplementary Table 1**) in this paper without resonant scanning, and addressed the moving stripes later using a computational method described below.

4.3 Computational detection and removal of ROIs affect by moving stripe artifacts

Because each moving stripe modulates the fluorescent baselines of ROIs on the stripe in a synchronous fashion, it is possible to separate the affected ROIs from unaffected ROIs based on their spatial distributions and the temporal patterns of their traces. When analyzing raw light-sheet datasets, we employed a computational approach to detect the ROIs affected by moving stripes in our whole-brain datasets and then exclude these ROIs from analysis. Pulse-like artifacts could, in principle, be mistakenly identified as spikes by the automatic spike detection process, thus any ROIs containing even a single pulse-like artifact should be excluded from further analysis (future analyses could, in theory, enable pulse-like artifacts to be subtracted away, sparing these ROIs for downstream analysis). To identify them, we looked for ROIs with highly correlated pulse-like or fast-fluctuating activities distributed along the fish's medio-lateral axis (**Supplementary Fig. 4b,c,** see **Methods**). To facilitate the identification of contaminated ROIs, we clustered ROIs with similar temporal traces. We first applied a 250-ms moving Hanning window to each ROI's spike raster for smoothing and estimating its firing rate. Subsequently, we used the Uniform Manifold Approximation and Projection[54] (UMAP) algorithm to map the smoothed raster of each ROI onto a 2-dimensional manifold (**Supplementary Fig. 14a**). Following this mapping, we utilized the Density-Based Spatial Clustering of Applications with Noise[55] (DBSCAN) algorithm to separate the ROIs into clusters.

Each cluster on the 2-dimensional UMAP manifold contained ROIs with similar spiking activities (**Supplementary Fig. 5; Supplementary Fig. 14a**). Next, we visually inspected each cluster's ROIs' temporal traces and their spatial distributions within the fish (**Supplementary Fig. 5**). Our criteria to identify an artifact cluster were: 1) the temporal traces of the cluster's ROIs exhibited synchronous pulse-like or fast-fluctuating patterns (**Supplementary Fig. 5,** orange arrows); 2) the spatial distribution of the cluster's ROIs had the same spatial features of the artifact stripes or shadows, i.e., the ROIs were concentrated in a region (or regions) that was narrow in the x and z axes (< 100 μm) and spanned from a certain location (possibly in the middle of the brain, as there are blood vessels) to the edge of the brain opposite to the edge of light sheet incidence, along the y axis (light sheet illumination axis) (**Supplementary Fig. 5**, green arrows). A cluster was identified as an artifact cluster only when it satisfied both these criteria. To better illustrate these criteria, three artifact-contaminated clusters and three non-artifact clusters, both randomly selected from Fish B, are presented in **Supplementary Fig. 5**. Once we determined a cluster was contaminated by artifact, we removed all the corresponding ROIs, and used the remaining ROIs to repeat the UMAP-DBSCAN process until all the clusters with artifact contamination were removed (see **Methods**). In the future, specific algorithms might be developed to identify and decompose these highly correlated pulse-like artifacts as independent components, easily separated from the neural activity, so that artifact-contaminated ROIs can also be used.

These different methods to address moving stripes let researchers choose the balance of stripe pattern suppression, spatiotemporal coverage, and experimental duration that best fit each study. For acute (<30 minute) experiments one might opt for transient cardiac arrest. To image a 3D-FOV with small axial range (z-axis range, e.g., <120 μm), one might choose addition of a resonant scanner to suppress stripes. Our goal was to demonstrate voltage imaging at large 3D-FOV using the simplest possible optical and experimental setup. Therefore, we opted for our computational method to detect and eliminate affected ROIs. In our experiments, affected ROIs only accounted for ~25% of all ROIs (averaged in Fish A-D), distributed across the whole brain. Our analyses were based on clean traces from unaffected ROIs, which were the majority (n=12935, 15135, 19039, 15998 neurons, or yields of 73%, 59%, 75%, and 92% of total ROIs for Fish A-D, respectively), distributed throughout the whole brain. These putative neural traces led to consistent findings and results on sequential neural activity across trials and animals (**Figs. 3-5, Extended Data Figs. 9,10**). For the dataset in each of the figures, we indicated if and how the moving stripe artifacts were addressed in **Supplementary Table 1**.

5. Normalized temporal standard deviation map (NTSDM)

To better visualize the moving stripes' distributions in of the brain, we generated a normalized temporal standard deviation map (NTSDM) of each time-series recording. To obtain this map, we first corrected for photobleaching by normalizing each frame's intensity using the mean intensity of the frame. Then we calculated the standard deviation of each pixel's intensity over time, and normalized this standard deviation map by dividing it by the square root of each pixel's temporal mean intensity, which corresponds to the theoretical shot noise. The rationale behind this calculation is:

The temporal standard deviation (TSD) of a pixel's intensity trace is contributed to by shot noise, real neural activity, moving-stripe induced temporal artifacts, and other noise (e.g., camera noise) whose contribution assumingly remains unchanged during an imaging trial. In our imaging, each pixel within a neuron ROI on average detected ~250 photons per frame, providing a shot noise more than two times of the camera noise (~7e-). Assuming there was no neural activity or moving stripe, each pixel's noise could be approximated using its shot noise. We calculated each pixel's noise using its intensity trace's temporal standard deviation. Therefore, if there were no neural activity or moving stripe, by normalizing the recording's standard deviation map using its shot noise, we would obtain a near solid color map that does not highlight anything. In real imaging experiments, both neural activity and moving stripes cause fluctuations in a pixel's intensity trace, which increases the standard deviation of the trace and the value in the normalized temporal standard deviation map. As the intensity fluctuation caused by moving stripes usually has much higher amplitude compared to the fluorescent changes caused by real neural voltage activity (otherwise the moving stripe will not be a big issue), these moving stripes will stand out in the normalized temporal standard deviation map (NTSDM) as bright stripes along the direction of light-sheet illumination.

We found this NTSDM in general worked well in highlighting the moving stripes in time-series recordings. But this method also has some limitations. It neglects low-intensity pixels where shot noise is not dominant. It also highlights areas that exhibit a lot of neural activity. Its photobleaching correction algorithm might also be improved. Probably due to these limitations, the normalized standard deviation maps of the top and bottom brain layers showed higher background resembling the shapes of brain sections in the raw images (**Extended Data Fig. 3b,** top and bottom rows**)**. Despite these limitations, NTSDMs were sufficient for visualizing the moving stripe locations and comparing moving stripes in the same layer over time.

6. Observations related to the blood-flow hypothesis for moving stripe artifacts

(1) We observed the moving stripes originating at different offsets from the left side of the brain (the side where the light sheet entered the brain) (**Extended Data Fig. 4,** magenta arrows). Besides the stripes originating from the left side, some moving stripes originated within the brain.

(2) Blood cells can not only scatter light, but also refract light like microlenses [38]. With this lens-like property, blood cells in the fish brain can focus or diverge parallel illumination light to produce both bright and dark stripe patterns.

(3) We did not perform a consistency analysis of the moving stripe distribution across different fish brains, as variations affecting the moving stripe distribution between imaging sessions for individual fish were difficult to control. However, we did see some patterns about where, at the brain region level, the moving stripes more likely occurred in the zebrafish brain: the lower brain layers typically tended to have more moving stripes (e.g., **Extended Data Fig. 3b**, z = -170 μm) and almost every fish had moving stripes in the optic tectum region near the middle brain layers (e.g., **Extended Data Fig. 3b,** z = -82 μm). That being said, in our experiments we encountered 3-4 fish (out of dozens of fish that we imaged) that had significantly fewer (approximately 80-90% less) moving stripe artifacts than other fish. We don't know the exact reason for this, but these fish exhibited good neural activity and we didn't observe any abnormality both visually and when

analyzing their data. We speculate they could have less blood flow (zebrafish can live until 7 dpf without blood flow [39]), or their blood cells might be less scattering or have refractive index closer to their surrounding environment. The above are just our observations, and we did not carefully study these observations, as characterizing the properties of fish lines is not the main focus of our paper. Here we just mention them to note that while there might be some consistency of the moving stripe distributions across fish, these artifacts can also vary a lot from fish to fish in terms of their exact locations and quantity.

7. Limitations of ratiometric methods for correcting moving-stripe-induced temporal artifacts

One might consider using ratiometric methods (e.g., comparing the voltage signal to a reference fluorophore) to compensate for artifacts, such as stripe artifacts. We did not implement such ratiometric methods, because the ratiometric approaches may not be able to efficiently correct the GEVI baseline intensity fluctuation caused by the moving artifacts in our case. This is because the intensity of the reference fluorophore may not scale proportionally to the fluctuations of the GEVI intensity, due to fluorescence saturation of either fluorophore at high-intensity excitation. We estimate that in our experiments, the GEVI reached approximately 40% of its maximum fluorescence intensity. At this level, the GEVI's baseline intensity does not change proportionally to the excitation intensity. Although ratiometric approaches could be a backup plan in the case where the stripe artifacts cannot be addressed using other approaches, we would like to note that the ratiometric approach is not an ideal solution to correct moving artifacts, for several additional reasons: (1) First, crosstalk of the reference fluorescence and the GEVI fluorescence could occur due to emission spectrum overlap. It is difficult to find a reference fluorophore that can be efficiently excited with the same excitation light as for the GEVI fluorophore, while having a non-overlapping emission spectrum. Alternatively, the overlapped part of the spectrum could be filtered out using emission filters, leading to undesired light loss. (2) Second, dual channel imaging requires doubling the number of cameras, increasing the cost, the complexity of the hardware, and the amount of data to analyze. (3) Third, the reference fluorophore will occupy a precious color channel that could otherwise be used for other applications, such as optogenetics or fluorescence indicators of other neural signals (e.g., neurotransmitters).

8. Voltage indicator testing and expression

We tested and compared five different voltage indicators in larval zebrafish. The five indicators were ASAP3-Kv[28], Voltron2-Kv[29], Ace-mNeon2-Kv[30], pAce-Kv[30], and Positron2-Kv[31]. We first transiently expressed the five indicators in larval zebrafish using embryonic micro-injection[32]. We imaged their spontaneous activity and compared their SNRs per action potential (SNR_{AP}), which were calculated as the ratio between the fluorescence intensity change per action potential and the shot noise at the spike peak per neuron per frame ($SNR_{AP} = \left(N_{peak} - N_0\right) / \sqrt{N_{peak}}$, N_{peak} the detected photon number at the spike peak per neuron per frame, N_0 the detected photon number at the resting potential right before the measured spike per neuron per frame; these peaks and resting potentials were estimated by eye). We observed that, compared to ASAP3-Kv,

Positron2-Kv and Voltron2-Kv exhibited ~10× brightness, while Ace-mNeon2-Kv and pAce-Kv showed ~5× brightness. This brightness difference might result from a difference in molecular brightness, or a difference in expression density on zebrafish neural membranes. We found that Voltron2-Kv and Positron2-Kv exhibited the highest SNR_{AP} among all tested indicators, in the zebrafish that we made, ~1.4× of those of Ace-mNeon2-Kv and pAce-Kv, and ~2.4× of that of ASAP3-Kv. We then constructed transgenic zebrafish lines with pan-neuronal expression of Voltron2-Kv and Positron2-Kv using the Tol2 transposon system[32]. We used the Gal4/UAS system[33] to express Voltron2-Kv and Positron2-Kv, with the Gal4 gene driven by the pan-neuronal promoter HuC[34]. For each of the Positron2-Kv and Voltron2-Kv indicators, we made one fish line. We didn't observe much voltage activity in the Voltron2-Kv fish line we made, which could have been due to any number of factors, many of which have nothing to do with Voltron2 itself; given that our goal was simply to probe whether our microscope was capable of imaging voltage in neurons distributed across an entire zebrafish brain, we used the Positron2-Kv fish line in our experiments, as a somewhat arbitrary choice.

We chose to use the Gal4/UAS expression system due to its high expression strength[35,36], but this might also make our transgenic Positron2-Kv fish susceptible to this silencing effect. As a comparison to the Gal4/UAS promoter, we tried directly expressing Positron2-Kv using a HuC promoter. We found that directly expressing Positron2-Kv using the HuC promoter, compared to using HuC:Gal4 and tandem UAS, did result in more pan-neuronal expression, but this also decreased fluorescent brightness (**Supplementary Fig. 6**, showing a comparison of confocal images of a 6 day-post-fertilization (dpf) F2 generation Tg(HuC:Gal4; 2xUAS:Positron2-Kv) fish and a 5-dpf F1 generation Tg(HuC:Positron2-Kv) fish; confocal imaging does not generate stripe artifacts as it excites samples using high-NA light cones instead of parallel light sheet), potentially making brain-wide voltage imaging more difficult. The images of the two fish were acquired using the same microscope settings. Compared with the Tg(HuC:Positron2-Kv) fish, the Tg(HuC:Gal4; 2xUAS:Positron2-Kv) fish had higher brightness (~1.1× averaged over the whole brain, ~1.7× averaged over the whole optic tectum (OT), and ~2.6× averaged over the whole habenula (HB)). The Tg(HuC:Gal4; 2xUAS:Positron2-Kv) fish brain also showed more varied levels of expression across different brain regions. Please note, these were not systematic explorations: we did not control for precise fish age or generation, and examined only one fish for each type. We did not image the Tg(HuC:Positron2-Kv) fish under our light-sheet microscope, either. In any case, fish transgenic line generation is an ongoing area of innovation for the entire fish community, and as that field continues improving, so will the performance of voltage imaging.

9. Baseline noise quantification

Many factors, in addition to shot noise, can influence the stability of the fluorescence baseline of neurons when monitoring voltage activity. Some of these factors can include the readout noise (~7e- in our case) of camera pixels, limitations in the repeatability of galvo and piezo scanning, small vibrations of the setup and the sample, and fluctuations in the excitation laser power. To evaluate the extent to which these factors impact the fluorescence baseline stability in our experiments, we quantified the baseline noise of neurons that visually did not overlap with any moving stripe artifact by imaging the brain of a larval zebrafish pan-neuronally labeled with a cytosolic fluorescent marker Kaede (Tg(HuC:Gal4; UAS:Kaede)) at 200 Hz (**Supplementary Fig.**

7). We chose to use Kaede because we happened to have that fish line, and because the excitation and emission spectra of Kaede matched our current microscope. Kaede fish imaging was performed using the same protocols as during imaging whole brains of Positron2-Kv fish, except that we adjusted the excitation power to match the raw image intensity to that in our voltage imaging experiments. We extracted the average intensity traces of 72 (an arbitrary number) neuron ROIs distributed across a randomly selected brain plane (out of 30). To obtain these ROIs, an experimenter randomly searched around the areas in the brain plane that did not visually overlap with any moving stripe in the raw video and manually annotated them. After correcting for photobleaching by applying a high-pass filter to the raw traces (cut-off frequency: 15 Hz), we calculated the standard deviations of the traces to quantify the measured noise. Moreover, as a reference, we calculated the theoretical shot noise for each trace. To do that, we first calculated the mean intensity of each trace and converted the value to photon number by dividing it by the camera's conversion factor (0.13 in our case). The theoretical shot noise of each trace was calculated as the square root of the photon number. The measured noise of these neurons (quantified by the standard deviation of the traces) was on average 1.58±0.45 (mean ± standard deviation, n=72 neurons from 1 fish) times their theoretical shot noise (**Supplementary Fig. 7b**). The measured noise was on average 0.83±0.28 % (mean ± standard deviation, n=72 neurons from 1 fish) of the baseline intensity (**Supplementary Fig. 7b,c**).

10. Simulation of ASAP5-Kv neural traces at different imaging rates

For simulation of ASAP5-Kv neural traces at different imaging rates, we used the spikes detected in the 1000-Hz Positron2-Kv fish imaging data as the ground truth. To simulate ground truth voltage traces, we convolved these spikes with an action potential curve generated using the Hodgkin–Huxley model. Each action potential curve has a duration of 0.5 ms (full width at half maximum). We chose this short 0.5 ms duration to represent faster action potentials, as action potentials with longer durations generate wider fluorescent spikes and are easier to detect. To model the fluorescent traces in response to these voltage traces, we used the kinetic parameters of ASAP5-Kv at room temperature according to [37]. These parameters include the fast depolarization time constant $\tau_{D,fast} = 2.6\,ms$, the slow depolarization time constant $\tau_{D,slow} = 45.3\,ms$, the fast repolarization time constant $\tau_{R,fast} = 4.3\,ms$, and the slow repolarization time constant $\tau_{R,slow} = 55.9\,ms$, along with their respective proportions in the biphasic depolarization and repolarization processes. We calculated the fluorescent traces $F(t)$ using the following formula with a time step of 0.1 ms.

$$F(t) = \int_0^t \frac{dV(\tau)}{d\tau} \cdot R(t-\tau)d\tau \tag{1}$$

$$R(t) = \begin{cases} 1 - a_1 \exp\left(-\frac{t}{\tau_{D,fast}}\right) - (1-a_1)\exp\left(-\frac{t}{\tau_{D,slow}}\right), if \frac{dV}{dt} \geq 0 \\ a_2 \exp\left(-\frac{t}{\tau_{R,fast}}\right) + (1-a_2)\exp\left(-\frac{t}{\tau_{R,slow}}\right) - 1, if \frac{dV}{dt} < 0 \end{cases} \tag{2}$$

Here $V(t)$ is the simulated voltage traces, t is time, and a_1 (0.92) and a_2 (0.75) denote the proportions of the fast exponential components in the depolarization and the repolarization

processes, respectively. We assumed ASAP5-Kv's fluorescence to be linearly related to the neural voltage and set the scaling factor to 1. The 1000-Hz and 200-Hz traces were generated by sampling discrete points in the high temporal resolution (0.1 ms) traces at 1-ms and 5-ms intervals, respectively. Prior to spike detection, we added Gaussian noise to the 1000-Hz and 200-Hz traces, with the noise's standard deviation equal to 1/10 of the maximum spiking amplitude of the traces.

11. Putative UV-induced photoswitching of the Positron2 GEVI

Upon examining the raw ROI traces, in addition to photobleaching (**Supplementary Fig. 15a,** black arrows**)**, we noticed a decay in the overall fluorescence intensity of all ROIs as the 405 nm LED turned on (**Supplementary Fig. 15,** orange curve and red curves, after computational removal of moving stripe affected ROIs). After the LED was turned off, the decreased fluorescence recovered. We hypothesized that this decay and recovery in fluorescence intensity might result from reversible photoswitching of the Positron2 indicator when being exposed to 405-nm light. Previous studies[56] showed that shining 488-nm blue light could reversibly increase the fluorescence intensity of paQuasAr3, an opsin-based voltage indicator. Positron2 is based on the opsin-FRET design, which means the observed fluorescence from Positron2 is the fluorescence emitted by the fluorophore donor minus the fluorescence absorbed by the opsin acceptor. Therefore, assuming that 405-nm light could reversibly increase the absorbance efficiency of the opsin acceptor of Positron2, there would be a decay of the fluorescence at the onset of the light stimulus and a recovery of the fluorescence after the light stimulus was turned off, as we observed in our experiments. Further biophysical studies of voltage indicators in such contexts may be helpful in the future, but here we simply note that the phenomenon was reversible and easily isolated from the true signal. Since this LED-induced fluorescence intensity change affected the entire fish, its impact on neuronal temporal traces could be effectively mitigated during the background removal step of VolPy (**Supplementary Fig. 15**). Despite the LED-induced intensity change, the rise of activity at the onset of LED stimulation was visible in Group 1 neurons' raw traces and the VolPy-extracted traces (**Supplementary Fig. 15a,b,** orange arrows). In contrast, non-Group 1 neurons did not exhibit this sudden increase of activity following the onset of the light stimulus (**Supplementary Fig. 15a,b,** green arrows).

12. Design innovations of the remote-scanning light-sheet microscope

While at the conceptual level, our microscope follows the widespread idea of light-sheet microscopy with remote refocusing, there were several necessary innovations in the design and implementation of the microscope, that brought its capabilities to the level required for voltage imaging of neurons distributed across almost entire brains. First, we have designed a new remote scanning module, and the software and pipeline for calibration and control. We combined a large shift, high resonant frequency piezo bender scanner with a lightweight mirror for remote scanning, to enable 200-µm axial scanning at up to 300 Hz (or >100 µm at 500 Hz). We also developed the hardware, software, and pipeline (see "Implementation of the microscope design" section) to allow accurately calibrating and controlling the remote mirror movement. This is a nontrivial design, because accurately calibrating and controlling the movement of a piezo bender scanner—which operates at hundreds of hertz in open-loop mode, and exhibited hysteresis—was challenging. To

achieve the desired waveform, we had to measure the piezo bender's movement at a rate of tens of kilohertz, and at sub-micrometer precision, and conduct iterative fine adjustments of the driving signals that contained multiple unconstrained parameters. Second, remote refocusing enhances the microscope's optical scanning rate, but exacerbates the mismatch between the microscope's optical scanning speed, and the camera's acquisition speed. To alleviate this mismatch and improve imaging speed, we combined distributed planar imaging[57] with remote-refocusing light-sheet microscopy, efficiently doubling the volume rate for voltage imaging. This strategy could in principle be further scaled up, by chaining multiple distributed planar imaging modules and splitting the image to more cameras, to achieve even higher volume rates. Third, we are the first (to the best of our knowledge) to use sensors designed to combine high frame rates with large camera pixel numbers, for high-speed microscopy imaging. Indeed, we had to build a custom camera using this sensor, for our initial microscope implementation, and to assess its performance. Our custom camera's firmware and software integrate open-source architectures widely used in the neuroscience community (Firmware: Open-Ephys ONIX; Software: Bonsai), and could significantly accelerate future high-speed camera development, reducing the time required to adapt novel sensor chips into microscope architectures. Since our initial work, commercial camera manufacturers have now introduced cameras based on this sensor, easing its adoption by other labs.

References

1. Levoy, M., Ng, R., Adams, A., Footer, M. & Horowitz, M. Light field microscopy. in *ACM SIGGRAPH 2006 Papers on - SIGGRAPH '06* (ACM Press, New York, New York, USA, 2006). doi:10.1145/1179352.1141976.
2. Prevedel, R. *et al.* Simultaneous whole-animal 3D imaging of neuronal activity using light-field microscopy. *Nat. Methods* **11**, 727–730 (2014).
3. Bai, L. *et al.* Volumetric voltage imaging of neuronal populations in the mouse brain by confocal light-field microscopy. *Nat. Methods* **21**, 2160–2170 (2024).
4. Kunst, M. *et al.* A cellular-resolution atlas of the larval zebrafish brain. *Neuron* **103**, 21-38.e5 (2019).
5. Cong, L. *et al.* Rapid whole brain imaging of neural activity in freely behaving larval zebrafish (). *Elife* **6**, (2017).
6. Yoon, Y.-G. *et al.* Sparse decomposition light-field microscopy for high speed imaging of neuronal activity. *Optica* **7**, 1457 (2020).
7. Zhang, Z. *et al.* Imaging volumetric dynamics at high speed in mouse and zebrafish brain with confocal light field microscopy. *Nat. Biotechnol.* **39**, 74–83 (2021).
8. Wu, J. *et al.* Iterative tomography with digital adaptive optics permits hour-long intravital observation of 3D subcellular dynamics at millisecond scale. *Cell* **184**, 3318-3332.e17 (2021).

9. Hillman, E. M. C., Voleti, V., Li, W. & Yu, H. Light-Sheet Microscopy in Neuroscience. *Annu. Rev. Neurosci.* **42**, 295–313 (2019).

10. Stelzer, E. H. K. *et al.* Light sheet fluorescence microscopy. *Nature Reviews Methods Primers* **1**, 1–25 (2021).

11. Corsetti, S., Gunn-Moore, F. & Dholakia, K. Light sheet fluorescence microscopy for neuroscience. *J. Neurosci. Methods* **319**, 16–27 (2019).

12. Power, R. M. & Huisken, J. A guide to light-sheet fluorescence microscopy for multiscale imaging. *Nat. Methods* **14**, 360–373 (2017).

13. Ahrens, M. B., Orger, M. B., Robson, D. N., Li, J. M. & Keller, P. J. Whole-brain functional imaging at cellular resolution using light-sheet microscopy. *Nat. Methods* **10**, 413–420 (2013).

14. Vladimirov, N. *et al.* Light-sheet functional imaging in fictively behaving zebrafish. *Nat. Methods* **11**, 883–884 (2014).

15. Botcherby, E. J., Juskaitis, R., Booth, M. J. & Wilson, T. Aberration-free optical refocusing in high numerical aperture microscopy. *Opt. Lett.* **32**, 2007–2009 (2007).

16. Botcherby, E. J., Juškaitis, R., Booth, M. J. & Wilson, T. An optical technique for remote focusing in microscopy. *Opt. Commun.* **281**, 880–887 (2008).

17. Fahrbach, F. O., Voigt, F. F., Schmid, B., Helmchen, F. & Huisken, J. Rapid 3D light-sheet microscopy with a tunable lens. *Opt. Express* **21**, 21010–21026 (2013).

18. Creators Alfred Millett-Sikking1 Nathaniel H. Thayer2 Adam Bohnert2 Andrew G. York2 Show affiliations 1. @Calico 2. @calico. *Calico/Remote_refocus: Pre-Print*. doi:10.5281/zenodo.1146084.

19. Sparks, H. *et al.* Development a flexible light-sheet fluorescence microscope for high-speed 3D imaging of calcium dynamics and 3D imaging of cellular microstructure. *J. Biophotonics* **13**, e201960239 (2020).

20. Dibaji, H. *et al.* Axial de-scanning using remote focusing in the detection arm of light-sheet microscopy. *Res Sq* (2023) doi:10.21203/rs.3.rs-3338831/v1.

21. Voleti, V. *et al.* Real-time volumetric microscopy of in vivo dynamics and large-scale samples with SCAPE 2.0. *Nat. Methods* **16**, 1054–1062 (2019).

22. Bouchard, M. B. *et al.* Swept confocally-aligned planar excitation (SCAPE) microscopy for high speed volumetric imaging of behaving organisms. *Nat. Photonics* **9**, 113–119 (2015).

23. Yang, B. *et al.* Epi-illumination SPIM for volumetric imaging with high spatial-temporal resolution. *Nat. Methods* **16**, 501–504 (2019).

24. Yang, B. *et al.* DaXi-high-resolution, large imaging volume and multi-view single-objective light-sheet microscopy. *Nat. Methods* **19**, 461–469 (2022).

25. Sapoznik, E. *et al.* A versatile oblique plane microscope for large-scale and high-resolution imaging of subcellular dynamics. *Elife* **9**, (2020).

26. Hoffmann, M. & Judkewitz, B. Diffractive oblique plane microscopy. *Optica* **6**, 1166 (2019).

27. Hoffmann, M., Henninger, J., Veith, J., Richter, L. & Judkewitz, B. Blazed oblique plane microscopy reveals scale-invariant inference of brain-wide population activity. *Nat. Commun.* **14**, 8019 (2023).

28. Villette, V. *et al.* Ultrafast Two-Photon Imaging of a High-Gain Voltage Indicator in Awake Behaving Mice. *Cell* **179**, 1590-1608.e23 (2019).

29. Abdelfattah, A. S. *et al.* Sensitivity optimization of a rhodopsin-based fluorescent voltage indicator. *Neuron* **111**, 1547-1563.e9 (2023).

30. Kannan, M. *et al.* Dual-polarity voltage imaging of the concurrent dynamics of multiple neuron types. *Science* **378**, eabm8797 (2022).

31. Voltron and positron. *Janelia Research Campus* https://www.janelia.org/open-science/voltron-and-positron.

32. Kawakami, K. Transposon tools and methods in zebrafish. *Dev. Dyn.* **234**, 244–254 (2005).

33. Halpern, M. E. *et al.* Gal4/UAS transgenic tools and their application to zebrafish. *Zebrafish* **5**, 97–110 (2008).

34. Park, H. C. *et al.* Analysis of upstream elements in the HuC promoter leads to the establishment of transgenic zebrafish with fluorescent neurons. *Dev. Biol.* **227**, 279–293 (2000).

35. Scheer, N. & Campos-Ortega, J. A. Use of the Gal4-UAS technique for targeted gene expression in the zebrafish. *Mech. Dev.* **80**, 153–158 (1999).

36. Asakawa, K. *et al.* Genetic dissection of neural circuits by Tol2 transposon-mediated Gal4 gene and enhancer trapping in zebrafish. *Proc. Natl. Acad. Sci. U. S. A.* **105**, 1255–1260 (2008).

37. Hao, Y. A. *et al.* A fast and responsive voltage indicator with enhanced sensitivity for unitary synaptic events. *Neuron* **112**, 3680-3696.e8 (2024).

38. Ricci, P. *et al.* Removing striping artifacts in light-sheet fluorescence microscopy: a review. *Prog. Biophys. Mol. Biol.* **168**, 52–65 (2022).

39. Kugler, E. *et al.* The effect of absent blood flow on the zebrafish cerebral and trunk vasculature. *Vasc. Biol.* **3**, 1–16 (2021).

40. Kawashima, T. *et al.* Voltage imaging reveals circuit computations in the raphe underlying serotonin-mediated motor vigor learning. *Neuron* (2025) doi:10.1016/j.neuron.2025.05.017.

41. Abdelfattah, A. S. *et al.* Bright and photostable chemigenetic indicators for extended in vivo voltage imaging. *Science* **365**, 699–704 (2019).

42. Mu, Y. *et al.* Glia accumulate evidence that actions are futile and suppress unsuccessful behavior. *Cell* **178**, 27-43.e19 (2019).

43. Duque, M. *et al.* Ketamine induces plasticity in a norepinephrine-astroglial circuit to promote behavioral perseverance. *Neuron* **113**, 426-443.e5 (2025).

44. Jin, S.-W. *et al.* A transgene-assisted genetic screen identifies essential regulators of vascular development in vertebrate embryos. *Dev. Biol.* **307**, 29–42 (2007).

45. González-Rosa, J. M. Zebrafish models of cardiac disease: From fortuitous mutants to precision medicine. *Circ. Res.* **130**, 1803–1826 (2022).

46. Dietrich, A.-C., Lombardo, V. A., Veerkamp, J., Priller, F. & Abdelilah-Seyfried, S. Blood flow and bmp signaling control endocardial chamber morphogenesis. *Dev. Cell* **32**, 387 (2015).

47. Banjo, T. *et al.* Haemodynamically dependent valvulogenesis of zebrafish heart is mediated by flow-dependent expression of miR-21. *Nat. Commun.* **4**, 1978 (2013).

48. Foo, Y. Y. *et al.* Effects of extended pharmacological disruption of zebrafish embryonic heart biomechanical environment on cardiac function, morphology, and gene expression. *Dev. Dyn.* **250**, 1759–1777 (2021).

49. Arrenberg, A. B., Stainier, D. Y. R., Baier, H. & Huisken, J. Optogenetic control of cardiac function. *Science* **330**, 971–974 (2010).

50. Huisken, J. & Stainier, D. Y. R. Even fluorescence excitation by multidirectional selective plane illumination microscopy (mSPIM). *Opt. Lett.* **32**, 2608–2610 (2007).

51. Sancataldo, G. *et al.* Flexible multi-beam light-sheet fluorescence microscope for live imaging without striping artifacts. *Front. Neuroanat.* **13**, 7 (2019).

52. Glaser, A. K. *et al.* Multidirectional digital scanned light-sheet microscopy enables uniform fluorescence excitation and contrast-enhanced imaging. *Sci. Rep.* **8**, 13878 (2018).

53. Ricci, P. *et al.* Fast multi-directional DSLM for confocal detection without striping artifacts. *Biomed. Opt. Express* **11**, 3111–3124 (2020).

54. McInnes, L., Healy, J. & Melville, J. UMAP: Uniform Manifold Approximation and Projection for Dimension Reduction. *arXiv [stat.ML]* (2018).

55. Ester, M., Kriegel, H.-P., Sander, J. & Xu, X. A density-based algorithm for discovering clusters in large spatial databases with noise. in *Proceedings of the Second International Conference on Knowledge Discovery and Data Mining* 226–231 (AAAI Press, 1996).

56. Chien, M.-P. *et al.* Photoactivated voltage imaging in tissue with an archaerhodopsin-derived reporter. *Sci. Adv.* **7**, (2021).

57. Greer, C. J. & Holy, T. E. Fast objective coupled planar illumination microscopy. *Nat. Commun.* **10**, 4483 (2019).

Supplementary Figures

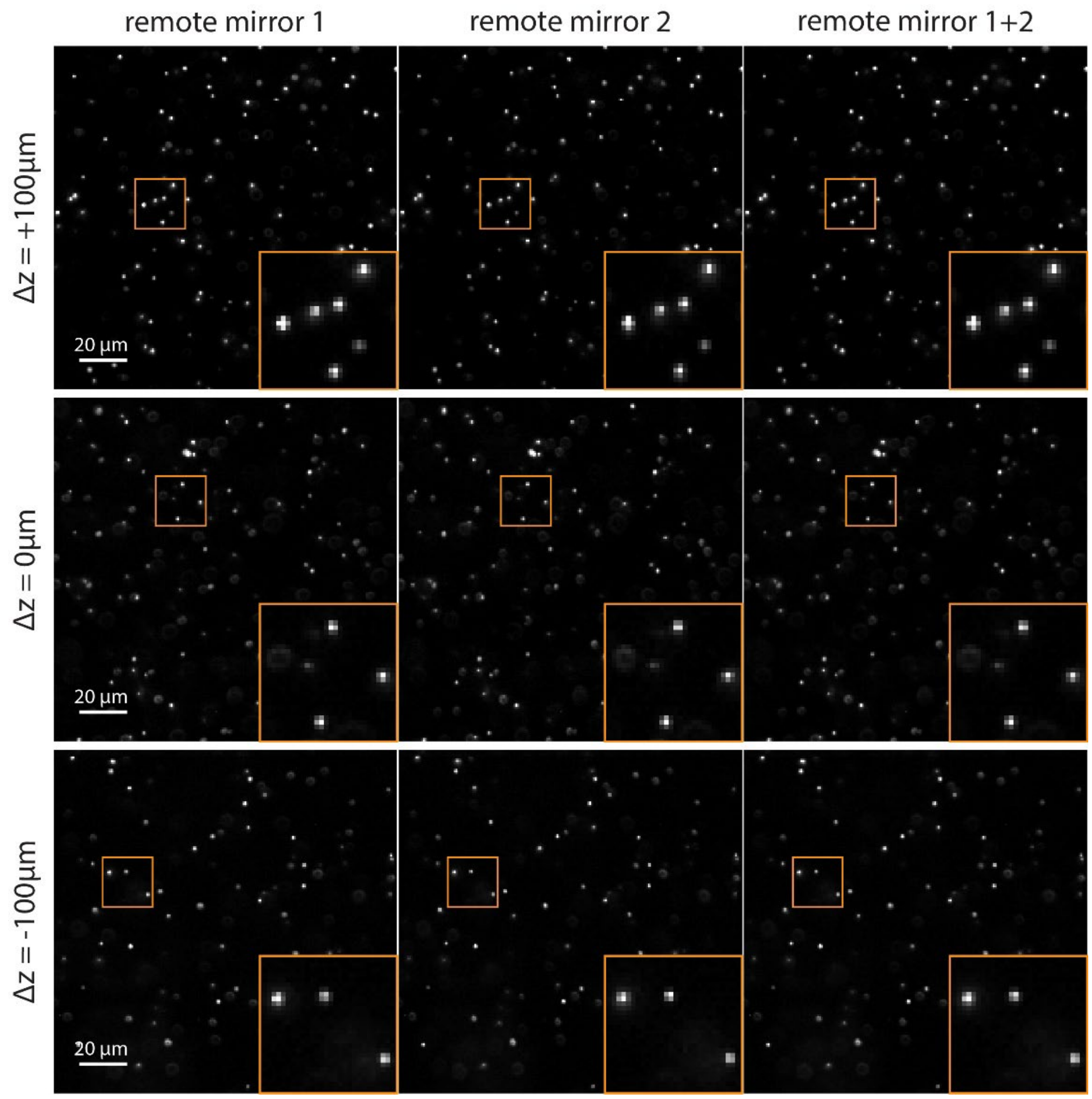

Supplementary Figure 1. High-precision alignment of images from two remote scanning mirrors across a 200-μm axial field-of-view. Left column, images of a fluorescent bead sample captured at three axial positions (top, Δz = +100μm, middle, Δz = 0μm, bottom, Δz = -100μm) using remote scanning mirror 1. Middle column, images of the fluorescent bead sample captured at the same axial positions as the left column using remote scanning mirror 2. Right column, overlay of images of the fluorescent bead sample at the three axial positions from both remote scanning mirrors. A magnified view of the smaller orange boxed area is shown in the bottom right corner of each image. Image intensities were normalized for display.

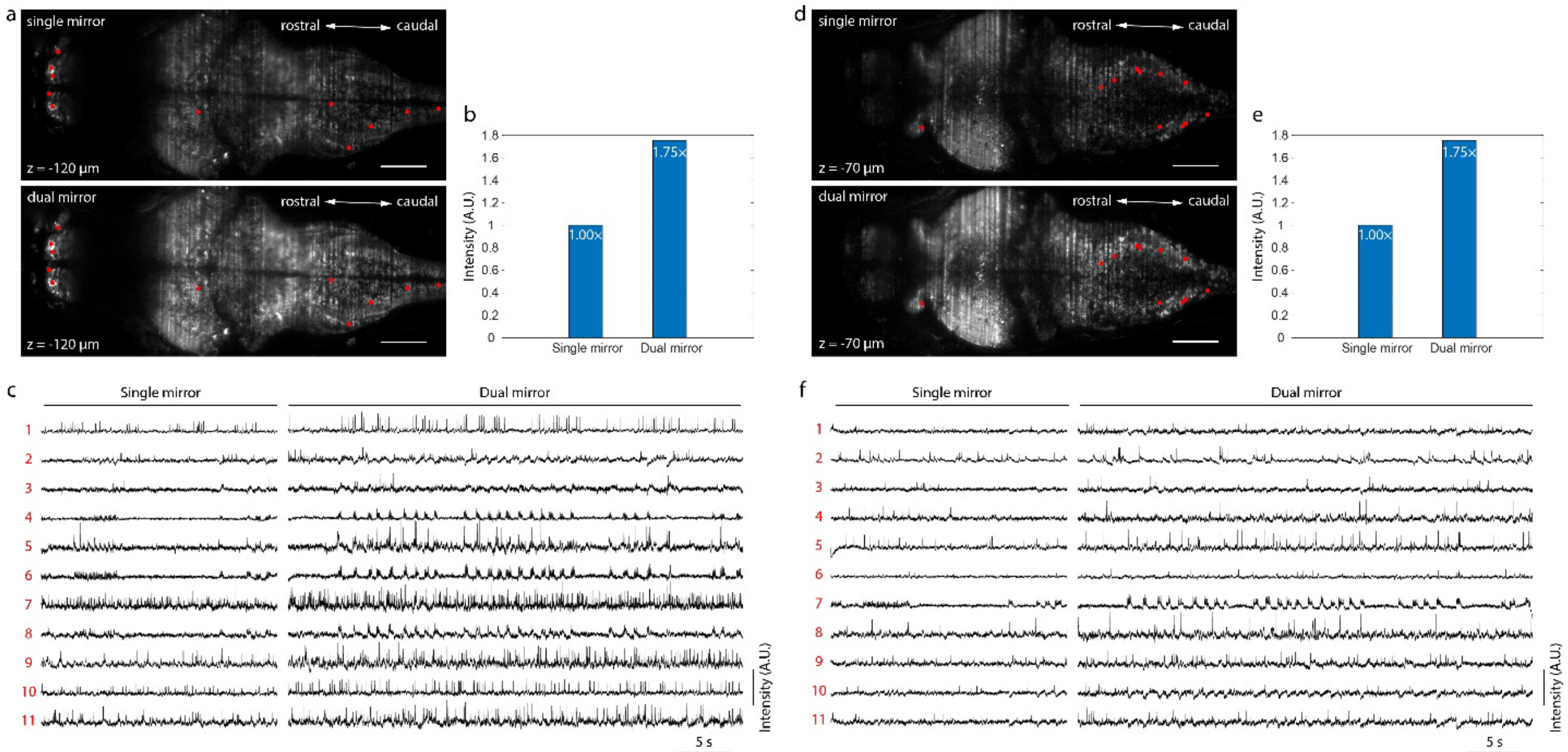

Supplementary Figure 2. Comparison of image intensity and signal amplitudes acquired with single mirror and dual mirror remote scanning. (a) Raw images of a randomly selected zebrafish brain plane (out of 30) acquired with single (top) and dual (bottom) remote scanning mirrors. Red spots mark 11 randomly selected neurons in the plane exhibiting neural activity. Moving stripes were addressed later using the computational method described in **Supplementary Note 4** and **Methods**. Scale bar: 100 µm. **(b)** The use of dual remote scanning mirrors increased the image intensity of this plane by 75%. **(c)** Intensity traces of the same neurons (highlighted by red spots in (a)) were compared between using single and dual remote scanning mirrors. ROIs affected by moving stripe artifacts were computationally identified and excluded for this comparison. **(d-f)** The same analysis as in (a-c) was performed for another randomly selected plane of the same zebrafish brain.

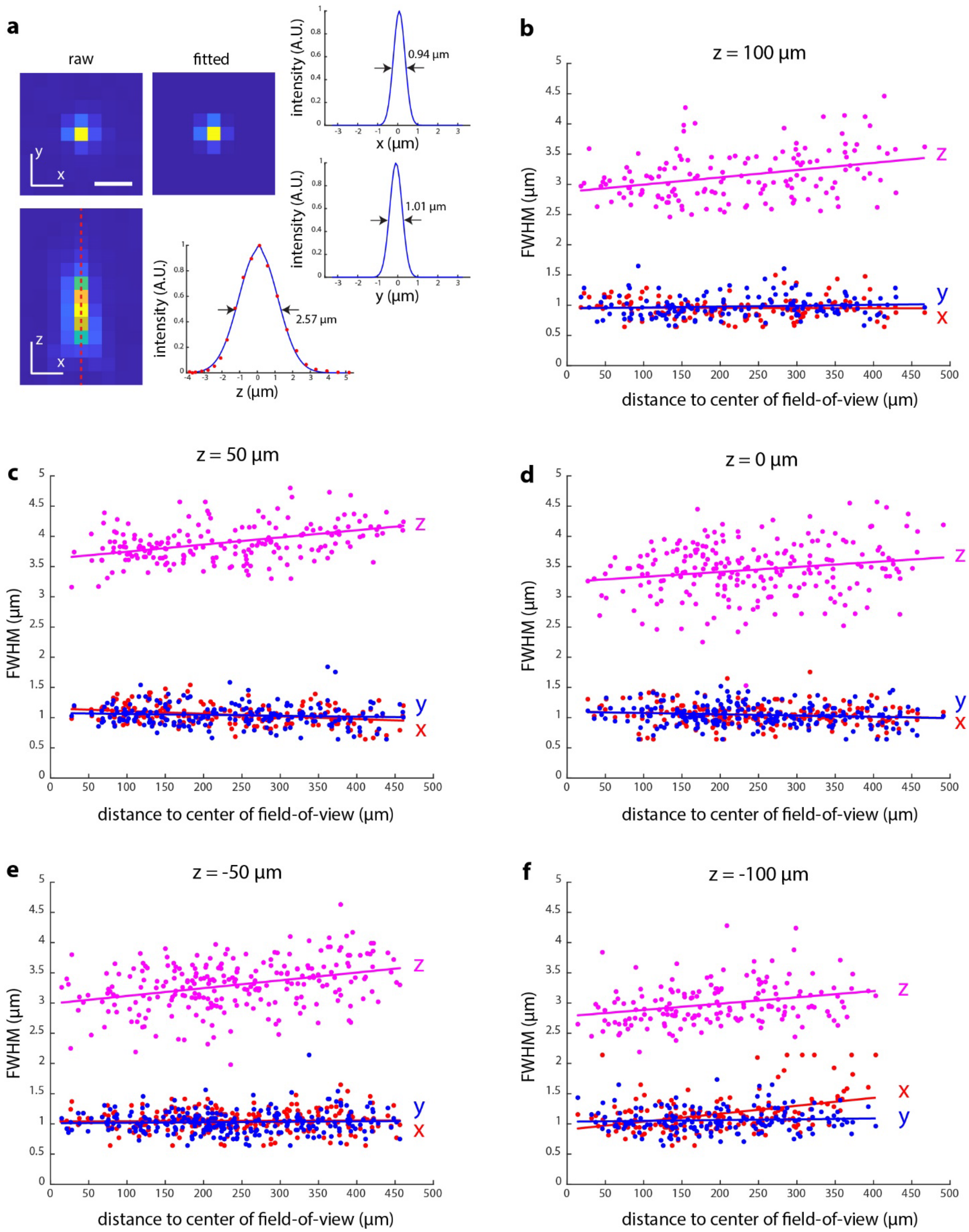
a
raw
fitted
y
x
z
x
intensity (A.U.)
x (μm)
0.94 μm
y (μm)
1.01 μm
z (μm)
2.57 μm
b
z = 100 μm
c
z = 50 μm
d
z = 0 μm
e
z = -50 μm
f
z = -100 μm
FWHM (μm)
distance to center of field-of-view (μm)
z
y
x

Supplementary Figure 3. Characterization of system point spread functions (PSFs) over the entire imaging field-of-view. (a) Measuring the full-width-at-half-maximum (FWHM) of a PSF. On the left, the raw xy and xz sections that intersect the brightest voxel of the 3D PSF are shown. The xy section is fitted by a 2D Gaussian function. The fitted image is displayed in indicated panel. On the right, x and y cross-section profiles of the fitted Gaussian function are shown, and FWHM values are marked. The axial (z) FWHM is determined by fitting the PSF's axial line profile with a double Gaussian function. **(b-f)** Visualization of the FWHM measurements of PSFs at varying radial positions and depths.

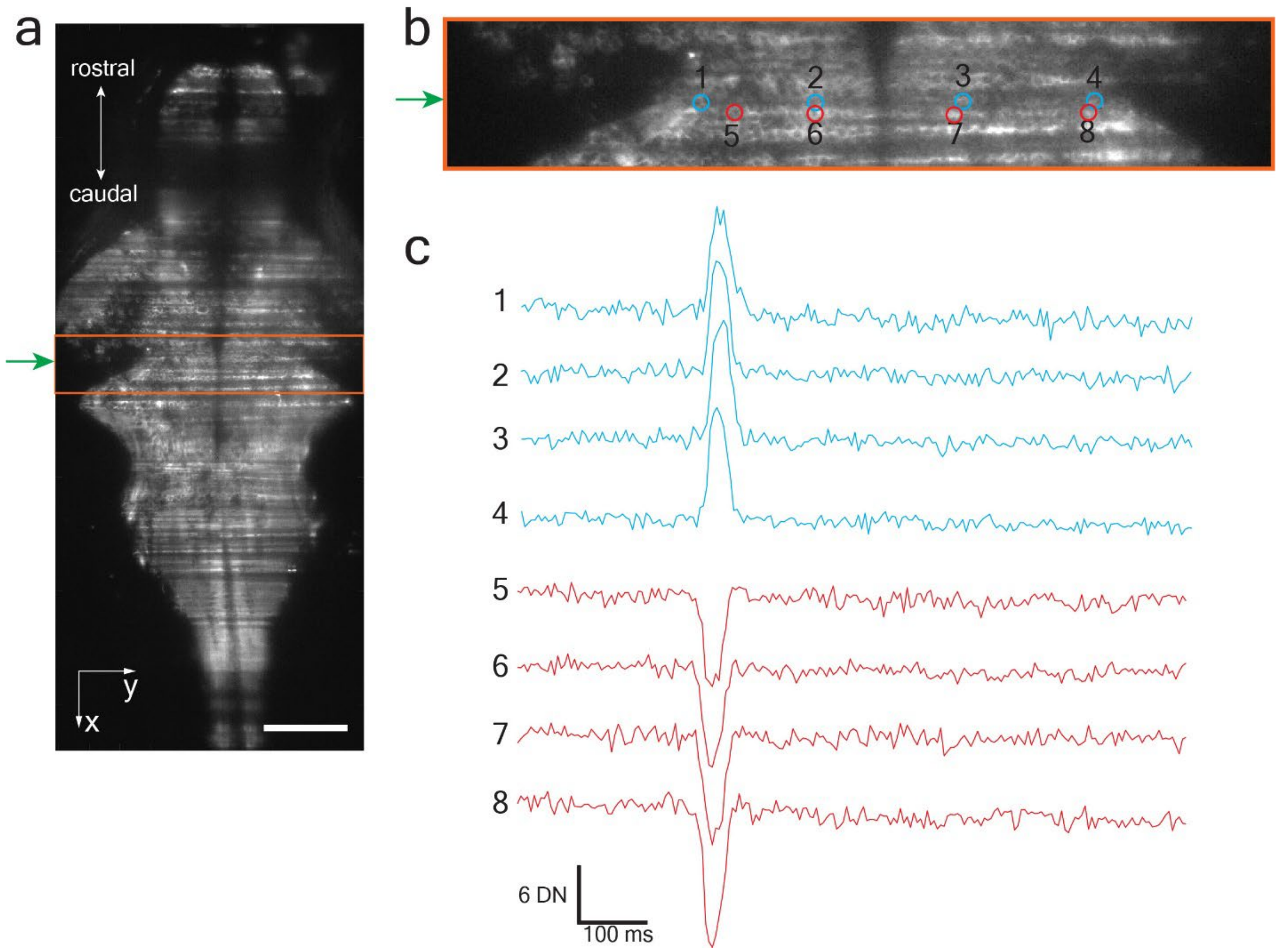

Supplementary Figure 4. Representative pulse-like artifacts on the temporal traces of ROIs affected by the stripe artifacts cast by small moving objects in light-sheet imaging. (a) Raw image frame from a video of a z-plane of the larval zebrafish brain without stripe mitigation. The light sheet is illuminated from the left side of the brain (green arrow). Scale bar: 100 μm. **(b)** Zoom-in view of the orange boxed region in (a). The raw temporal traces from arbitrarily annotated circular ROIs (cyan and red) are extracted. ROIs with the same color lay on the same artifact stripe. **(c)** Extracted raw temporal traces from the ROIs in (b). DN: digital number, measurement of the pixel intensity. Also see Supplementary Video 1.

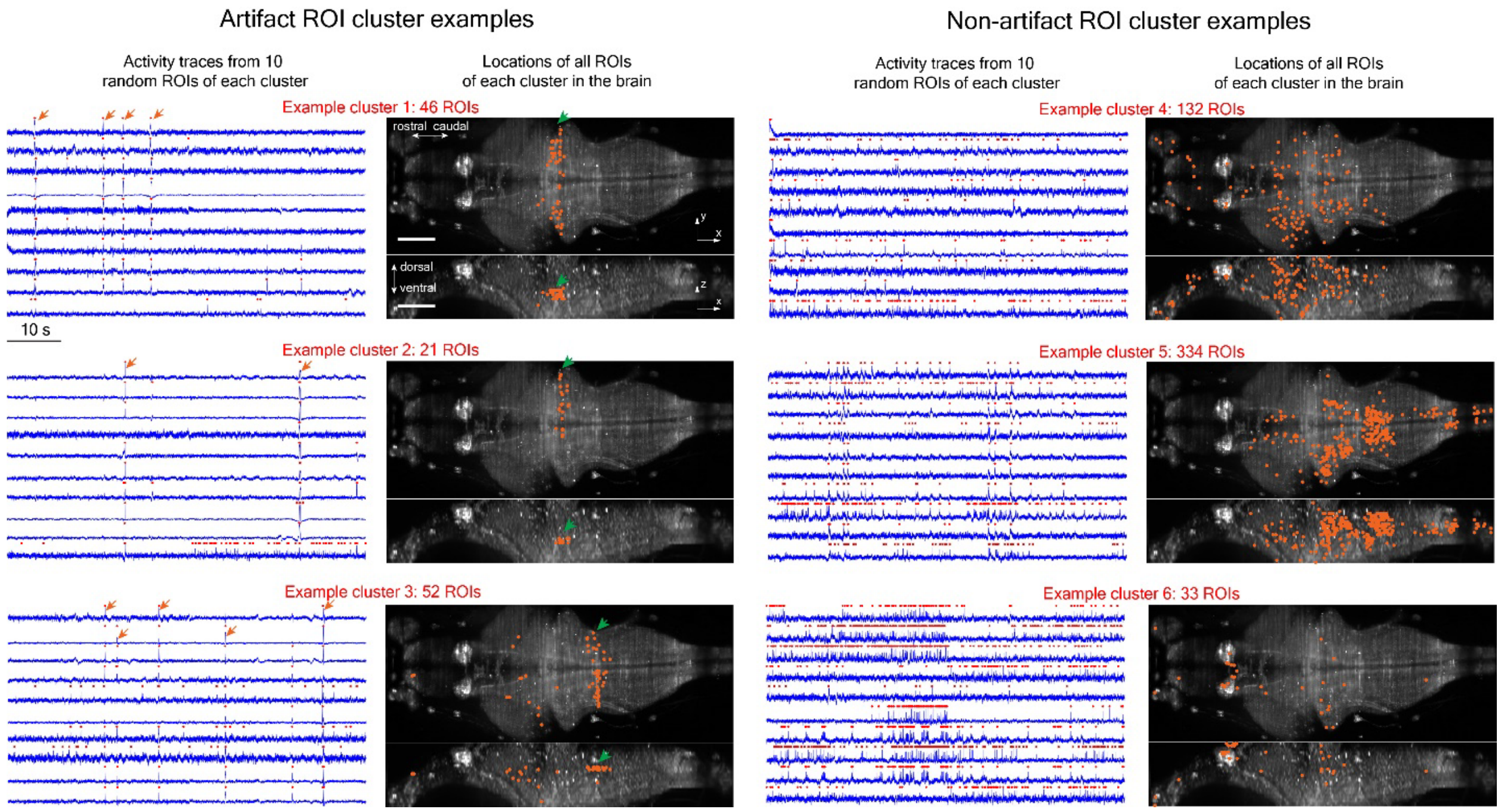

Supplementary Figure 5. Temporal traces and spatial distributions of randomly selected ROI clusters from Fish B that were contaminated (left column) and not contaminated (right column) by moving stripe artifacts. In each panel, the left side displays the temporal traces of 10 randomly selected ROIs within the cluster, plotted in blue. Detected spikes are marked as red dots on the traces. Orange arrows highlight putative synchronous pulse-like artifacts. On the right side of each panel, the spatial locations of the ROIs (orange dots) within the cluster are overlaid on the axial and lateral maximum intensity projections of the zebrafish brain. Green arrows indicate ROI clusters with spatial distributions resembling the shape of moving stripe artifacts along the illumination direction. Clusters exhibiting both synchronous pulse-like artifacts in their temporal traces and spatial distribution patterns similar to the stripe artifacts (example cluster 1-3 in the left column) were excluded from further analysis. Scale bar: 100 μm.

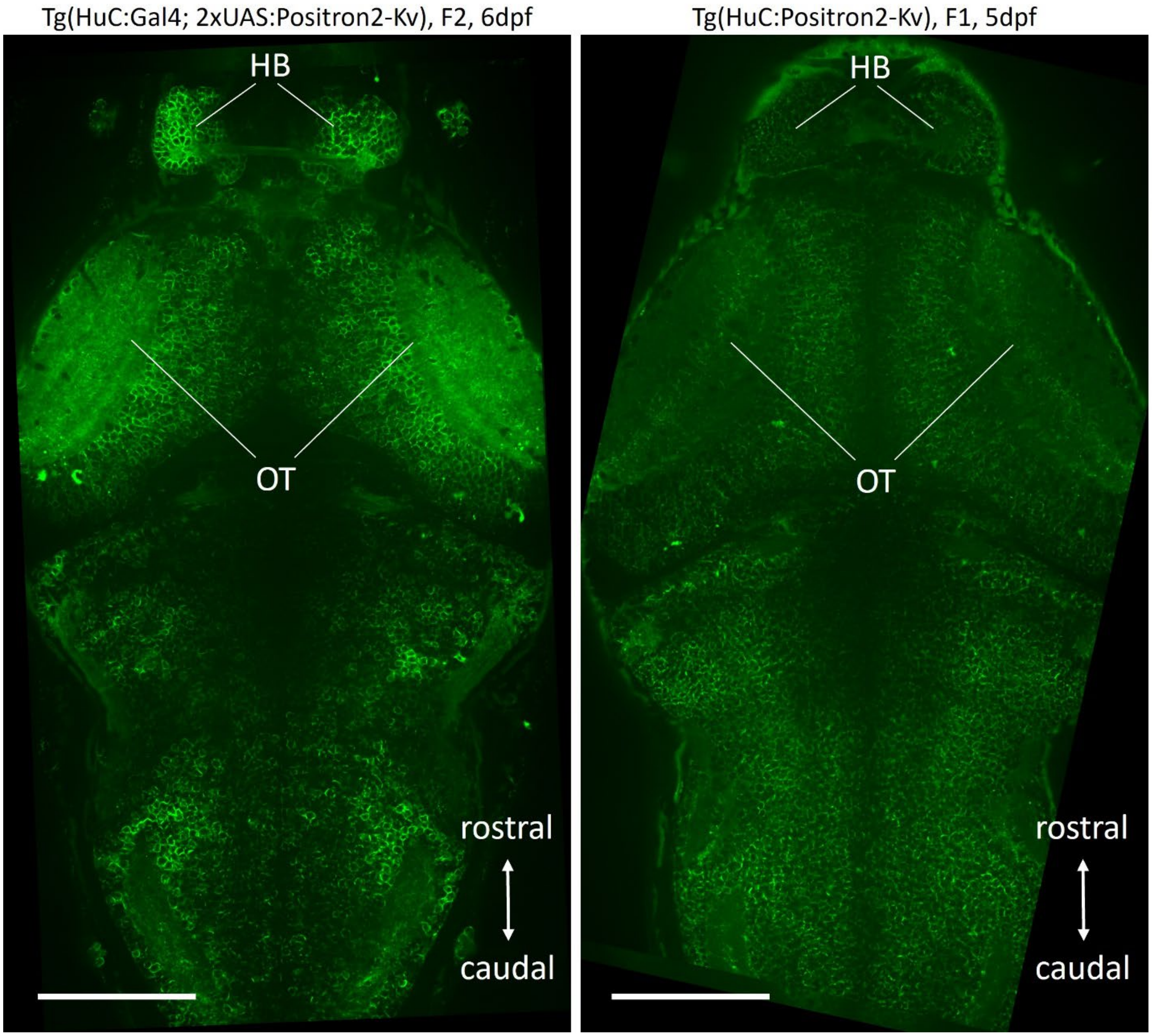

Supplementary Figure 6. Comparison of Positron2-Kv expression in zebrafish brains using different promoters. Confocal microscopy images of zebrafish brains expressing Positron2-Kv using Gal4/2×UAS (left, F2 generation at 6 dpf) and HuC (right, F1 generation at 5 dpf) promoters. Images were acquired using the same microscope settings and displayed using the same intensity scale. Note confocal imaging does not generate stripe artifacts as it excites samples using high-NA light cones instead of parallel light. OT: optic tectum. HB: habenula. Scale bar: 100 µm.

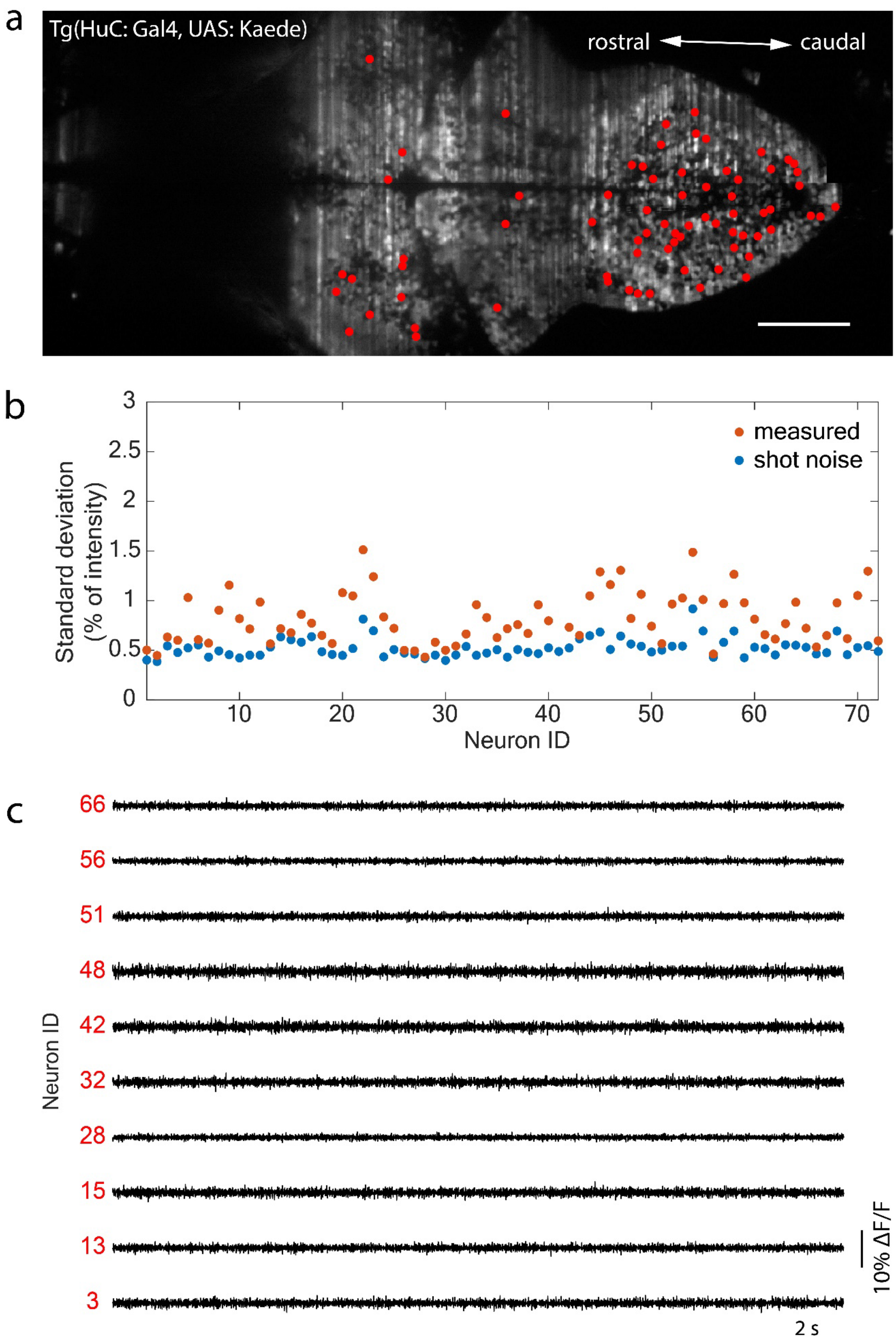
a
Tg(HuC: Gal4, UAS: Kaede)
rostral
caudal
b
Standard deviation (% of intensity)
measured
shot noise
Neuron ID
c
66
56
51
48
42
32
28
15
13
3
Neuron ID
10% ΔF/F
2 s

Supplementary Figure 7. Quantification of the baseline noise using pan-neuronal Kaede fish. (a) A randomly selected single-plane image (out of 30) of the Kaede fish brain was analyzed. Neurons (red spots) indicated were randomly selected across areas in the plane that did not visually overlap with any moving stripe in the raw video, for signal trace analysis. Scale bar: 100 µm. **(b)** To quantify the baseline noise, we calculated the standard deviation of the signal traces of the neurons marked in (a) and plotted the standard deviations (orange dots) as percentages relative to the baseline intensity. The theoretical shot noise of each signal trace was also plotted (blue dots). **(c)** Signal traces of 10 neurons randomly selected from the 72 analyzed neurons.

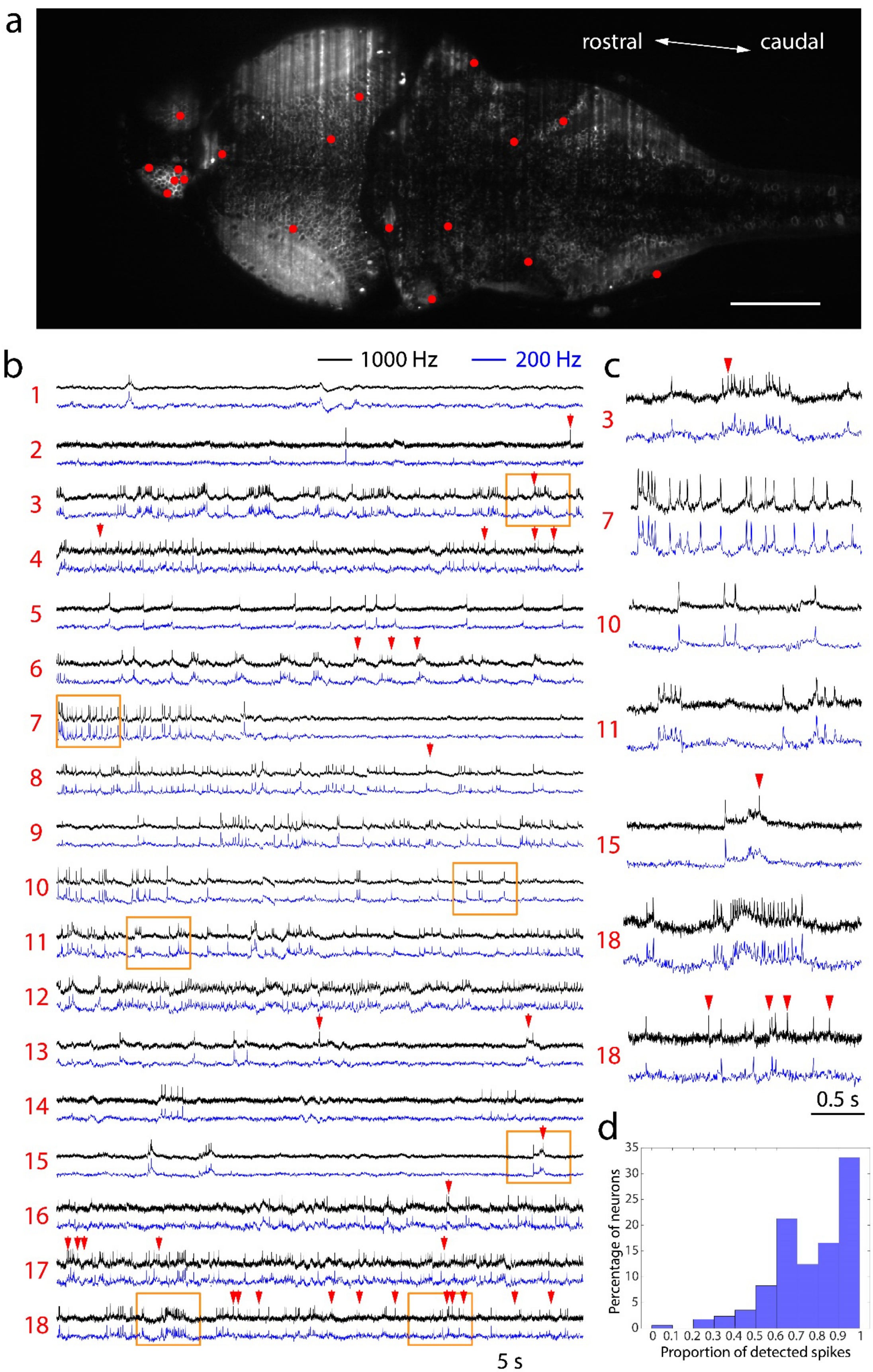
a
rostral
caudal
b
1000 Hz
200 Hz
1
2
3
4
5
6
7
8
9
10
11
12
13
14
15
16
17
18
5 s
c
3
7
10
11
15
18
18
0.5 s
d
Percentage of neurons
Proportion of detected spikes
0
5
10
15
20
25
30
35
0 0.1 0.2 0.3 0.4 0.5 0.6 0.7 0.8 0.9 1

Supplementary Figure 8. Quantification of spike detection rate using the Positron2-Kv indicator at a 200-Hz imaging rate. (a) Raw image of an imaged zebrafish brain plane. Red spots mark randomly selected neurons (out of a total of 201 neurons that showed spikes during the recording period and had SNR_{VolPy}>4. ROIs affected by moving stripe artifacts were computationally identified and removed prior to all analysis in this figure) whose traces are shown below in (b). Scale bar: 100 μm. **(b)** Neural traces extracted from the original recording at a 1-kHz imaging rate with a 40-μs exposure time (black traces) are compared with the neural traces extracted from a 200-Hz video downsampled from the 1-kHz recording. Red arrows indicate locations where spikes were missed in the 200-Hz traces. Orange boxes highlight regions magnified in (c). **(c)** Magnified views of the orange-boxed regions in (b). **(d)** Histogram showing the percentage of neurons categorized by their spike detection rates (defined as the number of detected spikes divided by the number of all spikes) in the 200-Hz neural traces.

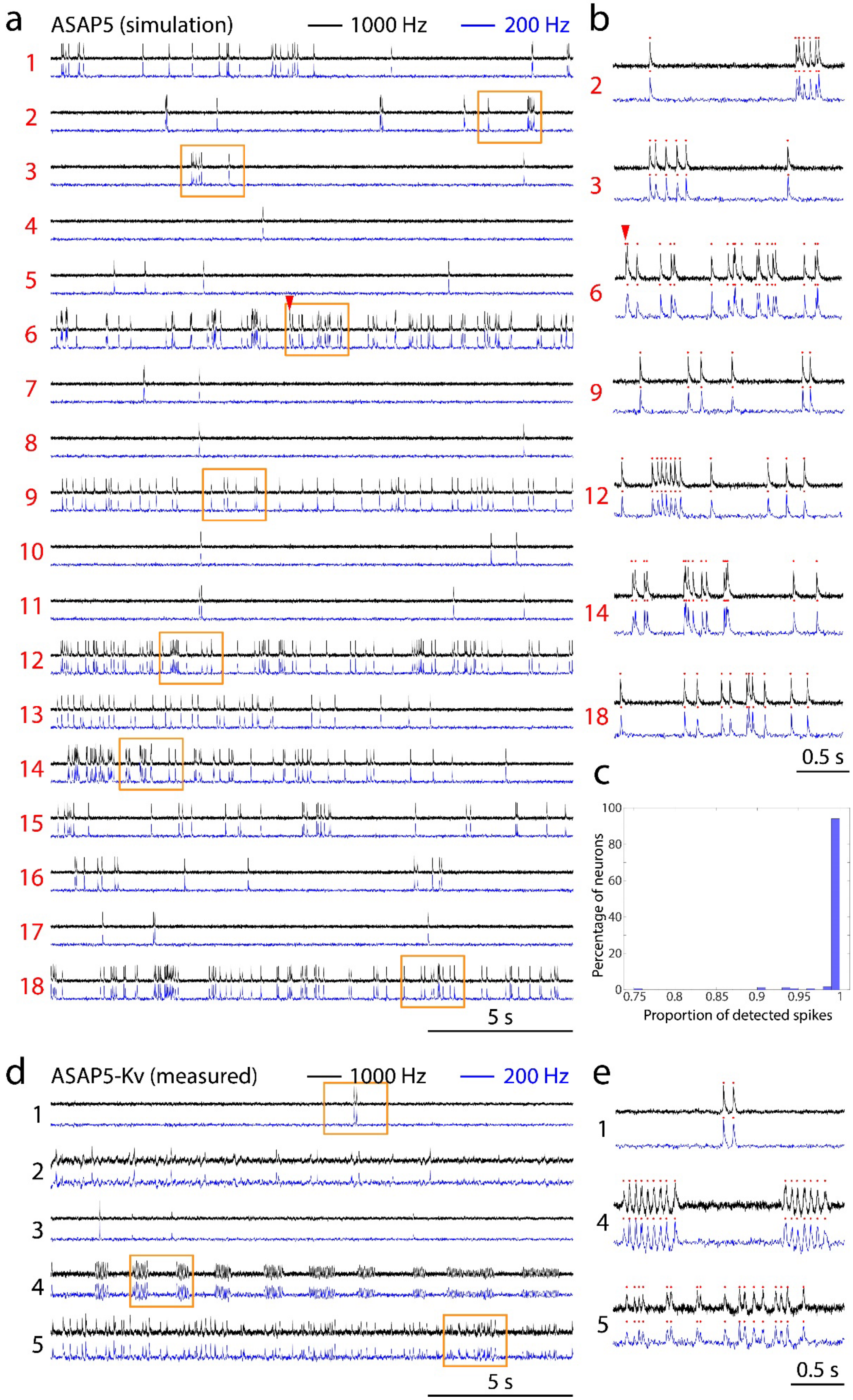
a
ASAP5 (simulation)
1000 Hz
200 Hz
1
2
3
4
5
6
7
8
9
10
11
12
13
14
15
16
17
18
5 s
b
2
3
6
9
12
14
18
0.5 s
c
Percentage of neurons
Proportion of detected spikes
0
20
40
60
80
100
0.75
0.8
0.85
0.9
0.95
1
d
ASAP5-Kv (measured)
1000 Hz
200 Hz
1
2
3
4
5
5 s
e
1
4
5
0.5 s

Supplementary Figure 9. **Quantification of spike detection rate using the ASAP5-Kv indicator at a 200-Hz imaging rate. (a)** Simulated neural traces at a 1-kHz imaging rate (black traces) are compared with the neural traces down sampled to a 200-Hz imaging rate. The red arrow indicates a location where a spike was missed in the 200-Hz traces. Orange boxes highlight regions magnified in (b). All traces were flipped vertically and normalized by their maximum amplitude for display. **(b)** Magnified views of the orange-boxed regions in (a). Red dots indicate detected spikes. **(c)** Histogram showing the percentage of neurons categorized by their spike detection rates in the 200-Hz neural traces, based on simulation results. **(d)** Measured ASAP5-Kv neural traces in larval zebrafish at a 1-kHz imaging rate (black traces) are compared with the neural traces down sampled to a 200-Hz imaging rate. All traces were flipped vertically and normalized by their maximum amplitude for display. ROIs affected by moving stripe artifacts were computationally identified and removed before neural activity analysis. Orange boxed areas are shown in magnified views in (e). Red dots in (e) indicate detected spikes.

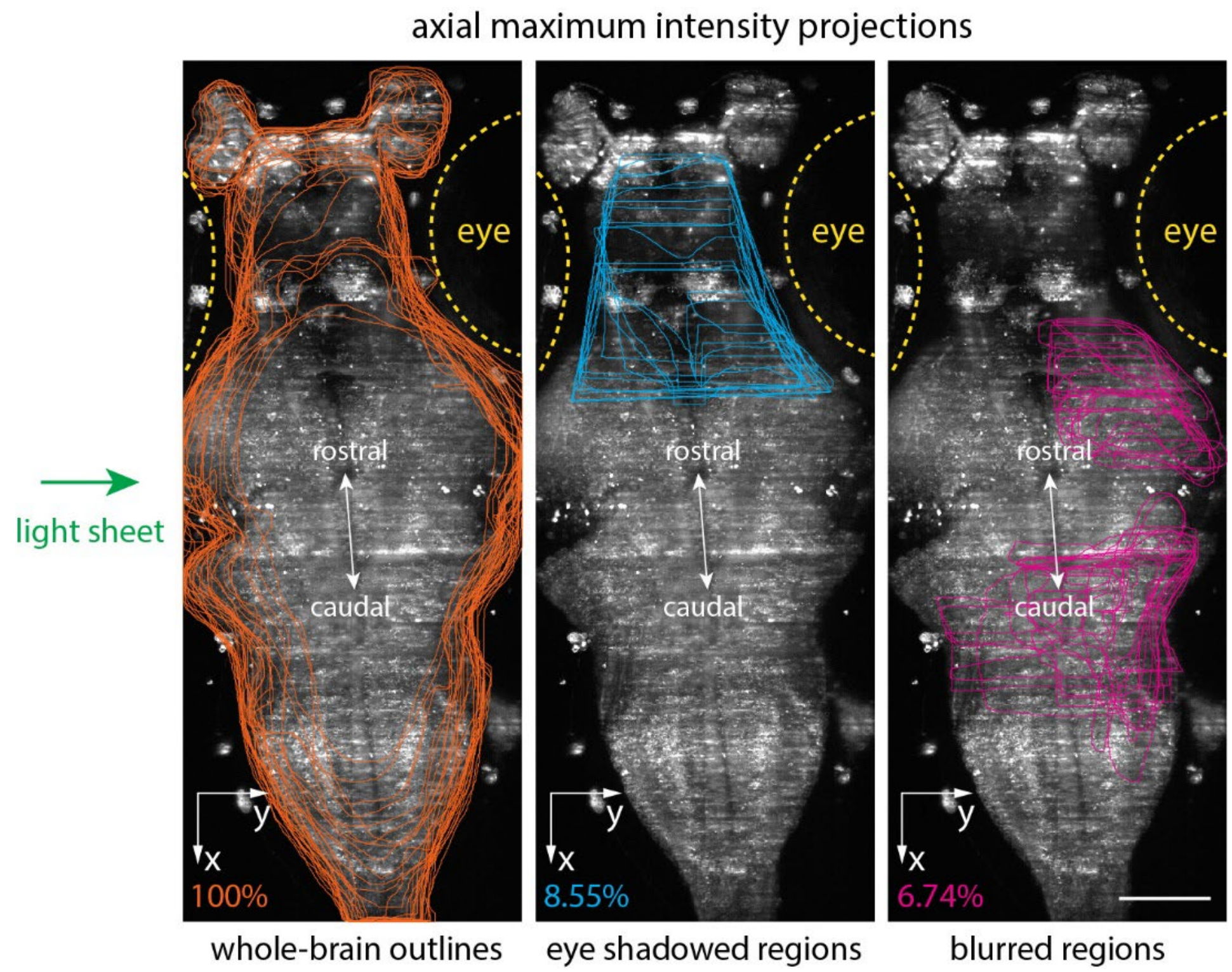

Supplementary Figure 10. **Quantifying the eye shadowed regions and the blurred regions as percentages of the imaged whole brain.** Contours of the whole brain (orange), the regions that were shadowed by eyes (cyan), and the blurred regions where single cells cannot be resolved (magenta), were drawn on individual z-plane images (30 planes in total, 5.86 μm z step size) of the whole zebrafish brain. To draw these contours, we visually examined the raw image of each z-plane and drew the 2-D contours of the whole brain (orange), the dark region shadowed by the fish's eye (cyan), and the regions where we could not visually distinguish single cells (magenta). These 2-D contours were projected along the z axis and displayed on the axial maximum intensity projections (MIPs) of the imaged brain. Yellow dashed lines indicate the fish's eyes. Within the imaged brain (left, orange contours), 8.55% (middle, cyan contours) of the full brain region was shadowed by fish's eyes, 6.74% (right, magenta contours) was too blurred to visually resolve single cells. The light sheet (green arrow) was illuminated towards the left side of the brain. This analysis was done using static raw light-sheet images. Scale bar: 100 μm.

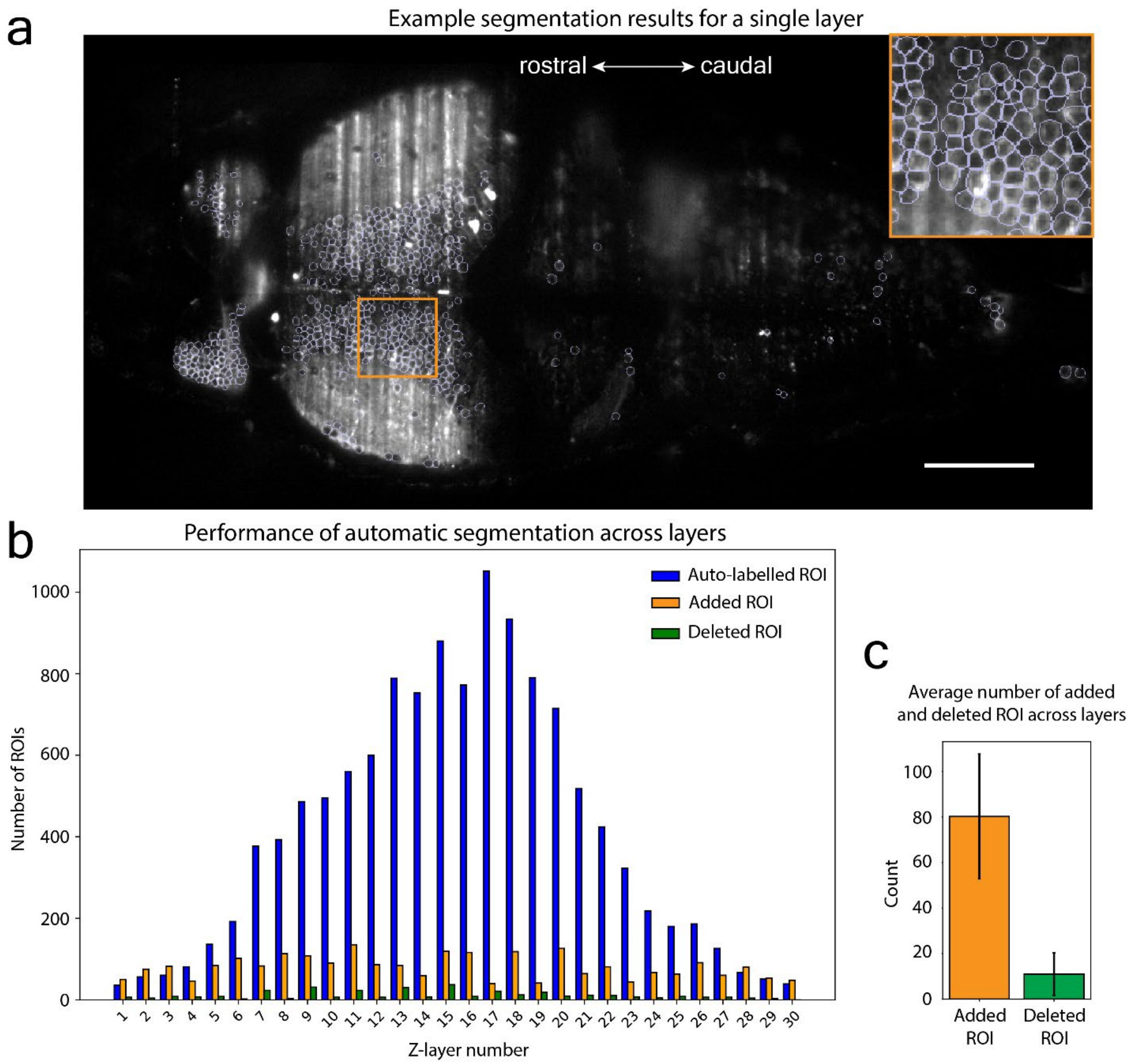

Supplementary Figure 11. Performance of our automatic ROI segmentation model. **(a)** The segmentation results for a randomly selected single z-layer (out of 30) from a single unlabeled fish. The ROI contours (light purple lines) are overlaid on the image. A magnified view of the orange boxed area is shown in the top right corner. The model labeled ROIs using temporally averaged images and did not exclude ROIs affected moving stripe artifacts. Scale bar: 100 μm. **(b)** Segmentation performance of the model on an entire fish. Blue bar: the number of ROIs labeled by the model; orange bar: the number of ROIs added by the experimenter, indicating ROIs that the model missed; green bar: the number of ROIs deleted by the experimenter, indicating ROIs that the model falsely labeled. Z-layers are numbered according to their spatial depth in the brain. Smaller numbers correspond to deeper layers. **(c)** On average, the experimenter added 80.2 ± 27.5 ROIs (mean ± standard deviation, n=30 layers) and removed 10.9 ± 9.4 (mean ± standard deviation, n=30 layers) ROIs from the model's output.

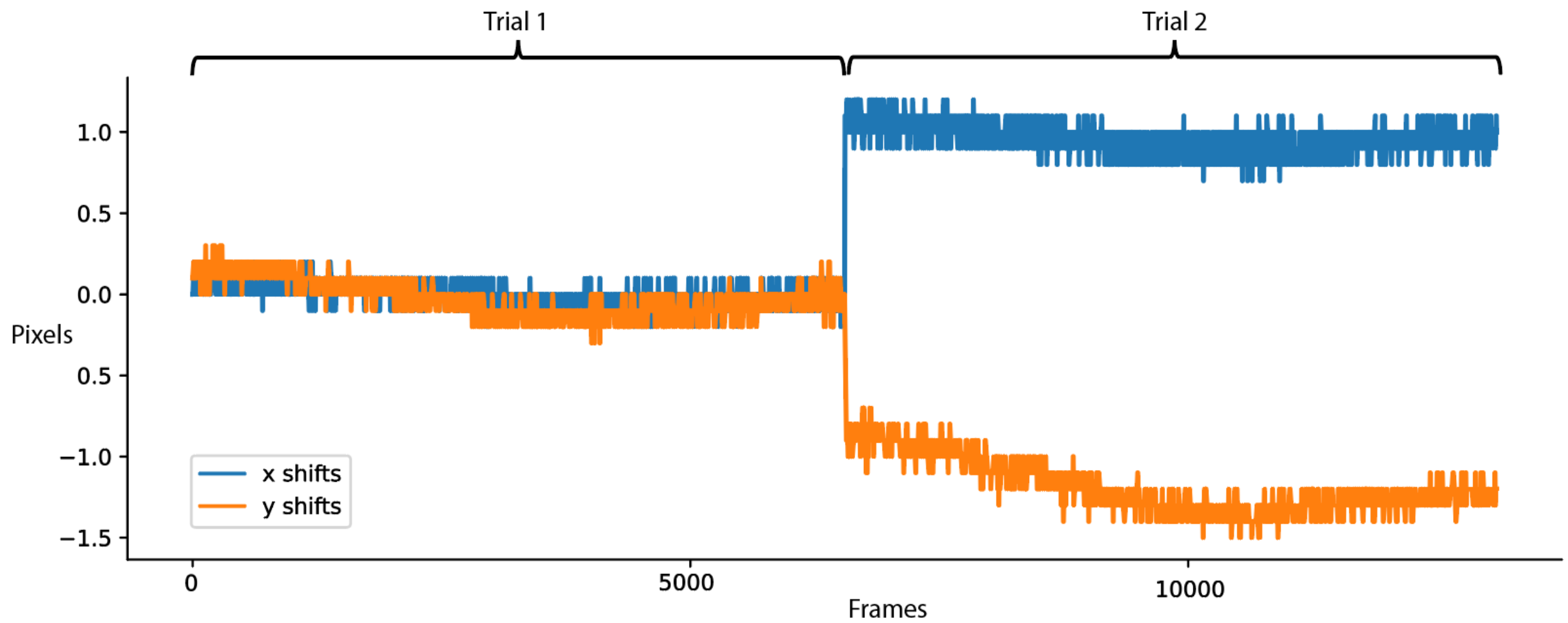

Supplementary Figure 12. Motion correction result for a randomly selected brain layer. The corrected drift in at each layer, as measured by the motion correction algorithm NoRMCorre, within an imaging trial (35 s) was less than 1 pixel (0.73 μm), and the drift between two trials was < 2 pixels. To align the neuron positions of two trials, trial 2 used the first frame of trial 1 as reference for motion correction. There was a 20-min dark session between the two trials.

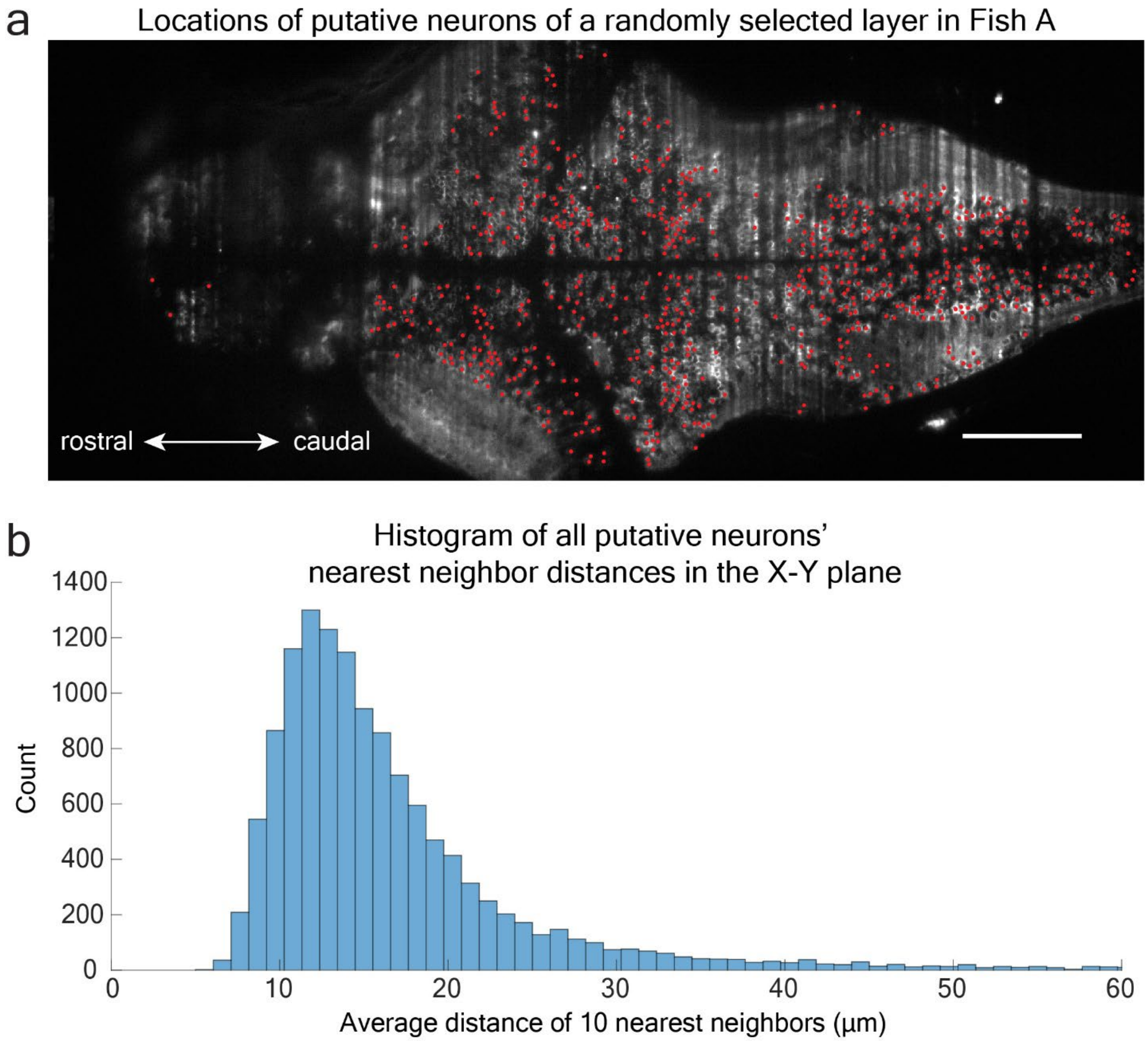

Supplementary Figure 13. A quantification of putative neuron density across the x-y planes in Fish A. We calculated the average distance of each putative neuron's centroid to its 10 nearest neighbors' centroids along the X-Y directions within 30 individual z-planes of Fish A. The average distance was 17.8 ± 15.2 µm (mean ± SD, n = 12935 neurons). ROIs affected by moving stripe artifacts were computationally identified and excluded for this analysis. **(a)** The locations of putative neurons (red dots) of a randomly selected layer in Fish A are shown. Scale bar: 100 µm. **(b)** Histogram illustrating the counts of putative neurons in Fish A, categorized by their mean distances to the ten nearest neighboring ROIs.

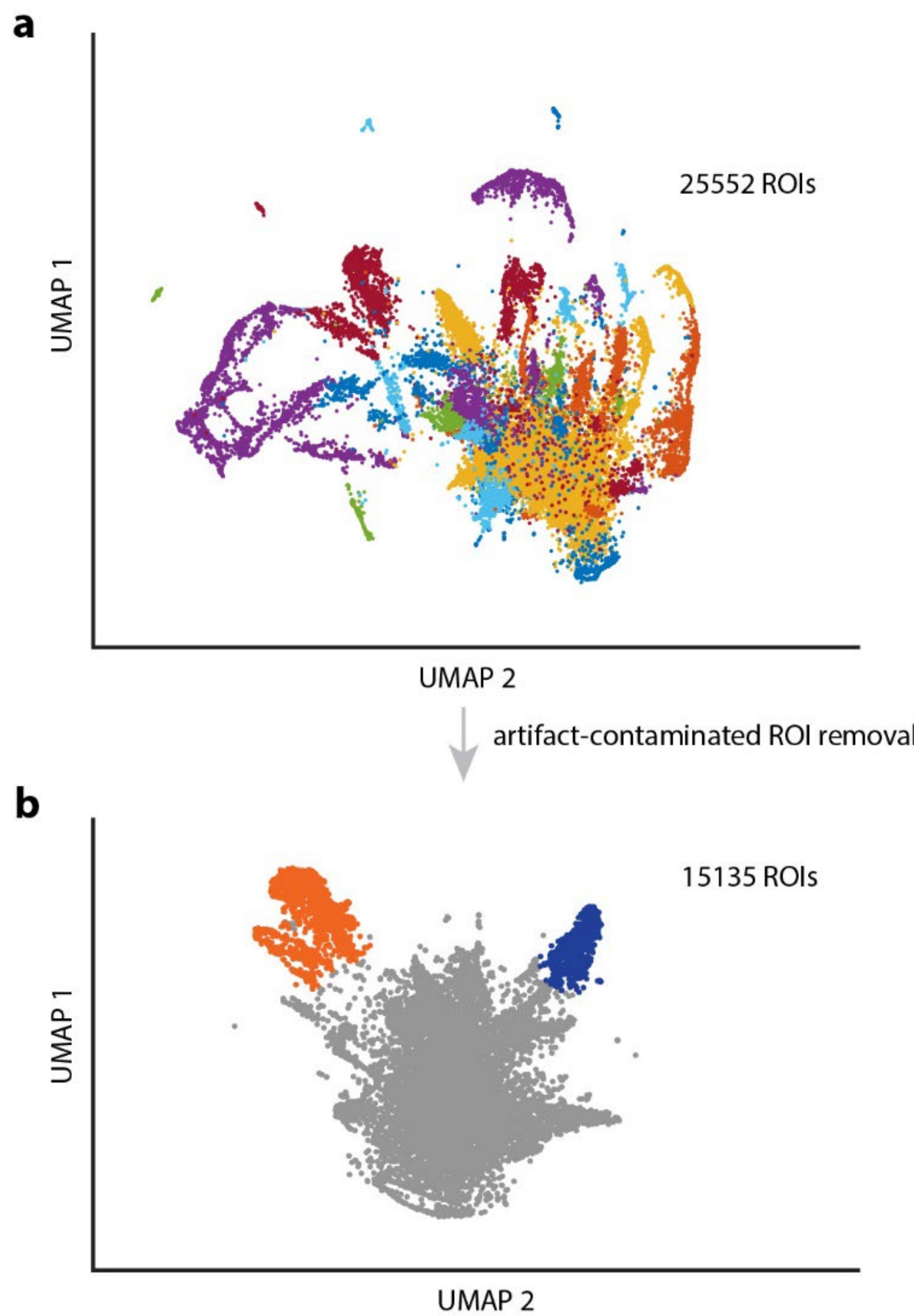

Supplementary Figure 14. 2D UMAP visualizations of ROIs before and after the removal of artifact-contaminated ROIs in Fish B. (a) 25552 ROIs were mapped into 2D space using the UMAP algorithm based on their temporal traces. Different colors represent different clusters. **(b)** After identifying and removing the ROIs whose temporal traces are contaminated by the stripe artifacts, 15135 ROIs remained and were considered as putative neurons. The putative neurons were then clustered into three groups (indicated in three colors) for subsequent analysis.

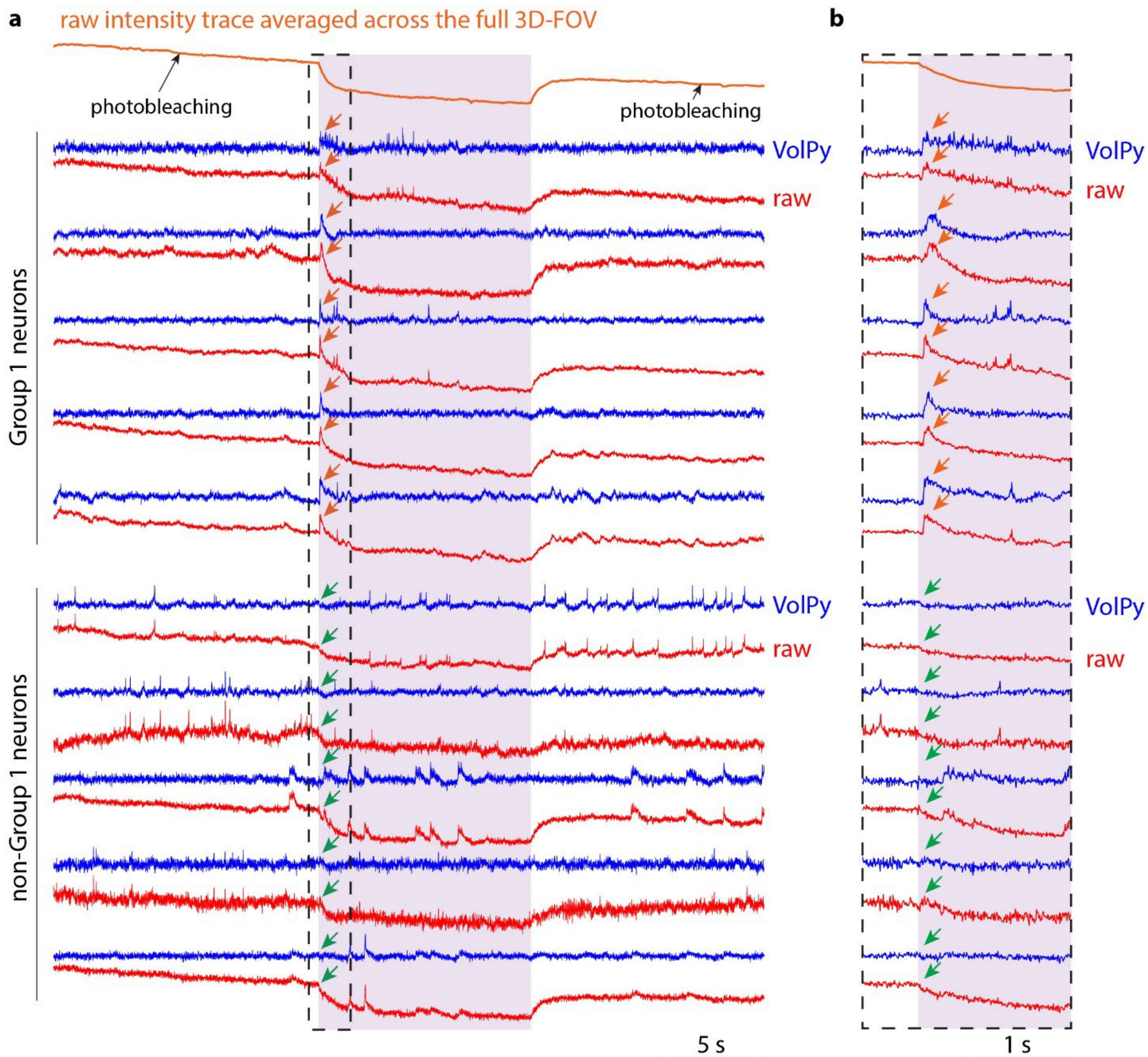

Supplementary Figure 15. Photoswitching effects in VolPy-extracted temporal traces, and raw temporal traces, of neurons in Group 1 (i.e., neurons that have increased activity in response to the onset of the light stimulus) and neurons not in Group 1. (a) Comparison of the VolPy (blue) and raw (red) traces of neurons. Purple color indicates the period of light stimulus. For Group 1 neurons, orange arrows highlight increased activity following the light stimulus onset in both the raw temporal traces and the VolPy-extracted temporal traces. Green arrows highlight corresponding locations in the raw traces and VolPy-extracted traces of non-Group 1 neurons. ROIs affected by moving stripe artifacts were computationally identified and removed for this analysis. **(b)** Enlarged view of the dashed box in (a).

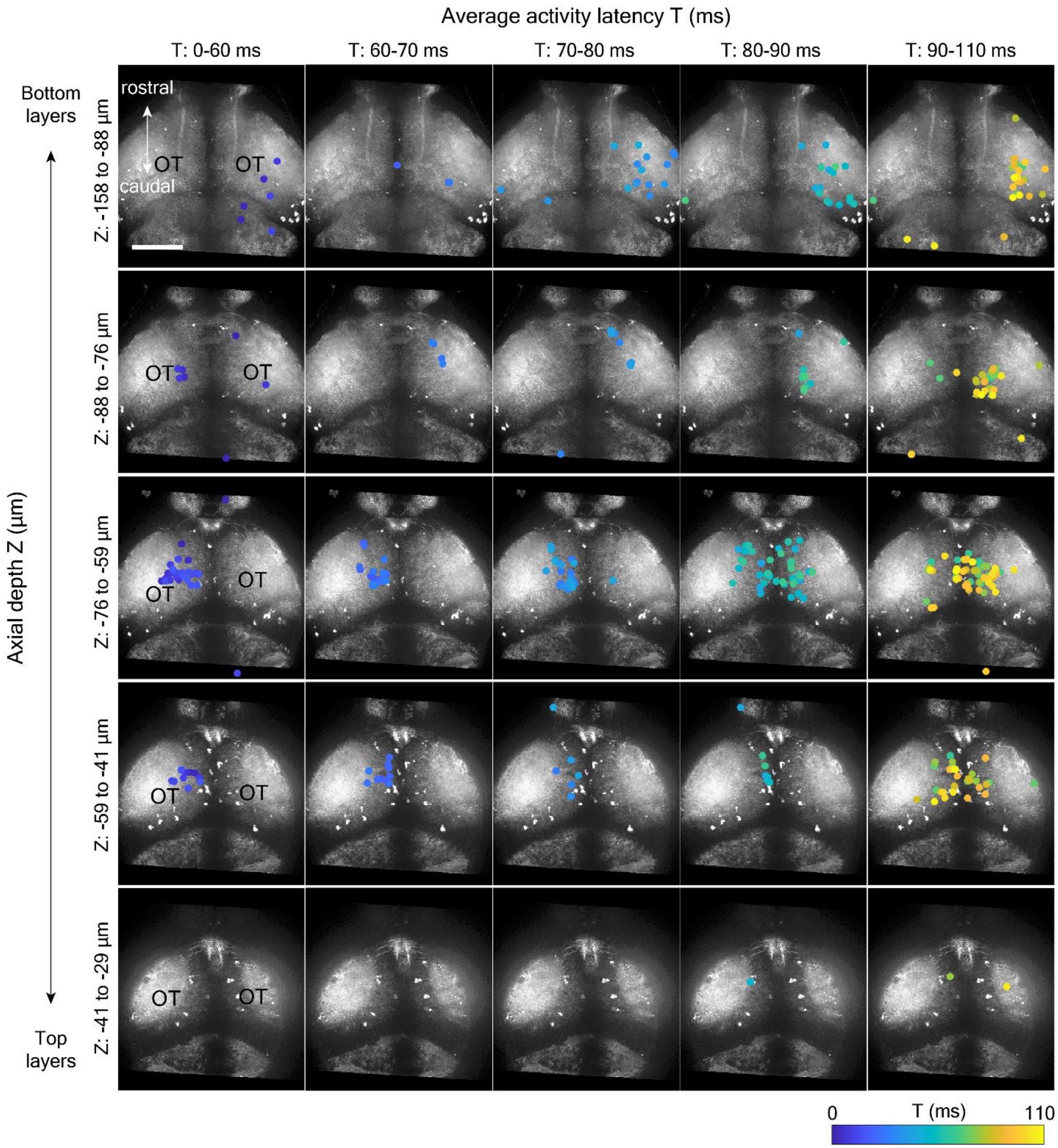

Supplementary Figure 16. Locations of Fish C's UV-responding neurons (Group 1 neurons) overlaid with confocal images of the optic tectum region. Each row shows a z-section of the brain of a certain depth range, as indicated on the left of the row. Within each row, from left to right, neurons falling within different activity latency windows (indicated on top of each column) are shown. The depth ranges and latency windows were chosen arbitrarily to better visualize the spatial-temporal correlations of UV-elicited neural activity. Neurons are plotted as dots in colors coded by their activity latency. The bright white dots are occasional neurons, visible in these z-projections but found here and there in all fish, with very high expression levels of GEVIs, for

unknown reasons. ROIs affected by moving stripe artifacts were computationally identified and excluded for this analysis. OT, optic tectum. Scale bar: 100 μm.

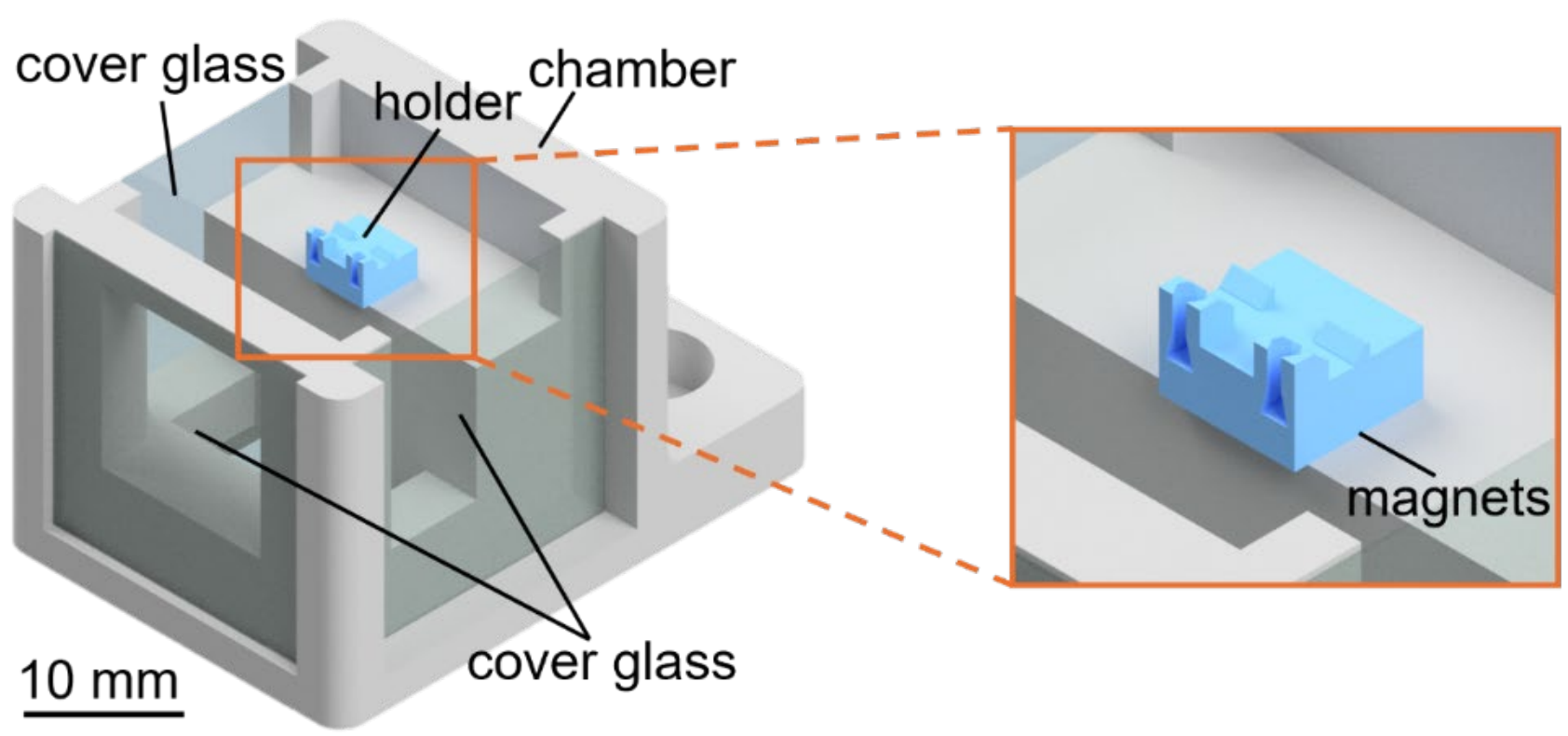

Supplementary Figure 17. Illustration of the fish holder (blue) and chamber (gray). The holder was attached to the chamber using two magnets. There were two slots on the holder where the fish could be mounted, depending on which side the light sheet came from.

Supplementary Tables

Supplementary Table 1. Imaging conditions for the light-sheet data shown in figures

Figure number	Zebrafish genotype	Number of z-planes	Z-step size	Volume rate	Single or dual remote mirrors	Method to handle moving stripes	Light-sheet arm
Fig. 2	Tg(HuC:Gal4; 3×UAS:Positron2-Kv); nacre	30	5.86 µm	200.8Hz	Single	Computational (affected ROI removal)	no RS
Fig. 3 (Fish B)	Tg(HuC:Gal4; 3×UAS:Positron2-Kv); nacre	30	5.86 µm	200.8Hz	Single	Computational	no RS
Fig. 4 (Fish A, B)	Tg(HuC:Gal4; 3×UAS:Positron2-Kv); nacre	30	5.86 µm	200.8Hz	Single	Computational	no RS
Fig. 5 (Fish B)	Tg(HuC:Gal4; 3×UAS:Positron2-Kv); nacre	30	5.86 µm	200.8Hz	Single	Computational	no RS
Fig. 5 (Fish C)	Tg(HuC:Gal4; 2×UAS:Positron2-Kv); nacre	30	5.86 µm	200.8Hz	Single	Computational	no RS
Extended Data Fig. 2b	Tg(HuC:Gal4; 2×UAS:Positron2-Kv); nacre	1	-	200.8Hz	Single	-	no RS
Extended Data Fig. 3a	PFA-fixed Tg(HuC:Gal4; 2×UAS:Positron2-Kv); nacre	30	5.86 µm	200.8Hz	Single	-	no RS
Extended Data Fig. 3b	Tg(HuC:Gal4; 2×UAS:Positron2-Kv); nacre	30	5.86 µm	200.8Hz	Single	Biological (transient cardiac arrest)	no RS
Extended Data Fig. 4	Tg(HuC:Gal4; 2×UAS:Positron2-Kv; flk:DsRed); nacre	30	5.86 µm	200.8Hz	Single	-	no RS
Extended Data Fig. 5	Tg(HuC:Gal4; 2×UAS:Positron2-Kv); nacre	20	6 µm	203Hz	Dual	Optical (resonant scanner)	RS
Extended Data Fig. 6	Tg(HuC:Gal4; 3×UAS:Positron2-Kv); nacre<br>Tg(HuC:Gal4; 2×UAS:Positron2-Kv); nacre	30	5.86 µm	200.8Hz	Single	Computational	no RS
Extended Data Fig. 7a	Tg(HuC:Gal4; 2×UAS:Positron2-Kv); nacre	30	5.86 µm	200.8Hz	Single	Visual inspection of raw recording	no RS
Extended Data Fig. 9 (Fish C, D)	Tg(HuC:Gal4; 2×UAS:Positron2-Kv); nacre	30	5.86 µm	200.8Hz	Single	Computational	no RS
Extended Data Fig. 10 (Fish A)	Tg(HuC:Gal4; 3×UAS:Positron2-Kv); nacre	30	5.86 µm	200.8Hz	Single	Computational	no RS
Extended Data Fig. 10 (Fish D)	Tg(HuC:Gal4; 2×UAS:Positron2-Kv); nacre	30	5.86 µm	200.8Hz	Single	Computational	no RS

Supplementary Fig. 2	Tg(HuC:Gal4; 2×UAS:Positron2-Kv); nacre	30	5.86 µm	200.8Hz	Single and dual	Computational	no RS
Supplementary Fig. 4	Tg(HuC:Gal4; 3×UAS:Positron2-Kv); nacre	30	5.86 µm	200.8Hz	Single	-	no RS
Supplementary Fig. 5	Tg(HuC:Gal4; 3×UAS:Positron2-Kv); nacre	30	5.86 µm	200.8Hz	Single	-	no RS
Supplementary Fig. 7	Tg(HuC:Gal4; UAS:Kaede); nacre	30	5.86 µm	200.8Hz	Single	Visual inspection of raw recording	no RS
Supplementary Fig. 8	Tg(HuC:Gal4; 2×UAS:Positron2-Kv); nacre	1	-	1000Hz	Dual	Computational	no RS
Supplementary Fig. 9d,e	Tg(HuC:Gal4; 2×UAS:ASAP5-Kv); nacre	1	-	1000Hz	Dual	Computational	no RS
Supplementary Fig. 10	Tg(HuC:Gal4; 3×UAS:Positron2-Kv); nacre	30	5.86 µm	200.8Hz	Single	-	no RS
Supplementary Fig. 11	Tg(HuC:Gal4; 3×UAS:Positron2-Kv); nacre	30	5.86 µm	200.8Hz	Single	Computational	no RS
Supplementary Fig. 15	Tg(HuC:Gal4; 3×UAS:Positron2-Kv); nacre	30	5.86 µm	200.8Hz	Single	Computational	no RS

Supplementary Table 2. Hardware parts and software used in the imaging setup

Category	Part name in this paper (if applicable)	Figure number	Part description	Part number	Source (Vendor)
Imaging path	OL1	Fig. 1	Imaging objective lens	XLUMPLFLN20XW	Olympus
	L1		Tube lens	TTL200MP	Thorlabs
	L2		Tube lens	TTL200-A + 2× AC508-750-A	Thorlabs
	PBS		Polarized beam splitter	PBS251	Thorlabs
	QW		Quarter wave plate	AQWP10M-580	Thorlabs
	F		Emission filter	FF01-571/72-25	IDEX/Semrock
	L3		Tube lens	TTL200MP + AC508-1000-A	Thorlabs
	OL2		Remote objective lens	UPLXAPO20X	Olympus
	PZ		Piezo scanner	PB4VB2S	Thorlabs
	M		Remote mirror	Customized 2×2×1 mm^3 mirror	Chroma
	KEM	Extended Data Fig. 1b, c	Knife edge mirror	MRAK25-P01	Thorlabs
			Camera relay lens 1	ACT508-1000-A + 3× ACT508-750-A	Thorlabs
			Camera relay lens 2	TL4X-SAP or CFI Plan Apo 4x	Thorlabs or Nikon
	C1		Camera 1	CB024MG-GP-X8G3	Ximea
	C2		Camera 2	CB024MG-GP-X8G3	Ximea
Illumination path (configuration 1, without RS)		Extended Data Fig. 1e	Illumination objective lens	Plan Apo Lambda 4x	Nikon
	TL		Tube lens	ITL200	Thorlabs
	SL		Scan lens	CLS-SL	Thorlabs
	Mask		Optical mask	Customized mask	PhotomaskPORTAL
			Galvo mirror	Saturn 5B	ScannerMAX
	RL1		Relay lens	AC127-075-A	Thorlabs
	RL2		Relay lens	AC127-050-A	Thorlabs
			Powell lens	LOCP-8.9R05-1.4	Laserline Optics
			Illumination laser	Cobolt 06-MLD 515nm	HÜBNER Photonics
Illumination path (configuration 2, with RS)		Extended Data Fig. 1f	Illumination objective lens	XLFLUOR4X/340	Olympus
	TL		Tube lens	ITL200	Thorlabs
	SL		Scan lens	CLS-SL	Thorlabs
	Mask		Optical mask	Customized mask	PhotomaskPORTAL
			Galvo mirror	Saturn 9B	ScannerMAX
	RL3		Relay lens	TL2X-SAP	Thorlabs
	RL4		Relay lens	AC127-075-A	Thorlabs
	RS		Resonant mirror scanner	RESSCAN	Sutter Instrument

			Powell lens	LOCP-8.9R05-1.4	Laserline Optics
			Illumination laser	Cobolt 06-MLD 515nm	HÜBNER Photonics
Microscope control and ata acquisition			LabVIEW software	LabVIEW 2021 32bit	National Instruments
			CompactRIO FPGA controller	cRIO-9038	National Instruments
			Analog output module	NI-9262	National Instruments
			Digital I/O module	NI-9401	National Instruments
			Data acquisition workstation	ThinkStation P350 SFF ×2	Lenovo
Sample holder and imaging chamber			Sample holder	Stereolithography (SLA) 3D-printed	
			Imaging chamber	3D-printed	
			Magnets	D101-N52	K&J Magnetics

Supplementary Table 3. DNA sequences (5′ to 3′) between the two Tol2 transposon arms in the Positron2-Kv plasmids for zebrafish embryo microinjections in this work

Constructs	DNA sequences (5′ to 3′)
2xUAS:Positron2-Kv	agcttttaatctcaaaaaacattaaatgaaatgcatacaaggttttatcctgctttagaactgtttgtatttaattatcaaactataagacagacaatctaatgccagtacacgctactcaaagttgtaaaacctcagatttaacttcagtagaagctgattctcaaaattgttagtgtcaagcctagctcttttggggctgaaaagcaatcctgcagtgctgaaaagcctctcacaggcagccgatgcgggaagaggtgtattagtcttgatagagaggctgcaaatagcaggaaacgtgagcagagactccctggtgtctgaaacacaggccagatgggccctcgacggtatcgataagaaaactagagattcttgtttaagcttgaggctgctgtcagggagctcatggacttcgagaattaaccctcactaaagggaatacgaagttatccggagtactgtcctccgacactagaggtcggagtactgtcctccgccatacgtagcagggtagactgtagagggtatataatggaacccatcgcgtctcagcctcactttgagctcctccacacgaattcgccatcatggctgacgtggaaaccgagaccggcatgattgcacagtggattgtctttgctattatggctgctgctgctattgcttttggagtggctgtgcactttcggccttcagagctgaagagcgcatactatatcaacattgccatctgcactatcgccgctaccgcttactatgcaatggccgtgaactaccaggacctgacaatgaatggtgaaaggcaggtggtctacgcaaagtatattgactgggtgctgaccacaccactgctcctgctcaacctcatcgtcatgaccaagatgggcggagtgatgatttcttgggtcatcggcgcagacattttcatgatcgtgtttggtattctgggcgccttcgaggatgaacacaagttcaaatgggtgtactttatcgctggatgtgtgatgcaggcagtcctgacatacgggatgtataacgccacttggaaagacgatctgaagaaaagccccgagtaccatagctcctatgtcagtctgctcgtcttcctgtcaatcctcttcgtgttttatcctgtcgtgtgggctttcgggtctggtagtggcgtgctgtccgtcgacaatgaggccattctcatgggaatcctggatgtgctcgctaagccactgtttggaatggggtgcctcattgcccatgagactatcttcaagatcggtactggctttccattcgacccccattatgtggaagtcctgggcgagcgcatgcactacgtcgatgttggtccgcgcgatggcacccctgtgctgttcctgcacggtaacccgacctcctcctacgtgtggcgcaacatcatcccgcatgttgcaccgacccatcgctgcattgctccagacctgatcggtatgggcaaatccgacaaaccagacctgggttatttcttcgacgaccacgtccgcttcatggatgccttcatcgaagccctgggtctggaagaggtcgtcctggtcattcacgactggggctccgctctgggtttccactgggccaagcgcaatccagagcgcgtcaaaggtattgcatttatggagttcatccgccctatcccgacctgggacgaatggccagaatttgcccgcgagaccttccaggccttccgcaccaccgacgtcggccgcaagctgatcatcgatcagaacgtttttatcgagggtacgctgccgatgggtgtcgtccgcccgctgactgaagtcgagatggaccattaccgcgagccgttcctgaatcctgttgaccgcgagccactgtggcgcttcccaaacgagctgccaatcgccggtgagccagcgaacatcgtcgcgctggtcgaagaatacatggactggctgcaccagtcccctgtcccgaagctgctgttctggggcaccccaggcgttctgatcccaccggccgaagccgctcgcctggccaaaagcctgcctaactgcaaggctgtggacatcggcccgggtctgaatctgctgcaagaagacaacccggacctgatcggcagcgagatcgcgcgctggctgtcgacgctcgagatttccggcgagccaaccactaagagcaggatcaccagcgagggcgagtacatcccccctggaccagatcgacatcaacgtgttctgctacgagaacgaggtgcaaagtcagcctatcctgaacacaaaggaaatggctccacagtctaagcctcccgaagagcttgagatgtccagtatgccaagtcccgtggctcccctccctgccaggactgaaggagtgattgacatgaggagtatgtcatctattgatagcttcatctcttgcgcaacagatttccccgaggctactcgattctaataagtttaaacggtggatcctggccgtcgttttactgtaccatcgatgatgatccagacatgataagatacattgatgagtttggacaaaccacaactagaatgcagtgaaaaaaatgctttatttgtgaaatttgtgatgctattgctttatttgtaaccattataagctgcaataaacaagttaacaacaacaattgcattcattttatgtttcaggttcagggggaggtgtgggaggttttttaaagcaagtaaaacctctacaaatgtggtatggctgattatgatcctctagatcagatct
3xUAS:Positron2-Kv	agcttttaatctcaaaaaacattaaatgaaatgcatacaaggttttatcctgctttagaactgtttgtatttaattatcaaactataagacagacaatctaatgccagtacacgctactcaaagttgtaaaacctcagatttaacttcagtagaagctgattctcaaaattgttagtgtcaagcctagctcttttggggctgaaaagcaatcctgcagtgctgaaaagcctctcacaggcagccgatgcgggaagaggtgtattagtcttgatagagaggctgcaaatagcaggaaacgtgagcagagactccctggtgtctgaaacacaggccagatgggccctcgacggtatcgataagaaaactagagattcttgtttaagcttgaggctgctgtcagggagctcatggacttcgagaattaaccctcactaaagggaatacgaagttatccggagtactgtcctccgacgcaaggtcggagtactgtcctccgacactagaggtcggagtactgtcctccgccatacgtagcagggtagactgtagagggtatataatggaacccatcgcgtctcagcctcactttgagctcctccacacgaattcgccatcatggctgacgtggaaaccgagaccggcatgattgcacagtggattgtctttgctattatggctgctgctgctattgcttttggagtggctgtgcactttcggccttcagagctgaagagcgcatactatatcaacattgccatctgcactatcgccgctaccgcttactatgcaatggccgtgaactaccaggacctgacaatgaatggtgaaaggcaggtggtctacgcaaagtatattgactgggtgctgaccacaccactgctcctgctcaacctcatcgtcatgaccaagatgggcggagtgatgatttcttgggtcatcggcgcagacattttcatgatcgtgtttggtattctgggcgccttcgaggatgaacacaagttcaaatgggtgtactttatcgctggatgtgtgatgcaggcagtcctgacatacgggatgtataacgccacttggaaagacgatctgaagaaaagccccgagtaccatagctcctatgtcagtctgctcgtcttcctgtcaatcctcttcgtgttttatcctgtcgtgtgggctttcgggtctggtagtggcgtgctgtccgtcgacaatgaggccattctcatgggaatcctggatgtgctcgctaagccactgtttggaatggggtgcctcattgcccatgagactatcttcaagatcggtactggctttccattcgacccccattatgtggaagtcctgggcgagcgcatgcactacgtcgatgttggtccgcgcgatggcacccctgtgctgttcctgcacggtaacccgacctcctcctacgtgtggcgcaacatcatcccgcatgttgcaccgacccatcgctgcattgctccagacctgatcggtatgggcaaatccgacaaaccagacctgggttatttcttcgacgaccacgtccgcttcatggatgccttcatcgaagccctgggtctggaagaggtcgtcctggtcattcacgactggggctccgctctgggtttccactgggccaagcgcaatccagagcgcgtcaaaggtattgcatttatggagttcatccgccctatcccgacctgggacgaatggccagaatttgcccgcgagaccttccaggccttccgcaccaccgacgtcggccgcaagctgatcatcgatcagaacgtttttatcgagggtacgctgccgatgggtgtcgtccgcccgctgactgaagtcgagatggaccattaccgcgagccgttcctgaatcctgttgaccgcgagccactgtggcgcttcccaaacgagctgccaatcgccggtgagccagcgaacatcgtcgcgctggtcgaagaatacatggactggctgcaccagtcccctgtcccgaagctgctgttctggggcaccccaggcgttctgatcccaccggccgaagccgctcgcctggccaaaagcctgcctaactgcaaggctgtggacatcggcccgggtctgaatctgctgcaagaagacaacccggacctgatcggcagcgagatcgcgcgctggctgtcgacgctcgagatttccggcgagccaaccactaagagcaggatcaccagcgagggcgagtacatcccccctggaccagatcgacatcaacgtgttctgctacgagaacgaggtgcaaagtcagcctatcctgaacacaaaggaaatggctccacagtctaagcctcccgaagagcttgagatgtccagtatgccaagtcccgtggctcccctccctgccaggactgaaggagtgattgacatgaggagtatgtcatctattgatagcttcatctcttgcgcaacagatttccccgaggctactcgattctaataagtttaaacggtggatcctggccgtcgttttactgtaccatcgatgatgatccagacatgataagatacattgatgagtttggacaaaccacaactagaatgcagtgaaaaaaatgctttatttgtgaaatttgtgatgctattgctttatttgtaaccattataagctgcaataaacaagttaacaacaacaattgcattcattttatgtttcaggttcagggggaggtgtgggaggttttttaaagcaagtaaaacctctacaaatgtggtatggctgattatgatcctctagatcagatct
