# Supplementary material for: Voltage imaging of neurons distributed across entire brains of larval zebrafish
Source: Nat Methods. 2026 Aug 14;23(9):1895–907. doi: 10.1038/s41592-026-03179-7 (PMC13541626; doi:10.1038/s41592-026-03179-7)
Supplement: Supplementary file 2 [file 41592_2026_3179_MOESM2_ESM.pdf]

nature portfolio

Corresponding author(s): Edward S. Boyden

Last updated by author(s): 4/8/2026

Reporting Summary

Nature Portfolio wishes to improve the reproducibility of the work that we publish. This form provides structure for consistency and transparency in reporting. For further information on Nature Portfolio policies, see our Editorial Policies and the Editorial Policy Checklist.

Statistics

For all statistical analyses, confirm that the following items are present in the figure legend, table legend, main text, or Methods section.

n/a	Confirmed	
☐	☒	The exact sample size (*n*) for each experimental group/condition, given as a discrete number and unit of measurement
☐	☒	A statement on whether measurements were taken from distinct samples or whether the same sample was measured repeatedly
☐	☒	The statistical test(s) used AND whether they are one- or two-sided<br>*Only common tests should be described solely by name; describe more complex techniques in the Methods section.*
☒	☐	A description of all covariates tested
☒	☐	A description of any assumptions or corrections, such as tests of normality and adjustment for multiple comparisons
☐	☒	A full description of the statistical parameters including central tendency (e.g. means) or other basic estimates (e.g. regression coefficient) AND variation (e.g. standard deviation) or associated estimates of uncertainty (e.g. confidence intervals)
☐	☒	For null hypothesis testing, the test statistic (e.g. *F*, *t*, *r*) with confidence intervals, effect sizes, degrees of freedom and *P* value noted<br>*Give P values as exact values whenever suitable.*
☒	☐	For Bayesian analysis, information on the choice of priors and Markov chain Monte Carlo settings
☒	☐	For hierarchical and complex designs, identification of the appropriate level for tests and full reporting of outcomes
☐	☒	Estimates of effect sizes (e.g. Cohen's *d*, Pearson's *r*), indicating how they were calculated

Our web collection on statistics for biologists contains articles on many of the points above.

Software and code

Policy information about availability of computer code

Data collection	For data acquisition, the microscope was controlled using custom LabVIEW software, a real-time FPGA device (NI cRIO-9038), and multiple digital and analog output modules (NI-9262, NI-9401). Raw images and videos were acquired using the commercial XIMEA CamTool software.
Data analysis	To analyze the data, raw images were initially reorganized and stitched into videos representing different sample layers using custom MATLAB and Python scripts. Next, neuronal regions of interest (ROIs) were identified using a custom-trained deep learning model based on Cellpose with post manual corrections. These sample layer videos and corresponding ROIs were then processed with the CAIMAN/VolPy software to extract neural activity, and the extracted activity along with ROI positions was further analyzed using custom MATLAB code.

For manuscripts utilizing custom algorithms or software that are central to the research but not yet described in published literature, software must be made available to editors and reviewers. We strongly encourage code deposition in a community repository (e.g. GitHub). See the Nature Portfolio guidelines for submitting code & software for further information.

Data

Policy information about availability of data

All manuscripts must include a data availability statement. This statement should provide the following information, where applicable:

- Accession codes, unique identifiers, or web links for publicly available datasets
- A description of any restrictions on data availability
- For clinical datasets or third party data, please ensure that the statement adheres to our policy

Raw light-sheet imaging data, analysis results, and other raw and processed data associated with this paper, are publicly available at https://doi.org/10.6019/S-BSST2153.

Human research participants

Policy information about studies involving human research participants and Sex and Gender in Research.

Reporting on sex and gender	No human participants were involved in the study
Population characteristics	No human participants were involved in the study
Recruitment	No human participants were involved in the study
Ethics oversight	No human participants were involved in the study

Note that full information on the approval of the study protocol must also be provided in the manuscript.

Field-specific reporting

Please select the one below that is the best fit for your research. If you are not sure, read the appropriate sections before making your selection.

☒ Life sciences ☐ Behavioural & social sciences ☐ Ecological, evolutionary & environmental sciences

For a reference copy of the document with all sections, see nature.com/documents/nr-reporting-summary-flat.pdf

Life sciences study design

All studies must disclose on these points even when the disclosure is negative.

Sample size	In this study, our primary goal was to demonstrate the potential of our new imaging technique rather than to draw definitive biological conclusions. Accordingly, we used a relatively small sample size. Specifically, for the visual stimulus experiments, we initially imaged eight zebrafish larvae (aged 5–6 days post fertilization) that expressed fluorescent voltage indicators. Each fish was subjected to two stimulation and imaging trials using identical settings. However, due to the large z-axis motion across trials in 3 fish and abnormal heart rate in 1 fish, we only analyzed the data from four zebrafish (designated Fish A, B, C, and D). Neural responses to visual stimulation and the occurrence of stimulation-independent burst events were subsequently analyzed across the two trials for these four subjects.
Data exclusions	Among the 8 imaged fish during the visual stimulus experiments, 3 fish had large z-axis motion between trials--hindering the alignment of the same neurons across trials--and 1 fish showed an unusually slow heart rate. Therefore, we excluded these four fish from further analysis. In addition, during the analysis of individual fish data, some of the neuron ROIs were contaminated by the stripe-shaped light-sheet illumination artifacts. We excluded the extracted neural traces from those ROIs in subsequent analyses following the VolPy processing.
Replication	Zebrafish were born, stained and screened, and imaged at different days. Good cell-membrane localized fluorescent signals were visible in all of the imaging experiments. The visual stimulus was repeated twice in two identical imaging trials for the four analyzed fish shown in this study. Consistent neural responses were recorded from both trials in Fish A, B, C, and D. During the visual stimulus experiment, stimulus-independent bursts repeated multiple times across two trials, showing consistent neural firing sequences in each fish.
Randomization	We screened the fluorescence of the larval zebrafish after staining and selected the ones with bright and pan-neuronal fluorescence for imaging. In analyzing the neural traces extracted by VolPy, the algorithms processed each neuron without making any assumptions. The results from the algorithms are independent of neuron IDs or the orders of neurons.
Blinding	Blinding was not relevant to this study. This study does not make any biological conclusions.

Reporting for specific materials, systems and methods

We require information from authors about some types of materials, experimental systems and methods used in many studies. Here, indicate whether each material, system or method listed is relevant to your study. If you are not sure if a list item applies to your research, read the appropriate section before selecting a response.

Materials & experimental systems

n/a	Involved in the study
☐	☒ Antibodies
☒	☐ Eukaryotic cell lines
☒	☐ Palaeontology and archaeology
☐	☒ Animals and other organisms
☒	☐ Clinical data
☒	☐ Dual use research of concern

Methods

n/a	Involved in the study
☒	☐ ChIP-seq
☒	☐ Flow cytometry
☒	☐ MRI-based neuroimaging

Antibodies

Antibodies used

1. γH2AX: GeneTex, cat# GTX127342; polyclonal rabbit; RRID: AB_2833105;
2. NeuN: Abcam, cat# ab177487; rabbit monoclonal clone EPR12763; RRID: AB_2532109;
3. Cleaved caspase-3: BD Pharmingen, cat# 559565; rabbit monoclonal clone C92-605; RRID: AB_397274;
4. Synaptophysin: Abcam, cat# ab32594; rabbit polyclonal; RRID: AB_778204;
5. Secondary antibody: Thermo Fisher Scientific (Invitrogen), Donkey anti-Rabbit IgG (H+L), Highly Cross-Adsorbed, Alexa Fluor™ Plus 647, cat# A32795TR; donkey polyclonal; RRID: AB_2866496;

Validation

1. γH2AX (GTX127342): Datasheet includes whole-mount zebrafish IHC images and protocol (Tris pH 9, 70 °C antigen retrieval), matching our retrieval conditions; also shows WB on zebrafish embryo extracts. RRID record available (AB_2833105).
2. NeuN (ab177487): Widely used rabbit monoclonal validated by Abcam for IHC-P, IHC-Fr, IF, WB, and Flow; RRID AB_2532109 lists broad cross-species use including zebrafish in the Antibody Registry profile.
3. Cleaved caspase-3 (BD 559565): Manufacturer describes specificity for the active (cleaved) form. The ZFIN antibody entry (https://zfin.org/ZDB-ATB-081107-1#summary) and published JoVE protocol (https://dx.doi.org/10.3791/51060) document whole-mount zebrafish IF with this antibody. RRID AB_397274.
4. Synaptophysin (ab32594): Abcam polyclonal used extensively for presynaptic labeling; RRID AB_778204. Zebrafish usage is documented in ZFIN (https://zfin.org/ZDB-ATB-161221-1).
5. Secondary (A32795TR): Highly cross-adsorbed donkey anti-rabbit Alexa Fluor Plus 647; manufacturer notes enhanced specificity and reduced cross-reactivity; RRID AB_2866496 is registered.

Animals and other research organisms

Policy information about studies involving animals; ARRIVE guidelines recommended for reporting animal research, and Sex and Gender in Research

Laboratory animals	Zebrafish: transgenic lines Tg(HuC:Gal4; 3xUAS:Positron2-Kv), Tg(HuC:Gal4; 2xUAS:Positron2-Kv), Tg(HuC:Gal4; UAS:Kaede), Tg(HuC:Gal4; 2×UAS:Positron2-Kv; flk:DsRed), and Tg(HuC:Positron2-Kv) at larval stages between 5 and 6 days post fertilization.
Wild animals	No wild animal was involved.
Reporting on sex	The study does not apply to one sex only.
Field-collected samples	This study did not include samples collected from the field.
Ethics oversight	All procedures related to zebrafish husbandry and handling were conducted in accordance with the US National Institutes of Health Guide for the Care and Use of Laboratory Animals and approved by the MIT Committee on Animal Care (protocol numbers: 1221-100-24 and 2411000756).

Note that full information on the approval of the study protocol must also be provided in the manuscript.
