# Supplementary material for: Voltage imaging of neurons distributed across entire brains of larval zebrafish
Source: Nat Methods. 2026 Aug 14;23(9):1895–907. doi: 10.1038/s41592-026-03179-7 (PMC13541626; doi:10.1038/s41592-026-03179-7)
Supplement: Supplementary file 3 [file 41592_2026_3179_MOESM3_ESM.pdf]

nature portfolio

Peer Review File

Voltage imaging of neurons distributed across entire brains of larval zebrafish

Corresponding Author: Ed Boyden

This file contains all editorial decision letters in order by version, followed by all author rebuttals in order by version.

A version of this paper was originally rejected for publication by Nature Methods, however that decision was reconsidered after appeal by the authors.

Version 0:

Decision Letter:

12th Mar 2024

Dear Ed,

Thank you for your patience. Your Article entitled "Imaging the voltage of neurons distributed across entire brains of larval zebrafish" has now been seen by two reviewers, whose comments are attached. While they find your work of potential interest, they have raised serious concerns which in our view are sufficiently important that they preclude publication of the work in Nature Methods, at least in its present form.

As you will see, the reviewers raise concerns about the image quality, missing methodological details as well as the limited number of fish imaged (which raises concerns about robustness of the approach).

Should further experimental data allow you to fully address these criticisms we would be willing to look at a revised manuscript (unless, of course, something similar has by then been accepted at Nature Methods or appeared elsewhere). This includes submission or publication of a portion of this work somewhere else. We hope you understand that until we have read the revised paper in its entirety we cannot promise that it will be sent back for peer-review.

Although we cannot publish your paper in its present form, it may be appropriate for another journal in the Nature Portfolio. If you wish to explore the journals and transfer your manuscript please use our manuscript transfer portal. You will not have to re-supply manuscript metadata and files, unless you wish to make modifications. For more information, please see our manuscript transfer FAQ page.

If you are interested in revising this manuscript for submission to Nature Methods in the future, please contact me to discuss your appeal before making any revisions. Otherwise, we hope that you find the reviewers' comments helpful when preparing your paper for submission elsewhere.

Best regards,
Nina

Nina Vogt, PhD
Senior Editor
Nature Methods

Reviewers' Comments:

Reviewer #1:
Remarks to the Author:
Achieving whole brain voltage imaging at cellular resolution would be highly valuable for understanding information flow

across brain regions at spike resolution. In comparison, most current calcium imaging efforts are limited by the slow kinetics of the sensor and imaging methods. Thus, I commend the authors for their efforts in working towards this goal.

Having said that, the current manuscript has not achieved the goal of whole-brain spike-resolution imaging at cellular resolution. The authors are recording 1/3 of the brain (i.e. not the majority of the brain) at 243 vps, which can potentially miss a significant number of spikes given the fast kinetics of the sensor. Setting aside the claim of whole brain voltage imaging, achieving voltage recording across a large portion of the brain would still be valuable. However, at the conceptual level, the authors' microscopy design closely follows existing designs for light sheet microscopy with remote focusing. The one major technological upgrade proposed is the use of a commercially available high-speed camera. Data analysis also uses existing methods and relies heavily on manual processing, and the quality of the data processing hasn't been sufficiently demonstrated. Thus, if this paper does not contribute significant original advances in hardware design, software design, or data processing, then the emphasis must be on the quality of the overall implementation, as the authors state that they have achieved a "microscope optimized for the imaging of the voltage of individual neurons distributed throughout the entire larval zebrafish brain". Here, I have several major concerns that would need to be addressed.

1. The biggest problem with the current implementation is the image quality of the microscope. The "stripe" artifacts are significantly worse than expected for most mature LSM implementations. As it stands, the authors have not demonstrated that 1) they fully understand the source of these artifacts, and 2) they can correctly remove these artifacts during data analysis. I would have serious concerns about building a complicated microscopy system with these artifacts, and how much to trust the data generated with this system. I'll elaborate in more detail on this issue:

1a. LSM systems do not generate stationary banding patterns if implemented correctly. I do not find the explanation that these are caused by pigment in the fish convincing. Given the brain images shown, the author must be using a pigment mutant (probably mitfa -/-, which should have been specified in the paper), or else much of the brain would be entirely occluded. These pigment mutants do not have pigment cells at the side of the head below the eyes. The stripes are also not occasional darker lines of occlusion in an otherwise uniformly bright sheet, which is what would be expected if they are due to pigment cells or occluding objects. Instead, they are intense alternating bands of light and dark, which suggests a potentially serious undiagnosed issue with the optical implementation, not the animal.
Some controls should be performed:
- Do these stripes exist when imaging a fluorescein bath? Or in a dense sample of fluorescent beads?
- Are the positions of the stripes consistent across samples?

1b. Similarly, I'm not convinced that the authors understand the source of the moving stripes, which can potentially contaminate real neural signals. In the supplementary movie shown (and in images in the figures), these moving stripes have two curious properties. One, they all appear to have a constant width across the entire brain (from left to right). Two, the stripe can either increase or decrease in brightness. Both properties are not expected due to scattering by blood flow, which 1) should interrupt the light sheet beam at different offsets from the side of the head, 2) cannot generate a suddenly brighter stripe of constant diameter across the brain (i.e. blood cells can only scatter light), 3) should occur at well-defined positions in the animal.
Again, some controls should be performed:
- Are these stripe artifacts observed in a post-fixation animal without blood flow? In fluoresceine or bead samples?
- Are the positions where the moving stripe occurs roughly consistent across animals? This should be the case if this is due to blood flow. Simply claiming (with no citations) that the circulatory system of animals is different across individuals is not sufficient. Age-matched sibling animals should be used for this control. There are also transgenic animals that label the circulatory system, which can be used to control for any minor differences in blood vessel layout.

1c. The high degree of artifacts in the images places a high burden on data processing and analysis. There are two concerns here – 1) with a high number of thin cell diameter stationary stripes, any drift in the sample would lead to changes in the "signal" over time, and 2) the moving stripes inherently resemble neural signals.
- There is no way to evaluate whether the authors have successfully compensated for both issues. One would need a ratiometric method (e.g. dual channel imaging) using a constant signal along with the positron signal to evaluate this.
- The claim that it's safe to remove synchronous signals on the order of 50 ms is odd, given that the authors then show traces in Fig. 3e (orange group) and Fig. 5c (bottom trace), which are clearly in the range of ~50 ms. Is this insufficient removal of contaminating stripe artifacts?
- The authors compensate for the static stripe pattern by multiplying with a constant factor per stripe, (line 425) - they claim this would not complicate spike detection. However, SNR across stripes of varying brightness can be different, which is not taken into account.
- It would be useful to have a visual of the spatial distribution of all the putative-neuron ROIs (3k out of starting # of ROIs of 25k) - to help ascertain the net effect of the artifacts. Is the distribution spatially even or left-side heavy (as could be deduced from lines 525-28)? It is especially important as sidedness is evoked later to suggest/corroborate UV processing in contralateral OT (line 594-95)
- Given the artifacts, the authors should include a negative control (e.g. GFP control) to determine how clean the baseline for activity measurements really is.

2. The excitation arm of the microscope is not sufficiently described. The sentence "the sample is excited by a laterally illuminated light sheet (515 nm wavelength) that was generated using a Powell lens and scanned using a galvo mirror (not shown, as these are used in the conventional way)" with no citations (!) is not a sufficient description for a publication, especially for a methods paper, and especially given significant quality issues associated the brain volumes shown in the manuscript (see point #1). Please provide the actual light path and configuration. It's not possible to fully evaluate a microscope with the entire excitation light path missing.

3. Two obvious alternatives to the strategies used in this manuscript are light field and SCAPE. Their relative merits to the proposed LSM are not sufficiently or correctly explained.
- Light field is not discussed in the introduction and has already claimed to achieve a similar level of optical performance (including previous publications by the Boyden lab) -- XLFM and SDXLFM have both previously claimed to image a similar number of cells across the entire zebrafish brain with volume rates of 100 Hz. A much more detailed discussion of the relative merits of these systems should be included in the introduction, to motivate the present study. Along the same lines, in what way are the experiments and analyses presented in this study not possible with existing LFM methods? This should be included in the Discussion section.
- The introduction of SCAPE is somewhat misleading. The original conception of SCAPE suffers from significant light loss. However, since the development of the refractive index change strategy and the commercial glass-tip objective lens, light loss is now only ~10%, which is significantly better than the 50% light loss in the LSM remote focusing strategy used in this study. The statement that the glass-tipped objective is not appropriate for large FOV imaging because only the center 450 um is diffraction limited is misleading. The pixel resolution achieved in this LSM implementation is ~730 nm, which is also not diffraction-limited. Optical resolution beyond the spatial resolution set by the camera sensor is empty resolution.

4. As mentioned earlier, the current manuscript has not achieved the goal of whole-brain spike-resolution imaging at cellular resolution. The authors are recording 1/3 of the brain (i.e. not the majority of the brain) at 243 vps, which can potentially miss a significant number of spikes given the fast kinetics of the sensor. Given that achieving voltage recording across a large portion of the brain is still valuable, there's no reason to overstate the case. Furthermore, statistics on how many spikes are estimated to be missing should be provided.

At present, I'll limit my comments to the major conceptual points above, though there are also some minor mistakes in the manuscripts. For example:
Line 128/129: "We found spatially clustered sub-group of neurons at the dorsal side…that fired ~70 ms prior to other neurons" It is the ventral side that fires earlier (see Fig 5d, green arrow vs magenta arrow).

Reviewer #2:
Remarks to the Author:
Wang et al. developed microscopy optimized for whole brain voltage imaging by using optimized remote focusing and camera system and succeeded in volumetric imaging of voltage changes at about 200 Hz from about 1/3 of the entire neurons in the zebrafish brain at the larval stage. Furthermore, they have identified populations of neurons that showed different response patterns to visual stimuli, presenting interesting aspects of the spatiotemporal dynamics of neuronal populations. This is a significant advancement in voltage imaging of neuronal populations, which is highly demanded and thus is very valuable for future research on neuronal dynamics from a wide area of the brain. However, some areas need improvements.

・A weak point of this study is that the authors presented data from only one fish. How would they validate the results? For example, is there any information indicating that there is no particular abnormality in the tissue or cells after recording?

・How densely distributed are the analyzed neurons (after subtracting the duplicated ROIs)? Providing the nearest neighbor distance would be preferable.

・Regarding the pulse-like artifacts, a previous study (Abdelfattah et al., 2019) avoided the shadowing effect by stopping the heart movement for a short term. Although this method has limitations, it would be nice to refer to this approach as one option.

・It is desirable to quantify the photobleaching of the recorded neuron populations.
Why did the authors set the duration of the voltage imaging to 35 sec? How long can the recording time be extended?

・line 605: According to the morphological information of the optic tectum, which layers in the retina would correspond to L1 and L2 indicated by the authors? For example, XYZ imaging of the recorded fish with a confocal microscope would allow precise observation of the layered structure of the retina and link it to the voltage information.

・If the authors established the 3XUAS:Positron2-Kv construct, its process should be described in the method section.

・In the current manuscript, the detailed design of the 3D-printed sample chamber was not provided. A schematic view of the chamber or a more detailed description is desirable.

・line 919: The authors mentioned "manual clustering analysis," but please explain how to analyze the data, including any criteria.

・The authors only showed a movie of stripe artifacts. The movie files of spontaneous activity and light-evoked responses also need to be provided.

・Figure 2d: It is better to show enlarged views of the burst firing patterns indicated by black arrowheads

・Figure 4h: Currently, the diagram is difficult to see. It is preferable to show the schematics more clearly.

・line 1238: The authors wrote, "The spike raster of the neurons outside the white dashed box is labeled using a light gray color," but plots in gray color seem not visible in Figure 5h.

・The authors often used expressions that are too casual. (eg. line 91 "say")

** For Nature Portfolio general information and news for authors, see http://npg.nature.com/authors.

Version 1:

Decision Letter:

19th Apr 2024

Dear Ed,

Thank you for your letter asking us to reconsider our decision on your Article, "Imaging the voltage of neurons distributed across entire brains of larval zebrafish". After careful consideration we have decided that we are willing to consider a version of your manuscript that is revised as outlined in your letter. Please note that we cannot promise sending the manuscript back to the reviewers at this point as we will need to assess how the concerns about image quality are addressed.

When revising your paper:

* include a point-by-point response to our referees and to any editorial suggestions

* please underline/highlight any additions to the text or areas with other significant changes to facilitate review of the revised manuscript

* address the points listed described below to conform to our open science requirements

* ensure it complies with our general format requirements as set out in our guide to authors at www.nature.com/naturemethods

* resubmit all the necessary files electronically by using the link below to access your home page

Link Redacted

Note: This URL links to your confidential home page and associated information about manuscripts you may have submitted, or that you are reviewing for us. If you wish to forward this email to co-authors, please delete the link to your homepage.

We hope to receive your revised paper within 2-3 months. If you cannot send it within this time, please let us know. In this event, we will still be happy to reconsider your paper at a later date so long as nothing similar has been accepted for publication at Nature Methods or published elsewhere.

OPEN SCIENCE REQUIREMENTS

REPORTING SUMMARY AND EDITORIAL POLICY CHECKLISTS
When revising your manuscript, please submit reporting summary and editorial policy checklists.

Reporting summary: https://www.nature.com/documents/nr-reporting-summary.zip
Editorial policy checklist: https://www.nature.com/documents/nr-editorial-policy-checklist.zip

If your paper includes custom software, we also ask you to complete a supplemental reporting summary.

Software supplement: https://www.nature.com/documents/nr-software-policy.pdf

Please submit these with your revised manuscript. They will be available to reviewers to aid in their evaluation if the paper is re-reviewed. If you have any questions about the checklist, please see http://www.nature.com/authors/policies/availability.html or contact me.

Please note that these forms are dynamic 'smart pdfs' and must therefore be downloaded and completed in Adobe Reader. We will then flatten them for ease of use by the reviewers. If you would like to reference the guidance text as you complete the template, please access these flattened versions at http://www.nature.com/authors/policies/availability.html.

Finally, please ensure that you retain unprocessed data and metadata files after publication, ideally archiving data in

perpetuity, as these may be requested during the peer review and production process or after publication if any issues arise.

DATA AVAILABILITY
Please include a “Data availability” subsection in the Online Methods. This section should inform readers about the availability of the data used to support the conclusions of your study, including accession codes to public repositories, references to source data that may be published alongside the paper, unique identifiers such as URLs to data repository entries, or data set DOIs, and any other statement about data availability. At a minimum, you should include the following statement: “The data that support the findings of this study are available from the corresponding author upon request”, describing which data is available upon request and mentioning any restrictions on availability. If DOIs are provided, please include these in the Reference list (authors, title, publisher (repository name), identifier, year). For more guidance on how to write this section please see:
http://www.nature.com/authors/policies/data/data-availability-statements-data-citations.pdf

CODE AVAILABILITY
Please include a “Code Availability” subsection in the Online Methods which details how your custom code is made available. Only in rare cases (where code is not central to the main conclusions of the paper) is the statement “available upon request” allowed (and reasons should be specified).

We request that you deposit code in a DOI-minting repository such as Zenodo, Gigantum or Code Ocean and cite the DOI in the Reference list. We also request that you use code versioning and provide a license.

For more information on our code sharing policy and requirements, please see:
https://www.nature.com/nature-research/editorial-policies/reporting-standards#availability-of-computer-code

MATERIALS AVAILABILITY
As a condition of publication in Nature Methods, authors are required to make unique materials promptly available to others without undue qualifications.

Authors reporting new chemical compounds must provide chemical structure, synthesis and characterization details. Authors reporting mutant strains and cell lines are strongly encouraged to use established public repositories.

More details about our materials availability policy can be found at https://www.nature.com/nature-portfolio/editorial-policies/reporting-standards#availability-of-materials

ORCID
Nature Methods is committed to improving transparency in authorship. As part of our efforts in this direction, we are now requesting that all authors identified as ‘corresponding author’ on published papers create and link their Open Researcher and Contributor Identifier (ORCID) with their account on the Manuscript Tracking System (MTS), prior to acceptance. This applies to primary research papers only. ORCID helps the scientific community achieve unambiguous attribution of all scholarly contributions. You can create and link your ORCID from the home page of the MTS by clicking on ‘Modify my Springer Nature account’. For more information please visit please visit www.springernature.com/orcid.

We look forward to hearing from you soon.

Best regards,
Nina

Nina Vogt, PhD
Senior Editor
Nature Methods

Version 2:

Decision Letter:

24th Jul 2025

Dear Ed,

Thank you for your letter detailing how you would respond to the reviewer concerns regarding your Article, "Imaging the voltage of neurons distributed across entire brains of larval zebrafish". We have decided to invite you to revise your manuscript as you have outlined, before we reach a final decision on publication. Please make sure that it is clear for every experiment if

and how stripe artifacts were addressed.

Please do not hesitate to contact me if you have any questions or would like to discuss these revisions further.

When revising your paper:

* include a point-by-point response to the reviewers and to any editorial suggestions

* please underline/highlight any additions to the text or areas with other significant changes to facilitate review of the revised manuscript

* address the points listed described below to conform to our open science requirements

* ensure it complies with our general format requirements as set out in our guide to authors at www.nature.com/naturemethods

* resubmit all the necessary files by using the link below to access your home page

Link Redacted

Note: This URL links to your confidential account and associated information about manuscripts you may have submitted, or that you are reviewing for us. If you wish to forward this email to co-authors, please delete this link.

We hope to receive your revised paper within four weeks. If you are substantially delayed, please let us know. In this event, we will still be happy to reconsider your paper at a later date so long as nothing similar has been accepted for publication at Nature Methods or published elsewhere.

OPEN SCIENCE REQUIREMENTS

REPORTING SUMMARY
When revising your manuscript, please update your reporting summary.

Reporting summary: https://www.nature.com/documents/nr-reporting-summary.zip

If your paper includes custom software, we also ask you to complete a supplemental reporting summary.

Software supplement: https://www.nature.com/documents/nr-software-policy.pdf

Please submit these with your revised manuscript. They will be available to reviewers to aid in their evaluation if the paper is re-reviewed. If you have any questions about the checklist, please see http://www.nature.com/authors/policies/availability.html or contact me.

Please note that these forms are dynamic 'smart pdfs' and must therefore be downloaded and completed in Adobe Reader. We will then flatten them for ease of use by the reviewers. If you would like to reference the guidance text as you complete the template, please access these flattened versions at http://www.nature.com/authors/policies/availability.html.

For any revision that includes light microscopy data, we ask our authors to please include a completed light microscopy reporting table [https://www.nature.com/documents/Light_microscopy_reporting_table.xlsx] to ensure the methods are described thoroughly. The table will be available to reviewers and ultimately published should the manuscript be accepted at the journal.

Finally, please ensure that you retain unprocessed data and metadata files after publication, ideally archiving data in perpetuity, as these may be requested during the peer review and production process or after publication if any issues arise.

EXTENDED DATA FIGURES
When re-submitting your manuscript, please ensure that any supplementary figures and tables that are crucial to the manuscript's conclusions are converted into Extended Data figures and tables to increase visibility of these data. Extended Data figures and tables are online-only (present in the online PDF and full-text HTML versions of the paper), peer-reviewed display items that provide essential background to the article but are not included in the main article due to space constraints. A maximum of ten Extended Data display items (figures and tables) is permitted.

DATA AVAILABILITY
Please include a "Data availability" subsection in the Online Methods. This section should inform readers about the availability of the data used to support the conclusions of your study, including accession codes to public repositories, references to source data that may be published alongside the paper, unique identifiers such as URLs to data repository entries, or data set

DOIs, and any other statement about data availability. At a minimum, you should include the following statement: “The data that support the findings of this study are available from the corresponding author upon request”, describing which data is available upon request and mentioning any restrictions on availability. If DOIs are provided, please include these in the Reference list (authors, title, publisher (repository name), identifier, year). For more guidance on how to write this section please see: http://www.nature.com/authors/policies/data/data-availability-statements-data-citations.pdf

For papers containing bioimaging data, we strongly recommend depositing the data to Bioimage Archive (https://www.ebi.ac.uk/bioimage-archive/). Associated accession codes and hyperlinks should be provided in the "Data Availability" section.

CODE AVAILABILITY
Please include a “Code Availability” subsection in the Online Methods which details how your custom code is made available. Only in rare cases (where code is not central to the main conclusions of the paper) is the statement “available upon request” allowed (and reasons should be specified).

We request that you deposit code in a DOI-minting repository such as Zenodo, Gigantum or Code Ocean and cite the DOI in the Reference list. We also request that you use code versioning and provide a license.

For more information on our code sharing policy and requirements, please see: https://www.nature.com/nature-research/editorial-policies/reporting-standards#availability-of-computer-code

MATERIALS AVAILABILITY
As a condition of publication in Nature Methods, authors are required to make unique materials promptly available to others without undue qualifications.

Authors reporting new chemical compounds must provide chemical structure, synthesis and characterization details. Authors reporting mutant strains and cell lines are strongly encouraged to use established public repositories.

More details about our materials availability policy can be found at https://www.nature.com/nature-portfolio/editorial-policies/reporting-standards#availability-of-materials

ORCID
Nature Methods is committed to improving transparency in authorship. As part of our efforts in this direction, we are now requesting that all authors identified as ‘corresponding author’ on published papers create and link their Open Researcher and Contributor Identifier (ORCID) with their account on the Manuscript Tracking System (MTS), prior to acceptance. This applies to primary research papers only. ORCID helps the scientific community achieve unambiguous attribution of all scholarly contributions. You can create and link your ORCID from the home page of the MTS by clicking on ‘Modify my Springer Nature account’. For more information please visit please visit www.springernature.com/orcid.

Please do not hesitate to contact me if you have any questions or would like to discuss these revisions further. We look forward to seeing the revised manuscript and thank you for the opportunity to consider your work.

Best regards,
Nina

Nina Vogt, PhD
Senior Editor
Nature Methods

Reviewers' Comments:

Reviewer #1 (Remarks to the Author):

The most significant improvement to the manuscript is the addition of the resonant scanners to remove the significant stripe artifacts associated with the initial submission (see Supplemental Figure 16). I commend the authors for making this necessary and critical addition to the particular system under consideration.
Given the clarifying comparison in Supplemental Fig16, extracting real spikes without the resonant scanner does not seem credible. As such, one would have expected the authors to update all result sections to datasets collected with the resonant scanner enabled. However, it’s unclear to me which figures in the revised manuscript are using the artifact-laden data from the

original submission and which ones are using the new system (Fig. 2a in the revised manuscript still clearly show the artifacts in question).
If the authors have now switched to the resonant scanner, then a number of supplemental figures related to artifact removal (e.g. Supplemental Figure 10, 11, 14, 15) are no longer necessary, significantly shortening the number of supplemental figures. Clarification of the above point is needed for further evaluation of the manuscript. If the data and analyses have not been updated to the version with the RS enabled, then the authors should do so and the manuscript can be evaluated based on the revised data/analyses. If the authors have already updated all figures following the addition of the RS scanner, please state this clearly.

Version 3:

Decision Letter:

Our ref: NMETH-A54806C

15th Dec 2025

Dear Ed,

Thank you for submitting your revised manuscript "Imaging the voltage of neurons distributed across entire brains of larval zebrafish" (NMETH-A54806C) and for your patience during the review process. The manuscript has now been seen by one of the original referees and their comments are below. The reviewers find that the paper has improved in revision, and therefore we'll be happy in principle to publish it in Nature Methods, pending minor revisions to satisfy the referees' final requests and to comply with our editorial and formatting guidelines.

Specifically, we ask you to moderate claims about whole-brain imaging throughout the mansucript, abstract and title.

We are now performing detailed checks on your paper and will send you a checklist detailing our editorial and formatting requirements within two weeks or so. Please do not upload the final materials and make any revisions until you receive this additional information from us.

TRANSPARENT PEER REVIEW
Nature Methods offers a transparent peer review option for new original research manuscripts. We encourage increased transparency in peer review by publishing the reviewer comments, author rebuttal letters and editorial decision letters if the authors agree. Such peer review material is made available as a supplementary peer review file. **Please state in the cover letter 'I wish to participate in transparent peer review' if you want to opt in, or 'I do not wish to participate in transparent peer review' if you don't.** Failure to state your preference will result in delays in accepting your manuscript for publication.

Please note: we allow redactions to authors' rebuttal and reviewer comments in the interest of confidentiality. If you are concerned about the release of confidential data, please let us know specifically what information you would like to have removed. Please note that we cannot incorporate redactions for any other reasons. Reviewer names will be published in the peer review files if the reviewer signed the comments to authors, or if reviewers explicitly agree to release their name. For more information, please refer to our FAQ page.

ORCID
IMPORTANT: Non-corresponding authors do not have to link their ORCIDs but are encouraged to do so. Please note that it will not be possible to add/modify ORCIDs at proof. Thus, please let your co-authors know that if they wish to have their ORCID added to the paper they must follow the procedure described in the following link prior to acceptance:
https://www.springernature.com/gp/researchers/orcid/orcid-for-nature-research

Author names using non-Roman characters
Nature Portfolio journals can support presentation of author names using non-Roman characters in the HTML version of the article. If you wish to, please include author names in parentheses after the Roman-character spelling; see example online here. Currently supported scripts are: Arabic, Chinese, Cyrillic, Devanagari, Greek, Hebrew, Hangul, Japanese and Persian. You will be asked to verify the rendering is correct at proof stage.

Thank you again for your interest in Nature Methods. Please do not hesitate to contact me if you have any questions. We will be in touch again soon.

Best regards,
Nina

Nina Vogt, PhD
Senior Editor
Nature Methods

Reviewer #1 (Remarks to the Author):

In summary, the authors present two implementations of light sheet microscopy for voltage imaging across the zebrafish brain. While the authors have made substantial and useful technological progress, both approaches are constrained by significant technical limitations.
The first implementation has less axial blurring (2.7 µm), but approximately 25% of ROIs are affected by moving stripe artifacts. These ROIs are excluded from analysis (“Our analyses were based on clean traces from unaffected ROIs”). While the authors invest considerable effort in computational corrections, they do not incorporate a second reference channel, which is a widely used solution in whole-brain imaging of worms.
The second implementation incorporates a resonant scanner but introduces further constraints, including a more limited axial range (120 µm) and greater axial blurring (6.1 µm), which increases susceptibility to motion-related artifacts.
Neither implementation achieves "whole brain" imaging. Notably, the analyses of responses to UV stimuli presented in the manuscript also do not require whole-brain imaging. Comparable conclusions could be obtained by sequentially recording a limited number of axial planes and assessing response latencies.
Overall, the manuscript provides a detailed and thorough characterization of both approaches. There is no need to over-claim about achieving “whole brain” imaging. As echoed by another reviewer, the frequent mention of “whole-brain” or “entire brain” imaging should be removed from the title and text. The method described achieves voltage recording of 1/6-1/4 of neurons across the brain, as stated in the manuscript: “The maximum putative neuron number in our data was thus 1/6 to 1/4 of the estimated neuron number (~78000) in a 7-dpf fish”.

Version 4:

Decision Letter:

29th Jun 2026

Dear Ed,

I am pleased to inform you that your Article, "Voltage imaging of neurons distributed across entire brains of larval zebrafish", has now been accepted for publication in Nature Methods. The received and accepted dates will be December 16th, 2023 and June 29th, 2026. This note is intended to let you know what to expect from us over the next month or so, and to let you know where to address any further questions.

Over the next few weeks, your paper will be copyedited to ensure that it conforms to Nature Methods style. Once your paper is typeset, you will receive an email with a link to choose the appropriate publishing options for your paper and our Author Services team will be in touch regarding any additional information that may be required. It is extremely important that you let us know now whether you will be difficult to contact over the next month. If this is the case, we ask that you send us the contact information (email, phone and fax) of someone who will be able to check the proofs and deal with any last-minute problems.

After the grant of rights is completed, you will receive a link to your electronic proof via email with a request to make any corrections within 48 hours. If, when you receive your proof, you cannot meet this deadline, please inform us at rjsproduction@springernature.com immediately.

Authors may need to take specific actions to achieve compliance with funder and institutional open access mandates.
If your research is supported by a funder that requires immediate open access (e.g. according to Plan S principles or the NIH public access policy) then you should select the gold OA route, and we will direct you to the compliant route where possible. Because authors warrant under our subscription licensing terms that they haven’t committed to licensing any version of their article under a licence inconsistent with the terms of our agreement – including the applicable embargo period – publication under the subscription model isn’t suitable for authors whose funders require no embargo.

If you have any questions about our publishing options, costs, Open Access requirements, or our legal forms, please contact ASJournals@springernature.com

If you have posted a preprint on any preprint server, please ensure that the preprint details are updated with a publication reference, including the DOI and a URL to the published version of the article on the journal website.

You may wish to make your media relations office aware of your accepted publication, in case they consider it appropriate to organize some internal or external publicity. Once your paper has been scheduled you will receive an email confirming the publication details. This is normally 3-4 working days in advance of publication. If you need additional notice of the date and time of publication, please let the production team know when you receive the proof of your article to ensure there is sufficient time to coordinate. Further information on our embargo policies can be found here:
https://www.nature.com/authors/policies/embargo.html

To assist our authors in disseminating their research to the broader community, our SharedIt initiative provides you with a unique shareable link that will allow anyone (with or without a subscription) to read the published article. Recipients of the link with a subscription will also be able to download and print the PDF.

As soon as your article is published, you will receive an automated email with your shareable link.

You can now use a single sign-on for all your accounts, view the status of all your manuscript submissions and reviews, access usage statistics for your published articles and download a record of your refereeing activity for the Nature journals.

Please note that you and any of your coauthors will be able to order reprints and single copies of the issue containing your article through Nature Portfolio's reprint website, which is located at http://www.nature.com/reprints/author-reprints.html. If there are any questions about reprints please send an email to author-reprints@nature.com and someone will assist you.

Please feel free to contact me if you have questions about any of these points.

Best regards,
Nina

Nina Vogt, PhD
Senior Editor
Nature Methods

** Visit the Springer Nature Editorial and Publishing website at www.springernature.com/editorial-and-publishing-jobs for more information about our career opportunities. If you have any questions please click here.**

Open Access This Peer Review File is licensed under a Creative Commons Attribution 4.0 International License, which permits use, sharing, adaptation, distribution and reproduction in any medium or format, as long as you give appropriate credit to the original author(s) and the source, provide a link to the Creative Commons license, and indicate if changes were made. In cases where reviewers are anonymous, credit should be given to 'Anonymous Referee' and the source.
The images or other third party material in this Peer Review File are included in the article's Creative Commons license, unless indicated otherwise in a credit line to the material. If material is not included in the article's Creative Commons license and your intended use is not permitted by statutory regulation or exceeds the permitted use, you will need to obtain permission directly from the copyright holder.
To view a copy of this license, visit https://creativecommons.org/licenses/by/4.0/

Reviewer #1:
Remarks to the Author:
Achieving whole brain voltage imaging at cellular resolution would be highly valuable for understanding information flow across brain regions at spike resolution. In comparison, most current calcium imaging efforts are limited by the slow kinetics of the sensor and imaging methods. Thus, I commend the authors for their efforts in working towards this goal.

Having said that, the current manuscript has not achieved the goal of whole-brain spike-resolution imaging at cellular resolution. The authors are recording 1/3 of the brain (i.e. not the majority of the brain) at 243 vps, which can potentially miss a significant number of spikes given the fast kinetics of the sensor.

We agree with the reviewer that our current method has not achieved whole-brain, spike-resolution imaging at cellular resolution. We had tried to make our language appropriately modest – e.g., using the phraseology “neurons distributed across entire brains” and so forth – **we will further work to make the language more modest**.

Can the number of neurons be improved? One main concern is that our fish line has some brain regions, where not all neurons are labeled (e.g., Fig. 2a, the dorsal side of the brain). To be honest, this is probably a problem with all zebrafish papers – none are probably fully pan-neuronal, and few papers check that every single neuron is expressing a given construct. **We will attempt to screen for fish with more pan-neuronal GEVI expression,** by performing more embryo injections, trying different promoters. But, the outcome is unpredictable - we will do our best, but these efforts may not succeed. In any case, improving fish line generation is not the main focus of our paper.

Regarding the possibility of missing spikes due to the low sampling rate, we will quantify the percentage of missed spikes by analyzing the neural voltage activity in a single z-plane video recorded at 6,000 Hz. **We will** down sample the 6000-Hz video by extracting a 200-Hz discrete frame series from the video, and **quantify how many spikes are missed in the down-sampled video** compared with the 6000-Hz video. Similarly, we can quantify the percentage of missed spikes at other sampling rates (e.g., 300 Hz, 500 Hz).

As a possible way to increase our microscope’s volume rate, **we will try running the cameras in an 8-bit mode**, which will enhance the frame rate by ~70%, but quadruple the readout noise. We will need to quantify how much the quadrupled readout noise will affect the signal-to-noise ratio (SNR) of the recorded neural traces, to determine if the 8-bit mode would be a valuable option, or whether it would incur corrupting levels of noise.

Setting aside the claim of whole brain voltage imaging, achieving voltage recording across a large portion of the brain would still be valuable. However, at the conceptual level, the authors’ microscopy design closely follows existing designs for light sheet microscopy with remote focusing. The one major technological upgrade proposed is the use of a commercially available

high-speed camera. Data analysis also uses existing methods and relies heavily on manual processing, and the quality of the data processing hasn't been sufficiently demonstrated.

In our current data analysis pipeline, the most time and labor consuming step is neuron ROI segmentation. We found that existing automatic ROI segmentation methods fall short of effectively identifying ROIs in our dataset. We hypothesize this inadequacy stems from discrepancies between the datasets used to train these learning-based methods, and the characteristics of our novel video data. To address this, **we plan to train a new neural network to segment our dataset**. Specifically, we will take the following approaches:

1. We will manually segment ROIs from our existing datasets from 4 different fish. **We will** use these segmentation results to repeat the current neural activity analysis for the 4 existing fish to **increase the number of animals (from N=1 to N>=4) in our experiments**. The segmented ROIs will also form the training dataset of the automatic segmentation network.

2. We will then use these 4 manually segmented fish to train a segmentation network based on a U-net architecture similar to the ones used in Cellpose (Pachitariu & Stringer, 2022)**.**

3. We will then evaluate the performance of the automatic segmentation method with the manually labeled ROIs. This includes spatial comparison of the ROI footprints. We will also compare the time series traces extracted from both methods, and evaluate them using the same analysis (used in Fig. 3, 4, 5) to see if our results generalize across fish.

4. Of course, we cannot be 100% sure that even after retraining, machine learning segmentation will work. **As a backup plan, we will develop non-AI segmentation methods, using traditional computer vision** customized to the specifics of our sample. Such a pipeline, instead of "detecting" neurons from the background, would segment the whole brain into neurons using a minimum-seeded watershed algorithm.

Thus, if this paper does not contribute significant original advances in hardware design, software design, or data processing, then the emphasis must be on the quality of the overall implementation, as the authors state that they have achieved a "microscope optimized for the imaging of the voltage of individual neurons distributed throughout the entire larval zebrafish brain". Here, I have several major concerns that would need to be addressed.

While we agree with the reviewer that at the conceptual level, our microscope follows the widespread idea of light-sheet microscopy with remote refocusing, there are several necessary innovations in the design and implementation of the microscope, that bring its capabilities to the level required for brain-wide voltage imaging. **We will emphasize these innovations more clearly.**

1. We have designed a new remote scanning module, and the software and pipeline for calibration and control. We combined a large shift, high resonant frequency piezo bender

scanner with a lightweight mirror for remote scanning, to enable 200-μm axial scanning at up to 300 Hz (or >100 μm at 500 Hz, both in practice). We also developed the hardware, software, and pipeline (see "Implementation of the microscope design" section in our manuscript) to allow accurately calibrating and controlling of the remote mirror movement. This is a nontrivial design.

2. Remote refocusing enhances the microscope's optical scanning rate, but exacerbates the mismatch between the microscope's optical scanning speed, and the camera's acquisition speed. To alleviate this mismatch and improve the imaging speed, we combined distributed planar imaging (Greer & Holy, 2019) with remote-refocusing light-sheet microscopy, efficiently doubling the volume rate for voltage imaging. This strategy could in principle be further scaled up to achieve a higher volume rate.

3. We developed a high-speed camera based on a newly available image sensor (Gpixel GSPRINT) and achieved the spatial resolution and SNR at the desired speed (7000 FPS). The camera's hardware design will be made open-source to the community. Additionally, the firmware and software for this camera incorporate widely adopted open-source designs commonly used within the neuroscience community (Firmware: Open-Ephys ONIX, Software: Bonsai). Together, this platform could significantly benefit future high-speed camera development and reduce the timeframe required to apply new commercial image sensors to scientific research.

1. The biggest problem with the current implementation is the image quality of the microscope. The "stripe" artifacts are significantly worse than expected for most mature LSM implementations. As it stands, the authors have not demonstrated that 1) they fully understand the source of these artifacts, and 2) they can correctly remove these artifacts during data analysis. I would have serious concerns about building a complicated microscopy system with these artifacts, and how much to trust the data generated with this system. I'll elaborate in more detail on this issue:

The "stripe" artifact in our images is a common issue for light-sheet imaging. This artifact and the possible strategies to alleviate it have been discussed in this review paper (Ricci et al., 2022). **We propose to fully characterize the stripe artifact**. **We may also explore possible ways to reduce the stripe artifact,** although given the persistence of this problem, a full solution may be beyond the scope of this paper. We are considering two optical strategies that we may attempt: (1) rapidly pivoting the light sheet in the illumination plane using an acousto-optic deflector (AOD) (Sancataldo et al., 2019), and (2) diffusing the light sheet in the illumination plane using a linear diffuser that is vibrated at high frequency (to eliminate laser speckles) (Taylor et al., 2018). Both strategies would try to reduce stripe artifacts at the imaging stage. The concern with these strategies is that, given the small ΔF/F of the voltage signals, only after implementation of these strategies would it be clear whether these strategies could reduce the stripe artifacts to a level that is negligible for real voltage signal traces.

In some previous work (Chhetri et al., 2015; Vladimirov et al., 2014) for whole-brain calcium imaging in larval zebrafish, a 2D plane of the sample was illuminated by scanning a Gaussian

beam across the 2D plane, so that during a single frame, each line on the plane was illuminated for only a tiny fraction (~1/180 if applied in our case) of the total recording time for that frame. In addition to this Gaussian beam scanning strategy, a 2D plane could be also illuminated using a real 2D light sheet (usually parallel light in the illumination plane), which continuously excites all fluorophores in the plane for the full recording time of a frame (Huisken et al. 2004; Pitrone et al. 2013). In terms of reducing stripe artifacts, the Gaussian beam strategy is preferable over the real light-sheet strategy, because a real 2D light sheet illuminates the sample using parallel light in the illumination plane, while a Gaussian beam illuminates the sample with a slightly convergent (~6° angle) light cone in the illumination plane. The convergent illumination can efficiently blur out the artifact stripe cast by a source object in a fan region. However, we cannot use a scanning Gaussian beam for illumination in our experiments. This is because the scanning Gaussian beam will significantly reduce (by a factor of ~180 in our case) the illumination time of GEVIs in each frame compared to using a real 2D light sheet. Consequently, the raw image intensity will drop significantly (by a factor of ~70 in our case) due to fluorescence saturation.

1a. LSM systems do not generate stationary banding patterns if implemented correctly. I do not find the explanation that these are caused by pigment in the fish convincing. Given the brain images shown, the author must be using a pigment mutant (probably mitfa -/-, which should have been specified in the paper), or else much of the brain would be entirely occluded. These pigment mutants do not have pigment cells at the side of the head below the eyes. The stripes are also not occasional darker lines of occlusion in an otherwise uniformly bright sheet, which is what would be expected if they are due to pigment cells or occluding objects. Instead, they are intense alternating bands of light and dark, which suggests a potentially serious undiagnosed issue with the optical implementation, not the animal.

While LSM systems do not generate stationary banding patterns by themselves, biological samples (e.g., larval zebrafish) do cause stripe artifacts in their raw images (Ricci et al. 2022; Taylor et al. 2018; Sancataldo et al. 2019). This is a common issue with LSM, and has been discussed in this review paper (Ricci et al., 2022). **We will emphasize this in revised text.**

We did use a pigment mutant fish (nacre) and will add that information in the manuscript. However, nacre fish still develop some opaque/scattering tissues on their skin at 5 dpf. In addition, while translucent, the larval zebrafish brain and the tissues around the brain are not optically uniform. The fish's skin is not flat, either. These non-uniform tissues can absorb, scatter, and refract the parallel light sheet, causing stripe-shaped artifact patterns in the raw images. We agree with the reviewer that simply using the word "pigment" here is not appropriate; **we will amend our text to make these descriptions more accurate and detailed.**

Regarding the reviewer's concern that the stripe artifact may come from the hardware instead of the animal, **we will perform the control experiments described in the following paragraphs, to test which case is true**.

Some controls should be performed:
- Do these stripes exist when imaging a fluorescein bath? Or in a dense sample of fluorescent beads?

To demonstrate if the stripe artifacts came from the optical setup or the animal, **we will image a 3D agarose sample** containing evenly distributed JF525 dye following the same protocols for 200-Hz whole zebrafish brain imaging. If the stripe artifacts still occur, we will diagnose their potential causes in the excitation light path, and fix the issue. Otherwise, such results would suggest the observed stripe artifacts were from the animal's biology.

- Are the positions of the stripes consistent across samples?

We will compare the positions of the stripes in agarose samples, live fish samples, and fixed fish samples (see details below) to see how these stripes' locations vary across samples. In addition, we will also image stripe artifacts in the same fish when the fish is placed at different lateral locations in the field-of-view. Specifically, we will translate the fish along the caudal-rostral axis (perpendicular to the stripes) to see if the stripe artifacts stay at the same locations in the fish brain. This should be the case if the artifacts are from the sample; otherwise it suggests the stripe artifacts could be associated with the nonuniformity of the light sheet, and we would locate the artifact sources in our hardware and solve the problem.

1b. Similarly, I'm not convinced that the authors understand the source of the moving stripes, which can potentially contaminate real neural signals. In the supplementary movie shown (and in images in the figures), these moving stripes have two curious properties. One, they all appear to have a constant width across the entire brain (from left to right). Two, the stripe can either increase or decrease in brightness. Both properties are not expected due to scattering by blood flow, which 1) should interrupt the light sheet beam at different offsets from the side of the head, 2) cannot generate a suddenly brighter stripe of constant diameter across the brain (i.e. blood cells can only scatter light), 3) should occur at well-defined positions in the animal.
Again, some controls should be performed:

We agree with the reviewer that we need to understand the source of the moving stripes to reduce or compensate for these artifacts. **We will perform additional experiments to show where the moving stripes were from, and try both hardware and computational strategies to reduce them.**

Regarding the three points that the reviewer made about the blood flow explanation, 1) we did observe the moving stripes originating at different offsets from the left side of the brain (the side where the light sheet entered the brain). Beside the stripes originating from the left side, some moving stripes originated within the brain (Fig. S6, clusters 7, 18, 22, 29, 32, 33, 34, green arrows), 2) blood cells can not only scatter light, but also refract light like microlenses (Miccio et al., 2015). With this lens-like property, blood cells in the fish brain can focus or diverge parallel illumination light to produce both bright and dark stripe patterns. 3) We did see consistency of the moving stripe distribution across sibling zebrafish brains at the brain region level.

We will conduct additional experiments (as proposed below) and show more results to elucidate the source of the moving stripe artifacts in a revised paper.

- Are these stripe artifacts observed in a post-fixation animal without blood flow? In fluoresceine or bead samples?

We will image the brain of a PFA-fixed transgenic Positron2-Kv larval zebrafish that we used for voltage imaging at 200 Hz to examine if there is any moving stripe artifact in the video. Additionally, **we may try to stop the zebrafish heart** (thus stopping blood flow) temporarily during imaging using 2,3-butanedione monoxime following the protocols in a previous study (Banjo et al., 2013). It is hard to predict if this method will work for fish at the age we are investigating (5 dpf vs 3.5 dpf in that paper), but if it is successful, we would be able to examine if the moving stripes disappear after the fish's heart is stopped. Reviewer 2 also referred to a paper (Abdelfattah et al., 2019) where the authors reduced the moving stripe artifacts by stopping the fish heart with forceps. But our committee on animal care is unlikely to let us do that, it seems.

- Are the positions where the moving stripe occurs roughly consistent across animals? This should be the case if this is due to blood flow. Simply claiming (with no citations) that the circulatory system of animals is different across individuals is not sufficient. Age-matched sibling animals should be used for this control. There are also transgenic animals that label the circulatory system, which can be used to control for any minor differences in blood vessel layout.

We will map the spatial locations of the moving stripes in the brains of age-matched sibling zebrafish that were imaged on the same day, and examine the consistency of the moving stripes' spatial locations across fish. To correlate the moving artifacts with the blood flow, **we will cross our transgenic GEVI fish with a fish line with vascular labeling (e.g., Tg(flk:DsRed), Tg(fli:EGFP))** to generate zebrafish larvae with both GEVI and blood vessel labeling. We will image both the moving stripe artifacts and the blood vessel layout in the same fish brain. We will compare the spatial positions of the moving stripe artifacts with the blood vessel layout in the fish brain.

1c. The high degree of artifacts in the images places a high burden on data processing and analysis. There are two concerns here – 1) with a high number of thin cell diameter stationary stripes, any drift in the sample would lead to changes in the "signal" over time, and 2) the moving stripes inherently resemble neural signals.
- There is no way to evaluate whether the authors have successfully compensated for both issues. One would need a ratiometric method (e.g. dual channel imaging) using a constant signal along with the positron signal to evaluate this.

Regarding concern 1, to eliminate the temporal artifacts caused by sample drift, we already paralyzed the fish during imaging and ran motion correction on our raw videos before extracting

the temporal traces. **We will calculate how much change in the "signal" over time can be caused by sample drift in our experiments**, by measuring the drift amplitude and the intensity differences of neighboring bright and dark stripes. **We will take additional measures (e.g., using higher concentration agarose for embedding) to further stabilize the sample during imaging.** On the other hand, we would like to note that even if the sample drift is large, as long as the stripe artifacts stay at the same locations in the brain (assuming the stripes are from the sample) and the motion correction is appropriately applied, these drift-induced changes in the "signal" over time will be compensated.

Regarding concern 2, we aim to address the artifact issue earlier on in the image formation and analysis pipeline. As we already mentioned, we will give attempts to optical strategies that might address the artifacts at the recording stage. **We also will attempt to develop algorithms to detect and mask the moving stripes in the raw videos** so that we could remove the entire affected regions before segmenting the neuron ROIs. However if these do not work, **we may explore ratiometric methods**. The reason that we are reluctant to try the ratiometric methods in the first place is that the ratiometric approach may not be able to efficiently correct the GEVI baseline intensity fluctuation caused by the moving artifacts. This is because the intensity of the reference fluorophore may not scale proportionally to the fluctuations of the GEVI intensity, due to fluorescence saturation of either fluorophore at high-intensity excitation. We estimate that in our experiments, the GEVI reached approximately 40% of its maximum fluorescence intensity. At this level, the GEVI's baseline intensity does not change proportionally to the excitation intensity. Still, we will keep the ratiometric approach as a backup plan in case the stripe artifacts cannot be addressed using other approaches. Nevertheless, we would like to note that the ratiometric approach is not an ideal solution to correct moving artifacts, for several additional reasons: 1) First, crosstalk of the reference fluorescence and the GEVI fluorescence could occur due to emission spectrum overlap. It is difficult to find a reference fluorophore that can be efficiently excited with the same excitation light as for the GEVI fluorophore, while having a non-overlapping emission spectrum. Alternatively, the overlapped part of the spectrum can be filtered out using emission filters, leading to undesired light loss. 2) Second, dual channel imaging requires doubling the number of cameras, increasing the cost, the complexity of the hardware, and the amount of data to analyze. 3) Third, the reference fluorophore will occupy a precious color channel that could otherwise be used for other applications, such as optogenetics or fluorescence indicators of other neural signals (e.g., neurotransmitters). If we try the ratiometric approach, we will use it purely for the purpose of evaluating our stripe removal pipeline, instead of as a strategy to compensate for the moving artifacts.

- The claim that it's safe to remove synchronous signals on the order of 50 ms is odd, given that the authors then show traces in Fig. 3e (orange group) and Fig. 5c (bottom trace), which are clearly in the range of ~50 ms. Is this insufficient removal of contaminating stripe artifacts?

The "synchronous signals on the order of 50 ms" criteria alone is not sufficient to identify ROIs contaminated by moving stripe artifacts. ROIs must also coincide with the moving stripes' locations to qualify for removal. The spatial distributions of the orange group ROIs in Fig. 3e and the bottom trace ROI in Fig. 5c did not align with a stripe artifact, nor did we find a group of

ROIs that had a stripe shaped spatial distribution while exhibiting synchronous signals to these ROIs. Therefore we did not exclude these ROIs and their traces in our analysis. **We will clarify this in the text.**

- The authors compensate for the static stripe pattern by multiplying with a constant factor per stripe, (line 425) - they claim this would not complicate spike detection. However, SNR across stripes of varying brightness can be different, which is not taken into account.

Sorry for the confusion associated with this sentence. We did not multiply a constant factor for each extracted trace to compensate for the static stripe patterns. We meant that having this static stripe pattern in our raw images was akin to scaling the traces that would have been extracted without stripe artifacts, by constant factors. We agree with the reviewer that the noise and SNR difference cannot be neglected here. Dark stripes could decrease the SNR of neural traces, and reduce spike detection accuracy. **We will modify this sentence in our manuscript.**

- It would be useful to have a visual of the spatial distribution of all the putative-neuron ROIs (3k out of starting # of ROIs of 25k) - to help ascertain the net effect of the artifacts. Is the distribution spatially even or left-side heavy (as could be deduced from lines 525-28)? It is especially important as sidedness is evoked later to suggest/corroborate UV processing in contralateral OT (line 594-95)

We will include a figure to show the spatial distribution of all the putative-neuron ROIs in the fish brain. **We will analyze the distribution** of the putative neuron ROIs in different regions of the brain, and take any difference into consideration in our analysis.

- Given the artifacts, the authors should include a negative control (e.g. GFP control) to determine how clean the baseline for activity measurements really is.

We will image a pan-neuronal RFP-labeled larval zebrafish brain (Tg(HuC:Gal4; UAS:RFP)) at 200 Hz and quantify how clean the baseline for activity measurements is. We will use RFP instead of GFP because RFP will allow use of the same excitation laser and emission filter as for the Positron2-Kv_{JF525} voltage indicator, key for mimicking the voltage imaging experiments.

2. The excitation arm of the microscope is not sufficiently described. The sentence “the sample is excited by a laterally illuminated light sheet (515 nm wavelength) that was generated using a Powell lens and scanned using a galvo mirror (not shown, as these are used in the conventional way)” with no citations (!) is not a sufficient description for a publication, especially for a methods paper, and especially given significant quality issues associated the brain volumes shown in the manuscript (see point #1). Please provide the actual light path and configuration. It’s not possible to fully evaluate a microscope with the entire excitation light path missing.

We will include a full diagram of the entire excitation light path and detailed description of the excitation arm design and construction in our manuscript.

3. Two obvious alternatives to the strategies used in this manuscript are light field and SCAPE. Their relative merits to the proposed LSM are not sufficiently or correctly explained.
- Light field is not discussed in the introduction and has already claimed to achieve a similar level of optical performance (including previous publications by the Boyden lab) -- XLFM and SDXLFM have both previously claimed to image a similar number of cells across the entire zebrafish brain with volume rates of 100 Hz. A much more detailed discussion of the relative merits of these systems should be included in the introduction, to motivate the present study. Along the same lines, in what way are the experiments and analyses presented in this study not possible with existing LFM methods? This should be included in the Discussion section.

We will add a section in our manuscript to discuss in detail the potentials and limitations of light-field microscopy (LFM) setups for voltage imaging, and their relative merits vs. those of our setup. In brief, LFM will be suitable for imaging the voltage of sparsely labeled neurons in a large volume. However, it will suffer from inherently low SNR when imaging densely labeled samples, such as the pan-neuronally GEVI labeled larval zebrafish brain. The low SNR is caused by increased shot noise, which stems from the mixing of fluorescence signals of different sample z-planes in raw light-field images. This increased shot noise is inherent and cannot be compensated for using SDXLFM. To image the entire larval zebrafish brain with dense labeling, we estimate that SDXLFM will have ~4× higher shot noise compared to our current light-sheet microscope.

- The introduction of SCAPE is somewhat misleading. The original conception of SCAPE suffers from significant light loss. However, since the development of the refractive index change strategy and the commercial glass-tip objective lens, light loss is now only ~10%, which is significantly better than the 50% light loss in the LSM remote focusing strategy used in this study. The statement that the glass-tipped objective is not appropriate for large FOV imaging because only the center 450 um is diffraction limited is misleading. The pixel resolution achieved in this LSM implementation is ~730 nm, which is also not diffraction-limited. Optical resolution beyond the spatial resolution set by the camera sensor is empty resolution.

It is true that our current setup loses 50% light at the polarized beam splitter, resulting in a light efficiency ~40% lower than SCAPE with the glass-tipped objective (the "Snouty" v2). As we mentioned in our manuscript, the lost 50% light could, in principle, be recovered by adding a second remote scanning module (i.e., a remote objective, a piezo bender scanner, and a remote mirror). We did not install this module because we did not find the image brightness to be limiting, in our experiments. Of course, doubled light efficiency would support higher SNR, reduced excitation power, and slower photobleaching. Therefore, **we may try to add a second remote scanning module to our current setup**. That said, we agree with the reviewer's point that the glass-tipped objective would be appropriate for imaging a FOV beyond the center 450 μm when the system resolution is bounded by the pixel size instead of diffraction. **We will update that statement in our manuscript.** Indeed, theoretically, SCAPE could be updated to

image the whole larval zebrafish brain at the same speed as our setup, with additions such as the same dual-camera system. Indeed, if the hardware is sufficiently optimized, perhaps any technology is capable of any kind of imaging, and SCAPE would be a leading candidate to be updated for voltage imaging setups of the future. We chose our current configuration over SCAPE because our configuration theoretically promised less aberration, and samples the fish brain in a way closer to conventional LSMs (Ahrens et al. 2013; Vladimirov et al. 2014; Dunn et al. 2016; Mu et al. 2019; Chen et al. 2018). But, as noted, after optimization, SCAPE could be adapted for imaging voltage activity across whole zebrafish brain in the future. **We will add text to the discussion in the manuscript to illustrate these principles**.

4. As mentioned earlier, the current manuscript has not achieved the goal of whole-brain spike-resolution imaging at cellular resolution. The authors are recording 1/3 of the brain (i.e. not the majority of the brain) at 243 vps, which can potentially miss a significant number of spikes given the fast kinetics of the sensor. Given that achieving voltage recording across a large portion of the brain is still valuable, there's no reason to overstate the case. Furthermore, statistics on how many spikes are estimated to be missing should be provided.

We will quantify the percentage of missed spikes by analyzing the neural voltage activity in a single z-plane video recorded at 6,000 Hz. We will downsample the 6000-Hz video by extracting a 200-Hz discrete frame series from the video, and quantify how many spikes are missed in the downsampled video compared with the 6000-Hz video. **We will amend the manuscript to avoid any overinterpretation of claims**.

At present, I'll limit my comments to the major conceptual points above, though there are also some minor mistakes in the manuscripts. For example:
Line 128/129: "We found spatially clustered sub-group of neurons at the dorsal side…that fired ~70 ms prior to other neurons"
It is the ventral side that fires earlier (see Fig 5d, green arrow vs magenta arrow).

We will correct this typo in our manuscript.

Reviewer #2:
Remarks to the Author:
Wang et al. developed microscopy optimized for whole brain voltage imaging by using optimized remote focusing and camera system and succeeded in volumetric imaging of voltage changes at about 200 Hz from about 1/3 of the entire neurons in the zebrafish brain at the larval stage. Furthermore, they have identified populations of neurons that showed different response patterns to visual stimuli, presenting interesting aspects of the spatiotemporal dynamics of neuronal populations. This is a significant advancement in voltage imaging of neuronal populations, which is highly demanded and thus is very valuable for future research on neuronal dynamics from a wide area of the brain. However, some areas need improvements.

· *A weak point of this study is that the authors presented data from only one fish. How would they validate the results? For example, is there any information indicating that there is no particular abnormality in the tissue or cells after recording?*

We actually already acquired imaging datasets from multiple fish. But, at time of submission, we had only analyzed the data from one fish. The most time-consuming step in our current data analysis pipeline is manual ROI segmentation. We used manual segmentation because we found that existing automatic ROI segmentation methods fell short of effectively identifying ROIs in our dataset. We hypothesize that this inadequacy stems from discrepancies between the datasets used to train these learning-based methods, and the characteristics of our video data. To address this, **we will train a new neural network to segment our dataset**. See the response to reviewer #1, second comment, for details.

We understand the reviewer's concern about the potential abnormalities induced by imaging. In our experiments, the excitation power for the fish brains was ~10mW at 515 nm wavelength. This power is much higher than the power (0.3mW) used in previous whole-brain calcium imaging experiments (Ahrens et al. 2013), opening questions about thermo- and photo-toxicity and damage to tissues and cells. As a way to reduce the negative effects of the high excitation power, we used an opaque optical mask to block the illumination to the fish's eyes, which have high absorbance of light due to the pigment cells of the retina. At the visual level, we examined the fish under a 5X stereoscope before and after imaging (usually containing three 35-s imaging trials). We did not see any visual abnormality in post-imaging fish. Their hearts beat normally in the same way as before recording. Neither color nor transparency change was observed. The fish could swim again after they recovered from paralysis. **We will acquire stereoscope pictures of fish before and after recording** and include the pictures for comparison in our manuscript. Still, it is possible that the abnormality can occur at other levels that are not visually evident under a stereoscope, e.g., microscale and cellular structure changes, or molecular alterations. **We will provide data to explore whether, at least at the level of standard observables of interest to the community,** we do not see negative effects: such examples could be:

a. continuous quantification of the morphology and activity of cells during imaging. Our data already capture that, and we have not yet seen standard signs of "damage" (e.g. cell blebbing or cell rounding, epileptic-like activity, or other obvious abnormal changes in the statistics of firing).

b. higher resolution anatomical imaging of the fish (eg. 40x confocal) before and after imaging.

c. behavioral (e.g. swimming, or more advanced stereotypic behavioral assays) and physiological observation (heart rate) of the fish before and after imaging.

d. (Tosheva et al. 2020) has a great summary of additional methods for cell-imaging, including e.g. apoptotic markers. We will investigate if and which of them could be translated to our samples in meaningful ways.

- *How densely distributed are the analyzed neurons (after subtracting the duplicated ROIs)? Providing the nearest neighbor distance would be preferable.*

We will include a figure in our manuscript to show the spatial distribution of all the putative neuron ROIs in the fish brain. We will calculate and provide the average nearest neighbor distance of neurons.

- *Regarding the pulse-like artifacts, a previous study (Abdelfattah et al., 2019) avoided the shadowing effect by stopping the heart movement for a short term. Although this method has limitations, it would be nice to refer to this approach as one option.*

Although stopping the fish's heart using forceps is currently not in our animal protocol, and we are concerned it is unlikely to pass MIT's animal care committee, **we may attempt to stop the zebrafish heart using a drug** (see reviewer #1, comment #9, above).

- *It is desirable to quantify the photobleaching of the recorded neuron populations. Why did the authors set the duration of the voltage imaging to 35 sec? How long can the recording time be extended?*

We will record the photobleaching curve of GEVIs in our experiments, even though photobleaching is not the limiting factor in our imaging experiments. During a 35-s recording, the GEVI's fluorescence intensity decreased by only ~10%. We can further lower the photobleaching rate using more photostable dyes for the GEVI, such as JF549 and JF585 (Abdelfattah et al. 2019), which are 3.9╳ and 7.9╳ more photostable than our current JF525 dye, respectively. The 35s duration of each imaging trial was arbitrary to some extent. We set the recording time to 35s to allow for >10s recording time both before and after a 10s visual stimulus. But, given that the datasets for a 35s recording are already very large (~250 GB per recording), we haven't really tried to push our recording duration to its true limit. During our recording, the data was streamed to SSD storage in real time. Therefore, in theory the maximum recording time is only limited by SSD storage size. However, in practice we noticed that our cameras occasionally dropped frames near the end of our 35s recordings. **We will examine this frame dropping issue further.** It is too early now to tell if this issue can be easily fixed as its source is still unclear. **We will perform a rigorous test of the maximum recording duration and include the results in the manuscript.**

▪ *line 605: According to the morphological information of the optic tectum, which layers in the retina would correspond to L1 and L2 indicated by the authors? For example, XYZ imaging of the recorded fish with a confocal microscope would allow precise observation of the layered structure of the retina and link it to the voltage information.*

We will image the fish's optical tectum under a confocal microscope immediately after voltage recording, and compare the layered structures seen, with our observations and hypotheses.

▪ *If the authors established the 3XUAS:Positron2-Kv construct, its process should be described in the method section.*

We will add details of this process, along with our experiences when making this fish line in our manuscript. In fact, we are actively screening more Positron2-Kv fish for more pan-neuronal expression. **We may also try different new GEVIs** and various expression strategies to improve the labeling and SNR of GEVIs in zebrafish.

▪ *In the current manuscript, the detailed design of the 3D-printed sample chamber was not provided. A schematic view of the chamber or a more detailed description is desirable.*

We will add a schematic view of the chamber and the fish holder and more detailed descriptions in our manuscript. **We will also share the CAD files** of the chamber and the holder for 3D printing in the supplementary materials.

▪ *line 919: The authors mentioned "manual clustering analysis," but please explain how to analyze the data, including any criteria.*

We will add more detailed explanations of this analysis step and describe the criteria we used.

▪ *The authors only showed a movie of stripe artifacts. The movie files of spontaneous activity and light-evoked responses also need to be provided.*

We will share these raw videos, **along with other raw data**, in a publicly accessible website link.

▪ *Figure 2d: It is better to show enlarged views of the burst firing patterns indicated by black arrowheads*

We will include the enlarged views of the burst firing pattern in the figure accordingly.

▪ *Figure 4h: Currently, the diagram is difficult to see. It is preferable to show the schematics more clearly.*

We will modify this figure to make the schematics more visible accordingly.

▪ *line 1238: The authors wrote, "The spike raster of the neurons outside the white dashed box is labeled using a light gray color," but plots in gray color seem not visible in Figure 5h.*

We will use a more visible color to label these neurons.

▪ *The authors often used expressions that are too casual. (eg. line 91 "say")*

We will modify our language accordingly.

REFERENCES

Abdelfattah, A. S., Kawashima, T., Singh, A., Novak, O., Liu, H., Shuai, Y., Huang, Y.-C., Campagnola, L., Seeman, S. C., Yu, J., Zheng, J., Grimm, J. B., Patel, R., Friedrich, J., Mensh, B. D., Paninski, L., Macklin, J. J., Murphy, G. J., Podgorski, K., … Schreiter, E. R. (2019). Bright and photostable chemigenetic indicators for extended in vivo voltage imaging. *Science*, *365*(6454), 699–704.

Banjo, T., Grajcarek, J., Yoshino, D., Osada, H., Miyasaka, K. Y., Kida, Y. S., Ueki, Y., Nagayama, K., Kawakami, K., Matsumoto, T., Sato, M., & Ogura, T. (2013). Haemodynamically dependent valvulogenesis of zebrafish heart is mediated by flow-dependent expression of miR-21. *Nature Communications*, *4*, 1978.

Chhetri, R. K., Amat, F., Wan, Y., Höckendorf, B., Lemon, W. C., & Keller, P. J. (2015). Whole-animal functional and developmental imaging with isotropic spatial resolution. *Nature*

Methods, *12*(12), 1171–1178.

Greer, C. J., & Holy, T. E. (2019). Fast objective coupled planar illumination microscopy. *Nature Communications*, *10*(1), 4483.

Miccio, L., Memmolo, P., Merola, F., Netti, P. A., & Ferraro, P. (2015). Red blood cell as an adaptive optofluidic microlens. *Nature Communications*, *6*, 6502.

Pachitariu, M., & Stringer, C. (2022). Cellpose 2.0: how to train your own model. *Nature Methods*, *19*(12), 1634–1641.

Ricci, P., Gavryusev, V., Müllenbroich, C., Turrini, L., de Vito, G., Silvestri, L., Sancataldo, G., & Pavone, F. S. (2022). Removing striping artifacts in light-sheet fluorescence microscopy: a review. *Progress in Biophysics and Molecular Biology*, *168*, 52–65.

Sancataldo, G., Gavryusev, V., de Vito, G., Turrini, L., Locatelli, M., Fornetto, C., Tiso, N., Vanzi, F., Silvestri, L., & Pavone, F. S. (2019). Flexible multi-beam light-sheet fluorescence microscope for live imaging without striping artifacts. *Frontiers in Neuroanatomy*, *13*, 7.

Taylor, M. A., Vanwalleghem, G. C., Favre-Bulle, I. A., & Scott, E. K. (2018). Diffuse light-sheet microscopy for stripe-free calcium imaging of neural populations. *Journal of Biophotonics*, *11*(12), e201800088.

Vladimirov, N., Mu, Y., Kawashima, T., Bennett, D. V., Yang, C.-T., Looger, L. L., Keller, P. J., Freeman, J., & Ahrens, M. B. (2014). Light-sheet functional imaging in fictively behaving zebrafish. *Nature Methods*, *11*(9), 883–884.

We are grateful to the reviewers for their constructive comments. We have done extensive innovation, experimentation, analysis, and writing, and we hope that we have now addressed their comments in full.

Regarding formatting of our response: each reviewer's comment is highlighted in gray. Our responses are in black font. Brown font indicates quotes from the revised manuscript that reflect the added/changed text in response to reviewers' comments. We numbered the figures in this response (i.e., in the black font parts) in the order they appear in this response. Their corresponding figure numbers in the revised manuscript are shown in parentheses following each figure number (all figures in the response to reviewers were added to the manuscript itself, unless otherwise indicated). In text quoted from the revised manuscript (brown font), figure numbers refer to actual numbers in the revised manuscript (since the text is quoted therefrom).

Reviewer #1:

Remarks to the Author:

Achieving whole brain voltage imaging at cellular resolution would be highly valuable for understanding information flow across brain regions at spike resolution. In comparison, most current calcium imaging efforts are limited by the slow kinetics of the sensor and imaging methods. Thus, I commend the authors for their efforts in working towards this goal.

Having said that, the current manuscript has not achieved the goal of whole-brain spike-resolution imaging at cellular resolution. The authors are recording 1/3 of the brain (i.e. not the majority of the brain) at 243 vps, which can potentially miss a significant number of spikes given the fast kinetics of the sensor.

(1) We agree with the reviewer that our current method has not achieved whole-brain, spike-resolution imaging at cellular resolution. We had tried quite hard to make our language appropriately modest – e.g., using the phraseology "neurons distributed across entire brains," and not claiming recording of every single neuron in the entire brain. We have worked further to make the language consistent throughout the revised manuscript (see **lines 32, 169-170, 388, 923, 1012, 1046, 1057, 1085-1086, 1265, 1270** in the revised manuscript, highlighted text), and welcome feedback from the editors as they see fit, regarding this point of framing.

(2) The fraction of neurons in the brain could in principle be improved, but some such efforts may be beyond the scope of the current paper (e.g., they relate to innovation in the field of zebrafish transgenesis). For example, our fish line has some brain regions where not all neurons are labeled (e.g., **Fig. 2a** in the original and revised manuscript, z = -23 µm, -51 µm, -79 µm, cyan arrows). In our paper, we used the "pan-neuronal" promoter HuC (also known as elavl3) to express the Gal4 transcription factor, which binds tandem UAS enhancers to activate Positron2-Kv expression in neurons. We chose to use the Gal4/UAS expression system due to its high gene expression strength (Scheer and Campos-Ortega 1999; Asakawa et al. 2008). However, in transgenic larval zebrafish, fluorescent reporter genes regulated by tandem UAS copies often display variable expression, likely as a result of transcriptional silencing mediated by DNA methylation (Akitake et al. 2011). We attempted to screen for fish with more pan-neuronal genetically encoded voltage indicator (GEVI) expression, by performing more embryo injections and trying different promoters. We found that directly expressing Positron2-Kv using the HuC promoter, compared to using HuC:Gal4 and tandem UAS, did result in more pan-neuronal expression, but this also decreased fluorescent brightness (**Figure 1**, showing a comparison of confocal images of a 6 day-post-fertilization (dpf) F2 generation Tg(HuC:Gal4; 2xUAS:Positron2-Kv) fish and a 5-dpf F1 generation Tg(HuC:Positron2-Kv) fish), potentially making brain-wide voltage imaging more difficult. The images of the two fish were acquired using the same microscope settings. Compared with the Tg(HuC:Positron2-Kv) fish, the Tg(HuC:Gal4; 2xUAS:Positron2-Kv) fish had higher brightness (~1.1× averaged over the whole brain, ~1.7× averaged over the whole optic tectum (OT), and ~2.6× averaged over the whole habenula (HB)). The Tg(HuC:Gal4; 2xUAS:Positron2-Kv) fish brain also showed more varied levels of expression across different brain regions. (Please note, these were not systematic explorations: we did not control for precise fish age or generation, and examined only one fish for each type. We did not image the Tg(HuC:Positron2-Kv) fish under our light-sheet microscope, either. The Tg(HuC:Positron2-Kv) fish could potentially be used in future experiments to improve the number of imaged neurons, given that our revised microscope's light efficiency was enhanced in a way that could make up for reduced neuron brightness (we

demonstrated a 75% improvement in light efficiency using dual remote mirrors in the revised microscope, see point (27)). In any case, fish transgenic line generation is an ongoing area of innovation for the entire fish community, and as that field continues improving, so will the performance of voltage imaging. The new text has been included in **lines 425-446** in the revised manuscript, and associated figures.

(3) Regarding the possibility of missing spikes due to the low sampling rate, please see our detailed response in point (30) below, which includes extensive new experiments, analyses, and more. In summary, due to the mismatch between Positron2-Kv's sub-millisecond kinetics (Kannan et al. 2022) and our 5-ms sampling interval, approximately 70% of the voltage spikes could be retrieved in the 200-Hz neural traces (**Figure 13**). However, we showed that this spike-missing problem can be potentially solved by using indicators with slower kinetics: 99.7% of the voltage spikes could be retrieved in the 200-Hz neural traces using ASAP5-Kv (Hao et al. 2024) in simulation (**Figure 14a-c**), and all spikes were preserved at a 200-Hz imaging rate in a zebrafish brain transiently expressing ASAP5-Kv (**Figure 14d-e**).

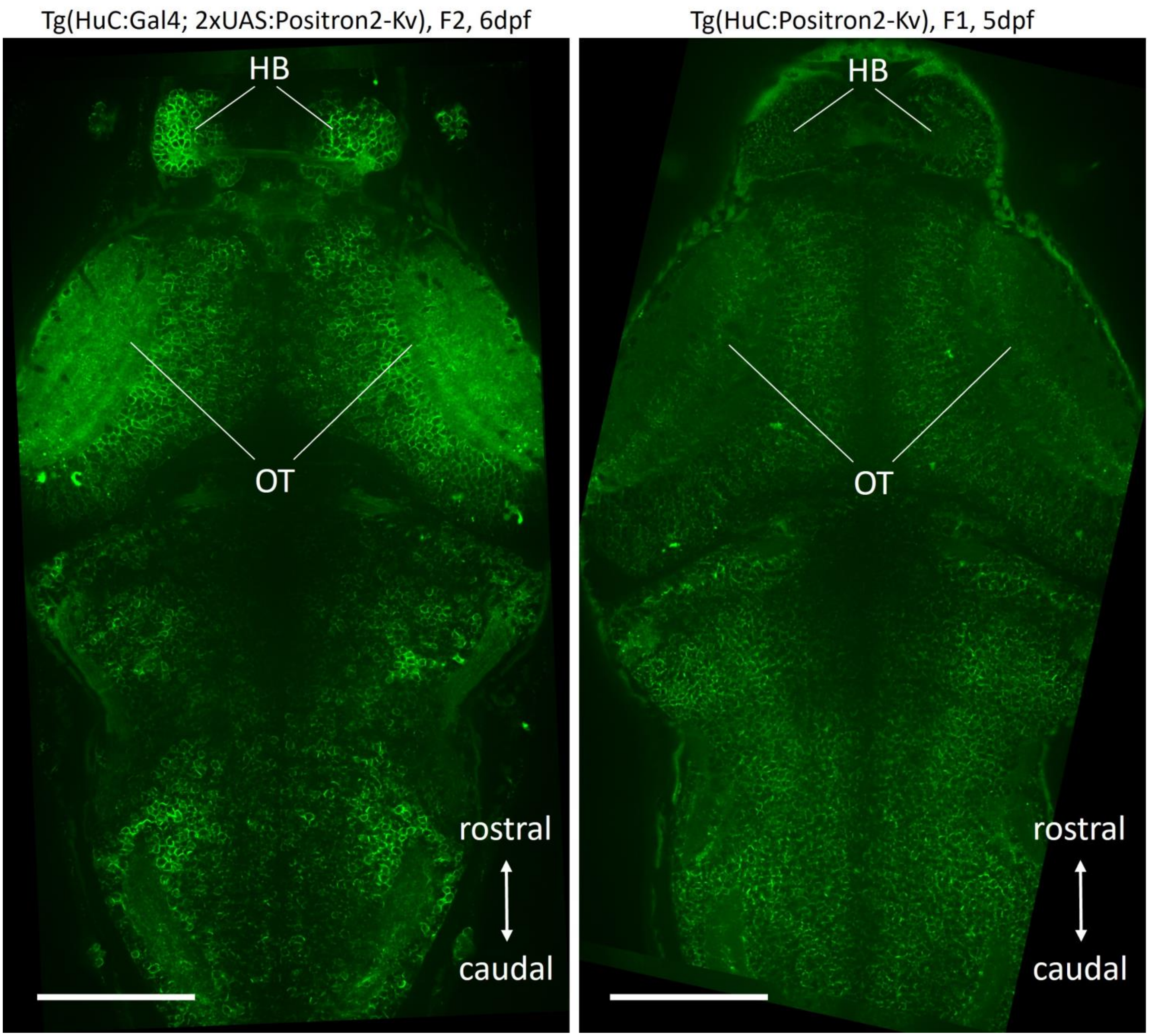

Figure 1 (Figure S29 in the revised manuscript). Comparison of Positron2-Kv expression in zebrafish brains using different promoters. Confocal microscopy images of zebrafish brains expressing Positron2-Kv using Gal4/2×UAS (left, F2 generation at 6 dpf) and HuC (right, F1 generation at 5 dpf) promoters. Images were acquired using the same microscope settings and displayed using the same intensity scale. OT: optic tectum. HB: habenula. Scale bar: 100 μm.

Setting aside the claim of whole brain voltage imaging, achieving voltage recording across a large portion of the brain would still be valuable. However, at the conceptual level, the authors' microscopy design closely follows existing designs for light sheet microscopy with remote focusing. The one major technological upgrade proposed is the use of a commercially available high-speed camera. Data analysis also uses existing methods and relies heavily on manual processing, and the quality of the data processing hasn't been sufficiently demonstrated.

(4) We have worked extensively to reduce the need for manual processing, and to validate the quality of the data processing. We trained a deep learning model to automatically segment regions-of-interest (ROIs) corresponding to putative neurons using the deep network models in Cellpose 3.0 (Stringer and Pachitariu 2024). To prepare the training data, motion correction was first applied to the video of each z-layer, followed by generating a static image by averaging the first 100 frames of the same layer. These static images were then spatially upsampled and interpolated by a factor of 2 in the x-y direction to enhance pixel density, facilitating easier cell labeling in the Cellpose 3.0 graphical user interface (GUI).

We first used the pre-trained Cellpose 3.0 model (Stringer and Pachitariu 2024), but realized the model performed poorly on our dataset, likely due to challenges with out-of-distribution generalization to our dataset. We therefore manually segmented a few thousand neurons and trained our own Cellpose model in the grayscale mode. In our manual segmentation process, we identified and outlined objects that were approximately the size of a neuron (~6.6 μm) and had ring-like boundaries due to membrane labeling. Using a "diam_mean" parameter value of 15 (to match the neuron size) and a learning rate of 0.02 (chosen somewhat arbitrarily, but we found it allowed the model to converge well during training), we observed that the model could correctly segment over 70% neurons after 1000 training epochs, treating the hand annotation as the ground truth. During testing, one can adjust parameters including "flow threshold", "cellprob threshold" and "diameter pixel" in the Cellpose GUI to increase the number of neurons detected. "Cellprob threshold" and "flow threshold" are two parameters controlling how sensitive the model is to detect ROIs (Stringer and Pachitariu 2024). Increasing the "flow threshold" or decreasing the "cellprob threshold" can result in more ROIs, but also more false positives. "Diameter pixel" refers to the average diameter the model uses to identify ROIs, in pixel units. Because increasing the ROI detection sensitivity will also raise the false positive detection rate, a user must choose the right balance to minimize the amount of manual correction. For this paper, we chose a "diameter pixel" of 18, "flow threshold" of 0.4 and "cellprob threshold" of 0. Diameter pixel of 18 was chosen to match the average size of the neuron in our dataset. We noticed that using other values could lead to over or under segmentation. "Flow threshold" and "cellprob threshold" were chosen as the default values given by the Cellpose GUI. We also adopted their iterative bootstrapping idea (Stringer and Pachitariu 2024), where we trained the model with labelled data, and iteratively fed manually-corrected predictions back into the model. In total, training data from five fish, comprising >80,000 ROIs, were used in training of our custom CellPose model. The model's performance (i.e. the number of ROIs labeled) improved as we expanded the training dataset. Eventually, it reached a point where adding more training ROIs did not improve the model's performance significantly. Therefore, we stopped the training after using 5 fish's ROIs for training, totaling over 80,000 ROIs.

We evaluated the performance of the segmentation model on a randomly selected fish dataset that had not been previously labeled (**Figure 2**). For each test layer, the segmentation model was applied to identify and annotate the ROIs. Next, an experienced experimenter visually inspected each layer to add ROIs that the model missed and removed incorrectly labeled ROIs. We used a flow threshold of 0, which disabled the mask rejection. This will result in an increased number of cells segmented but also increase the number of false positives. The visual inspection used the same criteria to identify ROIs as during our manual segmentation process, i.e., annotating objects that were approximately the size of a neuron (~6.6 μm) and that had ring-like

boundaries. On average, per layer, the experimenter added 80.2 ± 27.5 (mean ± standard deviation, n=30 layers) ROIs and removed 10.9 ± 9.4 (mean ± standard deviation, n=30 layers) ROIs from the model's output. In addition to this evaluation dataset, all fully analyzed datasets in the paper underwent this same automatic annotation and manual correction process to identify their ROIs. This point has been integrated as highlighted text in **lines 544-564, 1303-1332** in the revised manuscript, and associated figures.

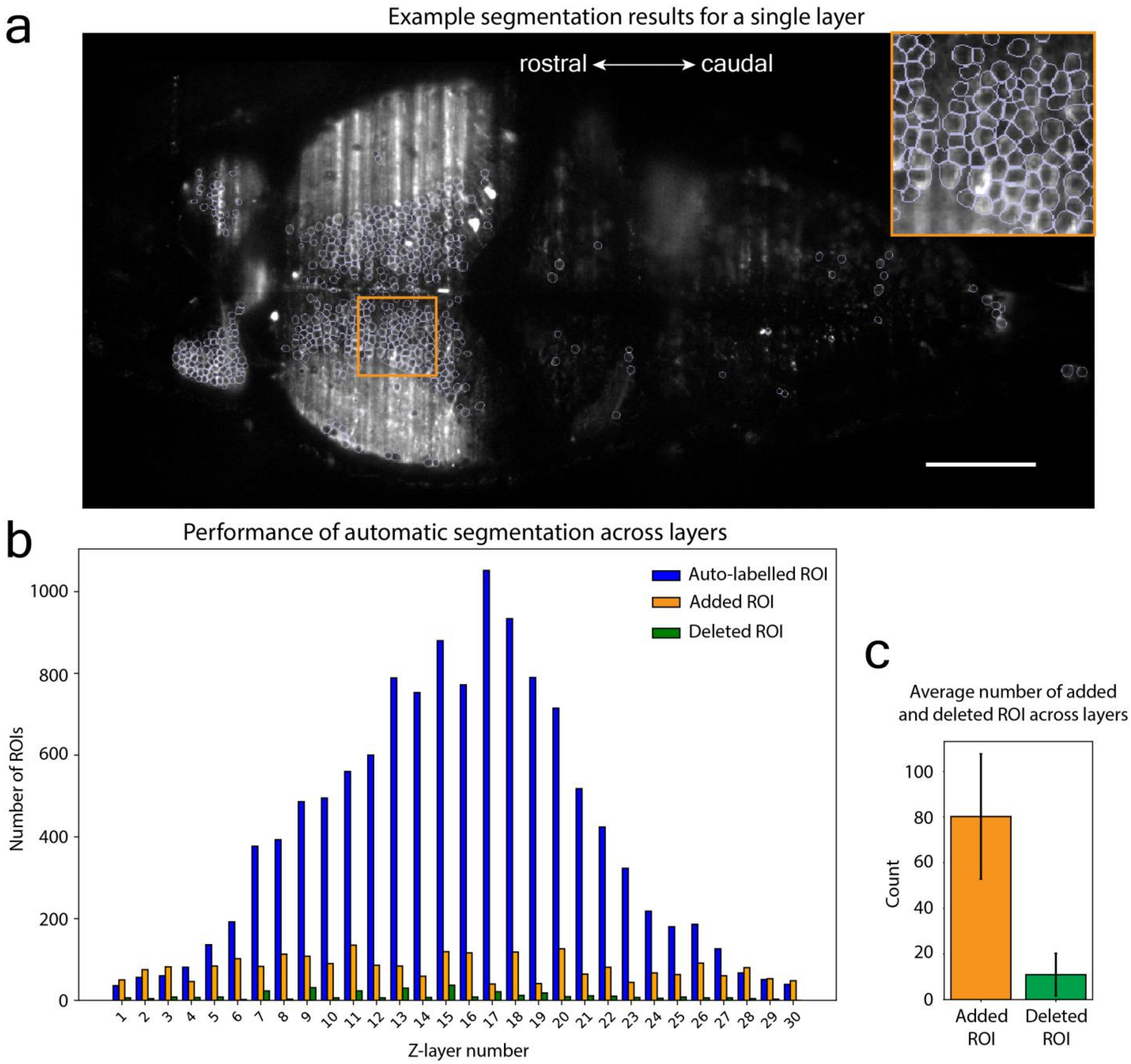

Figure 2. (Figure S18 in the revised manuscript) Performance of our automatic ROI segmentation model. **(a)** The segmentation results for a randomly selected single z-layer (out of 30) from a single unlabeled fish. The ROI contours (light purple lines) are overlaid on the image. A magnified view of the orange boxed area is shown in the top right corner. Scale bar: 100 μm. **(b)** Segmentation performance of the model on an entire fish. Blue bar: the number of ROIs labeled by the model; orange bar: the number of ROIs added by the experimenter, indicating ROIs that the model missed; green bar: the number of ROIs deleted by the experimenter, indicating ROIs that the model falsely labeled. Z-layers are numbered according to their spatial depth in the brain. Smaller numbers correspond to deeper layers. **(c)** On average, the experimenter added 80.2 ± 27.5 ROIs (mean ± standard deviation, n=30 layers) and removed 10.9 ± 9.4 (mean ± standard deviation, n=30 layers) ROIs from the model's output.

Thus, if this paper does not contribute significant original advances in hardware design, software design, or data processing, then the emphasis must be on the quality of the overall implementation, as the authors state that they have achieved a "microscope optimized for the imaging of the voltage of individual neurons distributed throughout the entire larval zebrafish brain". Here, I have several major concerns that would need to be addressed.

(5) While we agree with the reviewer that at the conceptual level, our microscope follows the widespread idea of light-sheet microscopy with remote refocusing, there were several necessary innovations in the design and implementation of the microscope, that brought its capabilities to the level required for voltage imaging of neurons distributed across entire brains. We have emphasized these innovations more clearly. (See **lines 1014-1039** in the revised manuscript, highlighted.)

1. We have designed a new remote scanning module, and the software and pipeline for calibration and control. We combined a large shift, high resonant frequency piezo bender scanner with a lightweight mirror for remote scanning, to enable 200-μm axial scanning at up to 300 Hz (or >100 μm at 500 Hz). We also developed the hardware, software, and pipeline (see "Implementation of the microscope design" section in our manuscript) to allow accurately calibrating and controlling the remote mirror movement. This is a nontrivial design, because accurately calibrating and controlling the movement of a piezo bender scanner—which operates at hundreds of hertz in open-loop mode, and exhibited hysteresis—was challenging. To achieve the desired waveform, we had to measure the piezo bender's movement at a rate of tens of kilohertz, and at sub-micrometer precision, and conduct iterative fine adjustments of the driving signals that contained multiple unconstrained parameters.

2. Remote refocusing enhances the microscope's optical scanning rate, but exacerbates the mismatch between the microscope's optical scanning speed, and the camera's acquisition speed. To alleviate this mismatch and improve imaging speed, we combined distributed planar imaging (Greer and Holy 2019) with remote-refocusing light-sheet microscopy, efficiently doubling the volume rate for voltage imaging. This strategy could in principle be further scaled up, by chaining multiple distributed planar imaging modules and splitting the image to more cameras, to achieve even higher volume rates.

3. We are the first (to the best of our knowledge) to use sensors designed to combine high frame rates with large camera pixel numbers, for high-speed microscopy imaging. Indeed, we had to build a custom camera using this sensor, for our initial microscope implementation, and to assess its performance. Our custom camera's firmware and software integrate open-source architectures widely used in the neuroscience community (Firmware: Open-Ephys ONIX; Software: Bonsai), and could significantly accelerate future high-speed camera development, reducing the time required to adapt novel sensor chips into microscope architectures. As we note, since our initial work, commercial camera manufacturers have now introduced cameras based on this sensor, easing its adoption by other labs.

1. The biggest problem with the current implementation is the image quality of the microscope. The "stripe" artifacts are significantly worse than expected for most mature LSM implementations. As it stands, the authors have not demonstrated that 1) they fully understand the source of these artifacts, and 2) they can correctly remove these artifacts during data analysis. I would have serious concerns about building a complicated microscopy system with these artifacts, and how much to trust the data generated with this system. I'll elaborate in more detail on this issue:

(6) The "stripe" artifact in our images is a common issue when using light-sheet microscopes to image "non-fully transparent" samples (Ricci et al. 2022; Taylor et al. 2018; Sancataldo et al. 2019; Ricci et al. 2020). Here non-fully transparent means that some structures, objects, or regions of the sample can absorb, refract, or scatter the excitation light sheet, altering its propagation. These structures, objects, or regions therefore will "cast" stripe-shaped artifacts extending from themselves to the edge of the sample, along the light-sheet illumination direction. While translucent, the larval zebrafish brain is non-fully transparent. The fish's skin has

different refractive index than water, and a non-flat surface, so it will refract the light sheet in a non-uniform way; zebrafish brain tissue is non-uniform and has heterogenous refractive indices, which can refract and scatter the light sheet; blood cells have round shapes and different intracellular refractive index than their surrounding environment, which could make them act as micro-lenses (Miccio et al. 2015) to converge or diverge light passing through them. Such “stripe” artifacts and possible strategies to alleviate them have been discussed in this review paper (Ricci et al. 2022). One idea that has been used in multiple works (Sancataldo et al. 2019; Ricci et al. 2020; Huisken and Stainier 2007) involves rapidly pivoting the illumination light sheet in the image plane so that these stripe artifacts are “dispersed” and averaged out over broader areas. In our revised paper, we fully characterized the stripe artifacts by performing a series of experiments (see **Figures 3-5,** and points **(10)-(17),(19)** below, for detail). The stripe artifacts in our experiments include “stationary stripes”, which are caused by stationary structures that affect light-sheet propagation, and “moving stripes”, which are caused by moving or dynamical structures that affect light-sheet propagation. In summary, our experiments demonstrated that 1) the “stripe” artifacts (unless otherwise specified, the term 'stripe' artifacts refers to both stationary and moving stripe artifacts.) originated from the sample instead of the microscope working alone, e.g. they were eliminated by imaging a sample of agarose (**Figure 3** and associated points below); 2) blood flow in the zebrafish brain was the cause of moving “stripe” artifacts (**Figure 4-5** and associated points below); 3) a light-sheet pivoting strategy could indeed remove “stripe” artifacts in the image formation phase (**Figure 7, 10b,** and associated points below). This point has been integrated in highlighted text in **lines 598-611, and 625-784** in the revised manuscript, and associated figures.

1a. LSM systems do not generate stationary banding patterns if implemented correctly. I do not find the explanation that these are caused by pigment in the fish convincing. Given the brain images shown, the author must be using a pigment mutant (probably mitfa -/-, which should have been specified in the paper), or else much of the brain would be entirely occluded. These pigment mutants do not have pigment cells at the side of the head below the eyes. The stripes are also not occasional darker lines of occlusion in an otherwise uniformly bright sheet, which is what would be expected if they are due to pigment cells or occluding objects. Instead, they are intense alternating bands of light and dark, which suggests a potentially serious undiagnosed issue with the optical implementation, not the animal.

(7) While LSM systems do not generate stationary banding patterns by themselves, the propagation of light sheets through biological samples (e.g., larval zebrafish) does cause stripe artifacts in their raw images (Ricci et al. 2022; Taylor et al. 2018; Sancataldo et al. 2019) (see point **(6)**). This is a common issue in the LSM field. The causes of the stripe artifacts and potential solutions are discussed in points **(6),(19)** and also in this review paper (Ricci et al. 2022). We have emphasized this in the revised text. (See **lines 598-611** in the revised manuscript, highlighted.)

(8) We did use a pigment mutant fish (mitfa$^{-/-}$, also known as nacre). We have added that information to the manuscript (see highlighted text in **lines 609-611, 1219-1221** in the revised manuscript). However, nacre fish still develop some opaque tissues at 5 dpf, for example, on the skin above the hindbrain, which could potentially affect the light-sheet illumination when imaging the top layers of the hindbrain. In addition, while translucent, the larval zebrafish brain and the tissues around the brain are not optically uniform in terms of transparency and refractive index (see point **(6)**). The fish’s skin is not flat, either. These non-uniform tissues can absorb, scatter, and refract the parallel light sheet, causing stationary stripe-shaped artifact patterns in the raw images. We agree with the reviewer that simply using the word “pigment” here is not appropriate; we amended our text to make these descriptions more accurate and detailed (see lines **598-611** in the revised manuscript, highlighted).

(9) Regarding the reviewer’s concern that the stripe artifact may come from an issue related to the hardware instead of the animal, we performed the control experiments described in points **(10)(11)**. In brief, our results (**Figure 3** and associated text) showed that the stripe artifacts were caused by small objects or structures in the animal that affected the light sheet propagation, not by the hardware itself. To examine the source of moving stripe artifacts, we conducted another set of experiments described in points **(12)-(17)**. In

brief, our experiments (**Figures 4-5** and associated text) showed that the moving stripe artifacts came from the animal's blood flow. This point has been integrated in highlighted text in **lines 625-681** in the revised manuscript, and associated figures.

Some controls should be performed:

- Do these stripes exist when imaging a fluorescein bath? Or in a dense sample of fluorescent beads?

(10) To test if the stripe artifacts came from the optical setup alone, we imaged a 3D agarose sample containing evenly distributed JF525 fluorescent dye following the same protocols that we used to perform 200-Hz voltage imaging of neurons distributed across entire brains in larval zebrafish. The raw images of the fluorescent agarose are shown in **Figure 3a**. In these agarose images, we saw a gradual variation (~±20% along an 800-µm x-axis range) of intensity along the x-axis, which is perpendicular to the illumination axis (**Figure 3c**). This gradual intensity variation was caused by the non-uniformity of the Powell lens-generated light sheet, due to imperfections on the lens surfaces. The intensity variation pattern was consistent across different depths (**Figure 3a**), did not change over time, and exhibited low spatial frequency along the x-axis ($<0.05\ \mu m^{-1}$, meaning the intensity changes in space were gradual and smooth) (**Figure 3d**). In contrast, the stripe artifacts observed in zebrafish brain images differed between different brain layers (**Figure 4b**) and featured sharp bright and dark bands (highlighted by red arrows in **Figure 3e**). These stripe artifacts were characterized by high spatial frequencies ranging from $0.05\ \mu m^{-1}$ to $0.2\ \mu m^{-1}$ along the x-axis (**Figure 3f**, red arrows). We did not observe such stripe artifacts in the agarose images. In addition, we also added 200-nm diameter fluorescent beads in the agarose. Sharp, in-focus images of these beads were clearly visible (**Figure 3a**, orange arrows), indicating the absence of stripe artifacts was not due to defocusing. This result showed that the stripe artifacts were not generated by the imaging setup itself.

The ~±20% gradual intensity variation along the x-axis, mentioned in the previous paragraph, is unlikely to pose challenges for imaging and extracting the voltage activity of neurons in the fish's brain. This is because: 1) the intensity variation was smooth and gradual across space, meaning that it does not corrupt the boundaries of individual neurons, and 2) the intensity variation stays stationary over time, ensuring that no temporal artifacts are introduced to the neural activity traces. If needed, this gradual intensity variation could be in principle corrected in the raw sample images by using reference images of a uniform fluorescent sample, such as a 3D fluorescent agarose sample.

This point has been integrated in highlighted text in **lines 625-650** in the revised manuscript, and associated figures.

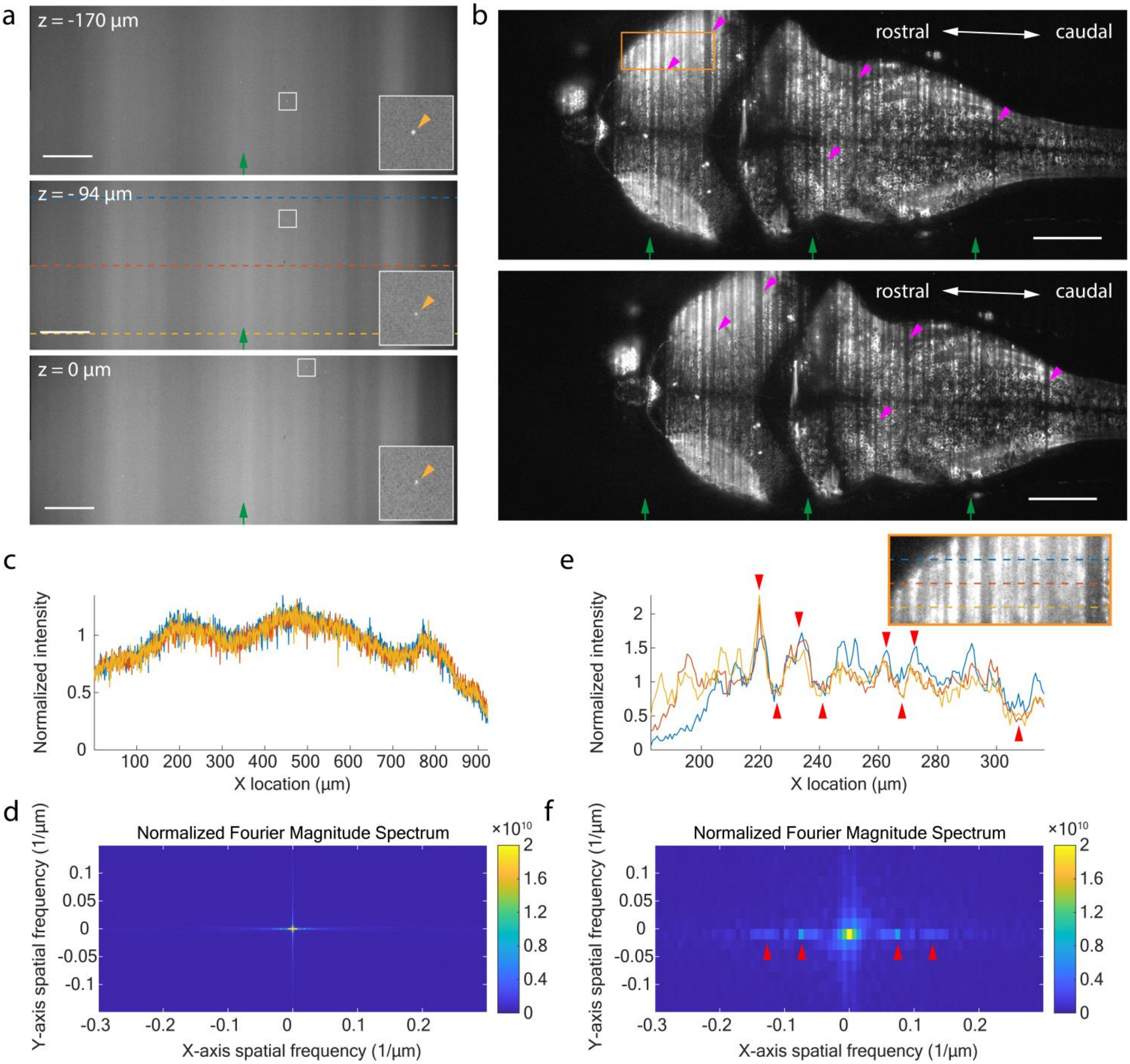

Figure 3. (Figure S13 in the revised manuscript) Control experiments indicated that the stripe artifacts did not come from the setup and were associated with the animal. (a) Raw light-sheet images of a JF525-dye containing agarose sample. Single-plane images at different depths (z) are shown. Green arrows denote the direction of light sheet illumination. The agarose also contained sparse 200-nm diameter fluorescent beads, whose focused, sharp images (orange arrows) can be seen in the enlarged boxes (they are too small to be seen easily, in the zoomed-out images). The intensity profiles along three lines (blue, red, and orange dashed lines) in the central image were plotted in (c). Scale bar: 100 µm. **(b)** Comparison of raw single-plane images of a zebrafish brain as the fish was moved along the x-axis (horizontal axis in these images). Stationary stripe artifacts (magenta arrows) remained fixed in position with respect to the brain, but not with respect to the microscope, suggesting these stationary stripes were associated with the fish itself. Green arrows denote the direction of light sheet illumination. The orange-boxed area in the top image was used for profile plotting and Fourier analysis in (e-f). The orange-boxed area avoids areas with fine neuron soma boundaries, which possess high spatial frequencies that could potentially complicate the Fourier analysis of stripe artifacts. Scale bar: 100 µm. **(c)** Intensity profiles along three lines (blue, red, and orange) in the central image of panel (a) were plotted, illustrating

the variation of intensity along the x-axis in the agarose image. These profiles were normalized by their mean values. **(d)** Magnitude spectrum of the two-dimensional Fourier transform of the central agarose image shown in panel (a), normalized by its mean value. **(e)** Intensity profiles along three lines (blue, red, and orange dashed lines) within the orange-boxed area (zoomed in at the top right corner) of the top image in panel (b). These profiles were normalized by their mean values. Red arrows show the locations of bright and dark stripes. **(f)** Magnitude spectrum of the two-dimensional Fourier transform of the orange-boxed area in panel (b), normalized by its mean value. Red arrows highlight high spatial frequency components corresponding to stripe artifacts.

- Are the positions of the stripes consistent across samples?

(11) The imaged fish slightly differed in their ages at the time of imaging (by half an hour to a few hours, as they were not imaged at the same time) and in their orientations in the agarose (we manually oriented the fish using forceps under a stereoscope; the fish could be slightly tilted by small angles, which was difficult to control under visual guidance); there may also have been minor differences in the shapes of their lateral skin surfaces and the structures of their brain tissues, due to non-identical genomes and different development stages of these fish. Therefore, it might be challenging to characterize the source of either the stationary stripes or the moving stripes by quantifying the stripe consistency across different samples -- all these factors, which we did not and could not control, might affect any such stripe consistency analysis. Alternatively, we performed another control experiment to test if the stationary stripes were sample vs. hardware dependent (**Figure 3b**). In this experiment, we shifted the fish along the x-axis (roughly aligned with the rostral-caudal axis, perpendicular to the illumination axis) in the lateral plane while imaging the zebrafish brain at 200.8 Hz. The raw images of the same brain plane at two different locations along the x-axis are shown in **Figure 3b**. The stripe patterns between the two brain images were highly consistent and remained at the same locations relative to the fish's brain (**Figure 3b**, magenta arrows), and not relative to the microscope's field-of-view. This result suggested that the stationary stripes were dependent on the samples, not the imaging setup. This point has been integrated in highlighted text in **lines 651-658** in the revised manuscript, and associated figures.

1b. Similarly, I'm not convinced that the authors understand the source of the moving stripes, which can potentially contaminate real neural signals. In the supplementary movie shown (and in images in the figures), these moving stripes have two curious properties. One, they all appear to have a constant width across the entire brain (from left to right). Two, the stripe can either increase or decrease in brightness. Both properties are not expected due to scattering by blood flow, which 1) should interrupt the light sheet beam at different offsets from the side of the head, 2) cannot generate a suddenly brighter stripe of constant diameter across the brain (i.e. blood cells can only scatter light), 3) should occur at well-defined positions in the animal.

Again, some controls should be performed:

(12) We agree with the reviewer that we need to understand the source of the moving stripes, and ideally point the way towards reducing or compensating for these artifacts. We performed several experiments (**Figure 4,5**, below) to examine the sources of moving stripes (see text in **(14)(15)(16)**). In brief, our results indicate that the moving stripes were caused by blood flow in the zebrafish brain. To compensate for these artifacts, we demonstrated a hardware approach (**Figure 7**, below) to reduce both the stationary and moving stripe artifacts by rapidly pivoting the light sheet in the imaging plane (see our response point **(19)** for more details). In brief, this approach adopted concepts from previous work (Sancataldo et al. 2019; Huisken and Stainier 2007; Ricci et al. 2020) aiming to address this stripe artifact problem. In our experiment, this approach removed all stationary stripes in raw images, and improved moving-stripe induced temporal artifacts in 88% of affected neural traces (**Figure 7**). This point has been integrated in highlighted text in **lines 659-743** in the revised manuscript, and associated figures.

(13) Regarding the three points that the reviewer made about the blood flow explanation, 1) we actually did observe moving stripes originating at different offsets from the left side of the brain (i.e., the side where the

light sheet entered the brain) (**Figure 5,** magenta arrows). Besides the moving stripes originating from the left side, some moving stripes originated within the brain (**Figure 5,** magenta arrows). 2) Blood cells can not only scatter light, but also refract light like microlenses (Ricci et al. 2022). With this lens-like property, blood cells in the fish brain can focus or diverge parallel illumination light to produce both bright and dark moving stripe patterns. 3) For the reasons we discussed in point (11), we did not perform a consistency analysis of the moving stripe distribution across different fish brains. However, we did see some patterns about where, at the brain region level, the moving stripes more likely occurred in the zebrafish brain: the lower brain layers typically tended to have more moving stripes (e.g., **Figure 4b**, z = -170 μm) and almost every fish had moving stripes in the optic tectum region near middle brain layers (e.g., **Figure 4b,** z = -82 μm). That being said, in our experiments we encountered 3-4 fish (out of dozens of fish that we imaged) that had significantly fewer (approximately 80-90% less) moving stripe artifacts than other fish. We don't know the exact reason for this, but these fish exhibited good neural activity, and we didn't observe any abnormality either visually, or when analyzing their data. We speculate they could have less blood flow (zebrafish can live until 7 dpf without blood flow (Kugler et al. 2021)), or their blood cells might be less scattering or have refractive index closer to their surrounding environment. We did not carefully study these observations, as characterizing the properties of fish lines is not the main focus of our paper. Here we just mention them to note that while there might be some consistency of the moving stripe distributions across fish, these artifacts can also vary a lot from fish to fish in terms of their exact locations and quantity. This point has been integrated as the first point in **Supplementary Note 1.**

- Are these stripe artifacts observed in a post-fixation animal without blood flow? In fluoresceine or bead samples?

(14) We imaged the brain of a paraformaldehyde (PFA)-fixed 5-dpf transgenic Positron2-Kv larval zebrafish at 200 Hz. We did not observe any moving stripe artifact in the recording (**Figure 4a**). To better visualize and map moving stripes' distributions in the fish brain, we computed the normalized temporal standard deviation map (NTSDM) of a given time-series recording. In brief, NTSDM can highlight moving stripe artifacts as bright "stripes" along the illumination direction. Please see point (17) for a detailed description of the computation process for NTSDM, and the underlying rationale. This point has been integrated in highlighted text in **lines 659-667** in the revised manuscript, and associated figures.

(15) Additionally, to further test the dependence of moving stripes on animals' blood flow, we stopped the zebrafish heart (thus stopping blood flow) temporarily during imaging using 2,3-butanedione monoxime (BDM), as used in a previous study (Ricci et al. 2022). The moving stripes observed prior to BDM treatment disappeared once the fish's heart was stopped (**Figure 4b, Movie S2**). The stopped heart started again after incubating the BDM-treated fish in fresh water for ~20 mins. Following the recovery of the fish's heartbeat, moving stripes reappeared in the video. (**Figure 4b, Movie S2**) This experiment clearly demonstrated that the moving stripes were dependent on the zebrafish's heart pulsing, and thus blood flow. This point has been integrated in highlighted text in **lines 667-674** in the revised manuscript, and associated figures.

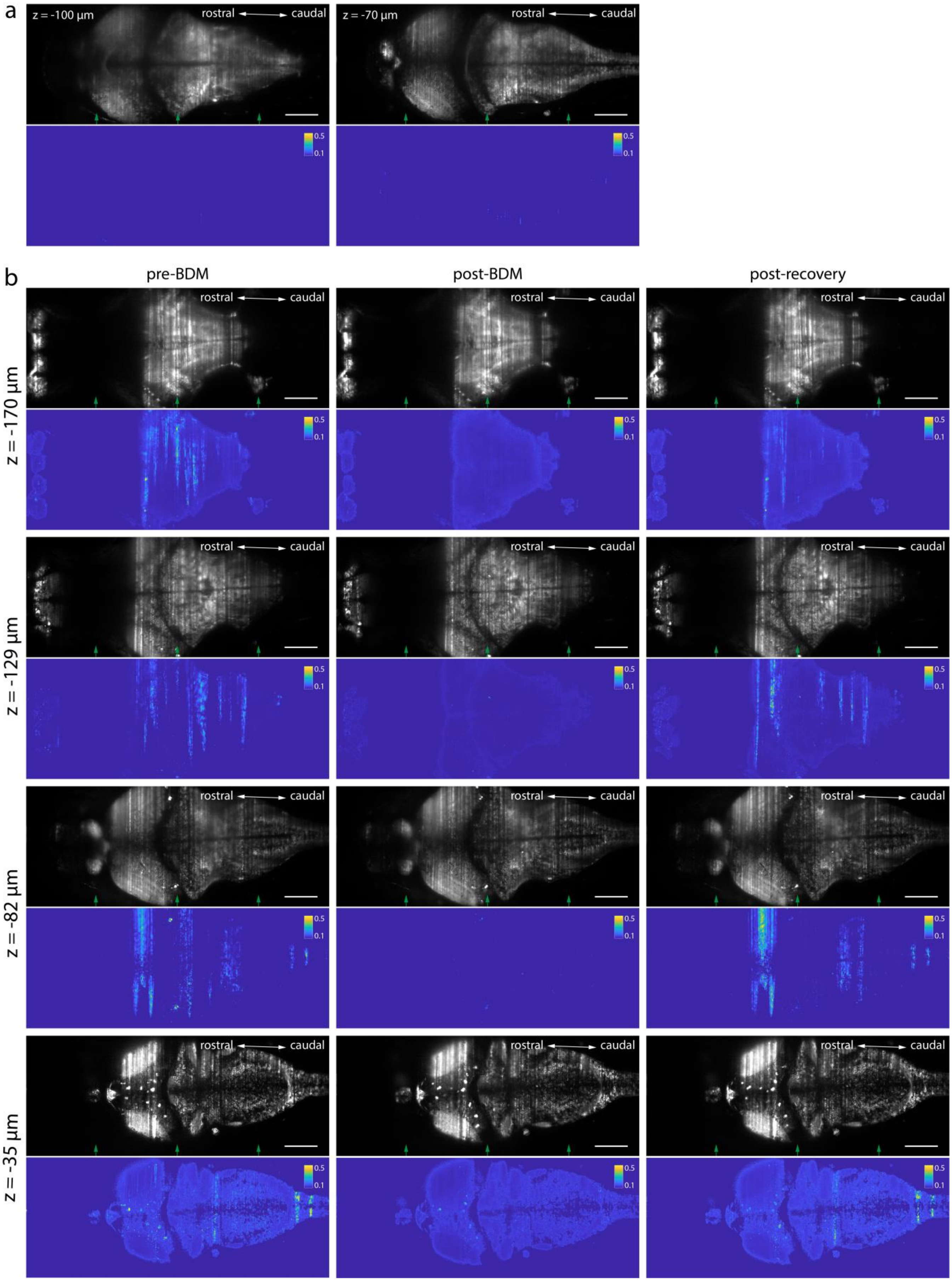
a
z = -100 µm
z = -70 µm
rostral
caudal
0.5
0.1
b
pre-BDM
post-BDM
post-recovery
z = -170 µm
z = -129 µm
z = -82 µm
z = -35 µm

Figure 4. (Figure S14 in the revised manuscript) Imaging of fixed fish and BDM-treated fish suggested that moving stripe artifacts were dependent on the animal, presumably on blood flow. (a) Fixed fish showed no moving stripe artifacts in raw videos. The top panel displays two randomly selected planes of a fixed brain, with green arrows indicating the direction of light-sheet illumination. Below each single-plane image, the corresponding normalized temporal standard deviation map (NTSDM, see point **(17)** for more details) is shown to visualize any moving stripes. No bright stripe was seen on the maps. Scale bar: 100 µm. **(b)** BDM treatment removed moving stripes in the live zebrafish brain. Each row presents the raw images of a randomly selected plane (out of 30) of a zebrafish brain and their corresponding normalized temporal standard deviation maps (NTSDM) before the BDM-treatment (left), after the BDM-treatment (middle), and post recovery (right). A total of four planes of the same zebrafish brain are shown. Moving stripe patterns that were visible before BDM treatment (left column) disappeared after the BDM treatment (middle column). After the fish recovered in fresh water, the moving stripe patterns appeared again (right column). Scale bar: 100 µm.

- Are the positions where the moving stripe occurs roughly consistent across animals? This should be the case if this is due to blood flow. Simply claiming (with no citations) that the circulatory system of animals is different across individuals is not sufficient. Age-matched sibling animals should be used for this control. There are also transgenic animals that label the circulatory system, which can be used to control for any minor differences in blood vessel layout.

(16) Instead of comparing the moving stripe distributions across different animals (for reasons we discussed in point (11)), we performed a precise experiment to examine the spatial correlation of moving stripes and the animal's blood vessels, as the reviewer suggested (**Figure 5**). In this experiment, we crossed our transgenic GEVI fish with Tg(flk:DsRed) fish, which have red vascular labeling, and imaged both the green GEVI signals and red blood vessels in the same imaging session by alternating emission filters. We correlated the spatial locations of moving stripes with blood vessels in the fish's brain (**Figure 5**). We found that every moving stripe (**Figure 5**, cyan stripes) originated near a corresponding blood vessel (**Figure 5**, magenta arrows). This finding strongly supports the conclusion that the moving stripes were caused by blood flow that altered the light-sheet propagation in a dynamical way. This point has been integrated in highlighted text in **lines 674-681** in the revised manuscript, and associated figures.

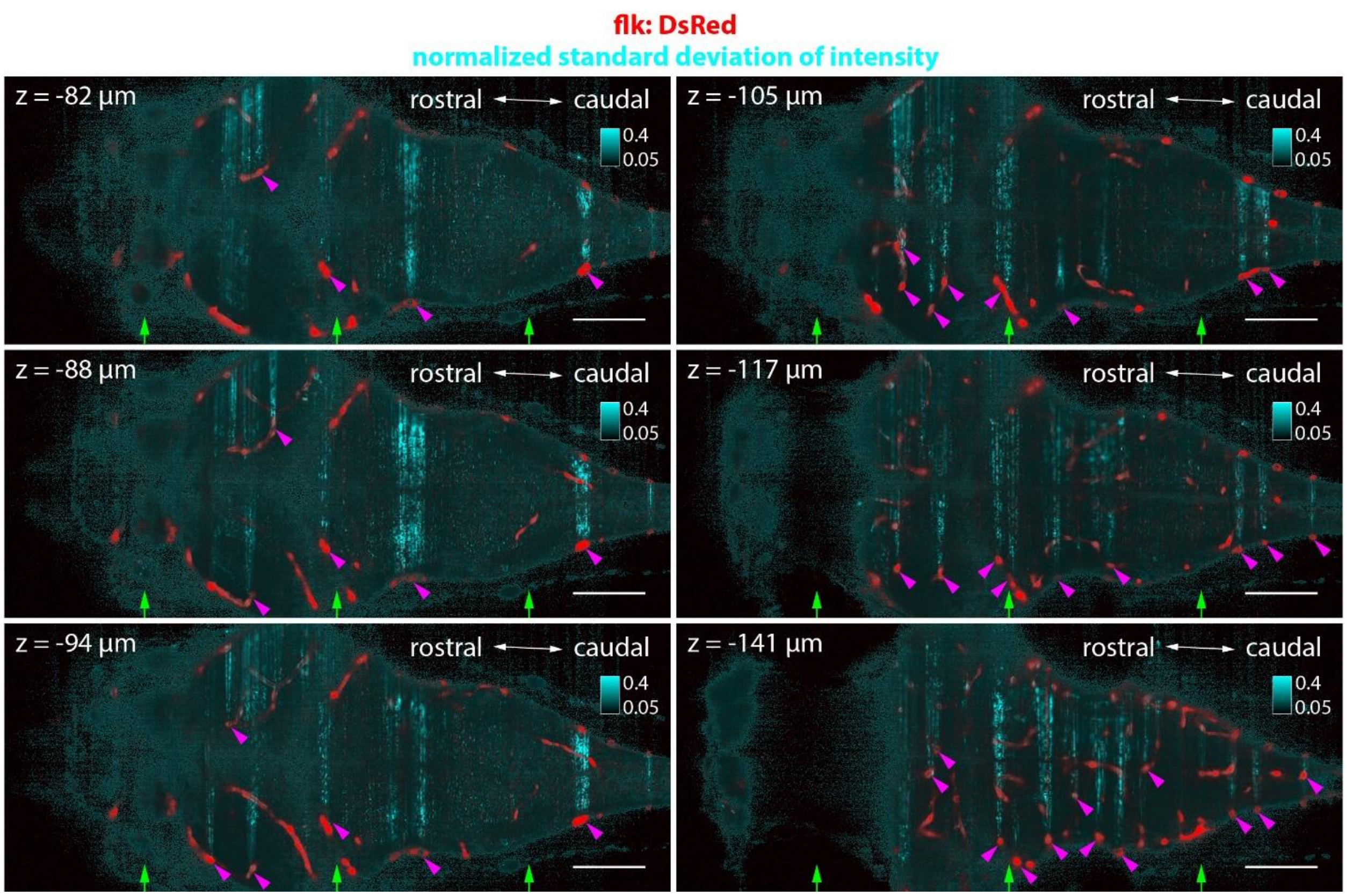

Figure 5 (Figure S15 in the revised manuscript). The spatial correspondence between the blood vessels and the moving stripes suggests that the moving stripe artifacts were caused by blood flow. This figure shows six randomly selected planes (out of 30) of a zebrafish brain that expressed both pan-neuronal Positron2-Kv indicator and a red vascular marker (flk:DsRed). In each plane, blood vessels (red) were overlaid with the map of moving stripes (cyan), which were represented by the normalized temporal standard deviation map (NTSDM, see point **(17)**) of the fluorescence intensity in the GEVI channel. These overlaid images show a consistent spatial relationship, with each moving stripe originating near a corresponding blood vessel (magenta arrows). Green arrows denote the direction of light-sheet illumination. Scale bar: 100 µm.

(17) Normalized temporal standard deviation map (NTSDM) and its rationale. To better visualize the moving stripes' distributions in of the brain, we generated a normalized temporal standard deviation map (NTSDM) of each time-series recording. To obtain this map, we first corrected for photobleaching by normalizing each frame's intensity using the mean intensity of the frame. Then we calculated the standard deviation of each pixel's intensity over time, and normalized this standard deviation map by dividing it by the square root of each pixel's temporal mean intensity, which corresponds to the theoretical shot noise. The rationale behind this calculation is:

The temporal standard deviation (TSD) of a pixel's intensity trace is contributed to by shot noise, real neural activity, moving-stripe induced temporal artifacts, and other noise (e.g., camera noise) whose contribution assumingly remains unchanged during an imaging trial. In our imaging, each pixel within a neuron ROI on average detected ~250 photons per frame, providing a shot noise more than two times of the camera noise (~7e-). Assuming there was no neural activity or moving stripe, each pixel's noise could be approximated using its shot noise. We calculated each pixel's noise using its intensity trace's temporal standard deviation. Therefore, if there were no neural activity or moving stripe, by normalizing the recording's standard deviation map using its shot noise, we would obtain a near solid color map that does not highlight anything. In real imaging experiments, both neural activity and moving stripes cause fluctuations in a pixel's intensity trace, which increases the standard deviation of the trace and the value in the normalized temporal standard deviation map. As the intensity fluctuation caused by moving stripes usually has much higher amplitude compared to the fluorescent changes caused by real neural voltage activity (otherwise the moving stripe will not be a big issue), these moving stripes will stand out in the normalized temporal standard deviation map (NTSDM) as bright stripes along the direction of light-sheet illumination.

We found this NTSDM in general worked well in highlighting the moving stripes in time-series recordings. But this method also has some limitations. It neglects low-intensity pixels where shot noise is not dominant. It also highlights areas that exhibit a lot of neural activity. Its photobleaching correction algorithm might also be improved. Probably due to these limitations, the normalized standard deviation maps of the top and bottom brain layers showed higher background resembling the shapes of brain sections in the raw images (**Figure 4b,** top and bottom rows**)**. Despite these limitations, NTSDMs were sufficient for visualizing the moving stripe locations and comparing moving stripes in the same layer over time. We did not intend to use these maps for any quantitative analysis in this paper. This point has been integrated in highlighted text in **lines 1372-1400** in the revised manuscript, and associated figures.

1c. The high degree of artifacts in the images places a high burden on data processing and analysis. There are two concerns here – 1) with a high number of thin cell diameter stationary stripes, any drift in the sample would lead to changes in the "signal" over time, and 2) the moving stripes inherently resemble neural signals.

- There is no way to evaluate whether the authors have successfully compensated for both issues. One would need a ratiometric method (e.g. dual channel imaging) using a constant signal along with the positron signal to evaluate this.

(18) Regarding concern 1, to eliminate the temporal artifacts caused by sample drift, we paralyzed the fish during imaging and ran motion correction using the NoRMCorre method (Pnevmatikakis and Giovannucci 2017) on our raw videos before extracting the temporal traces. For all the datasets analyzed in this work, the drift in the sample within an imaging trial (35 s) was less than 1 pixel (0.73 µm) on both x- and y-axes, and the drift between two trials was < 2 pixels on both x- and y-axes (**Figure 6**). This drift was corrected before extracting neural signals. In the 3 fish discarded for analysis due to concerns of misalignment, the cross-trial drift was significant (>5 pixels in x- or y-axes), while the within trial drift remained < 1 pixel. This point has been integrated in highlighted text in **lines 800-806** in the revised manuscript, and associated figures.

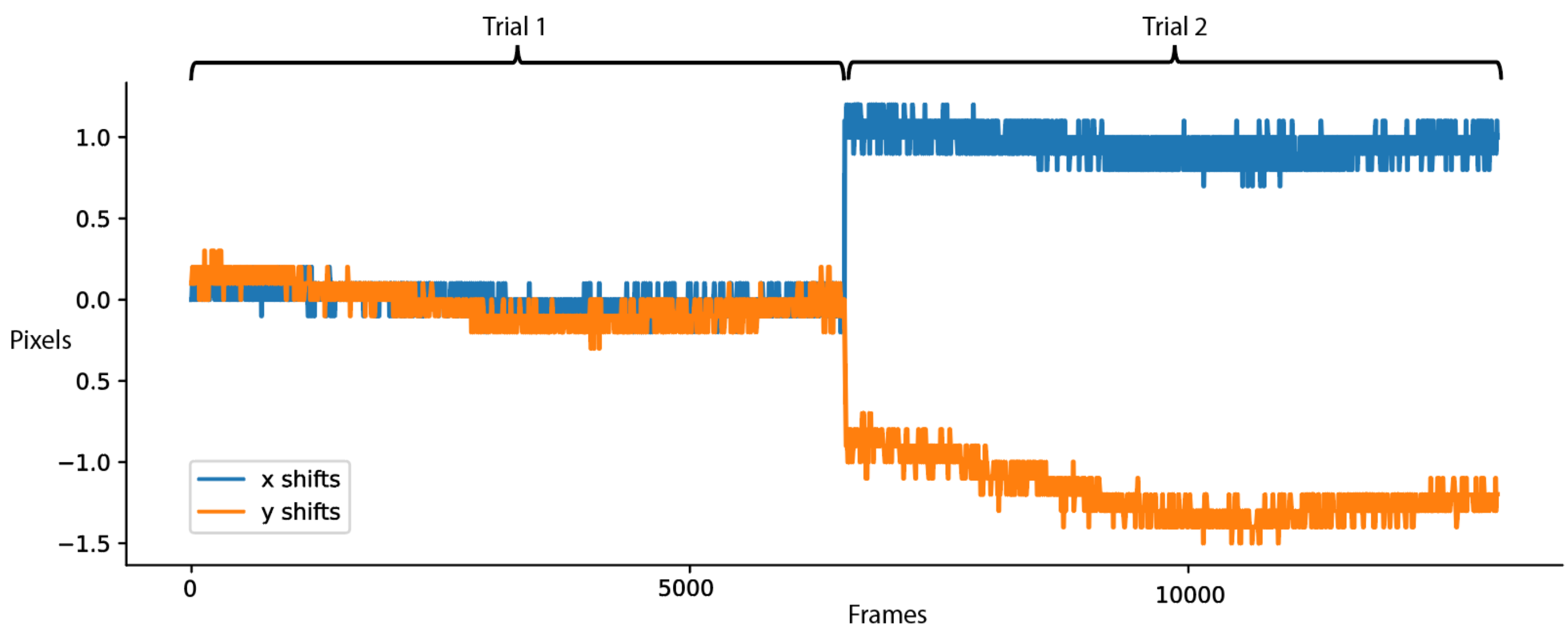

Figure 6 (Figure S20 in the revised manuscript). Motion correction result for a randomly selected brain layer. The corrected drift in at each layer, as measured by the motion correction algorithm NoRMCorre (Pnevmatikakis and Giovannucci 2017), within an imaging trial (35 s) was less than 1 pixel (0.73 µm), and the drift between two trials was < 2 pixels on both x and y-axes. To align the neuron positions of two trials, trial 2 used the first frame of trial 1 as reference for motion correction. The abrupt change of the curve at the start of trial 2 reflects a slight drift of the sample in the x and y axes during the 20-min dark session between the two trials.

(19) Regarding concern 2, we aim to address the stripe artifact issue as early as possible in the process. In the revised paper, we demonstrate an optical approach to remove both the stationary and moving stripe artifacts (**Figure 7**). This approach shares a concept used in earlier works, of using multidirectional illumination (Huisken and Stainier 2007; Sancataldo et al. 2019; Glaser et al. 2018; Ricci et al. 2020). In this experiment, we used a resonant mirror scanner to rapidly pivot the light-sheet in the image plane during each camera exposure. By doing this, the stripe artifacts were dispersed and averaged out over broader areas. In more detail: to efficiently remove stripe artifacts, the light sheet must be pivoted across a sufficiently large angle during a single frame exposure, so that stripe artifacts can be averaged out across sufficiently large areas. One specific challenge we encountered was that our microscope acquires images at a rate of several thousand frames per second—tens of times faster than those reported in previous studies (Huisken and Stainier 2007; Sancataldo et al. 2019; Glaser et al. 2018; Ricci et al. 2020). This high frame rate (and thus short exposure time) requires high pivoting speed, as well as precise timing control of the light-sheet pivoting to prevent frequency aliasing artifacts. To provide sufficient pivoting speed, we employed an 8-kHz resonant mirror scanner (RESSCAN, Sutter Instruments), which has a scan period of 127 µs, to pivot the light sheet. We set the camera exposure time to be the same as the resonant scanner's scan period (127 µs), ensuring that the light sheet completed a full pivoting cycle during each frame exposure, regardless of when the exposure started. Additionally, to prevent temporal artifacts caused by frequency aliasing between the volumetric light-sheet z-scanning and light-sheet pivoting, we synchronized the start of each volumetric z-scan with the start of a pivoting scan.

In our voltage imaging experiments, we deliberately shortened the frame exposure time to minimize motion blur caused by axial (z-axis) movement of the imaging plane during exposure. In our 30-plane, 200-Hz zebrafish brain imaging experiments, we set an exposure time of 40 µs while operating the camera at 6025 frames per second, resulting in an approximately 24% duty cycle for active exposure time. During a 40-µs exposure, the image plane shifted in z-axis by ~2.7 µm. However, as discussed in the previous paragraph, for our stripe removal experiments utilizing a resonant scanner, we had to match the exposure time to the resonant scan period (127 µs) to ensure completion of a full pivoting scan per frame exposure. Therefore, to reduce the motion blur in the stripe removal experiment, we reduced the axial scan range to 120 µm in the zebrafish brain and imaged 20 planes at 203 Hz. This corresponded to a frame rate of 4060 Hz and a 52% duty cycle for the active exposure time, and the focal plane shifted in z-axis by ~6.1 µm during each 127-µs exposure. We would like to note that, in principle, the number of imaged planes can scale linearly with an increase in the resonant frequency. For example, employing a 12-kHz resonant mirror scanner would enable this stripe removal approach to support imaging of $20 \times 12\ \mathrm{kHz}/8\ \mathrm{kHz} = 30$ planes of the zebrafish brain at 200 Hz. The shifting distance of the focal plane in the z-axis could also be potentially reduced using a faster resonant scanner that has a shorter scan period. For example, a 12-kHz resonant scanner has a scan period of 83.3 µs. This would reduce the exposure time and the focal plane shifting distance per exposure by $1 - \frac{83.3}{127} = 34\%$.

In the stripe removal experiments, we imaged 20 planes of a zebrafish brain at 203 Hz for a duration of 50s. During the first 30s, the resonant scanner was on. Then the resonant scanner was turned off for the remaining 20s. **Figure 7** compares the images and the neural traces of the same image planes between the periods when the resonant scanner was on and off. When the resonant scanner was running, both the static and the moving stripe artifacts were eliminated in the zebrafish brain (**Figure 7a,b,d,e, h,i,k,l**). Moreover, neuron activity spikes that were previously obscured by the temporal artifacts from the moving stripes became detectable (**Figure 7c,f,j,m,** red arrows).

As we discussed in point (17), the temporal standard deviation (TSD) can be used to approximate the noise of neural traces contaminated by the moving stripe artifacts, which is the dominant cause of temporal deviation in these traces. To evaluate the performance of our stripe removal approach, we measured the temporal standard deviations (TSDs) of the traces of moving-stripe affected neurons (meaning these neurons were affected by moving stripes when the resonant scanner was off)—as a way to quantify the strength of the moving stripe artifacts—and compared the TSDs with and without light-sheet pivoting. When the resonant scanner was activated, the TSDs of the traces of moving-stripe affected neurons decreased by an average of 42%, and the traces of more than 90% of these neurons showed reduced TSDs (**Figure 7g,n**). Additionally, we noticed that a small subset of neurons (<1% of all imaged neurons, **Figure 7c,f,j,m**,trace 11) that did not exhibit moving-stripe-induced artifacts in their traces when the resonant scanner was off, showed temporal artifacts in their traces when the resonant scanner was active. Why did this light-sheet pivoting approach fail to remove all moving stripe-caused temporal artifacts, or even exacerbate such artifacts in the traces of certain neurons? Conceptually, this light-sheet pivoting approach removes a stripe artifact by dispersing and averaging out the stripe over a broader, fan-shaped region behind the stripe source. Therefore, a larger pivoting angle generally leads to better artifact removal. However, our illumination objective's numerical aperture (NA=0.28) limited our maximum pivoting angle to 32.5 degrees. This angle might not be sufficiently large to eliminate the moving-stripe-induced temporal artifacts in the traces of all affected neurons, particularly those right behind a moving stripe source. Furthermore, by dispersing the moving stripe artifacts into previously unaffected regions, light-sheet pivoting might inadvertently introduce temporal artifacts to the traces of a minority of neurons that were originally artifact-free. In principle, these limitations could be addressed in the future by employing an illumination objective lens with a larger NA.

These points have been integrated in highlighted text in **lines 682-743** in the revised manuscript, and associated figures.

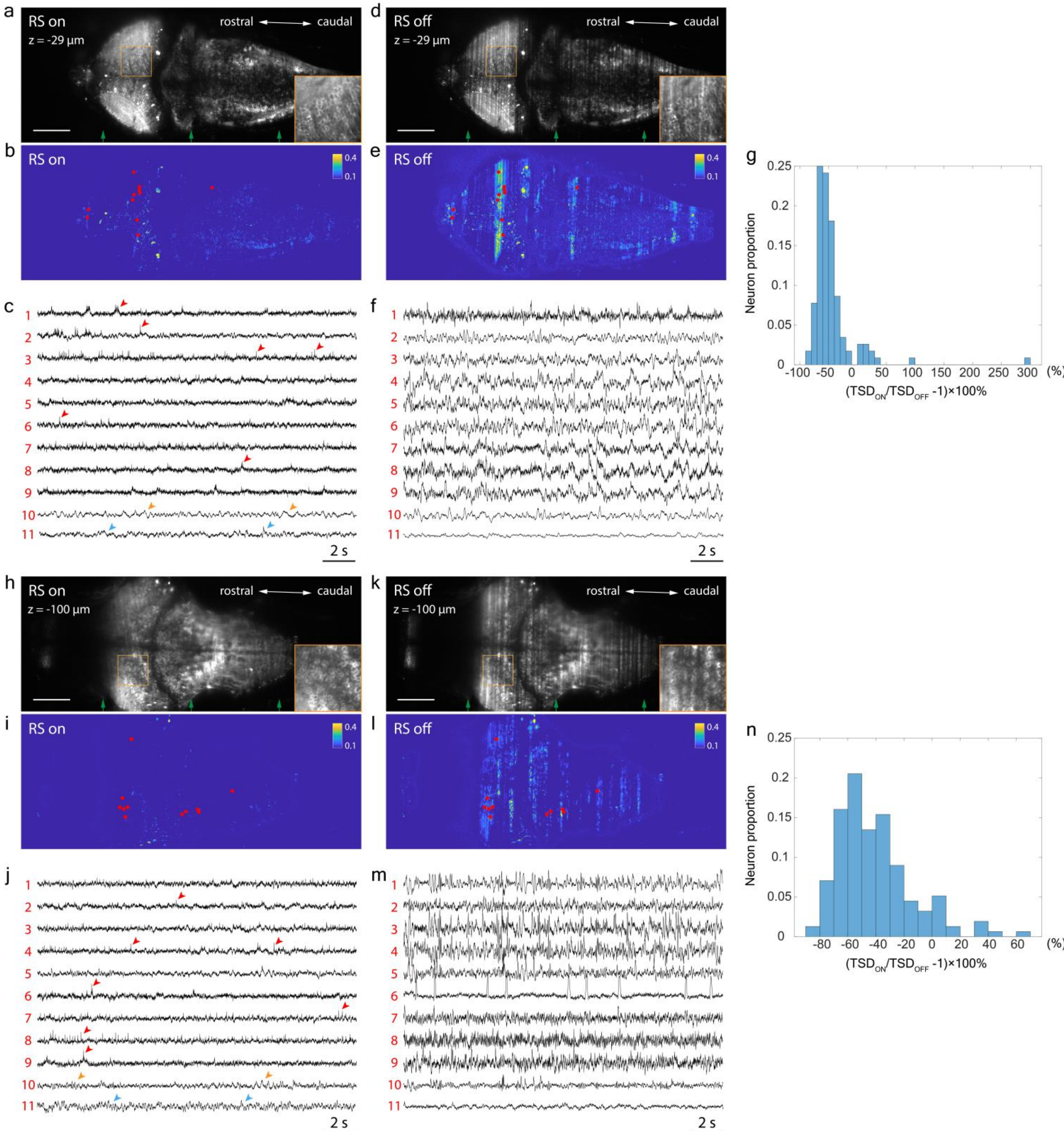

Figure 7 (Figure S16 in the revised manuscript). Removing stripe artifacts using a resonant mirror scanner. (a) Raw image of a randomly selected plane (out of 20) of a zebrafish brain expressing pan-neuronal Positron2-Kv indicator, with resonant scanner scanning (RS on). Green arrows indicate the direction of light-sheet illumination. A magnified view of the orange boxed region is shown in the bottom right corner. Scale bar: 100 µm. **(b)** The normalized temporal standard deviation map (NTSDM) of the brain plane shown in (a). Red spots indicate representative neurons whose traces are shown in (c,f) for comparison between conditions when the resonant scanner was on vs. off. **(c)** Intensity traces of the neurons labeled with red spots in (b). Three kinds of representative traces are shown. Traces 1-9: the resonant scanner scanning (RS on) reduced temporal artifacts in the traces of >90% moving-stripe-affected neurons. Red arrows indicate spikes that became detectable after stripe artifact removal. Trace 10: for less than 10% of the moving stripe-affected

neurons, resonant scanner scanning did not efficiently remove the temporal artifacts in their traces (orange arrows), probably due to the small pivoting angle (32.5 degrees) limited by the numerical aperture (NA=0.28) of the illumination objective lens. Trace 11: a small subset of neurons (<1% of all imaged neurons) that did not exhibit moving-stripe-induced artifacts in their traces when the resonant scanner was off showed temporal artifacts (blue arrows) in their traces when the resonant scanner was active. **(d-f)** Results from the same plane as in (a-c), without resonant scanner scanning (RS off). **(g)** Histogram displaying the distribution of temporal standard deviation (TSD) differences in the traces of neurons with the resonant scanner on (TSD_{ON}) versus off (TSD_{OFF}). Only the neurons within the plane in (a–f) and affected by moving stripe artifacts when the scanner was off were included in this analysis. **(h-n)** Results of the same analysis and comparison as in (a-g), but for a different randomly selected plane of the same zebrafish brain.

(20) We did not implement ratiometric methods, because they may not be able to efficiently correct GEVI baseline intensity fluctuation caused by moving artifacts in our case. This is because the intensity of the reference fluorophore may not scale proportionally to the fluctuations of GEVI intensity, due to fluorescence saturation of either fluorophore, at high-intensity excitation. We estimate that in our experiments, the GEVI reached approximately 40% of its maximum fluorescence intensity. At this level, the GEVI's baseline intensity does not change proportionally to the excitation intensity. Although ratiometric approaches could be a backup plan in the case where moving stripe artifacts cannot be addressed using other approaches, we would like to note that the ratiometric approach is not the ideal solution to correct for moving artifacts, for several additional reasons: 1) First, crosstalk of the reference fluorescence and the GEVI fluorescence could occur due to emission spectrum overlap. It is difficult to find a reference fluorophore that can be efficiently excited with the same excitation light as for the GEVI fluorophore, while having a non-overlapping emission spectrum. Alternatively, the overlapped part of the spectrum could be filtered out using emission filters, but light loss can be limiting in voltage imaging. 2) Second, dual channel imaging requires doubling the number of cameras, increasing the cost, the complexity of the hardware, and the amount of data to analyze. 3) Third, the reference fluorophore will occupy a precious color channel that could otherwise be used for other applications, such as optogenetics, or a fluorescence indicator of a second neural signal (e.g., a neurotransmitter). This point has been integrated as **Supplementary Note 2.**

- The claim that it's safe to remove synchronous signals on the order of 50 ms is odd, given that the authors then show traces in Fig. 3e (orange group) and Fig. 5c (bottom trace), which are clearly in the range of ~50 ms. Is this insufficient removal of contaminating stripe artifacts?

(21) The "synchronous signals on the order of 50 ms" criterion alone is not sufficient to identify ROIs contaminated by moving stripe artifacts. ROIs must also coincide with the moving stripes' locations to qualify for removal. The spatial distributions of the orange group ROIs in **Fig. 3e** in the original manuscript and the bottom trace ROI in **Fig. 5c** in the original manuscript did not align with a moving stripe artifact, nor did we find a group of ROIs that had a stripe shaped spatial distribution while exhibiting synchronous signals to these ROIs. Therefore, we did not exclude these ROIs and their traces in our analysis. We have further clarified this in the revised text (see highlighted text in **line 770-778** in the revised manuscript).

- The authors compensate for the static stripe pattern by multiplying with a constant factor per stripe, (line 425) – they claim this would not complicate spike detection. However, SNR across stripes of varying brightness can be different, which is not taken into account.

(22) We apologize for any confusion associated with our writing. We did not multiply by a constant factor for each extracted trace to compensate for the stationary stripe patterns. We meant that having this stationary stripe pattern in our raw images, was already like scaling the traces that would have been extracted without stationary stripe artifacts, by constant factors. We agree with the reviewer that the noise and SNR (signal-to-noise, defined as the average spike amplitude in a trace divided by the standard deviation of the trace) difference cannot be neglected here. Dark stationary stripes could decrease the SNR of neural traces, and

reduce spike detection accuracy. We have modified this sentence in our manuscript (see **line 612-624** in the revised manuscript, and associated video and figures).

- It would be useful to have a visual of the spatial distribution of all the putative-neuron ROIs (3k out of starting # of ROIs of 25k) – to help ascertain the net effect of the artifacts. Is the distribution spatially even or left-side heavy (as could be deduced from lines 525-28)? It is especially important as sidedness is evoked later to suggest/corroborate UV processing in contralateral OT (line 594-95)

(23) We have included new plots (see **Figure 8a-d**, below) to illustrate the spatial distribution along the lateral/left to right axis of the brain of all putative neurons (defined as all segmented ROIs after removing the artifact-contaminated ones) in four zebrafish brains. Because in our revised paper, we removed the SNR_{VolPy} thresholding step in the analysis to make our analysis approach more generalized and subject to fewer parameters, for each fish we plotted three histograms: one for all putative neurons, and two for putative neurons that passed two SNR_{VolPy} thresholds ($SNR_{VolPy}>2$, $SNR_{VolPy}>4$). The two thresholds were chosen somewhat arbitrarily, but hopefully give the reader a sense of what the data quality is like. Here SNR_{VolPy} is a parameter that VolPy calculates for each extracted neural trace. SNR_{VolPy} is defined as the ratio between the average detected spike amplitude and the standard deviation of the negative portions of the trace filtered by a 15-Hz high-pass filter (Cai et al. 2021). We found almost all (98.4%, n=4 fish) putative neurons had $SNR_{VolPy}>2$ (**Figure 8a-d**), probably because VolPy's denoising and footprint optimization algorithms automatically increased the SNR_{VolPy} of the extracted traces (Cai et al. 2021).

We did find that the one-sided illumination affected the distribution of neurons that we recorded on each side of the brain. The side of light-sheet incidence tended to have more putative neurons, presumably because the light sheet traveled through thinner tissues and maintained better optical quality on this side. In Fish A–D, 59%, 59%, 62%, and 58% of their putative neurons, respectively, were located on the left side of the brain—the side from which the light sheet was delivered. Consequently, we cannot simply attribute the asymmetric neuron distribution of the UV-responding neurons to contralateral UV processing in the optic tectum. We have revised our interpretation of the results in our UV stimulation experiments accordingly (see **lines 514-519, 854-864** in the revised manuscript, and associated figures). In principle, this asymmetric neuron distribution could be ameliorated by implementing dual light-sheet illumination from both sides of the brain.

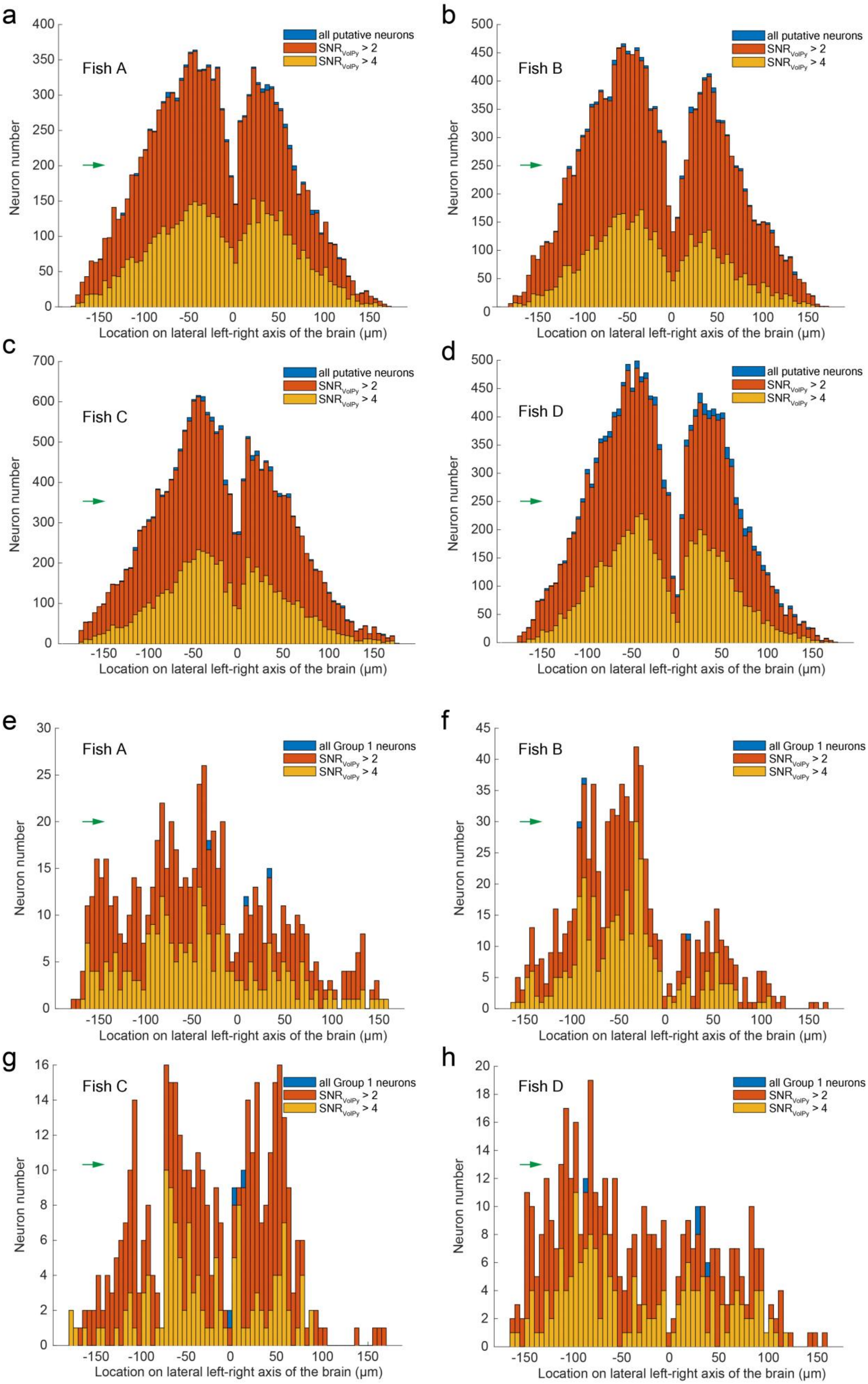
a
Fish A
all putative neurons
SNR VolPy > 2
SNR VolPy > 4
Neuron number
Location on lateral left-right axis of the brain (µm)
b
Fish B
all putative neurons
SNR VolPy > 2
SNR VolPy > 4
Neuron number
Location on lateral left-right axis of the brain (µm)
c
Fish C
all putative neurons
SNR VolPy > 2
SNR VolPy > 4
Neuron number
Location on lateral left-right axis of the brain (µm)
d
Fish D
all putative neurons
SNR VolPy > 2
SNR VolPy > 4
Neuron number
Location on lateral left-right axis of the brain (µm)
e
Fish A
all Group 1 neurons
SNR VolPy > 2
SNR VolPy > 4
Neuron number
Location on lateral left-right axis of the brain (µm)
f
Fish B
all Group 1 neurons
SNR VolPy > 2
SNR VolPy > 4
Neuron number
Location on lateral left-right axis of the brain (µm)
g
Fish C
all Group 1 neurons
SNR VolPy > 2
SNR VolPy > 4
Neuron number
Location on lateral left-right axis of the brain (µm)
h
Fish D
all Group 1 neurons
SNR VolPy > 2
SNR VolPy > 4
Neuron number
Location on lateral left-right axis of the brain (µm)

Figure 8 (Figure S9 in the revised manuscript). Histograms show the distribution of all putative neurons and all Group 1 neurons along the lateral left-right axis of the brains of Fish A-D. (a-d) For each of the Fish A-D, three histograms were plotted: one for all putative neurons, and two for putative neurons that passed two SNR_{VolPy} thresholds ($SNR_{VolPy}>2$, $SNR_{VolPy}>4$). Green arrows indicate the direction of light-sheet illumination. Location 0 on the horizontal axis in these histograms represents the midline of the brain, a boundary between the left and right hemispheres where the density of GEVI-labeled neurons is lower . **(e-h)** The same histograms for all Group 1 neurons of Fish A-D.

- Given the artifacts, the authors should include a negative control (e.g. GFP control) to determine how clean the baseline for activity measurements really is.

(24) To characterize fluorescence baseline, we imaged the brain of a larval zebrafish pan-neuronally labeled with a cytosolic fluorescent marker, Kaede (Tg(HuC:Gal4, UAS:Kaede)) at 200 Hz (**Figure 9**). We chose to use Kaede because we happened to have that fish line, and because the excitation and emission spectra of Kaede matched our current microscope. Kaede fish imaging was performed using the same protocols as during imaging whole brains of Positron2-Kv fish, except that we adjusted the excitation power to match the raw image intensity to that in our voltage imaging experiments. We extracted the average intensity traces of 72 (an arbitrary number) neuron ROIs distributed across a randomly selected brain plane (out of 30). To obtain these ROIs, an experimenter randomly searched around the areas in the brain plane that did not visually overlap with any moving stripe in the raw video and manually annotated them. After correcting for photobleaching by applying a high-pass filter to the raw traces (cut-off frequency: 15 Hz), we calculated the standard deviations of the traces to quantify the measured noise. Moreover, as a reference, we calculated the theoretical shot noise for each trace. To do that, we first calculated the mean intensity of each trace and converted the value to photon number by dividing it by the camera's conversion factor (0.13 in our case). The theoretical shot noise of each trace was calculated as the square root of the photon number. The measured noise of these neurons (quantified by the standard deviation of the traces) was on average 1.58±0.45 (mean ± standard deviation, n=72 neurons) times their theoretical shot noise (**Figure 9b**). The measured noise was on average 0.83±0.28 % (mean ± standard deviation, n=72 neurons) of the baseline intensity (**Figure 9b,c**). This point has been integrated in highlighted text in **lines 520-543** in the revised manuscript, and associated figures.

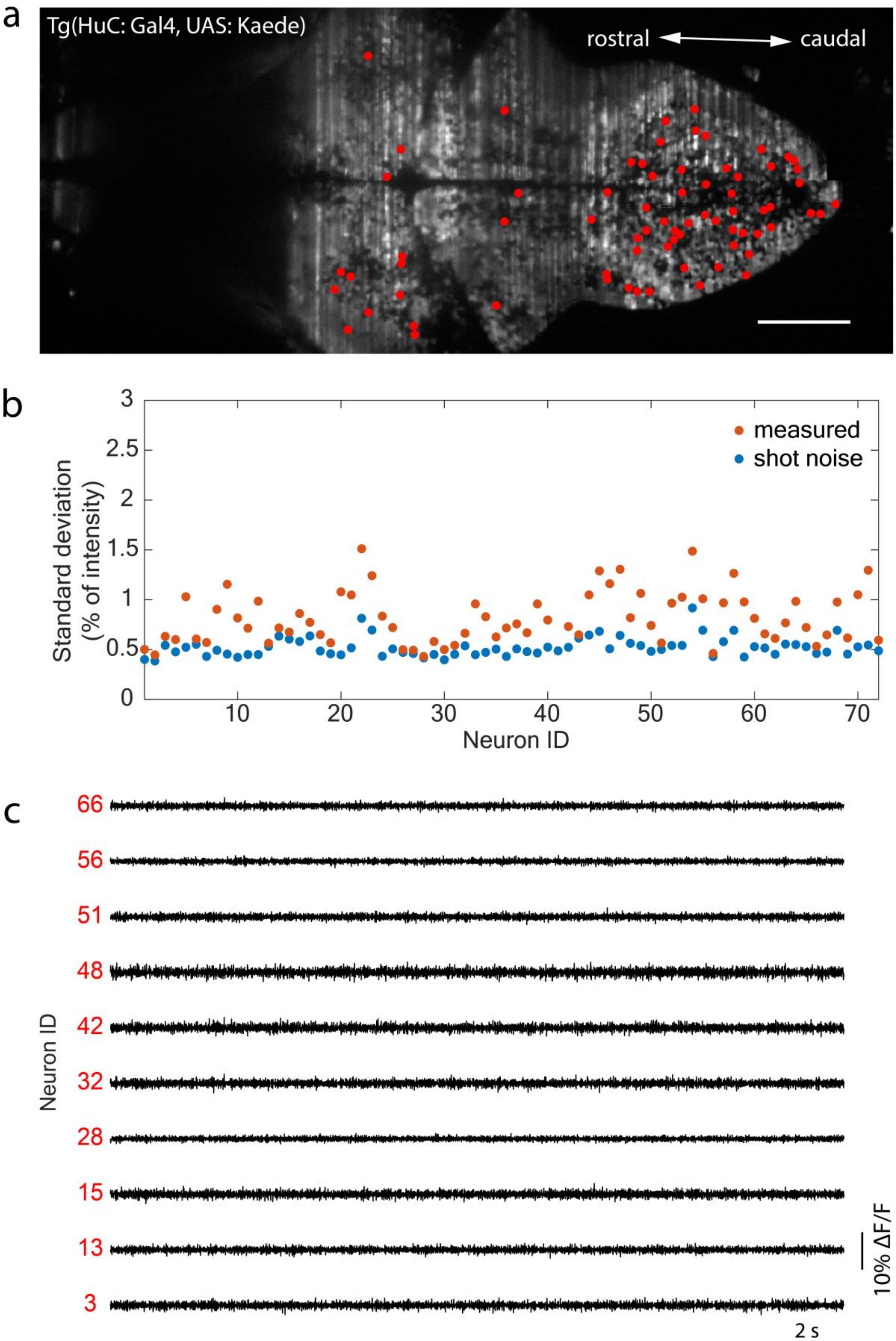

Figure 9 (Figure S12 in the revised manuscript). Quantification of the baseline noise using pan-neuronal Kaede fish. (a) A randomly selected single-plane image (out of 30) of the Kaede fish brain was analyzed. Neurons (red spots)

indicated were randomly selected across the areas in the plane that did not visually overlap with any moving stripe in the raw video, for signal trace analysis. Scale bar: 100 µm. **(b)** To quantify the baseline noise, we calculated the standard deviation of the signal traces of the neurons marked in (a) and plotted the standard deviations (orange dots) as percentages relative to the baseline intensity. The theoretical shot noise of each signal trace was also plotted (blue dots). **(c)** Signal traces of 10 neurons randomly selected from the 72 analyzed neurons.

2. The excitation arm of the microscope is not sufficiently described. The sentence "the sample is excited by a laterally illuminated light sheet (515 nm wavelength) that was generated using a Powell lens and scanned using a galvo mirror (not shown, as these are used in the conventional way)" with no citations (!) is not a sufficient description for a publication, especially for a methods paper, and especially given significant quality issues associated the brain volumes shown in the manuscript (see point #1). Please provide the actual light path and configuration. It's not possible to fully evaluate a microscope with the entire excitation light path missing.

(25) We have included in our revised paper a schematic figure (**Figure 10**, below) of the entire excitation light path, and a detailed description of the excitation arm design and construction (see **Methods** in the revised manuscript**, lines 1094-1137** in the manuscript, and associated figures). The schematic diagram includes two excitation arm configurations: configuration 1 is the excitation arm used for our standard, non-light-sheet-pivoting imaging experiments (**Figure 10a**); configuration 2 is the excitation arm with a resonant mirror scanner to enable rapid light-sheet pivoting, as used in our stripe removal experiments (**Figure 10b**).

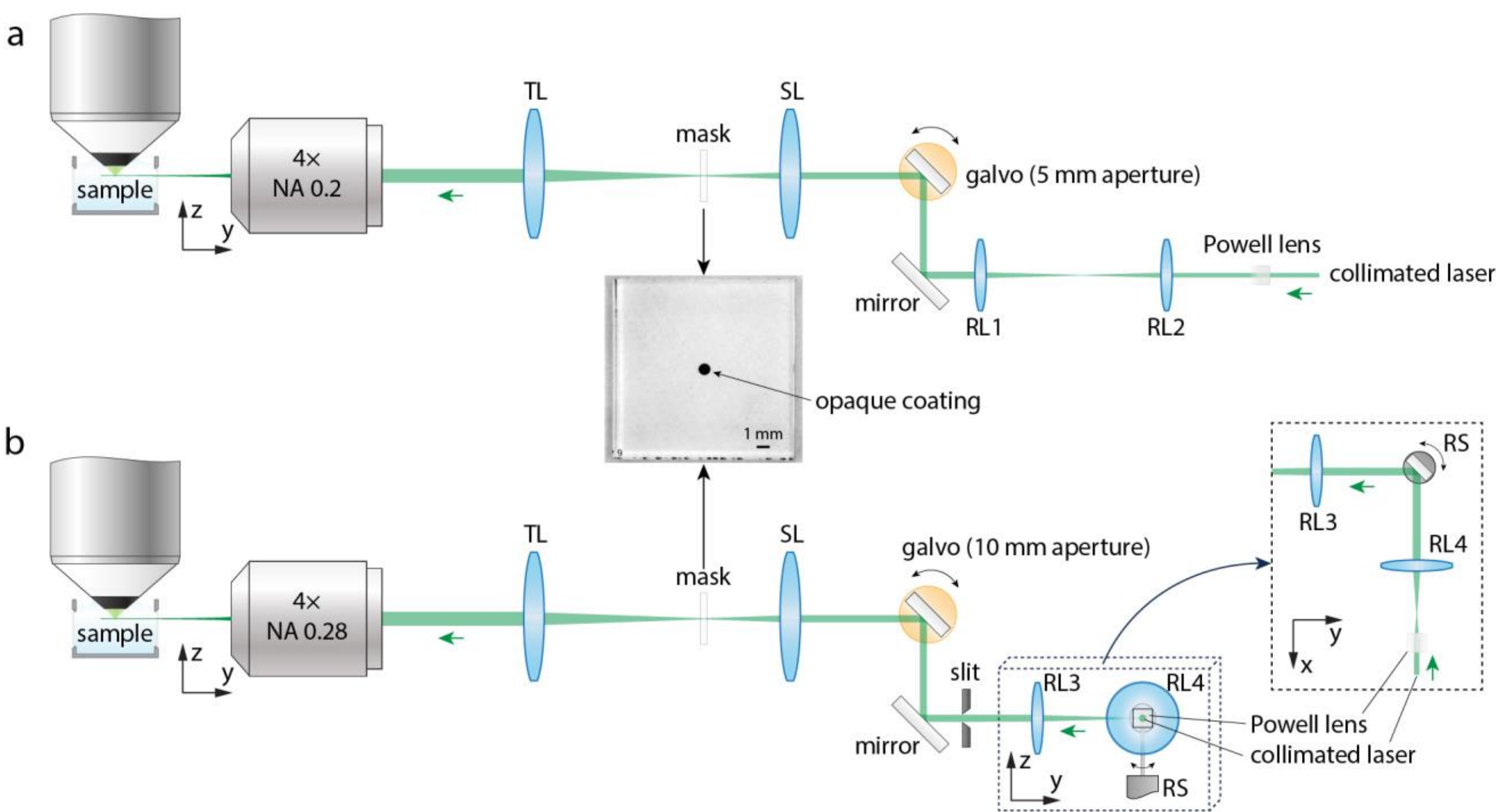

Figure 10 (Figure S17 in the revised manuscript). Schematic diagram of the excitation light path. (a) Excitation light path (configuration 1) without light-sheet pivoting capability. A collimated laser illuminated from the right side, first passing through a Powell lens and then a pair of relay lenses (RL1, RL2). After being deflected by the galvo scanner, the excitation light passed through a scan lens (SL) to form an enlarged light sheet near its focal plane. This enlarged light sheet finally passed through a 0.25× microscope composed of a tube lens (TL) and a 4× objective lens, to illuminate the sample. We placed an optical mask at the scan lens's focal plane to prevent the excitation light from directly entering the fish's eye. **(b)** Excitation light path (configuration 2) with light-sheet pivoting capability for stripe artifact removal. This configuration is in general the same as configuration 1, except that 1) a resonant mirror scanner was inserted between the relay lenses (RL3, RL4) to pivot the light sheet in the focal plane, 2) the galvo scanner had a larger aperture (10mm diameter) to deflect the excitation light that was rapidly scanned by the resonant scanner across ~8 mm range on the galvo scanner, 3) the illumination objective was changed from a Nikon 4x lens (f=50mm, NA=0.2) to an Olympus 4x lens, which has a smaller focal length (45mm) and larger numerical aperture (NA = 0.28), 4) the relay lenses had different focal

lengths (RL3, f=100mm, RL4, f=75mm) to match the aperture size of the resonant mirror scanner, and 5) an adjustable slit was added between the galvo and RL3 to control the thickness and divergence of the light sheet. A top view of the resonant scanner part of the light path, including the resonant scanner, the relay lens pair, and the Powell lens, is in the dashed box on the right side of the panel.

Here is the text description of the excitation arms that we included in the **Methods** section in our revised manuscript:

For fluorescence excitation, we built two versions of the light-sheet excitation arm: the first configuration for standard, non-light-sheet pivoting voltage imaging (**Fig. S17a**) and the second configuration with a resonant mirror scanner to pivot the light sheet for stripe removal (**Fig. S17b**).

In the first configuration (**Fig. S17a**), a collimated laser beam (515nm, beam diameter ~1.5 mm, Cobolt 06-MLD, HÜBNER Photonics) first passed a Powell lens (5 degrees fan angle, Laserline Optics) to form a divergent fan of light in the horizontal plane. This fan beam then traveled through a pair of 4f relay lenses (RL2, f=50mm, Thorlabs, RL1, f=75mm, Thorlabs) and reached a single-axis galvo mirror (mirror aperture=5mm, Saturn 5B, ScannerMAX). The galvo mirror reflected the fan beam to a scan lens (CLS-SL, Thorlabs), which focused the light into a horizontal light sheet at its focal plane. Finally, this light sheet passed through a 0.25×, reversed microscope composed of a tube lens (ITL200, Thorlabs) and an excitation objective lens (Plan Apo Lambda 4x, Nikon) to illuminate the sample. The light sheet illuminated at the sample has an approximate width of 1mm, around 20% larger than the fish brain's length. During imaging, the galvo mirror scanned the excitation beam vertically, resulting in axial shifts of the light sheet at the sample. This galvo mirror can support a sinusoidal scan frequency exceeding 2000 Hz with our desired scan range. To prevent excitation light from directly entering the fish's eyes, we installed an optical mask (3-mm thick glass) with a 900-µm diameter disk-shaped chrome coating (optical density>5, PhotomaskPORTAL) on it at the scan lens's focal plane. The optical mask was made by applying a circular chromium coating with a diameter of 900 µm on one side of a square glass piece. This glass is 3 mm thick and measures 14 mm on each side.

The second configuration (**Fig. S17b**) of the excitation light-sheet arm was constructed based on the first configuration's design. Several modifications were made to enable it to rapidly pivot the light sheet for stripe removal. First, a resonant mirror scanner (resonant frequency=8kHz, RESSCAN, Sutter Instrument) was installed between the two relay lenses (RL3, RL4). Second, a galvo mirror with a larger, 10-mm aperture size (Saturn 9B, ScannerMAX) was used. This larger, 10-mm aperture was required because before the excitation light reached the galvo mirror, it was rapidly scanned by the resonant scanner along the horizontal axis. This made the excitation light rapidly move back and forth across an ~8-mm range on the galvo mirror along the horizontal axis. To ensure the excitation light always was deflected by the galvo mirror, a larger mirror aperture was needed. Third, an illumination objective lens (XLFLUOR4X/340, Olympus) with a larger numerical aperture (NA=0.28) was used, which allowed a larger pivoting angle. Fourth, we changed the focal lengths of the relay lens pair (RL3, f=100mm, achromatic doublet, Thorlabs; RL4, f=75mm, achromatic doublet, Thorlabs), to match the excitation beam size to the resonant scanner's aperture. Finally, an adjustable mechanical slit (VA100CP, Thorlabs) was installed between RL3 and the galvo mirror to control the thickness and divergence of the light sheet. This slit was not required for the first light-sheet arm configuration as the light sheet's thickness and divergence was already optimized for larval zebrafish by carefully selecting the focal lengths of the optical elements. However, in the second light-sheet arm configuration, we found slightly reducing the slit width, thus the light-sheet divergence, enhanced the image quality. This is probably because in our stripe removal experiment, the focal plane shifted by a larger axial distance of 6.1µm during each exposure. So compared to using the first light-sheet arm configuration, the motion blur was more prominent. When imaging the zebrafish brain, at locations near the light-sheet waist (the thinnest location), the motion blur became a more limiting factor and consequently the light-sheet thickness became a less limiting factor. This gave us some room to reduce the light-sheet's thickness at locations far from the light-sheet waist within the sample, where light-sheet thickness was a more limiting factor for image quality, by slightly reducing the light-sheet divergence, at the cost of increasing the thickness near the light-sheet's waist.

3. Two obvious alternatives to the strategies used in this manuscript are light field and SCAPE. Their relative merits to the proposed LSM are not sufficiently or correctly explained.

- Light field is not discussed in the introduction and has already claimed to achieve a similar level of optical performance (including previous publications by the Boyden lab) -- XLFM and SDXLFM have both previously claimed to image a similar number of cells across the entire zebrafish brain with volume rates of 100 Hz. A much more detailed discussion of the relative merits of these systems should be included in the introduction, to motivate the present study. Along the same lines, in what way are the experiments and analyses presented in this study not possible with existing LFM methods? This should be included in the Discussion section.

(26) We added a section in our manuscript (see highlighted text in **lines 52-75** in our revised manuscript, also the quoted text below) to discuss in detail the potentials and limitations of light-field microscopy (LFM) setups for voltage imaging, and their relative merits vs. those of our setup. In brief, at present, LFM will be suitable for imaging the voltage of sparsely labeled neurons in a large volume. However, it will suffer from inherently low SNR when imaging densely labeled samples, such as the larval zebrafish brain pan-neuronally labeled with GEVIs. The low SNR is caused by increased shot noise, which stems from the mixing of fluorescence signals of different z-planes of the sample in raw light-field images. This increased shot noise is inherent and cannot be compensated for using SDXLFM. To image the entire larval zebrafish brain with dense labeling, we estimate that SDXLFM will have ~4✕ higher shot noise compared to our current light-sheet microscope.

Here is the section we added in the introduction section (**lines 52-75,** highlighted text) of our revised paper, on the potential and limitations of light-field microscopy (LFM) for voltage imaging:

Light-field microscopy (LFM) (Levoy et al. 2006; Prevedel et al. 2014) holds great promise for population-level voltage imaging (Bai et al. 2024) due to its high volumetric imaging rate, single-shot acquisition capability, compatibility with widefield illumination, and simple hardware setup. However, applying LFM to whole-brain voltage imaging in larval zebrafish presents two key challenges, both stemming from the intrinsic properties of LFM. The first challenge relates to LFM's spatial resolution. In voltage imaging, the fluorescent signals emanate specifically from neurons' membranes, which appear as cell contours in raw images. These neuronal contours contact each other in a densely labeled zebrafish brain, necessitating a spatial resolution much smaller than the neuronal cell body size (3-7 µm) (Kunst et al. 2019) to distinguish individual neurons labeled with GEVIs. Current light-field techniques can achieve at best a lateral resolution of approximately 3 µm when capturing the entire ~200-µm thick larval zebrafish brain in a single snapshot (Cong et al. 2017; Yoon et al. 2020). This limitation comes from an inherent trade-off in LFM between its spatial resolution and axial field-of-view. While this resolution can be improved by combining LFM with optical sectioning (Zhang et al. 2021) and galvo scanning (Wu et al. 2021), doing so requires multiple snapshots to capture the whole zebrafish brain, which significantly reduces the volumetric imaging rate. The second challenge for LFM in voltage imaging is its low signal-to-noise ratio (SNR). The low SNR is caused by increased shot noise, which results from the mixing of fluorescence signals of different sample z-planes in raw light-field images. This increased shot noise is inherent and cannot be efficiently compensated for. To image the entire larval zebrafish brain with dense labeling, we estimate that LFM will have more than 4× shot noise compared to a light-sheet microscope. (A rough estimate is, to extract signals of a neuron, the baseline fluorescence in LSM contains the fluorescence of no more than two layers (~6 µm thick per layer, but a neuron may reside at the interface of two adjacent layers, which have a total thickness of ~12 µm). As a comparison, the baseline fluorescence in LFM contains superimposed fluorescence from the whole brain volume (~200 µm thick), resulting in shot noise that is at least $\sqrt{200/12} \sim 4.08$ times of that in LSM.)

- The introduction of SCAPE is somewhat misleading. The original conception of SCAPE suffers from significant light loss. However, since the development of the refractive index change strategy and the

commercial glass-tip objective lens, light loss is now only ~10%, which is significantly better than the 50% light loss in the LSM remote focusing strategy used in this study. The statement that the glass-tipped objective is not appropriate for large FOV imaging because only the center 450 um is diffraction limited is misleading. The pixel resolution achieved in this LSM implementation is ~730 nm, which is also not diffraction-limited. Optical resolution beyond the spatial resolution set by the camera sensor is empty resolution.

(27) It is true that our current setup loses 50% light (assuming the light is fully unpolarized) at the polarized beam splitter (PBS), resulting in a light efficiency ~40% lower than SCAPE with the glass-tipped objective (the “Snouty” v2). As we mentioned in our original manuscript, the lost 50% light could, in principle, be recovered by adding a second remote scanning module (i.e., a remote objective, a piezo bender scanner, and a remote mirror). We did not install the second module in our original work because we did not find the image brightness to be limiting in the experiment conducted. Of course, enhanced light efficiency would support higher SNR, reduced excitation power, and slower photobleaching. Therefore, in the revised manuscript, we demonstrated dual remote scanning, and increased the fluorescence intensity by 75% in zebrafish brains (**Figure 12b,e**, please see **Supplementary Table 2** for the imaging conditions for each dataset). The images formed from each of the two remote scanning modules could be aligned on the image sensor with sub-pixel precision across a 200-µm axial range (**Figure 11**). The enhancement in fluorescence intensity was less than 100% because the emission light of GEVIs was partially polarized due to the linearly polarized excitation light (this is because linearly polarized light can more efficiently excite transition dipoles of GEVI fluorophores that align with the excitation light’s polarization axis (Phil Brooks et al. 2024)), and we oriented the PBS to guide the emission light with the same polarization axis as the excitation light to the first remote scanning module. In other words, in our voltage imaging experiments using a single remote scanning module, more than 50% (1/1.75 ~ 57%) emission light was already utilized at the polarized beam splitter.

To ensure alignment of images from the two modules on the image sensors during volumetric imaging, we implemented a two-step calibration process using a 3D fluorescent bead sample. In the first step, we adjusted the X-Y positions (perpendicular to its optical axis) of the second remote objective lens to align the two fluorescent bead images across a series of static z-planes (**Figure 11**). This alignment achieved sub-pixel accuracy across a 200-µm thick volume. In the second step, while the microscope was running, we iteratively adjusted the local values of the second piezo bender’s driving signals to synchronize the image focus of the second module with that of the first module, on a plane-by-plane basis. This step resembled the process to synchronize the axial scanning of the light sheet with the movement of the first remote mirror (**see Methods**).

This point has been integrated in highlighted text in **lines 139-143, 243-258, 1138-1148** in the revised manuscript, and associated figures.

(28) We agree with the reviewer’s point that the glass-tipped objective would be appropriate for imaging a larger FOV beyond the center 450 µm when the system resolution is bounded by the pixel size instead of diffraction. We updated that statement in our manuscript (see highlighted text in **lines 129-130** in the manuscript). Indeed, theoretically, SCAPE could be updated to image the whole larval zebrafish brain at the same speed as our setup, with additions such as the same dual-camera system. Indeed, if the hardware is sufficiently optimized, perhaps SCAPE would be a leading candidate to be updated for voltage imaging in the future. We chose our current configuration over SCAPE, at the time, because our configuration theoretically promised less aberration, and samples the fish brain in a way closer to conventional LSMs (Ahrens et al. 2013; Vladimirov et al. 2014; Dunn et al. 2016; Mu et al. 2019; X. Chen et al. 2018). But, as noted, after optimization, SCAPE could be adapted for imaging voltage activity across whole zebrafish brain, in the future. We modified text in the introduction in the revised manuscript to illustrate these principles (see highlighted text in **lines 129-130, 134-136** in our revised manuscript).

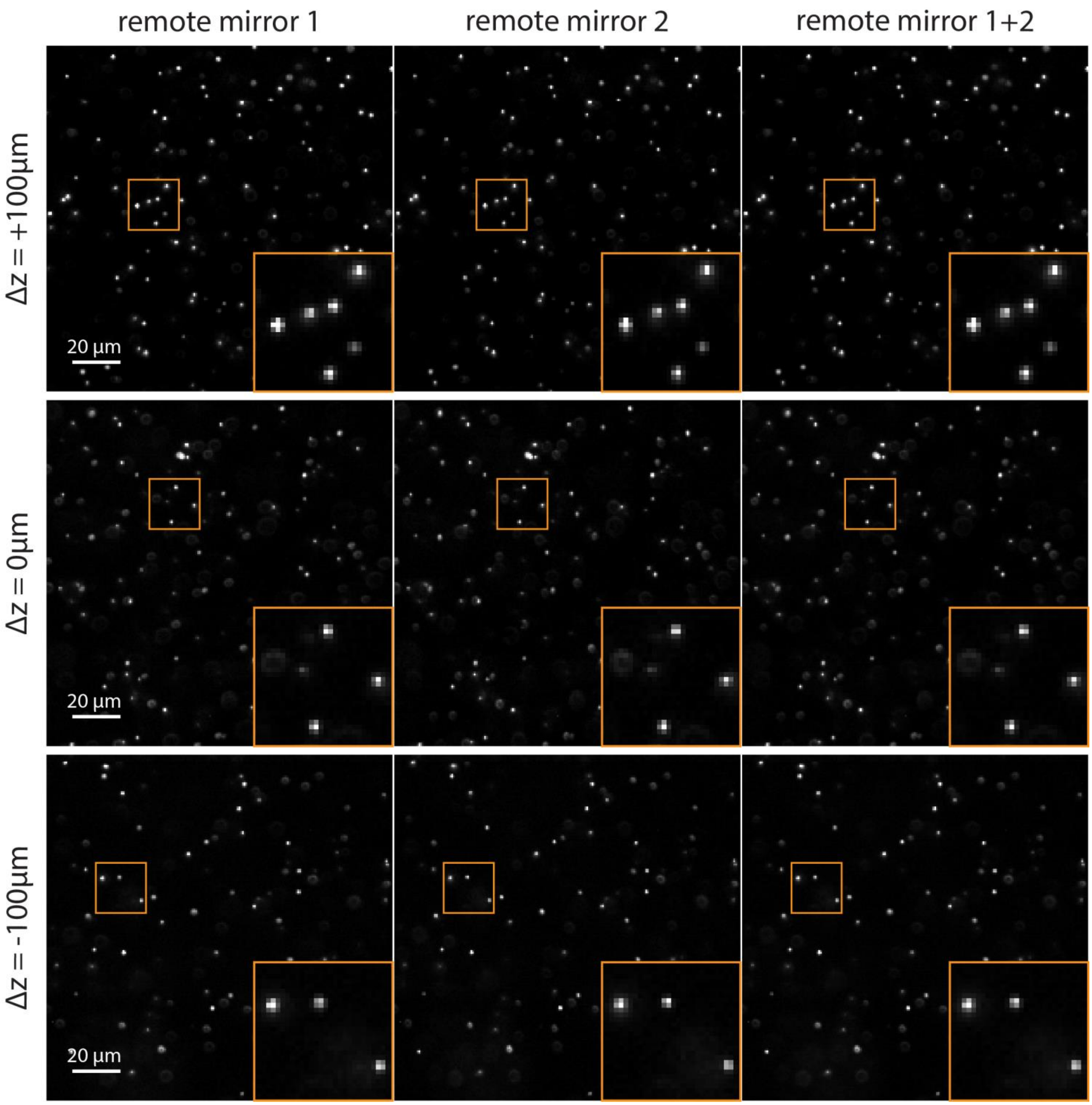

Figure 11 (Figure S1 in the revised manuscript). High-precision alignment of images from two remote scanning mirrors across a 200-μm axial field-of-view. Left column, images of a fluorescent bead sample captured at three axial positions (top, Δz = +100μm, middle, Δz = 0μm, bottom, Δz = -100μm) using remote scanning mirror 1. Middle column, images of the fluorescent bead sample captured at the same axial positions as the left column using remote scanning mirror 2. Right column, overlay of images of the fluorescent bead sample at the three axial positions from both remote scanning mirrors. A magnified view of the smaller orange boxed area is shown in the bottom right corner of each image. Image intensities were normalized for display.

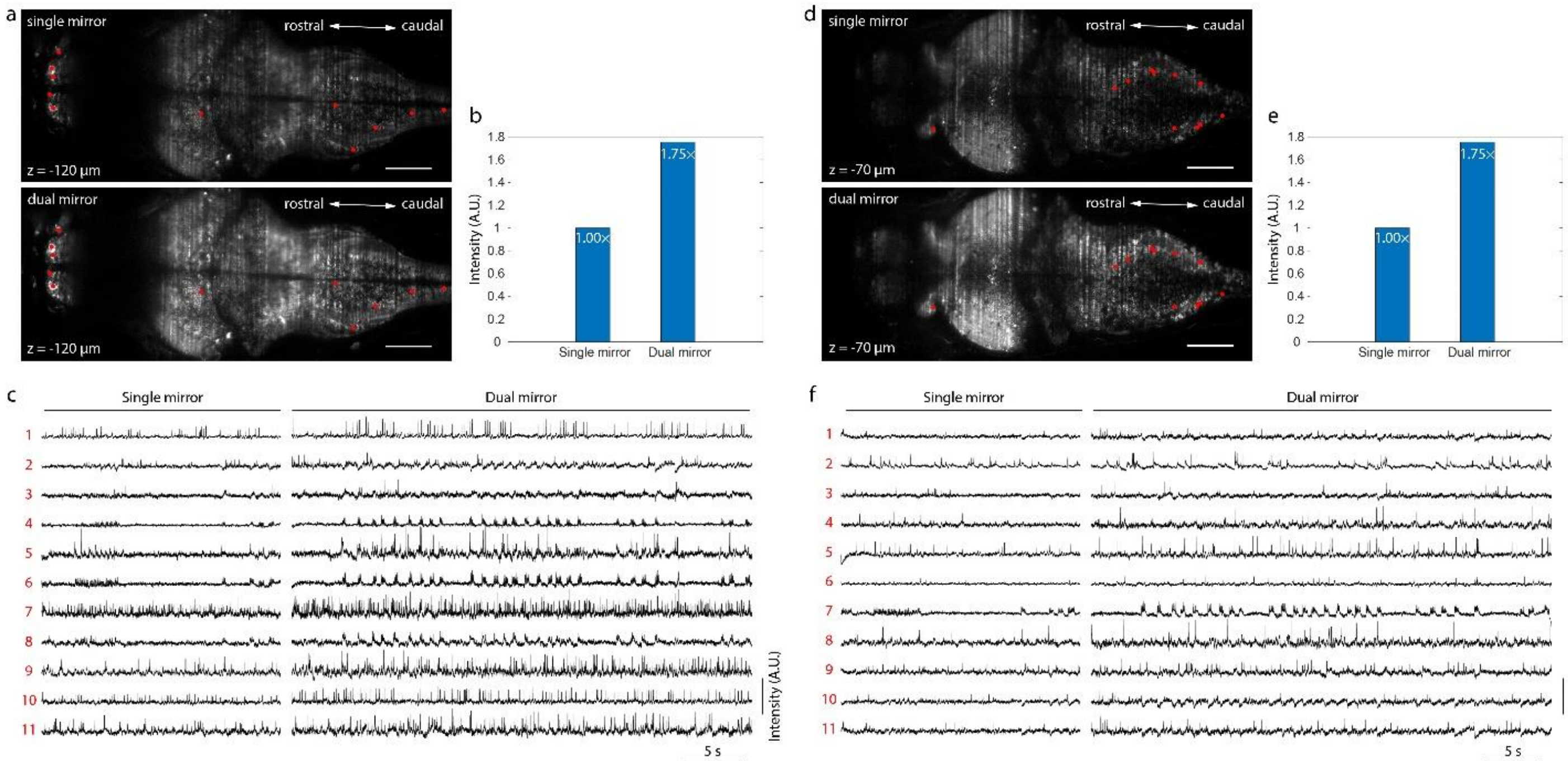

Figure 12 (Figure S2 in the revised manuscript). Dual remote scanning mirrors enhanced image intensity and signal amplitudes. (a) Raw images of a randomly selected zebrafish brain plane (out of 30) acquired with single (top) and dual (bottom) remote scanning mirrors. Red spots mark 11 randomly selected neurons in the plane exhibiting neural activity. Scale bar: 100 µm. **(b)** The use of dual remote scanning mirrors increased the image intensity of this plane by 75%. **(c)** Intensity traces of the same neurons (highlighted by red spots in (a)) were compared between using single and dual remote scanning mirrors. **(d-f)** The same analysis as in (a-c) was performed for another randomly selected plane of the same zebrafish brain.

4. As mentioned earlier, the current manuscript has not achieved the goal of whole-brain spike-resolution imaging at cellular resolution. The authors are recording 1/3 of the brain (i.e. not the majority of the brain) at 243 vps, which can potentially miss a significant number of spikes given the fast kinetics of the sensor. Given that achieving voltage recording across a large portion of the brain is still valuable, there's no reason to overstate the case. Furthermore, statistics on how many spikes are estimated to be missing should be provided.

(29) We amended the manuscript to avoid any overinterpretation of claims (see highlighted text in **lines 32, 169-170, 388, 923, 1012, 1046, 1057, 1085-1086, 1265, 1270** in our revised manuscript).

(30) We quantified the percentage of missed spikes by analyzing the neural voltage activity in a single z-plane video recorded at 1000 Hz with a 40-µs exposure time. We downsampled the 1000-Hz video by extracting a 200-Hz discrete frame series from the video, and quantified how many spikes were missed in the downsampled video compared with the 1000-Hz video. After excluding neurons showing no spikes and neurons affected by moving stripe artifacts, traces of a total of 201 neurons in the z-plane were analyzed. Note we did not count the neurons showing no spikes, which had 'perfect' spike detection in a sense. On average, 30.0% of the spikes (out of all spikes of the 201 neurons) observed in the 1000-Hz traces were missed in the 200-Hz traces (**Figure 13**). This is not surprising as the Positron2-Kv indicator we used has sub-millisecond rise and fall times. As we discussed in our original manuscript, to reduce spike loss, we could potentially use GEVIs with slower kinetics. In the revised paper, we validated this approach through simulation and experiments (**Figure 14**). In the simulation, we compared the detected spikes reported by ASAP5-Kv (Hao et al. 2024), which has ~2.6-ms depolarization and ~4.3-ms repolarization time constants at room temperature, between 1-kHz and 200-Hz imaging rates (see **Methods** in the manuscript). The results show that ASAP5-Kv significantly reduced the chance of missing spikes in the 200-Hz traces: 99.7% of all the spikes observed in the

1-kHz traces (n=201 neurons) were reliably retrieved from the 200-Hz traces (**Figure 14c**). Experimentally, we also imaged transiently expressed ASAP5-Kv indicator in larval zebrafish at 1000 Hz with a 40-µs exposure time. Because the expression was transient and sparse, we only captured several neurons with spike activity. But still, the spikes observed in the 1000-Hz traces were well preserved in the 200 Hz traces, which were obtained by extracting discrete, evenly spaced points in the 1000-Hz traces (**Figure 14d,e)**. In the future, a voltage indicator that combines the kinetics of ASAP5-Kv and the brightness of Positron2, used in a stable panneuronal fish line, could potentially be used to prevent spike loss while maintaining high SNR.

For simulation of ASAP5-Kv neural traces at different imaging rates, we used the spikes detected in the 1000-Hz Positron2-Kv fish imaging data as the ground truth. To simulate ground truth voltage traces, we convolved these spikes with an action potential curve generated using the Hodgkin–Huxley model. Each action potential curve has a duration of 0.5 ms (full width at half maximum). We chose this short 0.5 ms duration to represent faster action potentials, as action potentials with longer durations generate wider fluorescent spikes and are easier to detect. To model the fluorescent traces in response to these voltage traces, we used the kinetic parameters of ASAP5-Kv at room temperature according to (Hao et al. 2024). These parameters include the fast depolarization time constant $\tau_{D,fast} = 2.6\ ms$, the slow depolarization time constant $\tau_{D,slow} = 45.3\ ms$, the fast repolarization time constant $\tau_{R,fast} = 4.3\ ms$, and the slow repolarization time constant $\tau_{R,slow} = 55.9\ ms$, along with their respective proportions in the biphasic depolarization and repolarization processes. We calculated the fluorescent traces $F(t)$ using the following formula with a time step of 0.1 ms.

$$F(t) = \int_0^t \frac{dV(\tau)}{d\tau} \cdot R(t-\tau)d\tau$$

$$R(t) = \begin{cases} 1 - a_1 \exp\left(-\frac{t}{\tau_{D,fast}}\right) - (1-a_1)\exp\left(-\frac{t}{\tau_{D,slow}}\right), if \frac{dV}{dt} \geq 0 \\ a_2 \exp\left(-\frac{t}{\tau_{R,fast}}\right) + (1-a_2)\exp\left(-\frac{t}{\tau_{R,slow}}\right) - 1, if \frac{dV}{dt} < 0 \end{cases}$$

Here $V(t)$ is the simulated voltage traces, t is time, and a_1 (0.92) and a_2 (0.75) denote the proportions of the fast exponential components in the depolarization and the repolarization processes, respectively. We assumed ASAP5-Kv's fluorescence to be linearly related to the neural voltage and set the scaling factor to 1. The 1000-Hz and 200-Hz traces were generated by sampling discrete points in the high temporal resolution (0.1 ms) traces at 1-ms and 5-ms intervals, respectively. Prior to spike detection, we added Gaussian noise to the 1000-Hz and 200-Hz traces, with the noise's standard deviation equal to 1/10 of the maximum spiking amplitude of the traces.

This point has been integrated in highlighted text in **lines 470-492, 1236-1240, 1402-1423** in the revised manuscript, and associated figures.

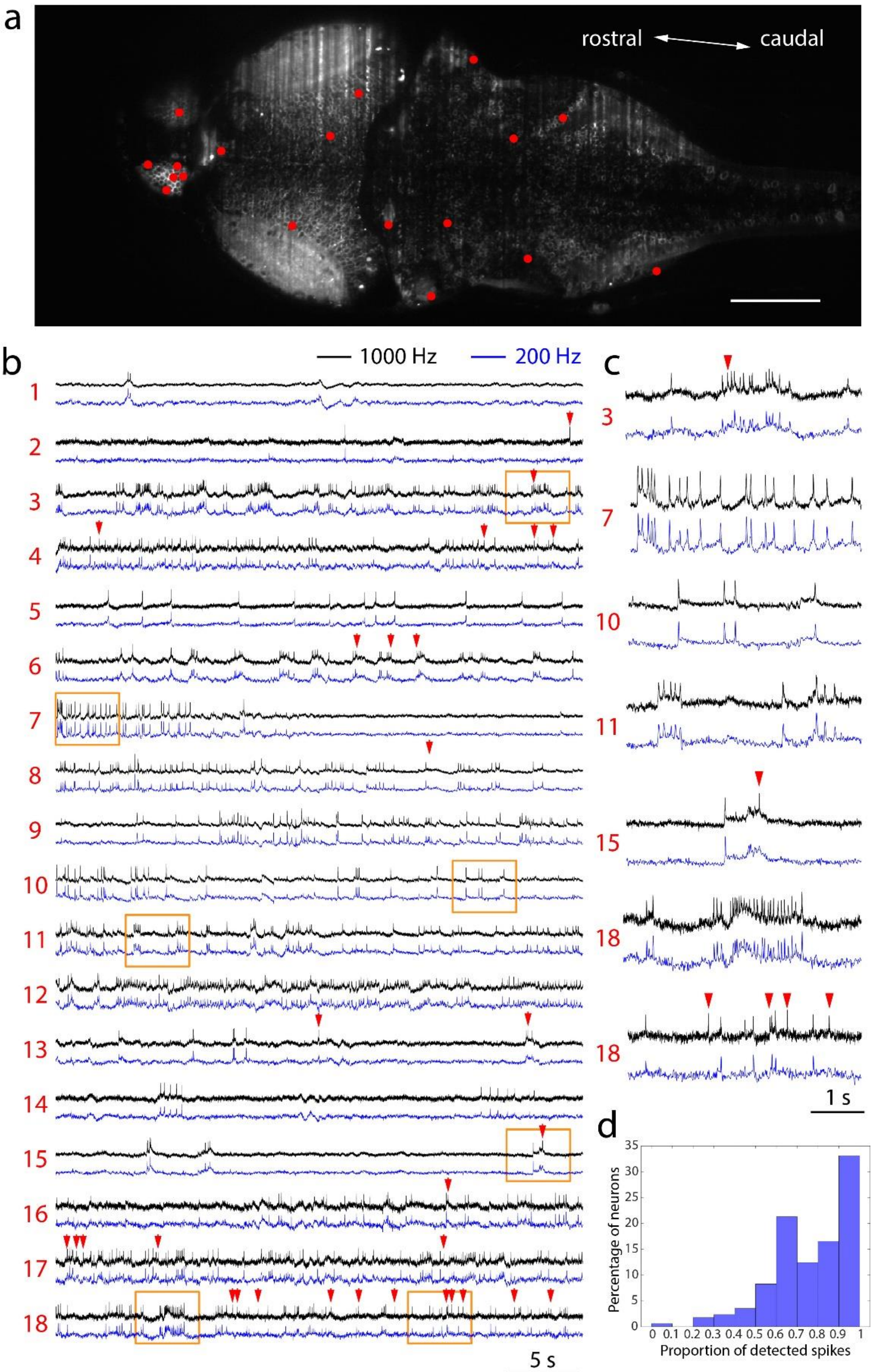
a
rostral
caudal
b
1000 Hz
200 Hz
1
2
3
4
5
6
7
8
9
10
11
12
13
14
15
16
17
18
5 s
c
3
7
10
11
15
18
18
1 s
d
Percentage of neurons
Proportion of detected spikes
0 0.1 0.2 0.3 0.4 0.5 0.6 0.7 0.8 0.9 1
0
5
10
15
20
25
30
35

Figure 13 (Figure S6 in the revised manuscript). Quantification of spike detection rate using the Positron2-Kv indicator at a 200-Hz imaging rate. (a) Raw image of an imaged zebrafish brain plane. Red spots mark randomly selected neurons (out of a total of 201 neurons that showed spikes and were not affected by moving stripe artifacts) whose traces are shown below in (b). Scale bar: 100 µm. **(b)** Neural traces extracted from the original recording at a 1-kHz imaging rate with a 40-µs exposure time (black traces) are compared with the neural traces extracted from a 200-Hz video downsampled from the 1-kHz recording. Red arrows indicate locations where spikes were missed in the 200-Hz traces. Orange boxes highlight regions magnified in (c). **(c)** Magnified views of the orange-boxed regions in (b). **(d)** Histogram showing the percentage of neurons categorized by their spike detection rates (defined as the number of detected spikes divided by the number of all spikes) in the 200-Hz neural traces.

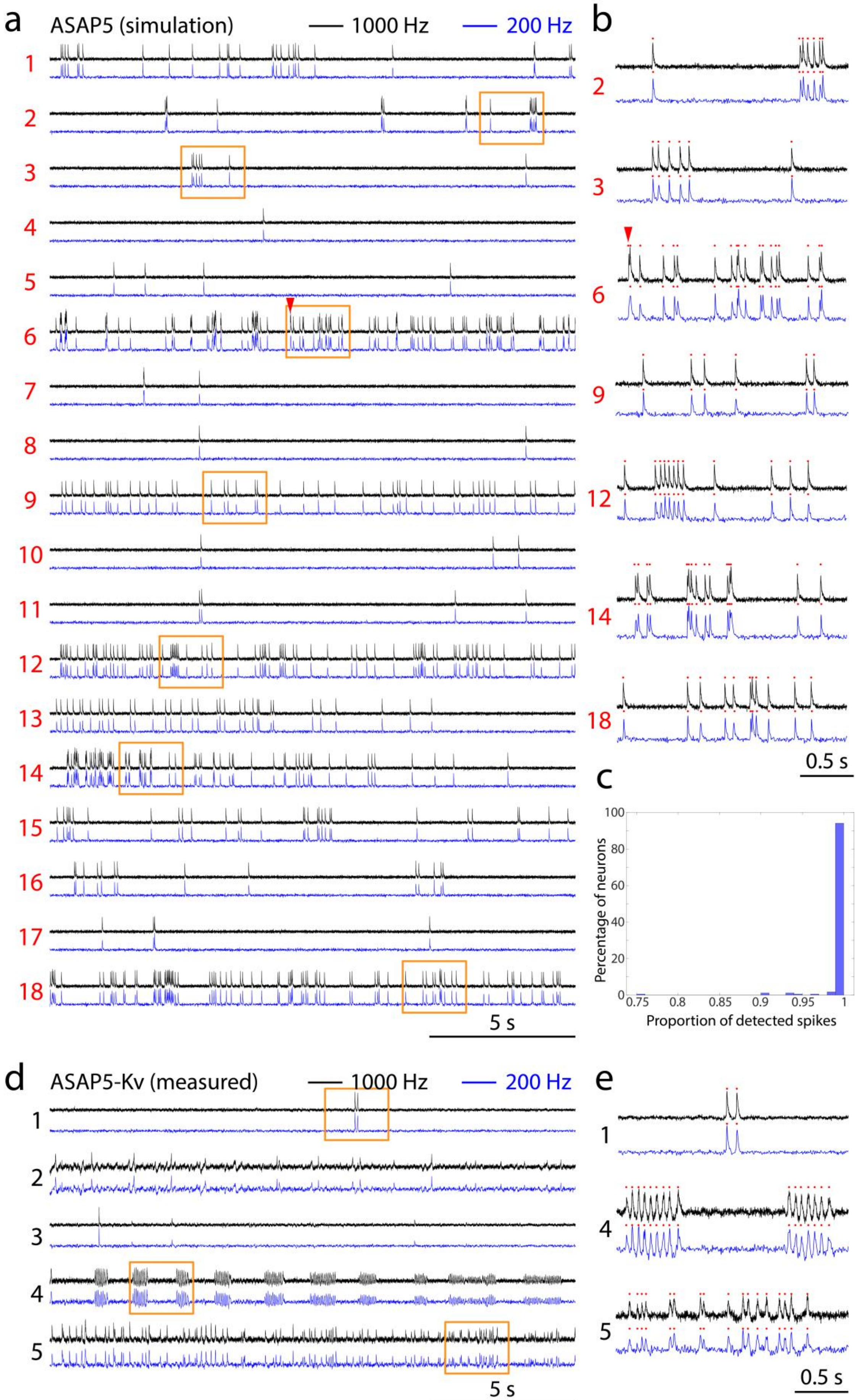

a
ASAP5 (simulation)
1000 Hz
200 Hz
1
2
3
4
5
6
7
8
9
10
11
12
13
14
15
16
17
18
5 s
b
2
3
6
9
12
14
18
0.5 s
c
Percentage of neurons
100
80
60
40
20
0
0.75
0.8
0.85
0.9
0.95
1
Proportion of detected spikes
d
ASAP5-Kv (measured)
1000 Hz
200 Hz
1
2
3
4
5
5 s
e
1
4
5
0.5 s

Figure 14 (Figure S7 in the revised manuscript). Quantification of spike detection rate using the ASAP5-Kv indicator at a 200-Hz imaging rate. (a) Simulated neural traces at a 1-kHz imaging rate (black traces) are compared with the neural traces down sampled to a 200-Hz imaging rate. The red arrow indicates a location where a spike was missed in the 200-Hz traces. Orange boxes highlight regions magnified in (b). All traces were flipped vertically and normalized by their maximum amplitude for display. **(b)** Magnified views of the orange-boxed regions in (a). Red dots indicate detected spikes. **(c)** Histogram showing the percentage of neurons categorized by their spike detection rates in the 200-Hz neural traces, based on simulation results. **(d)** Measured ASAP5-Kv neural traces in larval zebrafish at a 1-kHz imaging rate (black traces) are compared with the neural traces down sampled to a 200-Hz imaging rate. All traces were flipped vertically and normalized by their maximum amplitude for display. Orange boxed areas are shown in magnified views in (e). Red dots in (e) indicate detected spikes.

At present, I'll limit my comments to the major conceptual points above, though there are also some minor mistakes in the manuscripts. For example:

Line 128/129: "We found spatially clustered sub-group of neurons at the dorsal side…that fired ~70 ms prior to other neurons"

It is the ventral side that fires earlier (see Fig 5d, green arrow vs magenta arrow).

(31) We corrected this typo in our manuscript. (see **line 165** in our revised manuscript)

Reviewer #2:

Remarks to the Author:

Wang et al. developed microscopy optimized for whole brain voltage imaging by using optimized remote focusing and camera system and succeeded in volumetric imaging of voltage changes at about 200 Hz from about 1/3 of the entire neurons in the zebrafish brain at the larval stage. Furthermore, they have identified populations of neurons that showed different response patterns to visual stimuli, presenting interesting aspects of the spatiotemporal dynamics of neuronal populations. This is a significant advancement in voltage imaging of neuronal populations, which is highly demanded and thus is very valuable for future research on neuronal dynamics from a wide area of the brain. However, some areas need improvements.

・A weak point of this study is that the authors presented data from only one fish. How would they validate the results?

(32) In the revised manuscript, we have conducted many more experiments, on additional fish. We have now conducted visual stimulation experiments on a total of 8 fish over 3 separate days. Of these, 3 were excluded from the analysis due to large fish position drift between trials (>2 pixels), possibly because the fish moved slightly. Additionally, one fish exhibited a slow heart rate. We therefore didn't continue trial 2 in that fish. Excluding these 4 fish, we analyzed data from the remaining 4 fish (now referred to as Fish A, B, C, and D). The image quality is consistent across these fish, in terms of the number of ROIs extracted and their SNR (see **Figure 8** above).

We revised the manuscript to include analysis results from all four fish. This includes the spatiotemporal firing patterns elicited by the ultraviolet (UV) stimulus, which demonstrate a high degree of consistency across all fish (**Fig. 4** in the manuscript). We also examined the spatiotemporal firing patterns of spontaneous burst sequences (**Fig. 5** in the manuscript). Here, we observed both consistent and variable features across the four fish. This variation is not unexpected. Since these burst sequences do not correlate with our controlled UV stimulus, it is possible that they reflect the same or different behaviors or internal states. We discuss these in detail in the following paragraphs and on **lines 786-993** of the revised manuscript, and associated figures.

In the analysis, we also made two changes in the analytical method: (1) In the original manuscript, we applied an SNR threshold to exclude ROIs with low SNR prior to clustering. In the revised manuscript, we conducted clustering on all ROIs without SNR thresholding. This is a more generalized approach that is subject to less parameters. (2) Additionally, we used our custom-trained model in Cellpose (Stringer and Pachitariu 2024) (also see **point (4)**) along with manual correction for ROI segmentation, resulting in non-overlapping ROIs (as shown in **Figures 2,19**). For ease of identification, we changed the names of the four fish to Fish A, B, C, and D. Fish B is the Fish 1 in the original manuscript. We updated the text in the manuscript to reflect these changes:

(Starting at **line 832** in the revised manuscript) Regions-of-interest (ROIs) corresponding to putative neurons were segmented using a custom-trained deep learning model based on Cellpose, followed by manual corrections (**see Methods**). This resulted in identification of 17700, 25552, 25464, and 17419 ROIs in Fish A through Fish D, respectively. The segmented ROIs were subsequently processed through VolPy's temporal trace extraction pipeline, which includes background removal, trace denoising, and spike extraction. For spike detection, an adaptive thresholding approach was employed within VolPy.

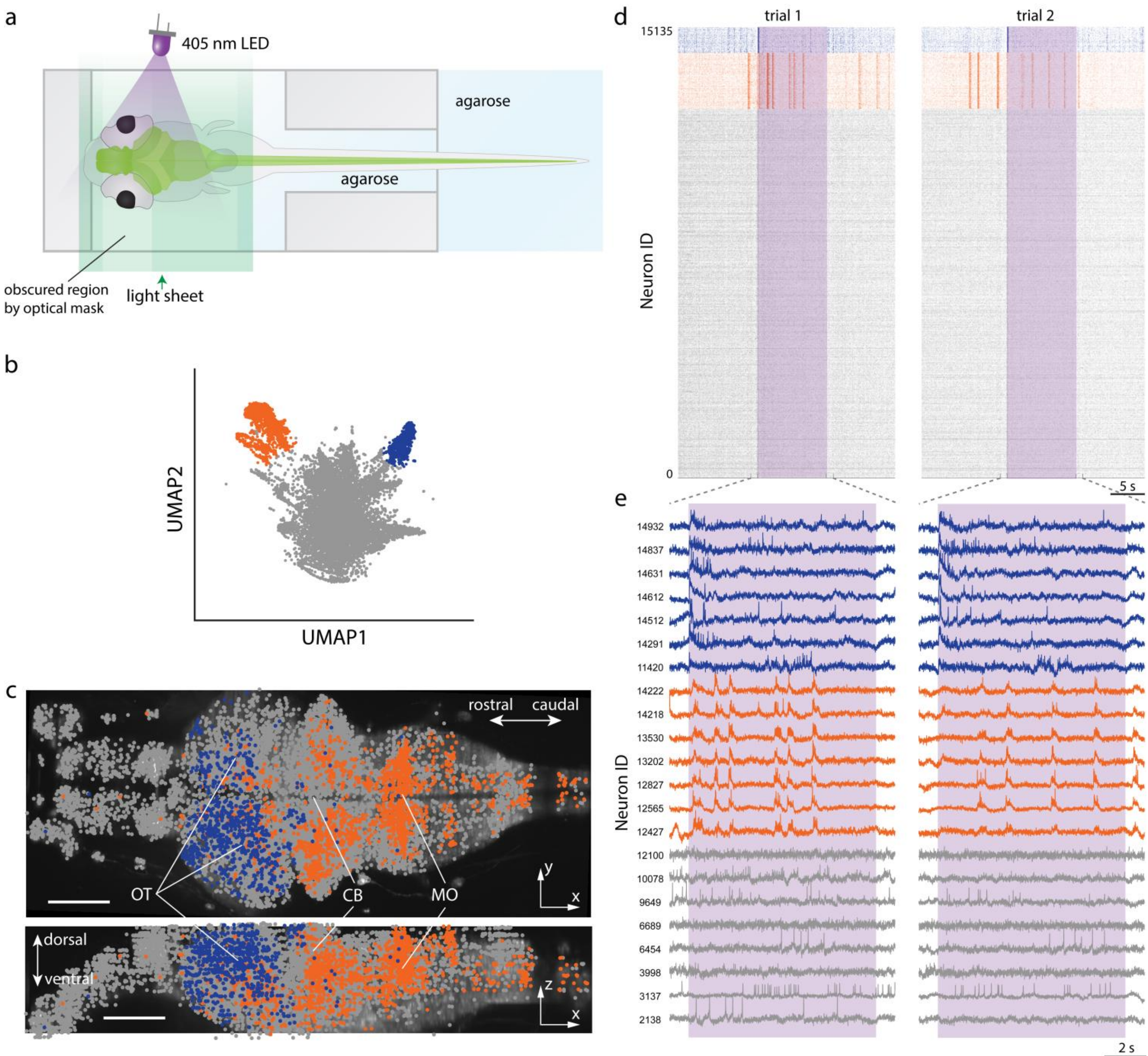

Figure 15 (new Figure 3 in the revised manuscript, corresponding to original Figure 3). Imaging the activity of neurons distributed throughout an entire zebrafish brain during visual stimulation. (a) Light stimulation turns on at a lateral side (here illustrated from the right side, but it can also be from the left side) of the fish for 10s during each trial. **(b)** Spiking activities of all putative neurons in Fish B transformed to a 2D space with UMAP. Neurons in each color group have similar firing patterns. **(c)** Neurons' spatial locations in Fish B. Group 1 neurons (blue) are concentrated in the optic tectum, Group 2 neurons (orange) are mostly located in the hindbrain, Group 3 (gray) neurons are distributed across the brain. OT: optic tectum. CB: cerebellum. MO: medulla oblongata. Scale bar: 100 µm. **(d)** Neuron spike raster plots for Fish B. Four distinct activity patterns are observed in each Fish: Group 1 neurons (blue) have increased activity immediately after UV stimulation onset. Group 2 neurons (orange) have multiple episodes of increased activity throughout the trials. Group 3 neurons (gray) have spontaneous activities throughout the trials. **(e)** Representative traces of neurons' putative subthreshold and spiking activity from (d).

We then apply the same analysis on the ROIs' time series to remove ROIs corrupted by moving stripe artifacts (see point (21)) and clustered the remaining ROIs based on their temporal dynamics. In the revised manuscript, we excluded the beginning and end segments (~1 s) of data from each imaging trial. This was done because we found that Volpy may introduce some distortion at the beginning or end of the signal, due to its filtering process, known as the "edge effect" (Proakis and Manolakis 1995). By discarding these segments, we aimed to minimize the impact of potential edge distortions associated with the digital filters employed by VolPy for processing neural traces. As a result, the green neuron group (Group 4) in **Fig. 3** in the original manuscript, which showed increased intensity in their traces at the beginning of each trial, passively disappeared in the new Figure 3 in the revised manuscript. We discuss the updated results in the following text:

(Starting from **lines 865** in the revised manuscript) We applied UMAP to project the putative neurons onto a 2D manifold (**Fig. 3b, S25b**) and utilized DBSCAN to partition the neurons into distinct clusters. This analysis revealed a specific group of neurons (Group 1, **Fig. 3b**, blue) that demonstrated increased spiking activity following the onset of UV (405 nm) light stimulation during each of the two trials. Notably, this response was triggered by the light turning ON, but not OFF, aligning with previous findings from calcium imaging studies (Bartel et al. 2021). Another cluster (Group 2, **Fig. 3b**, orange) exhibited recurring burst activity throughout the recordings, which appeared uncorrelated with the stimulus. The remaining neurons, which lacked distinct temporal patterns, were classified as Group 3 (**Fig. 3b**, gray). Neurons in different groups exhibited different spatial distributions in the brain. Averaged across all 4 fish, Group 1 neurons were located mostly (~92%) in the optic tectum (**Fig. 3c**, blue). Group 2 neurons were mostly (~90%) located in the hindbrain of the fish (**Fig. 3c**, orange). Group 3 neurons were scattered across the brain (**Fig. 3c**, gray). Please note – we do not intend these groups to be considered as fundamental scientific classes of cell type; they are simply meant to represent the kind of pattern our technology can unveil. Such patterns could lead to hypotheses that could be tested with further investigations, e.g. causal perturbation, and varying behavioral and other contexts.

……

We examined the temporal dynamics of Group 1 neurons in response to the onset of UV stimulation. We found that there were more Group 1 neurons (70%, 79%, 57%, and 67% in Fish A-D, respectively) located on the left side of the OT region than on the right (**Fig. 3c**, **Fig. S9e-h**). In all four fish, the excitation light-sheet illuminated from the left of the brain. In Fish A and B, UV stimulation was delivered from the right side, whereas in Fish C and D, UV stimulation was delivered from the left. These observations suggest that the predominance of neurons on the left side is likely due to the incident direction of the excitation light-sheet. The left side exhibited better SNR (**Fig. S9**), as the light sheet traveled through thinner brain tissue and maintained better quality in this region. In principle, this one-sided heavy neuron distribution could be resolved by implementing dual light-sheet illumination from both sides of the brain.

Next, we examined the temporal structure of Group 1 neurons to identify potential patterns in their activity. We smoothed each Group 1 cell's spike raster with a 100-ms Hanning window to estimate its spiking rate. We then determined the peak firing rate of each Group 1 neuron and its timing relative to the UV stimulation onset, which we called the latency. We ordered the cells by their mean latencies, averaged across the two trials, with smaller IDs indicating earlier timing of the respective neuron's peak activity (**Fig. 4a,e, Fig. S26a,e**). The same ordering persisted across the two trials (confirmed by correlation analysis of the cells' mean latencies across two trials, **Fig. 4b,f, Fig. S26b,f**), revealing a consistent temporal sequence spanning approximately 200 ms in response to the onset of the UV stimulus (**Fig. 4a,e, Fig. S26a,e**).

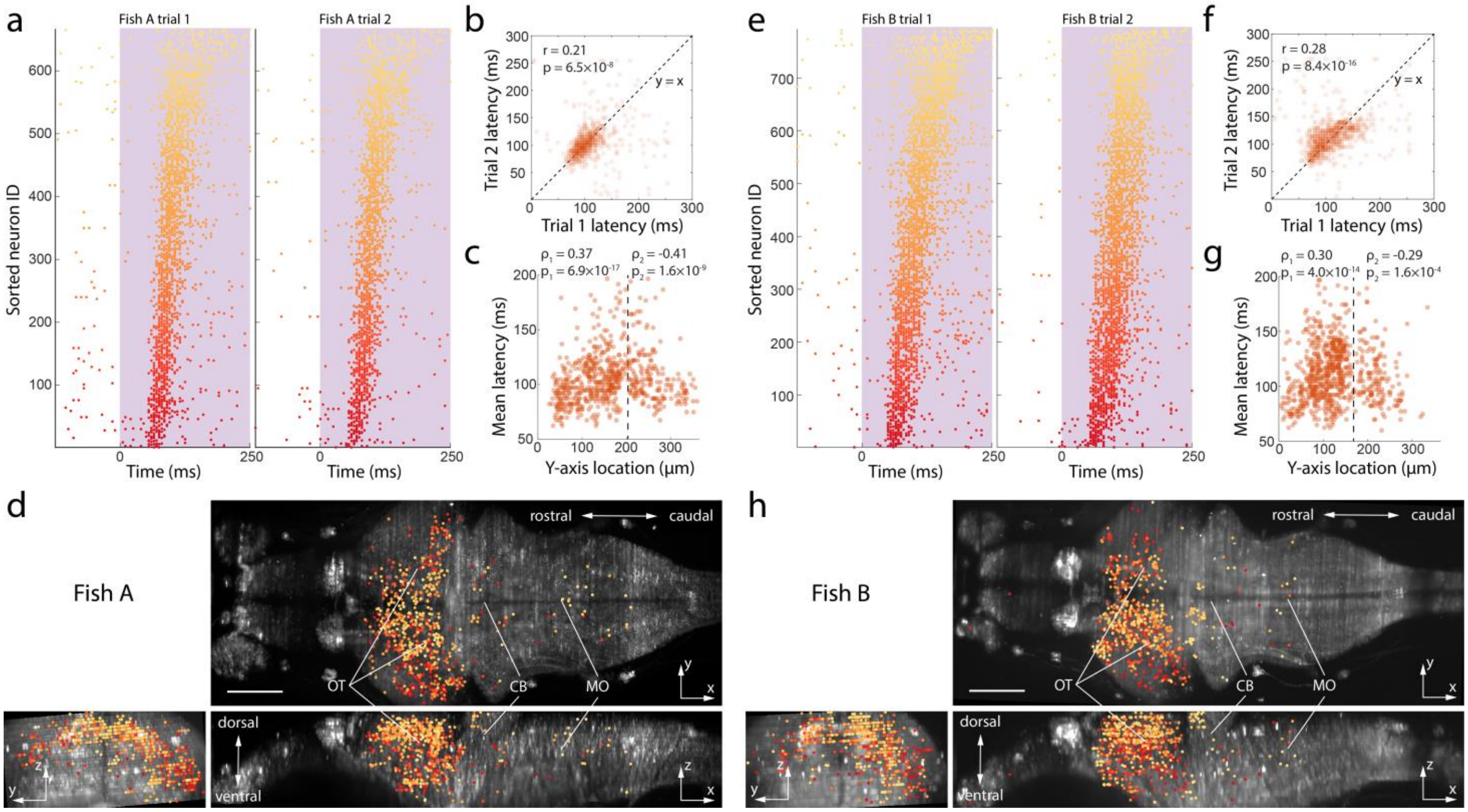

Figure 16 (new Figure 4 in the revised manuscript, corresponding to original Figure 4). Spatial mapping of neurons' firing at different times in stimulus-evoked sequences. (a) The sequential order of Fish A's Group 1 neurons' peak rate latencies in trial 1 is similar in trial 2. Neuron IDs are sorted based on their peak spiking rate latencies averaged across trials 1 and 2. **(b)** Fish A Group 1 neurons' peak spiking rate latencies for trial 1, measured relative to UV onset, have a positive correlation with those of trial 2, as determined by Pearson's correlation (r and p values provided). **(c)** Neuron firing latency vs. neuron location along the y-axis (from left lateral to right lateral in the brain). During the UV stimulus, neurons located more laterally fired earlier than neurons located more medially. The dashed line represents the midline separating the left and right lateral halves of the brain. Spearman's rank correlation was calculated independently for the left and right halves, with ρ and p values provided. **(d)** Group 1 neurons' spatial positions color coded by their firing latency. The majority of Group 1 neurons are located on the lateral-left side of the optic tectum. OT: optic tectum. CB: cerebellum. MO: medulla oblongata. Scale bar: 100 μm. **(e-h)** As in (a-d), for Fish B.

Curiously, the temporal sequence of the neurons' activities corresponded to distinct locations within the brain. We observed the cells' mean latency to peak firing was correlated with neurons' location on the medial-lateral axis (**Fig. 4c, g**). Neurons that fired earlier were located at lateral locations of the OT, whereas those that fired later were situated in more medial locations (**Fig. 4d, h**). The spatiotemporal firing patterns elicited by the UV stimulus were largely consistent across all four fish, on both the left and right sides of the OT. One exception occurred in Fish C on the right lateral side, where neurons in the medial portion of the OT fired earlier in the sequence. This deviation may be influenced by the lower SNR observed on the optic tectum's right side, or factors we did not control in this experiment, e.g., the animal's behavior.

The above addition to the manuscript validated the results of Fig. 4 in the original manuscript in multiple fish. Next we added the following to the manuscript to validate the results of Fig. 5 in the original manuscript.

(Starting from **line 954** in the revised manuscript) Next, we examined the temporal characteristics of Group 2 neurons, present in all four fish, which exhibited multiple bursts within their activity patterns (**Fig. 5**). To define a burst event, we first smoothed each neuron's spike raster with a 70-ms moving Hanning window to estimate its spiking rate. The population spiking rate was then determined by averaging the smoothed raster across all Group 2 neurons (**Fig. 5a**). A burst was detected when the spiking rate increased above a threshold, set at five times that of the average population spiking rate across two trials (**Fig. 5a**). This threshold was chosen

somewhat arbitrarily, to isolate burst events. Upon inspection, we noted that these bursts were not fully synchronized across neurons, but instead occurred at slightly different times, across the population of neurons involved in bursts. Curious to see if the slight differences in timing were consistent, we investigated whether there was a reliable temporal ordering of Group 2 neurons' activities during bursts. We identified each Group 2 neuron's peak firing rate, and timing thereof, within every burst event. We then ordered the Group 2 neurons based on their peak firing rate timing averaged across all the burst events (**Fig. 5a,b,f,g, Fig. S27**). Smaller IDs indicate that the respective neuron's peak activity occurred earlier in the burst event.

Indeed, neurons that fired earlier in the first burst of a series, fired earlier in later bursts of the series (**Fig. 5a,b,f,g, Fig. S27**) ––– there was a consistent ordering of firing of neurons, within these nearly, but not-quite, synchronized bursts. We asked whether the temporal features of Group 2 neurons also corresponded to any spatial characteristics, as in the case of light stimulus-evoked activity, above. We noticed that in Fish B and C, a specific region in the MO contained many neurons that fired early during burst events, and were concentrated within a defined volume (~20×110×40 μm^3, x by y by z) (**Fig. 5c, h,** magenta arrows**, 5d,e,i,j,** magenta color). Through visual inspection, the spatial position of these earliest-firing neurons (**Fig. 5d,e,i,j,** magenta color) coincided with a specific physical neuronal cluster, potentially in rhombomere 5, close to or potentially the same as the cluster of MiD2 reticulospinal neurons[48]. By recording their activity using calcium imaging in fictively swimming larval zebrafish, Chen et. al.[48] found that the MiD2 neurons are involved in bilateral swimming, with no selectivity for turning actions. However, that study could not determine the order of activation across different neuron populations in these motor pathways, due to the limited speed of calcium indicators and the slow optical sampling rate used (2 to 3 Hz). Our study lacks fictive swimming recordings to establish any behavioral correlates of the neuronal activities we observed, so we cannot make any conclusions regarding the precise identity of the neurons we have pinpointed. But if this were a full scientific study, optogenetics, anatomical tracing, and other kinds of experiments could be used to pinpoint the identity and causal role of the neurons observed. For the purposes of the current study, we simply highlight the ability of our technology to reveal the precisely timed operation of this cluster of neurons, relevant to other circuitry.

Curiously, the putative physical cluster of neurons identified in Fish B and C that fired early in the bursts was not observed in Fish A and D (**Fig. S27)**. This variation across fish is not unexpected. Unlike Group 1 neurons, whose activities are elicited by the controlled UV stimulus, the burst patterns of Group 2 neurons do not correlate with the UV stimulus. The distinct burst patterns observed in Fish A and D may reflect different underlying neural computations or behaviors compared with those in Fish B and C. Our experiment did not monitor possible behaviors. Therefore, we were unable to explore the differences between these burst patterns. Nevertheless, the observed consistency and variability in these burst patterns could lead to new hypotheses for future research.

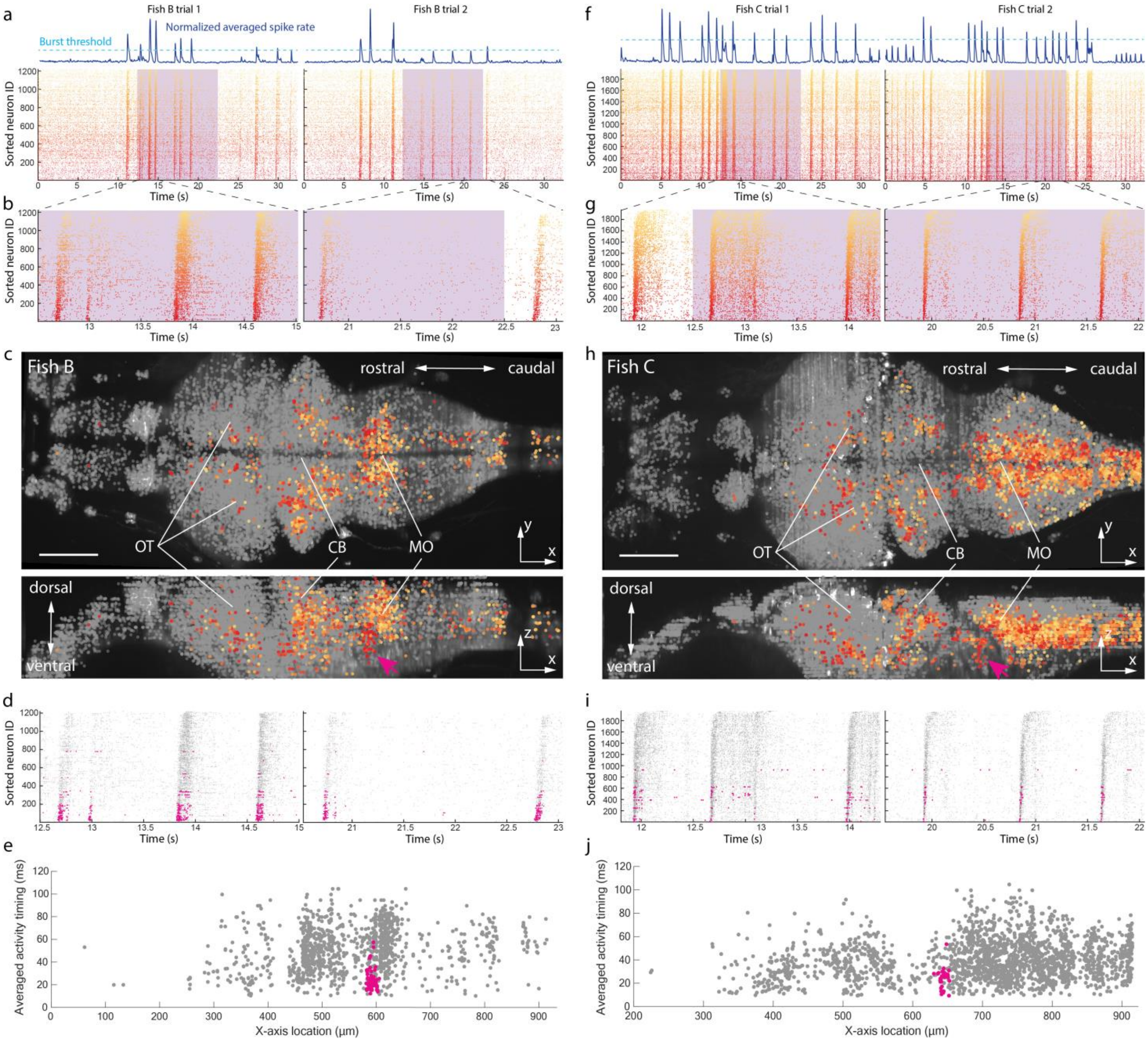

Figure 17 (new Figure 5 in the revised manuscript, corresponding to original Figure 5). Spatial mapping of the timing of activity within burst sequences, across the zebrafish brain. **(a)** Raster plot showing bursting in Group 2 neurons during the light stimulation experiments in Fish B. These neurons correspond to the orange group in Figure 3b's UMAP plot. We define a burst as a period when the average spike rate, taken over an interval of 70 ms, is larger than 5 times the mean spike rate, over the whole trial. The start of each burst was defined as the first time point in the burst when the average spike rate surpassed this threshold. Neurons were sorted by their activation timing, averaged across all burst events. The two trials were separated by a 20 min dark session. The UV stimulus period is indicated in purple. **(b)** Magnified views of the boxed areas in (a), illustrating a consistent sequence of neural activation during bursts. This sequential order is color-coded from deep red (early) to light orange (late). **(c)** 3-D locations of neurons from (a), superimposed on the dorsal (top) and lateral (bottom) brain views (MIPs) of Fish B. The neuron firing sequence is color-coded using the same gradient of colors as in (b). The magenta arrow highlights a putative physical cluster of early firing neurons. OT: optic tectum. CB: cerebellum. MO: medulla oblongata. Scale bar: 100 µm. **(d)** The putative physical cluster of neurons highlighted by the magenta arrow in (c) fired earlier during each burst event. **(e)** Group 2 neurons' x-axis locations and activity timing averaged across all bursts in two trials were plotted as dots, with the putative physical cluster of neurons highlighted by the magenta arrow in (c) shown in magenta, the rest of the neurons in gray. **(f-j)** as in (a-e), for Fish C.

For example, is there any information indicating that there is no particular abnormality in the tissue or cells after recording?

(33) In our experiments, the total excitation power for the fish brain was ~10mW at 515 nm wavelength. This power is much higher (~33.3 ×) than the power (0.3mW) used in previous whole-brain calcium imaging experiments (Ahrens et al. 2013). This higher power is required for our voltage imaging experiments to ensure sufficient photons are collected in each frame, as our imaging rate is 250 times higher than that in whole-brain calcium imaging experiments (Ahrens et al. 2013). We used an opaque optical mask to block illumination to the fish's eyes, which have high absorbance of light due to the pigment cells of the retina. To examine whether this power induced any abnormality to the fish's tissues and cells, we performed immunostaining against four markers of cellular and synaptic health and compared their levels in fish with and without light-sheet exposure. These markers were the apoptotic marker cleaved caspase-3, the DNA damage marker H2AX, the neuronal nucleus marker NeuN, and the synaptic marker Synaptophysin, as we and others have used in prior neurotechnology safety assays (Linghu et al. 2020; Grossman et al. 2017). The exposed fish were illuminated by a light sheet scanning across the fish brain at 200.8 Hz, with a total power of 10.4 mW, for a continuous duration of 70 s. This exposure duration corresponded to the total exposure time of two 35-second imaging trials in our visual stimulation experiments. For H2AX, we fixed the fish ~1 hour after exposure, as H2AX signals reflecting DNA damage can be detected just minutes after damage occurs (Mah, El-Osta, and Karagiannis 2010). For the other three markers, we kept fish overnight before fixing them for immunostaining, as apoptosis, neuron number change and synaptic change are not instantaneous. Our results showed no significant difference in the levels of these markers between the exposed fish and the non-exposed fish (**Figure 18**).

This point has been included in highlighted text in **lines 578-595** in the revised manuscript, and associated figures.

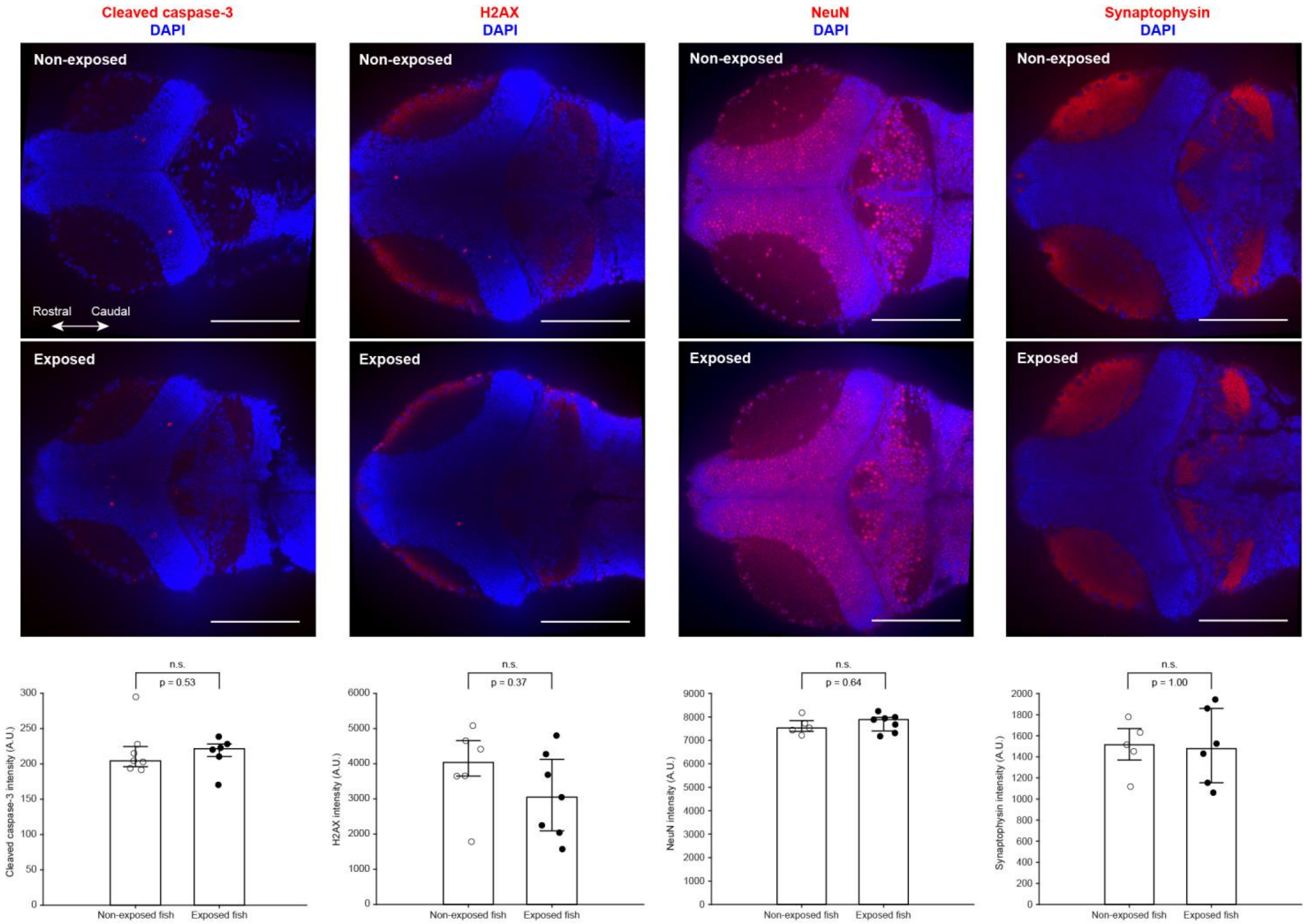

Figure 18 (Figure S19 in the revised manuscript). Light-sheet exposure as in our voltage imaging experiments did not alter the levels of four markers in fish brains. Each column corresponds to a specific cellular and/or synaptic health marker: the apoptotic marker cleaved caspase-3, the DNA damage marker H2AX, the neuronal nucleus marker NeuN, and the synaptic marker synaptophysin, as we have used in previous neurotechnology safety studies (Linghu et al. 2020; Grossman et al. 2017). At the top of each column, representative single-plane immunostaining images display the markers (red) and nuclear DAPI staining (blue) in the brains of exposed fish and non-exposed fish. At the bottom, bar plots show the marker intensities between exposed and non-exposed fish. For the non-exposed and exposed groups in each column, from left to right, n = 7,6,6,7,5,7,5,6 fish, respectively. No significant differences were observed for any marker, as determined by the Wilcoxon rank-sum test. n.s., not significant. P-values for each test are provided. Scale bar: 100 µm.

・How densely distributed are the analyzed neurons (after subtracting the duplicated ROIs)? Providing the nearest neighbor distance would be preferable.

(34) Based on the literature (Hill et al. 2003), for a 7-dpf zebrafish, there are on average 78000 neurons in a volume of approximately 0.20 mm^3. Assuming the neurons are distributed evenly, we can calculate the volume occupied per neuron as 2.6×10^{-6} mm^3. If we assume each neuron occupies a cubic volume, then the length of each side of this cube is the cube root of the volume per neuron, which is 14 µm. This can be interpreted as the average distance between the centroids of neurons in the zebrafish brain.

We calculated neuron density of all putative neurons in Fish A (arbitrarily chosen). To assess the spatial distribution of putative neurons (after artifact-contaminated ROI removal), we measured the average distance

between each putative neuron's centroid and its 10 nearest neighbors' centroids in the same Z-plane, and plotted the results as a histogram (**Figure 19**). The putative neurons in Fish A had separation distances ~31% larger than the literature-based estimate, with a mean distance of 17.8 ± 15.2 µm (mean ± SD, n = 12935 putative neurons). The larger separation distance is possibly due to expression variation in different brain regions (see point (2)), removal of moving-stripe-contaminated ROIs, and reduced image quality in certain brain regions due to tissue scattering (see **Fig. S3** in the original manuscript, now **Fig. S8** in the revised manuscript).

This analysis has been included in the highlighted text in **lines 846-854, 1281-1286** in the manuscript, and associated figures.

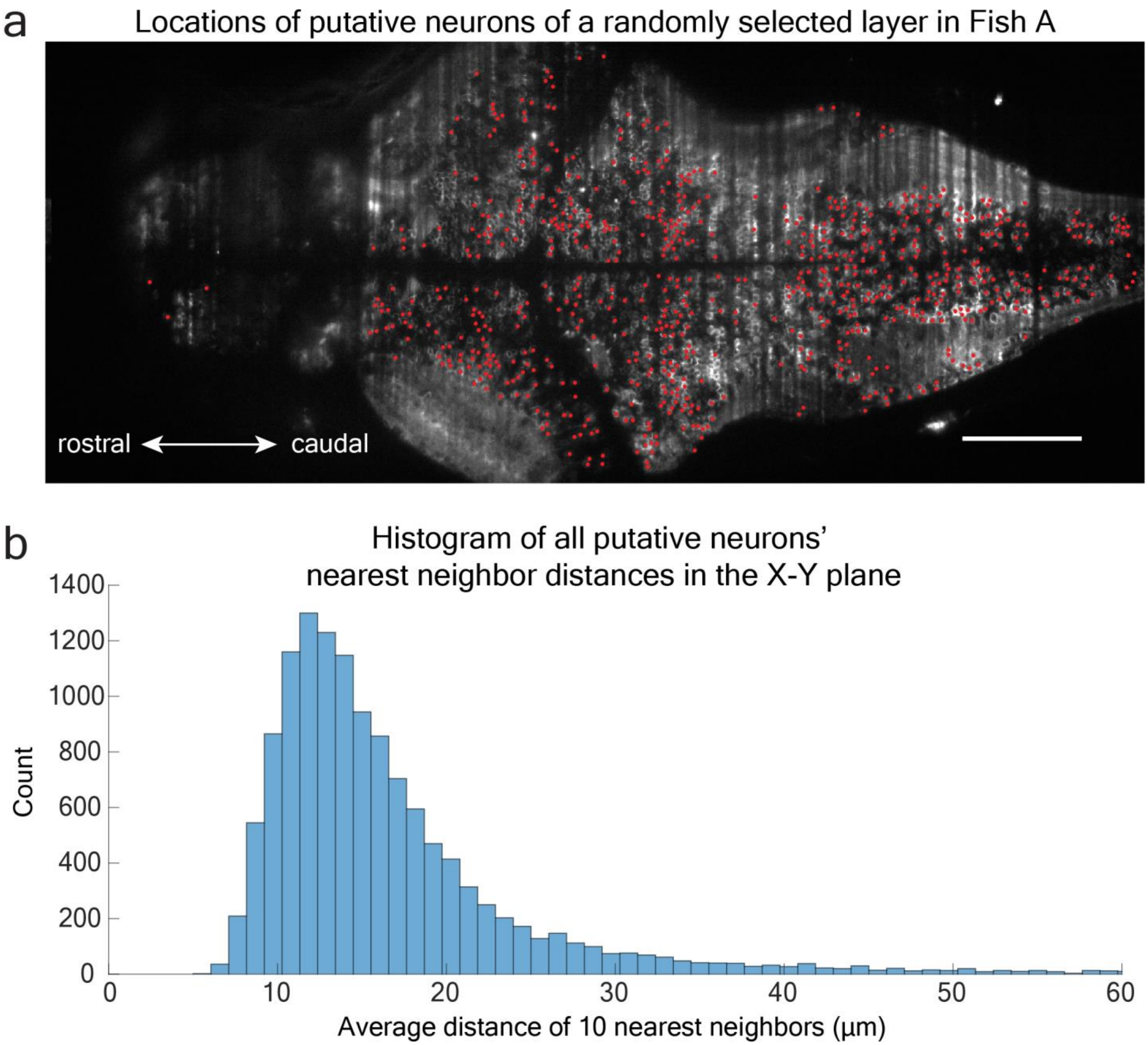

Figure 19 (Figure S21 in the revised manuscript). A quantification of putative neuron density across the x-y planes in Fish A. We calculated the average distance of each putative neuron's centroid to its 10 nearest neighbors' centroids along the X-Y directions within 30 individual z-planes of Fish A. The average distance was 17.8 ± 15.2 µm (mean ± SD, n = 12935 neurons). **(a)** The locations of putative neurons (red dots) of a randomly selected layer in Fish A

are shown. Scale bar: 100 μm. **(b)** Histogram illustrating the counts of putative neurons in Fish A, categorized by their mean distances to the ten nearest neighboring ROIs.

Regarding the pulse-like artifacts, a previous study (Abdelfattah et al., 2019) avoided the shadowing effect by stopping the heart movement for a short term. Although this method has limitations, it would be nice to refer to this approach as one option.

(35) Stopping the fish's heart using forceps is currently not in our animal protocol, and we are concerned it would be unlikely to pass MIT's animal care committee for approval. Alternatively, we stopped the zebrafish heart using 2,3-butanedione monoxime (BDM, a myosin ATPase inhibitor), as utilized in a previous study (Banjo et al. 2013). Please refer to point (15) in our response to the first reviewer for more details. That said, because the effects of BDM on the fish's natural state and brain activity are not well elucidated, here we used BDM only for the purpose of characterizing the sources of the moving stripes, instead of as a general strategy to remove the moving stripe artifacts in all conditions.

・It is desirable to quantify the photobleaching of the recorded neuron populations.

Why did the authors set the duration of the voltage imaging to 35 sec? How long can the recording time be extended?

(36) We chose 35 seconds for each trial, because the UV stimulation was on for a period of 10s, and we wanted to ensure that there was at least 10s of data prior to, and after, UV stimulation.

(37) Although we set each trial's time to be 35s, we were able to continuously image the whole fish brain for a much longer period, which, in principle, is limited by photobleaching and storage. We demonstrated this capability by performing a 200-s recording of the whole zebrafish brain at a 200.8 Hz volume rate (**Figure 20**). We showed traces from two arbitrarily selected neuron ROIs (Cell A, Cell B) from this dataset, and found that voltage spikes (extracted through VolPy) could still be detected at the end of the recording, with somewhat lower amplitudes due to photobleaching (32% lower in Cell A, 6% lower in Cell B). This data and analysis have been included in **lines 810-815** in our manuscript, and associated figures.

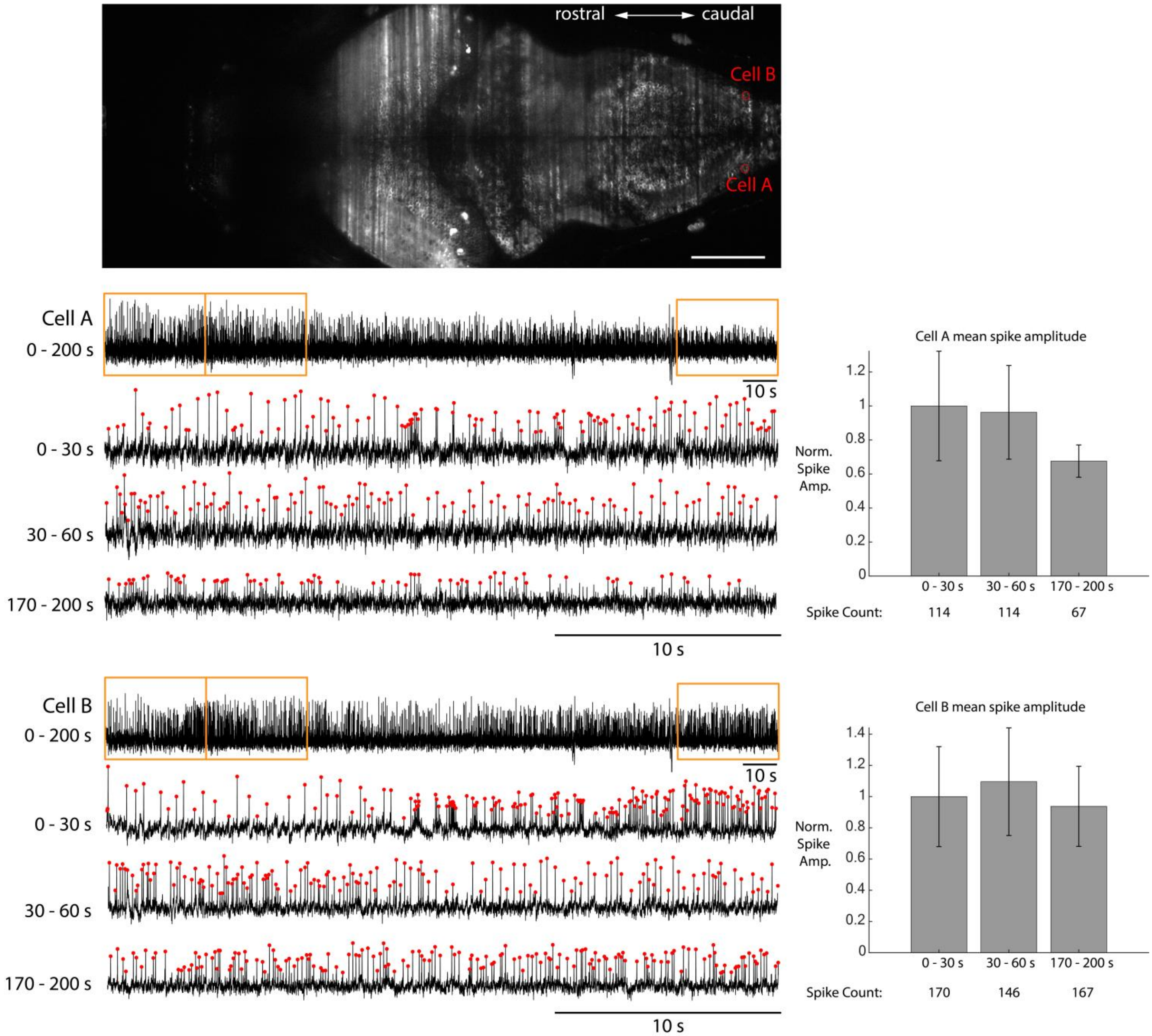

Figure 20 (Figure S22 in the revised manuscript). Voltage activity from a 200-s long imaging trial. The top panel displays a raw image of a randomly selected brain layer (out of a total of 30), with two cells (Cell A and Cell B, arbitrarily selected) marked for neural trace analysis. Below, the full 200-s neural traces of Cell A and Cell B are shown. The first 60 s and the last 30 s of the traces (highlighted with orange boxes in the top trace) are displayed with expanded time scales. Red dots show spikes detected by VolPy. On the right side of the neural traces, bar plots display the spike counts and normalized mean spike amplitudes ± standard deviations for each section (0-30s, 30-60s, and 170-200s). Scale bar in the top panel: 100 µm.

(38) We further quantified the photobleaching curve of Positron2-Kv (stained with JF525) fluorescence in our experiments for a 10-min period of time (**Figure 21**). The fluorescence dropped by 50% after imaging for approximately 210 s. We noticed that in **Figure 20**, the spike amplitude reductions of Cell A and Cell B were slower than the photobleaching curve shown in **Figure 21**, which decreased by 42% over the same time period. Several factors could explain this: 1) The filtering and denoising steps in Volpy may boost spike

amplitudes, counteracting the effect of photobleaching. 2) The photobleaching measurement in **Figure 21** represented an average response, whereas these two cells provided only individual examples.

This result has been included in the highlighted text in **lines 815-822** in the revised manuscript, and associated figures.

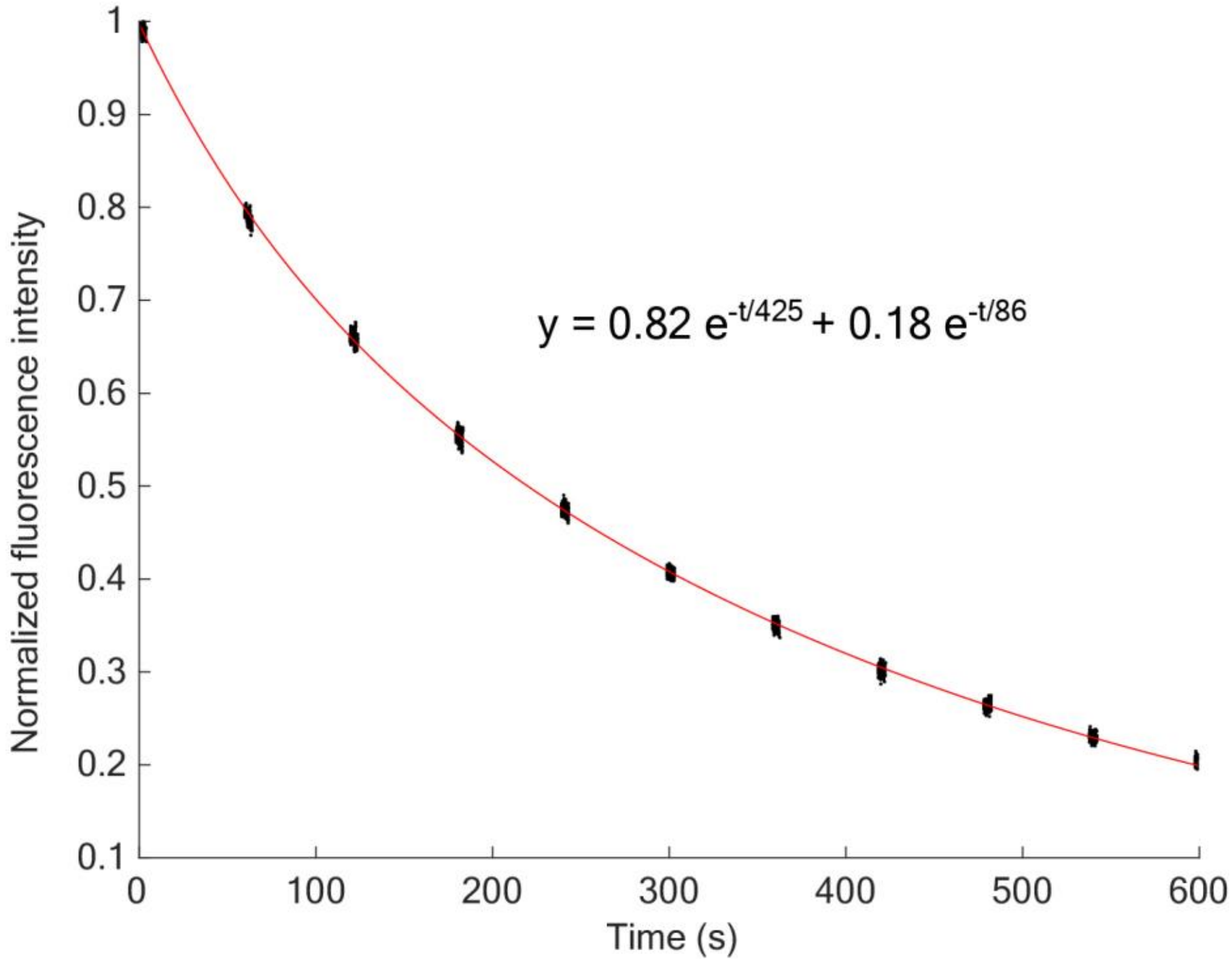

Figure 21 (Figure S23 in the revised manuscript). Normalized photobleaching curve of a zebrafish brain expressing Positron2-Kv indicator stained with JF525. The fish brain was continuously illuminated by a 515-nm light sheet that was scanned at 200.8 Hz, with a total average power of 10.4 mW. To save storage space, we recorded videos of the brain for 4.5 s at 1-min intervals. The measured fluorescent intensities averaged across the imaged brain volume at different time points were plotted as black dots, with a double-exponential fitted curve shown in red. The formula of the fitted curve was also shown.

・line 605: According to the morphological information of the optic tectum, which layers in the retina would correspond to L1 and L2 indicated by the authors? For example, XYZ imaging of the recorded fish with a confocal microscope would allow precise observation of the layered structure of the retina and link it to the voltage information.

(39) The annotated layers L1 and L2 in our original figures were schematic representations of the layered structure of the optic tectum. They were intended to guide non-expert readers in understanding the lateral-medial propagation of neural activity within this region. Our goal was to simplify the complex information flow for enhanced accessibility without oversimplifying the underlying neuroanatomy. To prevent potential misinterpretation of our L1 and L2 annotations as precise morphological maps of the brain by readers, we decided to remove the L1 and L2 diagram (original Figure 4h) from Figure 4 in the revised manuscript.

We have the potential to register our dataset to publicly available atlases (e.g. https://zebrafishexplorer.zib.de/, https://mapzebrain.org/home). However, to the best of our knowledge the existing publicly available atlases do not provide annotated masks for fine-scale subsets of the optic tectum.

Regarding the suggestion to utilize XYZ imaging with a confocal microscope for precise observation of the tectal layers, we conducted additional experiments where we imaged the optic tectum of a zebrafish brain (Fish

C) using a confocal microscope right after two trials of light-sheet voltage imaging. The confocal imaging provided smaller pixel size (0.16 × 0.16 × 1 μm) compared to our light-sheet setup (0.73 × 0.73 × 5.86 μm), allowing for more detailed visualization of the optic tectum. We successfully co-registered the functional light-sheet data with the confocal images and now include overlays of firing neurons' centroids in multiple z-sections of the brain (**Figure 22)**. Although the functional data from this dataset (Fish C) exhibited slightly more complex neuronal firing patterns (see **lines 915-922** in the revised manuscript) compared to Fish A, B, and D, the overall trend—lateral neurons firing before medial ones—remained consistent with our original findings.

While the confocal data did not qualitatively improve our understanding of optic tectum structures, it does show that functional imaging can be augmented with high-resolution structural data. This integration showcases the potential for future research to employ advanced techniques such as expansion microscopy, ExFISH (F. Chen et al. 2016), or ExSEQ (Alon et al. 2021).

We have included the confocal data and the above point in our revised manuscript. (See highlighted text in **lines 942-953** in the revised manuscript, and associated figures.)

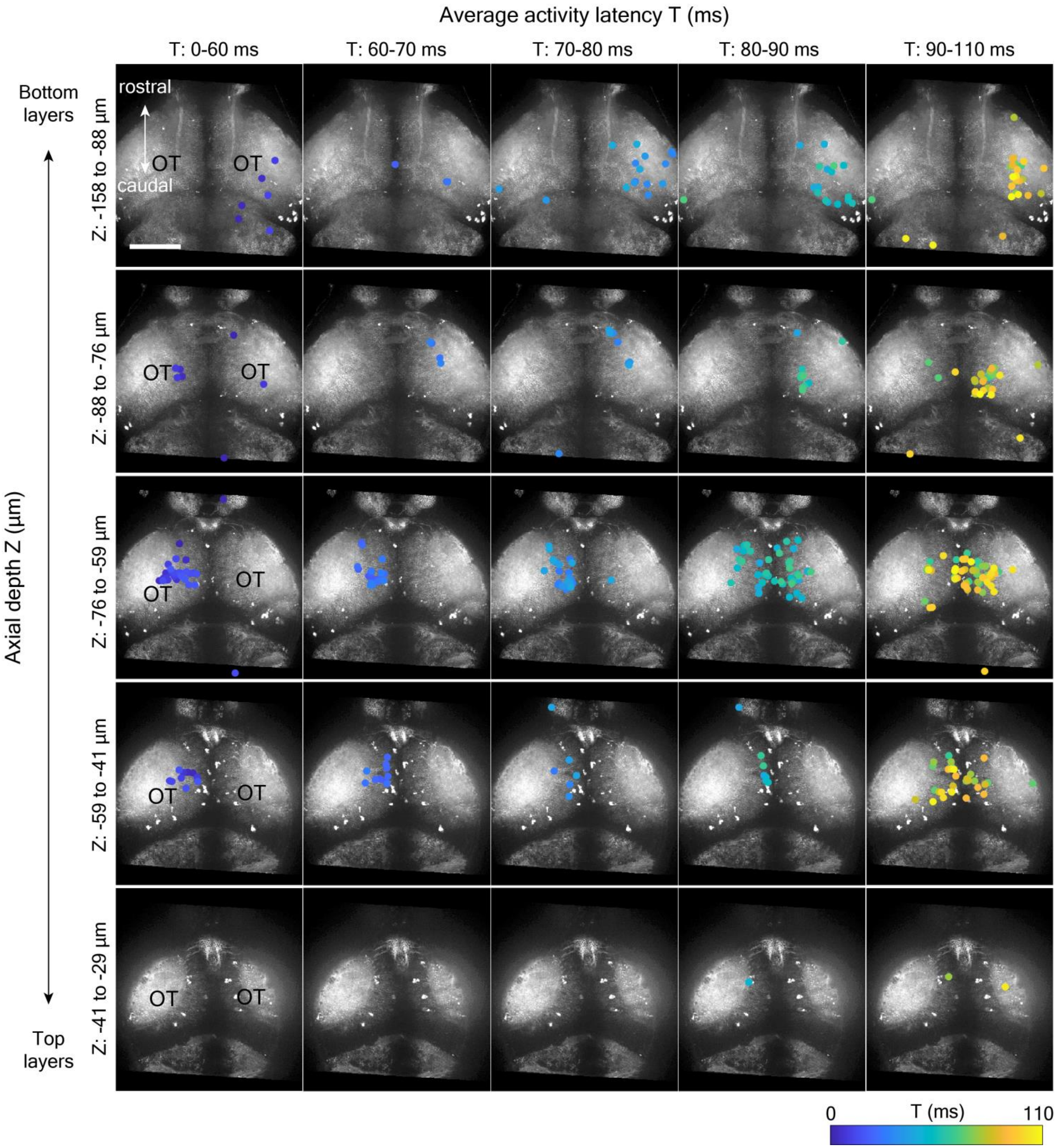

Figure 22 (Figure S30 in the revised manuscript). Locations of Fish C's UV-responding neurons (Group 1 neurons) overlaid with confocal images of the optic tectum region. Each row shows the maximum intensity projection (MIP) of a z-section of the brain of a certain depth range, as indicated on the left of the row. Within each row, from left to right, neurons falling within different activity latency windows (indicated on top of each column) are shown. The depth ranges and latency windows were chosen arbitrarily to better visualize the spatial-temporal correlations of UV-elicited neural activity. Neurons are plotted as dots in colors coded by their activity latency. The bright white dots are occasional neurons, visible in these z-projections but found here and there in all fish, with very high expression levels of GEVIs, for unknown reasons. OT, optic tectum. Scale bar: 100 µm.

・If the authors established the 3XUAS:Positron2-Kv construct, its process should be described in the method section.

(40) We have added the following text in the **Methods** section (**lines 1221-1226**, and **Supplementary Table 3**) in our revised manuscript to describe how we established the 3×UAS:Positron2-Kv construct.

We designed the Positron2-Kv plasmids for micro-injection and synthesized the plasmids through a company (Epoch). In our design, DNA constructs consisting of tandemly linked copies of UAS sequences, the Positron2 gene, and a Kv2.1 soma-targeting sequence were inserted between the two Tol2 arms in a Tol2 plasmid vector. We also inserted Kir2.1 and ER export sequences between the Positron2 gene and the Kv2.1 sequence to facilitate membrane trafficking. The DNA sequences of the Positron2-Kv constructs are provided in the **Supplementary Table 3**.

・In the current manuscript, the detailed design of the 3D-printed sample chamber was not provided. A schematic view of the chamber or a more detailed description is desirable.

(41) We have added a detailed description of the 3D-printed chamber design and a schematic figure (**Figure 23**). The CAD files for the chamber were also included, as supplementary files, in the revised paper. (See highlighted text in **lines 1255-1257, 1427-1433** in the manuscript)

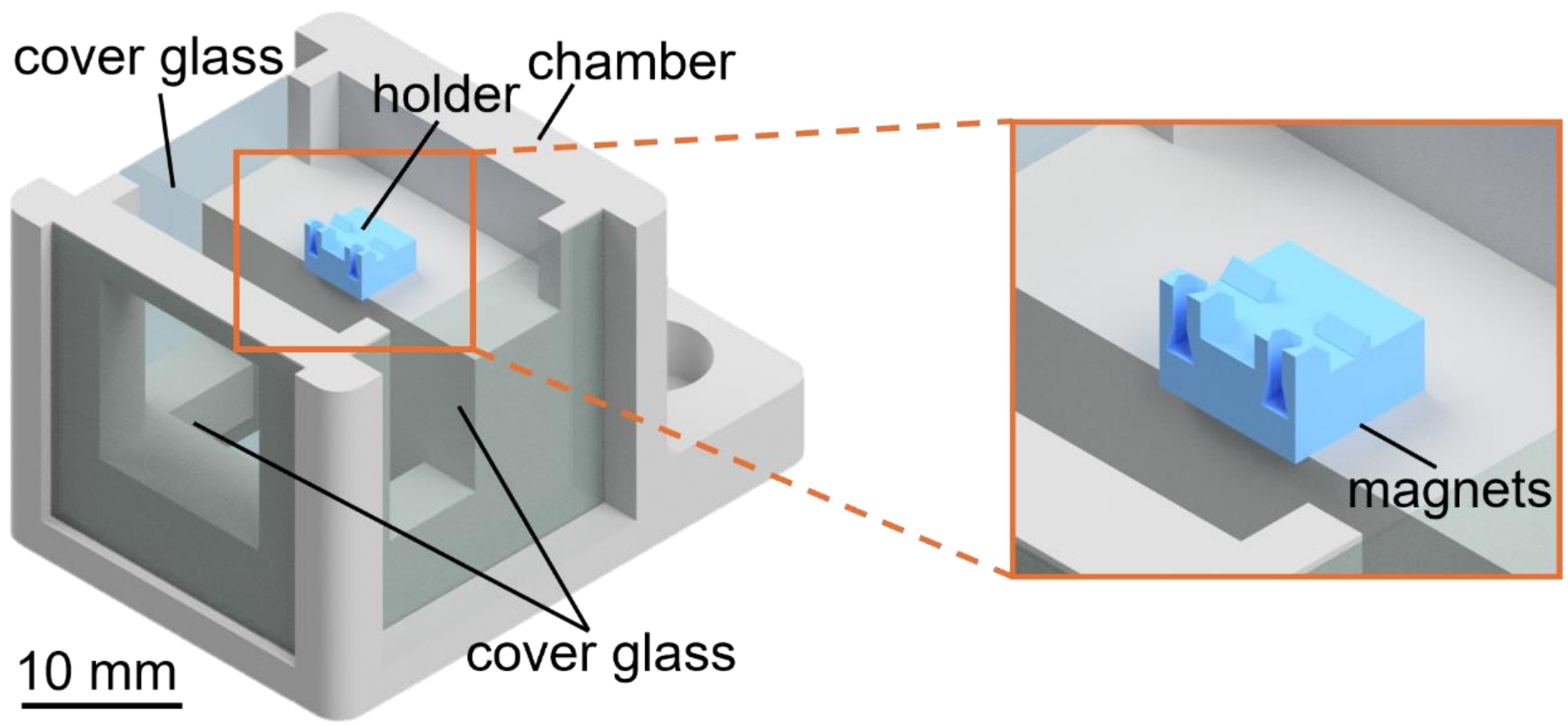

Figure 23 (Figure S28 in the revised manuscript). **Illustration of the fish holder (blue) and chamber (gray).** The holder was attached to the chamber using two magnets. There were two slots on the holder where the fish could be mounted, depending on which side the light sheet came from.

・line 919: The authors mentioned "manual clustering analysis," but please explain how to analyze the data, including any criteria.

(42) In this resubmission, we no longer use an SNR threshold to eliminate ROIs before the analysis, in order to make our analysis method more general and subject to fewer parameters (see point (32)). For the analysis, we first use UMAP and DBSCAN to cluster and remove ROIs with moving stripe artifacts (**Fig. S11** in the revised manuscript**)**. After we removed all the ROIs affected by moving stripe artifacts, we applied UMAP and

DBSCAN again to separate the remaining ROIs into the clusters shown in **Figure 3** in the manuscript. The detailed text is below (and also in the **Methods** section **(lines 1337-1370)** in the manuscript).

Artifact removal: After extracting putative spiking activity from each Region of Interest (ROI), we employed the Uniform Manifold Approximation and Projection (UMAP) algorithm to identify and remove ROIs that may have been influenced by recording artifacts, such as those caused by excitation intensity variation due to blood flow. To achieve this, we first applied a 250-ms moving Hanning window to each ROI's spike raster for smoothing and estimation of its firing rate. The entire recording data was structured into an N × D matrix, where N represented the number of ROIs and D represented the number of temporal samples. This matrix was fed to the UMAP algorithm and transformed into an N × 2 matrix, where each ROI was represented as a point within a 2-dimensional manifold. We then employed the Density-Based Spatial Clustering of Applications with Noise (DBSCAN) algorithm to identify clusters within the 2D representations of ROIs, effectively sorting ROIs with similar temporal spiking patterns into groups. We then visually identified the ROI cluster that was most well-separated from the main cluster in the 2D UMAP representation. We then labeled the ROIs of the identified cluster with a unique cluster ID and removed them from the total set of ROIs. The remaining ROIs were then mapped and clustered again using UMAP DBSCAN for the next iteration. The iteration was continued until the 2D UMAP representation became near Gaussian distributed. Following this clustering step, we scrutinized the temporal patterns and spatial distributions of ROIs within each cluster to detect any indications of artifacts resulting from recording imperfections, which were excluded from further analysis. Our criteria to identify an artifact-contaminated cluster were: 1) the temporal traces of the cluster's ROIs exhibited synchronous pulse-like patterns (**Figure S11**, orange arrows); 2) the spatial distribution of the cluster's ROIs had the same spatial features as the artifact moving stripes or shadows, i.e., the ROIs were concentrated in a region (or regions) that was narrow in the x and z axes (< 100 µm) and spanned from a certain location (possibly in the middle of the brain, as there are blood vessels) to the edge of the brain opposite to the edge of light sheet incidence, along the y axis (light sheet illumination axis) (**Figure S11**, green arrows). A cluster was identified as an artifact cluster only when it satisfied both these criteria. The remaining ROIs, after moving stripe artifact removal, were putatively treated as neurons, sorted into different groups using the same UMAP DBSCAN algorithm, and awaited further analysis.

Clustering based on neuron putative spiking activity: After removing all the ROIs with artifacts, we clustered the rest of the ROI again using UMAP and DBSCAN, based on their temporal characteristics. This analysis revealed a specific group of neurons (Group 1, **Figure 3b**, blue) that demonstrated increased spiking activity following the onset of 405 nm light stimulation during two trials. Notably, this response was triggered by the light turning ON, but not OFF, aligning with previous findings from calcium imaging studies (Bartel et al. 2021). Another cluster (Group 2, **Figure 3b**, orange) exhibited recurring burst activity throughout the recordings, which appeared uncorrelated with the stimulus. The remaining neurons, which lacked distinct temporal patterns, were classified as Group 3 (**Figure 3b**, gray).

· The authors only showed a movie of stripe artifacts. The movie files of spontaneous activity and light-evoked responses also need to be provided.

(43) We included a link at the end of the revised manuscript (https://mitprod-my.sharepoint.com/:f:/g/personal/zgwang_mit_edu/EpNWh5wfTQhKj19x98XoWAwB8IEEceKJEC4p8UXJ592mmg?e=aQ3Xbw, see **"Data Availability"** section) to publicly share the raw videos of individual planes of a representative zebrafish brain (Fish A) during the visual stimulation experiment, and the raw videos of individual planes of the fish brain shown in **Fig. 2** in the manuscript during spontaneous activity imaging.

· Figure 2d: It is better to show enlarged views of the burst firing patterns indicated by black arrowheads

(44) We have included enlarged views of these burst patterns in **Figure 2d** in the revised manuscript.

・Figure 4h: Currently, the diagram is difficult to see. It is preferable to show the schematics more clearly.

(45) We have removed this diagram in the revised paper for two considerations. First, as pointed out by reviewer 1, the one-sided illumination caused an imbalanced distribution of putative neurons on each side of the fish brain (see point **(23)**). Consequently, it is hard to claim that the one-sided heavy distribution of UV-responding neurons in our experiments was due to contralateral UV information processing in the optic tectum. Second, the L1 and L2 lines in Figure 4h of the original manuscript were intended for a general illustration of hypothesized circuity, rather than as a precise morphological map of the brain. In fact, in our single-color, pan-neuronal labeled images, clear boundaries between the morphological layers in the zebrafish optic tectum were not readily discernible, even under a confocal microscope (see point Error! Reference source not found.). To prevent potential misinterpretation by readers, we decided to remove this diagram. We modified our discussion accordingly in the text on the possible circuities underlying the observed UV-responsive patterns (see highlighted text in **lines 865-953** in the revised manuscript, and associated figures).

・line 1238: The authors wrote, “The spike raster of the neurons outside the white dashed box is labeled using a light gray color,” but plots in gray color seem not visible in Figure 5h.

(46) In our revised paper, we removed the white dashed box for this analysis to make this analysis subject to fewer arbitrary parameters. Instead, we plotted the neurons in the magenta arrow pointed cluster in magenta color and the remaining neurons in Group 3 in gray color. We used a darker gray color to make it more visible (see **Figure 5 d,e,i,j** in the revised manuscript).

・The authors often used expressions that are too casual. (eg. line 91 “say”)

(47) We revised line 91 in the original manuscript (now **line 119**) and modified our language in the whole manuscript to make it more formal.

References

Ahrens, Misha B., Michael B. Orger, Drew N. Robson, Jennifer M. Li, and Philipp J. Keller. 2013. “Whole-Brain Functional Imaging at Cellular Resolution Using Light-Sheet Microscopy.” *Nature Methods* 10 (5): 413–20.

Akitake, Courtney M., Michelle Macurak, Marnie E. Halpern, and Mary G. Goll. 2011. “Transgenerational Analysis of Transcriptional Silencing in Zebrafish.” *Developmental Biology* 352 (2): 191–201.

Alon, Shahar, Daniel R. Goodwin, Anubhav Sinha, Asmamaw T. Wassie, Fei Chen, Evan R. Daugharthy, Yosuke Bando, et al. 2021. “Expansion Sequencing: Spatially Precise in Situ Transcriptomics in Intact Biological Systems.” *Science (New York, N.Y.)* 371 (6528). https://doi.org/10.1126/science.aax2656.

Asakawa, Kazuhide, Maximiliano L. Suster, Kanta Mizusawa, Saori Nagayoshi, Tomoya Kotani, Akihiro Urasaki, Yasuyuki Kishimoto, Masahiko Hibi, and Koichi Kawakami. 2008. “Genetic Dissection of Neural Circuits by Tol2 Transposon-Mediated Gal4 Gene and Enhancer Trapping in Zebrafish.” *Proceedings of the National Academy of Sciences of the United States of America* 105 (4): 1255–60.

Bai, Lu, Lin Cong, Ziqi Shi, Yuchen Zhao, Yujie Zhang, Bin Lu, Jing Zhang, et al. 2024. “Volumetric Voltage Imaging of Neuronal Populations in the Mouse Brain by Confocal Light-Field Microscopy.” *Nature Methods* 21 (11): 2160–70.

Banjo, Toshihiro, Janin Grajcarek, Daisuke Yoshino, Hideto Osada, Kota Y. Miyasaka, Yasuyuki S. Kida, Yosuke Ueki, et al. 2013. “Haemodynamically Dependent Valvulogenesis of Zebrafish Heart Is Mediated by Flow-Dependent Expression of MiR-21.” *Nature Communications* 4: 1978.

Bartel, Philipp, Filip K. Janiak, Daniel Osorio, and Tom Baden. 2021. “Colourfulness as a Possible Measure of Object Proximity in the Larval Zebrafish Brain.” *Current Biology: CB* 31 (5): R235–36.

Cai, Changjia, Johannes Friedrich, Amrita Singh, M. Hossein Eybposh, Eftychios A. Pnevmatikakis, Kaspar Podgorski, and Andrea Giovannucci. 2021. “VolPy: Automated and Scalable Analysis Pipelines for Voltage Imaging Datasets.” *PLoS Computational Biology* 17 (4): e1008806.

Chen, Fei, Asmamaw T. Wassie, Allison J. Cote, Anubhav Sinha, Shahar Alon, Shoh Asano, Evan R. Daugharthy, et al. 2016. “Nanoscale Imaging of RNA with Expansion Microscopy.” *Nature Methods* 13 (8): 679–84.

Chen, Xiuye, Yu Mu, Yu Hu, Aaron T. Kuan, Maxim Nikitchenko, Owen Randlett, Alex B. Chen, et al. 2018. “Brain-Wide Organization of Neuronal Activity and Convergent Sensorimotor Transformations in Larval Zebrafish.” *Neuron* 100 (4): 876-890.e5.

Cong, Lin, Zeguan Wang, Yuming Chai, Wei Hang, Chunfeng Shang, Wenbin Yang, Lu Bai, Jiulin Du, Kai Wang, and Quan Wen. 2017. “Rapid Whole Brain Imaging of Neural Activity in Freely Behaving Larval Zebrafish ().” *ELife* 6 (September). https://doi.org/10.7554/eLife.28158.

Dunn, Timothy W., Yu Mu, Sujatha Narayan, Owen Randlett, Eva A. Naumann, Chao-Tsung Yang, Alexander F. Schier, Jeremy Freeman, Florian Engert, and Misha B. Ahrens. 2016. “Brain-Wide Mapping of Neural Activity Controlling Zebrafish Exploratory Locomotion.” *ELife* 5 (March): e12741.

Glaser, Adam K., Ye Chen, Chengbo Yin, Linpeng Wei, Lindsey A. Barner, Nicholas P. Reder, and Jonathan T. C. Liu. 2018. “Multidirectional Digital Scanned Light-Sheet Microscopy Enables Uniform Fluorescence Excitation and Contrast-Enhanced Imaging.” *Scientific Reports* 8 (1): 13878.

Greer, Cody J., and Timothy E. Holy. 2019. “Fast Objective Coupled Planar Illumination Microscopy.” *Nature Communications* 10 (1): 4483.

Grossman, Nir, David Bono, Nina Dedic, Suhasa B. Kodandaramaiah, Andrii Rudenko, Ho-Jun Suk, Antonino M. Cassara, et al. 2017. “Noninvasive Deep Brain Stimulation via Temporally Interfering Electric Fields.” *Cell* 169 (6): 1029-1041.e16.

Hao, Yukun A., Sungmoo Lee, Richard H. Roth, Silvia Natale, Laura Gomez, Jiannis Taxidis, Philipp S. O’Neill, et al. 2024. “A Fast and Responsive Voltage Indicator with Enhanced Sensitivity for Unitary Synaptic Events.” *Neuron* 112 (22): 3680-3696.e8.

Hill, Adrian, C. Vyvyan Howard, Uwe Strahle, and Andrew Cossins. 2003. “Neurodevelopmental Defects in Zebrafish (Danio Rerio) at Environmentally Relevant Dioxin (TCDD) Concentrations.” *Toxicological Sciences: An Official Journal of the Society of Toxicology* 76 (2): 392–99.

Huisken, Jan, and Didier Y. R. Stainier. 2007. “Even Fluorescence Excitation by Multidirectional Selective Plane Illumination Microscopy (MSPIM).” *Optics Letters* 32 (17): 2608–10.

Kannan, Madhuvanthi, Ganesh Vasan, Simon Haziza, Cheng Huang, Radosław Chrapkiewicz, Junjie Luo, Jessica A. Cardin, Mark J. Schnitzer, and Vincent A. Pieribone. 2022. “Dual-Polarity Voltage Imaging of the Concurrent Dynamics of Multiple Neuron Types.” *Science (New York, N.Y.)* 378 (6619): eabm8797.

Kugler, Elisabeth, Ryan Snodgrass, George Bowley, Karen Plant, Jovana Serbanovic-Canic, Noémie Hamilton, Paul C. Evans, Timothy Chico, and Paul Armitage. 2021. “The Effect of Absent Blood Flow on the Zebrafish Cerebral and Trunk Vasculature.” *Vascular Biology (Bristol, England)* 3 (1): 1–16.

Kunst, Michael, Eva Laurell, Nouwar Mokayes, Anna Kramer, Fumi Kubo, António M. Fernandes, Dominique Förster, Marco Dal Maschio, and Herwig Baier. 2019. “A Cellular-Resolution Atlas of the Larval Zebrafish Brain.” *Neuron* 103 (1): 21-38.e5.

Levoy, Marc, Ren Ng, Andrew Adams, Matthew Footer, and Mark Horowitz. 2006. “Light Field Microscopy.” In *ACM SIGGRAPH 2006 Papers on - SIGGRAPH ’06*. New York, New York, USA: ACM Press. https://doi.org/10.1145/1179352.1141976.

Linghu, Changyang, Shannon L. Johnson, Pablo A. Valdes, Or A. Shemesh, Won Min Park, Demian Park, Kiryl D. Piatkevich, et al. 2020. “Spatial Multiplexing of Fluorescent Reporters for Imaging Signaling Network Dynamics.” *Cell* 183 (6): 1682-1698.e24.

Mah, L-J, A. El-Osta, and T. C. Karagiannis. 2010. “GammaH2AX: A Sensitive Molecular Marker of DNA Damage and Repair.” *Leukemia* 24 (4): 679–86.

Miccio, L., P. Memmolo, F. Merola, P. A. Netti, and P. Ferraro. 2015. “Red Blood Cell as an Adaptive Optofluidic Microlens.” *Nature Communications* 6 (March): 6502.

Mu, Yu, Davis V. Bennett, Mikail Rubinov, Sujatha Narayan, Chao-Tsung Yang, Masashi Tanimoto, Brett D. Mensh, Loren L. Looger, and Misha B. Ahrens. 2019. “Glia Accumulate Evidence That Actions Are Futile and Suppress Unsuccessful Behavior.” *Cell* 178 (1): 27-43.e19.

Phil Brooks, F., 3rd, Hunter C. Davis, J. David Wong-Campos, and Adam E. Cohen. 2024. “Optical Constraints on Two-Photon Voltage Imaging.” *Neurophotonics* 11 (3): 035007.

Pnevmatikakis, Eftychios A., and Andrea Giovannucci. 2017. “NoRMCorre: An Online Algorithm for Piecewise Rigid Motion Correction of Calcium Imaging Data.” *Journal of Neuroscience Methods* 291 (November): 83–94.

Prevedel, Robert, Young-Gyu Yoon, Maximilian Hoffmann, Nikita Pak, Gordon Wetzstein, Saul Kato, Tina Schrödel, et al. 2014. “Simultaneous Whole-Animal 3D Imaging of Neuronal Activity Using Light-Field Microscopy.” *Nature Methods* 11 (7): 727–30.

Ricci, Pietro, Vladislav Gavryusev, Caroline Müllenbroich, Lapo Turrini, Giuseppe de Vito, Ludovico Silvestri, Giuseppe Sancataldo, and Francesco Saverio Pavone. 2022. “Removing Striping Artifacts in Light-Sheet Fluorescence Microscopy: A Review.” *Progress in Biophysics and Molecular Biology* 168 (January): 52–65.

Ricci, Pietro, Giuseppe Sancataldo, Vladislav Gavryusev, Alessandra Franceschini, Marie Caroline Müllenbroich, Ludovico Silvestri, and Francesco Saverio Pavone. 2020. “Fast Multi-Directional DSLM for Confocal Detection without Striping Artifacts.” *Biomedical Optics Express* 11 (6): 3111–24.

Sancataldo, Giuseppe, Vladislav Gavryusev, Giuseppe de Vito, Lapo Turrini, Massimiliano Locatelli, Chiara Fornetto, Natascia Tiso, Francesco Vanzi, Ludovico Silvestri, and Francesco Saverio Pavone. 2019. “Flexible Multi-Beam Light-Sheet Fluorescence Microscope for Live Imaging without Striping Artifacts.” *Frontiers in Neuroanatomy* 13 (February): 7.

Scheer, N., and J. A. Campos-Ortega. 1999. “Use of the Gal4-UAS Technique for Targeted Gene Expression in the Zebrafish.” *Mechanisms of Development* 80 (2): 153–58.

Stringer, Carsen, and Marius Pachitariu. 2024. “Cellpose3: One-Click Image Restoration for Improved Cellular Segmentation.” https://doi.org/10.1101/2024.02.10.579780.

Taylor, Michael A., Gilles C. Vanwalleghem, Itia A. Favre-Bulle, and Ethan K. Scott. 2018. “Diffuse Light-Sheet Microscopy for Stripe-Free Calcium Imaging of Neural Populations.” *Journal of Biophotonics* 11 (12): e201800088.

Vladimirov, Nikita, Yu Mu, Takashi Kawashima, Davis V. Bennett, Chao-Tsung Yang, Loren L. Looger, Philipp J. Keller, Jeremy Freeman, and Misha B. Ahrens. 2014. “Light-Sheet Functional Imaging in Fictively Behaving Zebrafish.” *Nature Methods* 11 (9): 883–84.

Wu, Jiamin, Zhi Lu, Dong Jiang, Yuduo Guo, Hui Qiao, Yi Zhang, Tianyi Zhu, et al. 2021. “Iterative Tomography with Digital Adaptive Optics Permits Hour-Long Intravital Observation of 3D Subcellular Dynamics at Millisecond Scale.” *Cell* 184 (12): 3318-3332.e17.

Yoon, Young-Gyu, Zeguan Wang, Nikita Pak, Demian Park, Peilun Dai, Jeong Seuk Kang, Ho-Jun Suk, et al. 2020. “Sparse Decomposition Light-Field Microscopy for High Speed Imaging of Neuronal Activity.” *Optica* 7 (10): 1457.

Zhang, Zhenkun, Lu Bai, Lin Cong, Peng Yu, Tianlei Zhang, Wanzhuo Shi, Funing Li, Jiulin Du, and Kai Wang. 2021. “Imaging Volumetric Dynamics at High Speed in Mouse and Zebrafish Brain with Confocal Light Field Microscopy.” *Nature Biotechnology* 39 (1): 74–83.

Reviewers' Comments:

Reviewer #1:
Remarks to the Author:
The most significant improvement to the manuscript is the addition of the resonant scanners to remove the significant stripe artifacts associated with the initial submission (see Supplemental Figure 16). I commend the authors for making this necessary and critical addition to the particular system under consideration.

With all due respect, we do not think that adding the resonant scanners was the most significant improvement to the manuscript, nor was it the only, or best, way to remove moving stripe artifacts. Indeed, resonant scanners can introduce moving stripe artifacts of their own, where none were before, as noted in our previous submission, and discussed below. Instead, there are multiple valuable ways of removing moving stripe artifacts, which may be preferred in pursuit of different kinds of scientific question.

We first must clarify that data affected by moving stripe artifacts had been computationally removed before all data analyses, in the initial submission. Also, moving stripe artifacts affected a minority of the neurons imaged. (Please note: of stripes in raw images, only the moving ones introduced rapidly-fluctuating temporal artifacts into fluorescence traces in affected brain regions. Other, static, stripes did not affect data quality, any more than other static causes of variability (e.g., inevitable variability of protein expression across cells). Thus, our goal was to eliminate moving stripes, instead of all stripes. In this response, going forward, if we say "stripe" we mean "moving stripe.")

Thus, none of the conclusions of the original paper were affected by moving stripe artifacts. We have made this more clear in the revised manuscript (yellow highlighted text).

In the previous revised paper, in addition to A) increasing the number of animals experimented upon, B) analyzing the health of the animals and showing their well-being, C) proving the moving stripes were caused by blood flow, D) creating a deep learning-based method to automate the segmentation of cells, and E) creating a dual-mirror remote scanning strategy that nearly doubled the system's light efficiency, we devised multiple independent ways to address moving stripe artifacts, in addition to the computational method of identifying and removing data affected by the moving stripe artifact, which we showed to be capable of revealing biologically relevant findings in the original manuscript. We here summarize these innovations:

In the earlier revised manuscript, we presented three workarounds that 1) fully remove, by transient cardiac arrest, moving stripes, 2) suppress (using a resonant scanner) moving stripes, and 3) identify (by a computational strategy) and remove ROIs affected by moving stripes. The resonant scanner method is just one of these options, and each option has its own advantages and limitations. Here we clarify the trade-offs of each method:

(1) Transient cardiac arrest: We verified that moving stripes are from shadows cast by blood cells flowing through vessels in the zebrafish brain (Fig. S14, Fig. S15 and associated text). In past neuroscience studies of larval zebrafish, one strategy that has been used is to simply stop the heart. For example, in two recent studies performing voltage imaging in larval zebrafish (Kawashima et al. 2025; Abdelfattah et al. 2019), forceps were used to immobilize the heart mechanically for up to 30 mins, to remove moving stripes during voltage imaging. In one study (Abdelfattah et al. 2019), heart-stopped fish could respond to visual grating stimuli and fictively swim (as monitored using ventral nerve recording), in a well-established fictive behavior paradigm (Vladimirov et al. 2014), performing similarly to fish with normal heartbeats (Mu et al. 2019; Duque et al. 2025). Thus, heart-stopped fish maintained normal sensory and motor functions. In a second study (Kawashima et al. 2025), heart-stopped fish could not only respond to visual grating stimuli as normally as those with normal heartbeats, but also they could perform serotonin-mediated motor vigor learning as normal, for a period of 30 mins. In summary, heart-stopped fish maintained normal brain function, including complex functions such as learning. Perhaps this should not be too surprising: previous studies showed that larval zebrafish can survive in the total absence of blood flow until 7 days post fertilization, via oxygen diffusion into the body from outside, due to their small size (Kugler et al. 2021; Jin et al. 2007; González-Rosa 2022). Here, we chose to explore whether heart stoppage could eliminate moving stripes with a drug, BDM, for simplicity (Fig. S14b, post-BDM; references for BDM's usage include (Dietrich et al. 2015; Banjo et al. 2013; Foo et al. 2021). In our experiments, briefly immersing the animal in 45mM BDM for 3 minutes completely stopped the heart (Fig. S14b, post-BDM), and circulation was then fully restored upon washout, within minutes (Fig. S14b, post-recovery). Given that forceps-stopping has been shown to be compatible with a range of behaviors, it may be preferred to BDM, which like any drug, could have off-target effects. In the current study, we simply wanted to show that stopping the heart, which is an accepted practice in larval zebrafish neuroscience, would work in the context of our voltage microscopy. Going forward, it is possible that the most elegant way of stopping the heart would be optogenetics (https://www.science.org/doi/full/10.1126/science.1195929), using an opsin to inactivate cardiac contraction with light, so that the heart could be stopped precisely during the period of imaging, or perhaps slowed down in beat frequency so that moving stripes would occur with frequencies slow enough to be cleanly distinguished from neural spikes. In summary, given the larval zebrafish's good tolerance for blood- flow interruption, and empirical studies showing that the sensory, motor, and learning functions of the fish brain remained intact during such short-term heart stoppage (<30 minutes), transient cardiac arrest may be applicable for a wide range of biological studies.

(2) Resonant scanning: The resonant scanner suppresses moving stripes by quickly pivoting the lightsheet, so that the images result from light that has "averaged out" any artifacts because it's passed through a larger area. However, the resonant scanner's speed constraint (8kHz) limited our imaging to only a 120-μm thick volume, insufficient to cover the whole brain. In addition, the speed constraint also increased the axial (z-axis)

scan distance of the system focal plane during a frame exposure (from 2.7 μm to 6.1 μm). This in principle will lead to increased axial (z) motion blur and stronger background in raw images. While in principle, the axial field-of-view and the motion blur issue could be improved by increasing the scanning rate, to recover the axial field-of-view and axial motion blur to the level of our original setup, it would require a scanning rate of 27.1kHz ($8\ kHz \times 3/2 \times 6.1/2.7$). To our knowledge, there isn't a resonant scanner on the market that can scan at this rate, while providing the required aperture (~5mm in our case). Thus the resonant scanners available today, cannot support whole larval zebrafish brain voltage imaging. We would like to note that even for the volume accessible, the resonant scanner only suppressed moving stripes, instead of fully removing them (moving stripes were "averaged out", and only in ~90% of affected ROIs). Perhaps most importantly, because the resonant scanner "averaged out" moving stripes by spreading the light out across broader areas, moving stripes were sometimes *introduced* into previously unaffected areas, creating artifacts where none were before (~1% of all segmented ROIs; Fig. S16, trace 11, Fig. S16g,n). Thus while resonant scanning has promise, and in the future could be of use, it comes with caveats.

(3) Computational detection and ROI exclusion: The unsupervised clustering method from the original manuscript, still used in the revised version, flags stripe-contaminated ROIs and removes them from analysis. It requires no hardware modification or extra experimental preparation. It preserves imaging speed and accommodates whole-brain voltage imaging. But it discards affected ROIs (~25% of putative neurons in our datasets of Fish A-D) and accordingly decreases the density of putative neurons (by ~25%).

These biological, optical, and computational methods to address moving stripes let researchers choose the balance of stripe pattern suppression, spatiotemporal coverage, and experimental duration that best fit each study. For acute (<30 minute) experiments one might opt for transient circulatory arrest. To image a 3D-FOV with small axial range (z-axis range, e.g., <120 μm), one might choose addition of a resonant scanner to suppress stripes. Our goal was to demonstrate voltage imaging at large 3D-FOV using the simplest possible optical and experimental setup. Therefore, we opted for our computational method to detect and eliminate affected ROIs. In our experiments, affected ROIs only accounted for ~25% of all ROIs (in Fish A-D), distributed across the whole brain. Our analyses were based on clean traces from unaffected ROIs, which were the majority (n=12935, 15135, 19039, 15998 neurons, or yields of 73%, 59%, 75%, and 92% of total ROIs, for Fish A-D, respectively), distributed throughout the whole brain. These putative neural traces led to consistent findings and results on sequential neural activity across trials and animals (Figs. 3-5, Figs. S26-S27, and associated text).

We have updated our manuscript (see "**Dealing with the effects of stripe artifacts on the dataset**") to clarify the above points, in yellow highlighted text. We also updated the captions and associated text for the figures, and **Supplementary Table 2** to indicate if and how the moving stripes were addressed for each dataset.

Given the clarifying comparison in Supplemental Fig16, extracting real spikes without the resonant scanner does not seem credible.

As noted above, even without the resonant scanner (RS), the majority of brain regions were not shadowed by moving stripes, and thus traces from these regions were unaffected by the kind of temporal artifacts seen in Fig S16f,m. These clean traces could then be used for credible analysis.

Please note – the traces in Fig. S16f,m only looked noisy because, for traces 1-10, we intentionally selected traces from ROIs affected by moving stripes. (We updated the caption and associated text of Fig. S16 to make this point clearer.) In other words, we were deliberately trying to show the worst case. We did so to show the resonant scanner method's ability to suppress the amplitude of moving stripe-induced temporal artifacts (as seen in Fig. S16f,m). As noted above, such moving stripes affected only ~25% of all segmented ROIs in the brain, and most ROIs (~75%) produced clean traces yielding neurons for spike extraction. Thus, while spike extraction was unreliable in regions shadowed by moving stripes, this effect did not affect the vast majority of the neurons imaged.

As such, one would have expected the authors to update all result sections to datasets collected with the resonant scanner enabled.

We did not repeat our experiments (e.g., the UV experiments) on the setup with the resonant scanner, for the reasons above. In addition, the sequential activity we observed in our UV experiments involved neurons distributed across the full thickness of the brain (Fig. 3c, Fig. 4, Fig. 5, Fig. S26, Fig. S27). Using the setup with the resonant scanner to repeat our experiments (e.g., the UV experiments) will miss neurons in around one-third of the brain on the ventral or dorsal side (or both). While enabling the resonant scanner would rescue some (on the order of ~25%, as noted above, multiplied times the fraction of the total volume that could be imaged) segmented ROIs that would otherwise be discarded due to being shadowed by moving stripes, the reduced axial field-of-view (by ~⅓) would mean a net lower yield than if we performed computational artifact elimination. In the datasets taken on our original setup without a resonant scanner (e.g, datasets of Fish A-D in the UV experiment), we identified and excluded the ROIs affected by moving stripes (see yellow highlighted text, Fig. S11, Fig. S24 in our manuscript). Our findings associated with sequential activity in the brain were based on analysis of the uncontaminated neurons (see yellow highlighted text).

However, it's unclear to me which figures in the revised manuscript are using the artifact-laden data from the original submission and which ones are using the new system (Fig. 2a in the revised manuscript still clearly show the artifacts in question).

The stripe patterns from the animals in our raw light-sheet images include stationary stripes and moving stripes. Only moving stripes introduce fast-varying temporal artifacts to the fluorescence baseline, and in a minority of regions in the brain. In our original and revised manuscripts, ROIs

in these affected regions were excluded from analysis, and our findings were based on clean traces of neurons that were not affected by moving stripes.

In Figure 2a, we displayed stitched raw light-sheet images without any further processing, because we wanted to show, in full transparency, what one would expect to see in the raw images, such as unlabeled dark regions (blue arrows), and stripes (red arrows). But when we processed the raw data in Figure 2, we excluded all the moving stripe-affected ROIs. All the traces shown in Figure 2 were from neurons that were unaffected by moving stripes.

We have added two columns in **Supplementary Table 2** to indicate how the effect of moving stripes was alleviated for the datasets in each figure.

If the authors have now switched to the resonant scanner, then a number of supplemental figures related to artifact removal (e.g. Supplemental Figure 10, 11, 14, 15) are no longer necessary, significantly shortening the number of supplemental figures.

Actually, these figures are important because they characterize the moving stripes. And as noted, the resonant scanner can introduce moving stripes for neurons which did not have them before (see Fig. S16, trace 11, Fig. S16g,n) - it is not a “cure all”. Fig. S10 shows how moving stripes caused pulse-like temporal artifacts, and help with understanding of the moving stripes. Fig. S11 shows clustered raw ROIs traces affected and unaffected by moving stripes and helps clarify how affected ROIs were computationally identified. Fig. S14 and Fig. S15 characterized the root cause of moving stripes as blood flow, and demonstrated that temporarily stopping the heart can completely eliminate the moving stripes.

Clarification of the above point is needed for further evaluation of the manuscript. If the data and analyses have not been updated to the version with the RS enabled, then the authors should do so and the manuscript can be evaluated based on the revised data/analyses. If the authors have already updated all figures following the addition of the RS scanner, please state this clearly.

As discussed in the points above, there are many sacrifices incurred by using the resonant scanner - and updating the manuscript with only resonant scanner data would sacrifice a great deal of neuron yield, and incur additional optical complexity and even sometimes additional artifacts. In addition, all the analyses shown were with computational removal of the moving stripes.

REFERENCES

Abdelfattah, Ahmed S., Takashi Kawashima, Amrita Singh, Ondrej Novak, Hui Liu, Yichun Shuai, Yi-Chieh Huang, et al. 2019. “Bright and Photostable Chemigenetic Indicators for Extended in Vivo Voltage Imaging.” *Science* 365 (6454): 699–704.

Banjo, Toshihiro, Janin Grajcarek, Daisuke Yoshino, Hideto Osada, Kota Y. Miyasaka, Yasuyuki S.

Kida, Yosuke Ueki, et al. 2013. “Haemodynamically Dependent Valvulogenesis of Zebrafish Heart Is Mediated by Flow-Dependent Expression of miR-21.” *Nature Communications* 4:1978.
Dietrich, Ann-Christin, Verónica A. Lombardo, Justus Veerkamp, Florian Priller, and Salim Abdelilah-Seyfried. 2015. “Blood Flow and Bmp Signaling Control Endocardial Chamber Morphogenesis.” *Developmental Cell* 32 (3): 387.
Foo, Yoke Yin, Efthymios Motakis, Zenia Tiang, Shuhao Shen, Jason Kuan Han Lai, Wei Xuan Chan, Hadi Wiputra, et al. 2021. “Effects of Extended Pharmacological Disruption of Zebrafish Embryonic Heart Biomechanical Environment on Cardiac Function, Morphology, and Gene Expression.” *Developmental Dynamics: An Official Publication of the American Association of Anatomists* 250 (12): 1759–77.
González-Rosa, Juan Manuel. 2022. “Zebrafish Models of Cardiac Disease: From Fortuitous Mutants to Precision Medicine.” *Circulation Research* 130 (12): 1803–26.
Jin, Suk-Won, Wiebke Herzog, Massimo M. Santoro, Tracy S. Mitchell, Julie Frantsve, Benno Jungblut, Dimitris Beis, et al. 2007. “A Transgene-Assisted Genetic Screen Identifies Essential Regulators of Vascular Development in Vertebrate Embryos.” *Developmental Biology* 307 (1): 29–42.
Kawashima, Takashi, Ziqiang Wei, Ravid Haruvi, Inbal Shainer, Sujatha Narayan, Herwig Baier, and Misha B. Ahrens. 2025. “Voltage Imaging Reveals Circuit Computations in the Raphe Underlying Serotonin-Mediated Motor Vigor Learning.” *Neuron*, June. https://doi.org/10.1016/j.neuron.2025.05.017.
Kugler, Elisabeth, Ryan Snodgrass, George Bowley, Karen Plant, Jovana Serbanovic-Canic, Noémie Hamilton, Paul C. Evans, Timothy Chico, and Paul Armitage. 2021. “The Effect of Absent Blood Flow on the Zebrafish Cerebral and Trunk Vasculature.” *Vascular Biology (Bristol, England)* 3 (1): 1–16.

Reviewer’s comments.

Our responses.

Reviewer #1:

Remarks to the Author:

In summary, the authors present two implementations of light sheet microscopy for voltage imaging across the zebrafish brain. While the authors have made substantial and useful technological progress, both approaches are constrained by significant technical limitations.

The technical limitations mentioned here are not unique to our technique, but challenges that are common to light-sheet imaging more broadly (1). The goal of this work is not to resolve all existing technical limitations of light-sheet imaging (which arguably remains beyond the scope of any single study), but rather to show that voltage imaging of a substantial fraction of the neurons distributed throughout an entire vertebrate brain, is now possible. To achieve this capability, we incorporated multiple technical innovations (see Supplementary Note 12) that collectively increase the achievable imaging rate by nearly two orders of magnitude compared to conventional light-sheet approaches used for zebrafish brain imaging (2, 3), while maintaining cellular resolution and sufficient sensitivity to detect voltage signals. These are non-trivial technical improvements and mark a key milestone towards whole-brain voltage imaging.

The first implementation has less axial blurring (2.7 µm), but approximately 25% of ROIs are affected by moving stripe artifacts. These ROIs are excluded from analysis (“Our analyses were based on clean traces from unaffected ROIs”).

The moving stripe artifacts are a common issue in light-sheet imaging, and have been seen in previous zebrafish imaging studies (1, 2). In this work, we made substantial progress towards mitigating their impact: we have shown, through multiple control experiments, that these moving stripes came from the animal, more specifically, the blood flow, rather than from the hardware (ED_Figs. 2-4). We further show both biological and computational strategies to address these moving stripes (Supplementary Note 4).

While the authors invest considerable effort in computational corrections, they do not incorporate a second reference channel, which is a widely used solution in whole-brain imaging of worms.

We did not implement the ratiometric approach suggested by the reviewer, because it may not be able to efficiently correct the GEVI baseline intensity fluctuation caused by moving artifacts in our case. We image millisecond-scale GEVI signals at high-intensity excitation, which differs substantially from whole-brain calcium imaging in worms. In our case, the intensity of the reference fluorophore may not scale proportionally to the fluctuations of the GEVI intensity, due to fluorescence saturation of either fluorophore at high-intensity excitation. We estimate that in our experiments, the GEVI reached approximately 40% of its maximum fluorescence intensity. At this level, the GEVI's baseline intensity does not change proportionally to the excitation intensity.

Although ratiometric approaches could be a backup plan in the case where the stripe artifacts cannot be addressed using other approaches, we would like to note that the ratiometric approach is not an ideal solution to correct moving stripe artifacts, for several additional reasons: (1) First, crosstalk of the reference fluorescence and the GEVI fluorescence could occur due to emission spectrum overlap. It is difficult to find a reference fluorophore that can be efficiently excited with the same excitation light as for the GEVI fluorophore, while having a non-overlapping emission spectrum. Alternatively, the overlapped part of the spectrum could be filtered out using emission filters, leading to undesired light loss. (2) Second, dual channel imaging requires doubling the number of cameras, increasing the cost, the complexity of the hardware, and the amount of data to analyze. (3) Third, the reference fluorophore will occupy a precious color channel that could otherwise be used for other applications, such as optogenetics or fluorescence indicators of other neural signals (e.g., neurotransmitters).

This discussion was already in the last draft.

The second implementation incorporates a resonant scanner but introduces further constraints, including a more limited axial range (120 μm) and greater axial blurring (6.1 μm), which increases susceptibility to motion-related artifacts.

The resonant scanner implementation efficiently mitigated stripe artifacts (ED_Fig.5), while introducing certain trade-offs associated with the limited resonant scanner frequency (8kHz). We demonstrated this implementation not as a "cure-for-all" solution for our current experiments, but for two main reasons: 1) first, it provides a hardware-based stripe mitigation method that is complementary to biological and computational methods, and may offer unique advantages when imaging a thin volume (<120 μm); 2) second, the observed trade-offs are not fundamental to the approach. In the future, in principle, one could extend the axial range to cover the entire brain and to reduce the axial blurring by employing faster resonant scanners and optimized axial scanning curves that inherently minimize axial blur (e.g., staircase-like scanning functions).

Neither implementation achieves "whole brain" imaging.

We achieved imaging of up to ~1/4 neurons free of moving-stripe artifacts and distributed across the entire brain. In this paper, we revised our language to accurately describe this capability while clearly distinguish it with true "whole-brain voltage imaging".

Notably, the analyses of responses to UV stimuli presented in the manuscript also do not require whole-brain imaging.

It is true that in our experiments, the UV-responsive neurons were found primarily located in the optical tectum. However, without imaging neurons distributed throughout the brain, we wouldn't have mapped their locations and assessed if other brain regions were involved. Our technique allows us to observe the UV-stimuli responses without the risk of missing involved brain regions. Recording neurons within a confined volume, as suggested by the reviewer, would only make sense after first identifying the locations of these responsive neurons through brain-wide imaging.

Comparable conclusions could be obtained by sequentially recording a limited number of axial planes and assessing response latencies.

We note that this paper does not intend to establish any biological conclusions. We present the UV-experiment results only to show the kind of observations and hypotheses that can be produced with our technique.

Overall, the manuscript provides a detailed and thorough characterization of both approaches. There is no need to over-claim about achieving "whole brain" imaging. As echoed by another reviewer, the frequent mention of "whole-brain" or "entire brain" imaging should be removed from the title and text. The method described achieves voltage recording of 1/6-1/4 of neurons across the brain, as stated in the manuscript: "The maximum putative neuron number in our data was thus 1/6 to 1/4 of the estimated neuron number (~78000) in a 7-dpf fish".

Our method can image the voltage of up to ~1/4 neurons free of moving-stripe artifacts and distributed across the entire brains. We have revised our language carefully to make this clearly distinguished with "whole-brain imaging" and "voltage imaging across the entire brain".

Reviewer #2:

None

1. P. Ricci, V. Gavryusev, C. Müllenbroich, L. Turrini, G. de Vito, L. Silvestri, G. Sancataldo, F. S. Pavone, Removing striping artifacts in light-sheet fluorescence microscopy: a review. *Prog. Biophys. Mol. Biol.* **168**, 52–65 (2022).

2. M. B. Ahrens, M. B. Orger, D. N. Robson, J. M. Li, P. J. Keller, Whole-brain functional imaging at cellular resolution using light-sheet microscopy. *Nat. Methods* **10**, 413–420 (2013).

3. N. Vladimirov, Y. Mu, T. Kawashima, D. V. Bennett, C.-T. Yang, L. L. Looger, P. J. Keller, J. Freeman, M. B. Ahrens, Light-sheet functional imaging in fictively behaving zebrafish. *Nat. Methods* **11**, 883–884 (2014).
